# Supplementary material for: Synthesis and Characterization of New Boron Compounds Using Reaction of Diazonium Tetraphenylborate with Enaminoamides
Source: Molecules. 2022 Jan 7;27(2):367. doi: 10.3390/molecules27020367 (PMC8782005; doi:10.3390/molecules27020367)
Supplement: Supplementary file 1 [file molecules-27-00367-s001.zip › molecules-1528609-supplementary.pdf]

SUPLEMENTARY MATERIALS

Synthesis and characterization of new boron compounds using reaction of diazonium tetraphenylborate with enaminoamides

Markéta Svobodová [1*], Jan Svoboda [1], Bing-Han Li [2], Valerio Bertolasi [3], Luboš Socha [1], Miloš Sedlák [1], Lukáš Marek [1]

[1] Faculty of Chemical Technology, Institute of Organic Chemistry and Technology, University of Pardubice, Studentská 573, 532 10 Pardubice, Czech Republic; jan.svoboda@upce.cz (J.S.); st46953@upce.cz (L.S.); milos.sedlak@upce.cz (M.S.); lukas.marek3@student.upce.cz (L.M.)
[2] Department of Chemistry, National Tsing Hua University, Hsinchu 30013, Taiwan; w6520325@hotmail.com
[3] Department of Chemical and Pharmaceutical Sciences, University of Ferrara, Via L. Borsari 46, 44121 Ferrara, Italy; m38@unife.it
* Correspondence: marketa.svobodova@upce.cz

Summary

1. Experimental 7
1. 1 General Information 7
Scheme S1. Preparation of starting compounds. 8
2. NMR spectra 9
2.1 Triazaborinone 5a 9
Figure S1. 400 MHz ^{1}H NMR of compound **5a** in DMSO-d_6 9
Figure S2. 400 MHz 1D-NOESY (mixing time d8 = 800 ms, relaxation delay d1 = 2s) of compound **5a** in DMSO-d_6 10
Figure S3. 100 MHz ^{13}C NMR of compound **5a** in DMSO-d_6 10
Figure S4. 160 MHz ^{11}B NMR of compound **5a** in $CDCl_3$ 11
Figure S5. 500 MHz ^{1}H–^{15}N HMBC NMR of compound **5a** in DMSO-d_6 11
2.2 Triazaborinone 5b 12
Figure S6. 500 MHz ^{1}H NMR of compound **5b** in $CDCl_3$ 12
Figure S7. 400 MHz 1D-NOESY (mixing time d8 = 800 ms, relaxation delay d1 = 2s) of compound **5b** in $CDCl_3$ ... 13
Figure S8. 125 MHz ^{13}C NMR of compound **5b** in $CDCl_3$ 13
Figure S9. 128 MHz ^{11}B NMR of compound **5a** in $CDCl_3$ 14
Figure S10. 50 MHz ^{15}N NMR of compound **5a** in DMSO-d_6 14
Figure S11. 500 MHz ^{1}H–^{15}N HMBC NMR of compound **5b** in $CDCl_3$ 15
2.3 Triazaborinone 5c 16
Figure S12. 500 MHz ^{1}H NMR of compound **5c** in $CDCl_3$ 16
Figure S13. 125 MHz ^{13}C NMR of compound **5c** in $CDCl_3$ 17
Figure S14. 160 MHz ^{11}B NMR of compound **5c** in $CDCl_3$ 17
Figure S15. 500 MHz ^{1}H–^{15}N HMBC NMR of compound **5c** in $CDCl_3$ 18
2.4 Triazaborinone 5d 19

Figure S16. 500 MHz ^{1}H NMR of compound **5d** in $CDCl_3$ 19
Figure S17. 125 MHz ^{13}C APT NMR of compound **5d** in $CDCl_3$ 20
Figure S18. 160 MHz ^{11}B NMR of compound **5c** in $CDCl_3$ 20
Figure S19. 50 MHz ^{15}N NMR of compound **5d** in $CDCl_3$ 21
Figure S20. 500 MHz ^{1}H–^{15}N HMBC NMR of compound **5d** in $CDCl_3$ 21
2.5 Triazaborinone 5g 22
Figure S21. 500 MHz ^{1}H NMR of compound **5g** in $CDCl_3$ 22
Figure S22. 400 MHz 1D-NOESY (mixing time d8 = 800 ms, relaxation delay d1 = 2s) of compound **5g** in $CDCl_3$. 23
Figure S23. 125 MHz ^{13}C NMR of compound **5g** in $CDCl_3$ 23
Figure S24. 160 MHz ^{11}B NMR of compound **5g** in $CDCl_3$ 24
Figure S25. 50 MHz ^{15}N NMR of compound **5g** in $CDCl_3$ 24
Figure S26. 500 MHz ^{1}H–^{15}N HMBC NMR of compound **5g** in $CDCl_3$ 25
2.6 Triazaborinone 5h 26
Figure S27. 500 MHz ^{1}H NMR of compound **5h** in $CDCl_3$ 26
Figure S28. 125 MHz ^{13}C NMR of compound **5h** in $CDCl_3$ 27
Figure S29. 160 MHz ^{11}B NMR of compound **5h** in $CDCl_3$ 27
Figure S30. 50 MHz ^{15}N NMR of compound **5h** in $CDCl_3$ 28
Figure S31. 500 MHz ^{1}H–^{15}N HMBC NMR of compound **5h** in $CDCl_3$ 28
2.7 Oxazaborine 6a 29
Figure S32. 500 MHz ^{1}H NMR of compound **6a** in $CDCl_3$ 29
Figure S33. 125 MHz ^{13}C NMR of compound **6a** in $CDCl_3$ 30
Figure S34. 160 MHz ^{11}B NMR of compound **6a** in $CDCl_3$ 30
Figure S35. 500 MHz ^{1}H–^{15}N HMBC NMR of compound **6a** in $CDCl_3$ 31
2.8 Oxazaborine 6e 32
Figure S36. 500 MHz ^{1}H NMR of compound **6e** in $CDCl_3$ 32
Figure S37. 100 MHz ^{13}C NMR of compound **6e** in $CDCl_3$ 33
Figure S38. 100 MHz ^{1}H-^{13}C HSQC NMR of compound **6e** in $CDCl_3$ 33
Figure S39. 160 MHz ^{11}B NMR of compound **6e** in $CDCl_3$ 34
Figure S40. 500 MHz ^{1}H–^{15}N HMBC NMR of compound **6e** in $CDCl_3$ 34
2.9 Oxazaborine 6f 35
Figure S41. 500 MHz ^{1}H NMR of compound **6f** in $CDCl_3$ (Signal with asterix is –CH_3 from toluene.) 35
Figure S42. 125 MHz ^{13}C NMR of compound **6f** in $CDCl_3$ 36
Figure S43. 160 MHz ^{11}B NMR of compound **6f** in $CDCl_3$ 36
Figure S44. 50 MHz ^{15}N NMR of compound **6f** in $CDCl_3$ 37
Figure S45. 500 MHz ^{1}H–^{15}N HMBC NMR of compound **6f** in $CDCl_3$ 37
2.10 Oxazaborine 6g 38

Figure S46. 500 MHz ^{1}H NMR of compound **6g** in $CDCl_3$ 38
Figure S47. 125 MHz ^{13}C NMR of compound **6g** in $CDCl_3$ 39
Figure S48. 160 MHz ^{11}B NMR of compound **6g** in $CDCl_3$ 39
Figure S49. 400 MHz ^{1}H–^{15}N HMBC NMR of compound **6g** in $CDCl_3$ 40
2.11 Oxazaborine 6h 41
Figure S50. 500 MHz ^{1}H NMR of compound **6h** in $CDCl_3$ 41
Figure S51. 125 MHz ^{13}C NMR of compound **6h** in $CDCl_3$ (*Cyclohexane) 42
Figure S52. 160 MHz ^{11}B NMR of compound **6h** in $CDCl_3$ 42
Figure S53. 50 MHz ^{15}N NMR of compound **6h** in $CDCl_3$ 43
Figure S54. 500 MHz ^{1}H–^{15}N HMBC NMR of compound **6h** in $CDCl_3$ 43
2.12 Diazaborinone 7a 44
Figure S55. 500 MHz ^{1}H NMR of compound **7a** in $CDCl_3$ 44
Figure S56. 125 MHz ^{13}C NMR of compound **7a** in $CDCl_3$ 45
Figure S57. 160 MHz ^{11}B NMR of compound **7a** in $CDCl_3$ 45
Figure S58. 500 MHz ^{1}H–^{15}N HMBC NMR of compound **7a** in $CDCl_3$ 46
2.13 Diazaborinone 7b 47
Figure S59. 500 MHz ^{1}H NMR of compound **7b** in $CDCl_3$ 47
Figure S60. 125 MHz ^{13}C NMR of compound **7b** in $CDCl_3$ 48
Figure S61. 160 MHz ^{11}B NMR of compound **7b** in $CDCl_3$ 48
Figure S62. 50 MHz ^{15}N NMR of compound **7b** in $CDCl_3$ 49
Figure S63. 500 MHz ^{1}H–^{15}N HMBC NMR of compound **7b** in $CDCl_3$ 49
2.14 Diazaborinone 7c 50
Figure S64. 400 MHz ^{1}H NMR of compound **7c** in $CDCl_3$ 50
Figure S65. 125 MHz ^{13}C NMR of compound **7c** in $CDCl_3$ 51
Figure S66. 160 MHz ^{11}B NMR of compound **7c** in $CDCl_3$ 51
Figure S67. 500 MHz ^{1}H–^{15}N HMBC NMR of compound **7c** in $CDCl_3$ 52
2.15 Diazaborinone 7d 53
Figure S68. 500 MHz ^{1}H NMR of compound **7d** in $CDCl_3$ 53
Figure S69. 125 MHz ^{13}C NMR of compound **7d** in $CDCl_3$ 54
Figure S70. 160 MHz ^{11}B NMR of compound **7d** in $CDCl_3$ 54
Figure S71. 50 MHz ^{15}N NMR of compound **7d** in $CDCl_3$ 55
Figure S72. 500 MHz ^{1}H–^{15}N HMBC NMR of compound **7d** in $CDCl_3$ 55
2.16 Diazaborinone 7e 56
Figure S73. 500 MHz ^{1}H NMR of compound **7e** in $CDCl_3$ 56
Figure S74. 125 MHz ^{13}C NMR of compound **7e** in $CDCl_3$ 57
Figure S75. 160 MHz ^{11}B NMR of compound **7e** in $CDCl_3$ 57

Figure S76. 400 MHz ^1H–^{15}N HMBC NMR of compound **7e** in $CDCl_3$ (*CH_2 from ethanol) 58
2.17 Diazaborinone 7f 59
Figure S77. 500 MHz ^1H NMR of compound **7f** in $CDCl_3$ 59
Figure S78. 125 MHz ^{13}C NMR of compound **7f** in $CDCl_3$ 60
Figure S79. 160 MHz ^{11}B NMR of compound **7f** in $CDCl_3$ 60
Figure S80. 400 MHz ^1H–^{15}N HMBC NMR of compound **7f** in $CDCl_3$ 61
2.18 Diazaborinone 7g 62
Figure S81. 500 MHz ^1H NMR of compound **7g** in $CDCl_3$ 62
Figure S82. 125 MHz ^{13}C NMR of compound **7g** in $CDCl_3$ 63
Figure S83. 160 MHz ^{11}B NMR of compound **7g** in $CDCl_3$ 63
Figure S84. 500 MHz ^1H–^{15}N HMBC NMR of compound **7g** in $CDCl_3$ 64
2.19 Diazaborinone 7h 65
Figure S85. 500 MHz ^1H NMR of compound **7h** in $CDCl_3$ 65
Figure S86. 125 MHz ^{13}C NMR of compound **7h** in $CDCl_3$ 66
Figure S87. 160 MHz ^{11}B NMR of compound **7h** in $CDCl_3$ 66
Figure S88. 500 MHz ^1H–^{15}N HMBC NMR of compound **7h** in $CDCl_3$ 67
2.20 Triazaborine 8e 68
Figure S89. 500 MHz ^1H NMR of compound **8e** in $CDCl_3$ 68
Figure S90. 125 MHz ^{13}C NMR of compound **8e** in $CDCl_3$ 69
Figure S91. 160 MHz ^{11}B NMR of compound **8e** in $CDCl_3$ 69
Figure S92. 50 MHz ^{15}N NMR of compound **8e** in $CDCl_3$ 70
Figure S93. 500 MHz ^1H–^{15}N HMBC NMR of compound **8e** in $CDCl_3$ (* CH_2 from ethanol) 70
2.21 Triazaborine 8g 71
Figure S94. 500 MHz ^1H NMR of compound **8g** in $CDCl_3$ 71
Figure S95. 100 MHz ^{13}C NMR of compound **8g** in $CDCl_3$ 72
Figure S96. 128 MHz ^{11}B NMR of compound **8g** in $CDCl_3$ 72
2.22 Triazaborine 8h 73
Figure S97. 500 MHz ^1H NMR of compound **8h** in $CDCl_3$ 73
Figure S98. 125 MHz ^{13}C NMR of compound **8h** in $CDCl_3$ 74
Figure S99. 160 MHz ^{11}B NMR of compound **8h** in $CDCl_3$ 74
Figure S100. 50 MHz ^{15}N NMR of compound **8h** in $CDCl_3$ 75
Figure S101. 500 MHz ^1H–^{15}N HMBC NMR of compound 8h in $CDCl_3$ (* CH_2 from ethanol) 75
2.23 Oxazaborine–Triazaborine 9g 76
Figure S102. 400 MHz ^1H NMR of compound **9g** in $CDCl_3$ 76
Figure S103. 125 MHz ^{13}C NMR of compound **9g** in $CDCl_3$ 77
Figure S104. 160 MHz ^{11}B NMR of compound **9g** in $CDCl_3$ 77

Figure S105. 50 MHz ^{15}N NMR of compound **9g** in $CDCl_3$ 78
Figure S106. 500 MHz ^{1}H–^{15}N HMBC NMR of compound **9g** in $CDCl_3$ 78
2.24 Oxazaborine–Triazaborine 9h 79
Figure S107. 500 MHz ^{1}H NMR of compound **9h** in $CDCl_3$ 79
Figure S108. 125 MHz ^{13}C NMR of compound **9h** in $CDCl_3$ 80
Figure S109. 160 MHz ^{11}B NMR of compound **9h** in $CDCl_3$ 80
Figure S110. 50 MHz ^{15}N NMR of compound **9h** in $CDCl_3$ 81
Figure S111. 500 MHz ^{1}H–^{15}N HMBC NMR of compound **9h** in $CDCl_3$ 81
3. UV–Vis spectra 82
Figure S112. Normalized UV–VIS spectrum of oxazaborine **6g.** 82
Figure S113. UV–Vis spectrum in CH_2Cl_2. (**a**) triazaborinone **5a** and (**b**) triazaborinone **5b**. 82
Figure S114. UV–Vis spectrum in CH_2Cl_2. (**a**) triazaborinone **5c** and (**b**) triazaborinone **5d**. 82
Figure S115. UV–Vis spectrum in CH_2Cl_2. (**a**) triazaborinone **5g** and (**b**) triazaborinone **5h**. 83
Figure S116. UV–Vis spectrum in CH_2Cl_2 oxazaborine **6a**. 83
Figure S117. UV–Vis spectrum in CH_2Cl_2. (**a**) oxazaborine **6e** and (**b**) oxazaborine **6f**. 83
Figure S118. UV–Vis spectrum in CH_2Cl_2. (**a**) oxazaborine **6g** and (**b**) oxazaborine **6h**. 84
Figure S119. UV–Vis spectrum in CH_2Cl_2. (**a**) diazaborine 7**a** and (**b**) diazaborinone **7b**. 84
Figure S120. UV–Vis spectrum in CH_2Cl_2. (**a**) diazaborine **7c** and (**b**) diazaborinone **7d**. 84
Figure S121. UV–Vis spectrum in CH_2Cl_2. (**a**) diazaborine **7e** and (**b**) diazaborinone **7f**. 85
Figure S122. UV–Vis spectrum in CH_2Cl_2. (**a**) diazaborine **7g** and (**b**) diazaborinone **7h**. 85
Figure S123. UV–Vis spectrum in CH_2Cl_2. (**a**) triazaborine **8e** and (**b**) triazaborie **8h**. 85
Figure S124. UV–Vis spectrum in CH_2Cl_2. (**a**) oxazaborine–triazaborine **9g** and (**b**) oxazaborine–triazaborie **9h**.. 86
Table S1. UV–Vis parameters of prepared heterocyclic compounds in CH_2Cl_2. 87
Table S2. UV–Vis parameters of triazaborine **5g**. λ_{max} (nm), **ε** ($l{\cdot}cm^{-1}mol^{-1}$) 88
Table S3. UV–Vis parameters of oxazaborine **6g**. λ_{max} (nm), **ε** ($l{\cdot}cm^{-1}mol^{-1}$). 88
Table S4. UV–Vis parameters of diazaborinone **7a**. λ_{max} (nm), **ε** ($l{\cdot}cm^{-1}mol^{-1}$). 88
4. Crystallographic data 89
Table S5. Crystal data. 89
Figure S125. ORTEP view of the crystal structure of **5c** in the direction of the crystallographic axis *a*. 90
Figure S126. ORTEP view of the crystal structure of **5c** in the direction of the crystallographic axis *c*. 91
Figure S127. ORTEP view of the crystal structure of **5c** in the direction of the reciprocal cell axis *b**. 92
Figure S128. Triazaborine **5c**. The planes on which the double bonds C=N, C=N, and C=O lie. 93
Figure S129. ORTEP view of the crystal packing of triazaborinone **5d** in the direction of the crystallographic axis *a*. 94
Figure S130. ORTEP view of the crystal packing of triazaborinone **5d** in the direction of the crystallographic axis *b*. 95

Figure S131. ORTEP view of the crystal structure of **5g** in the direction of the crystallographic axis *b*. 96

Figure S132. ORTEP diagram of the oxazaborine **6a** asymmetric unit. 97

Figure S133. ORTEP view of the π–π stacking chain of **6e** in the direction of the crystallographic axis *c*. 98

Figure S134. ORTEP view of the crystal structure of **6e** in the direction of the crystallographic axis *b*. 99

Figure S135. ORTEP view of the crystal packing of diazaborinone **7f** in the direction of the crystallographic axis *a*. 100

Figure S136. ORTEP view of the crystal packing of diazaborinone **7f** in the direction of the crystallographic axis *b*. 101

5. Fluorescence 102

Figure S137. Triazaborinone **5g**. (**a**) Absorption spectrum and (**b**) Emission spectrum. 102

Figure S138. Triazaborinone **5h**. (**a**) Absorption spectrum and (**b**) Emission spectrum. 102

Figure S139. Diazaborinone **7a**. (**a**) Absorption spectrum and (**b**) Emission spectrum. 102

1. Experimental

1. 1 General Information

All chemicals and dried dichloromethane except those mentioned below were purchased from commercial suppliers (Acros Organics, Sigma-Aldrich, or Fluorochem). Toluene and diethylether were dried over sodium.

NMR spectra were measured in $CDCl_3$ or in DMSO-d_6 using a Bruker AVANCE III 400 spectrometer operating at 400 MHz (^{1}H) and 100 MHz (^{13}C) or using a Bruker Ascend 500 spectrometer operating at 500 MHz (^{1}H), 125 MHz (^{13}C), 160 MHz (^{11}B), and 50 MHz (^{15}N). All of the pulse sequences were taken from the Bruker software library. The ^{13}C NMR spectra were measured in a standard way and by means of the APT pulse sequence. The data are reported as follows. chemical shift in ppm (δ), multiplicity (s = singlet, d = doublet, q = quartet, m = multiplet, br s = broad singlet, br q = broad quartet). The coupling constants *J* are reported in Hertz (Hz). TMS was used as an internal standard for ^{1}H NMR in $CDCl_3$ (δ 0.00). $CDCl_3$ was used as an internal standard for ^{13}C NMR (the middle signal, δ 77.16). $B(OMe)_3$ was used as an external standard for ^{11}B NMR (δ 18.1). Nitromethane was used as an external standard for ^{15}N NMR (δ 0.00). DMSO-d_6 was used as an internal standard for both ^{1}H NMR (the middle signal δ 2.55) and ^{13}C NMR (the middle signal δ 39.6).

Elemental analyses were performed on a Flash 2000 CHNS Elemental Analyzer.

Melting points were measured on a Kofler Boetius PHMK 80/2644 hot-stage microscope and were not corrected.

High-resolution mass spectra were recorded on a MALDI LTQ Orbitrap XL (Thermo Fisher Scientific) equipped with a nitrogen UV laser (337 nm, 60 Hz, 8–20 μJ) in the positive ion mode. For the CID experiment using the linear trap quadrupole (LTQ) helium was used as the collision gas and 2,5-dihydroxybenzoic acid (DHB) as the MALDI matrix.

UV−Vis spectra were recorded on a UV−Vis spectrophotometer Hewlett-Packard 8453. IR spectra were recorded on a Nicolet iS50 equipped with an ATR diamond crystal (neat solid samples). The wavenumber range 2500–1700 cm^{-1} is excluded due to diamond absorption.

Steady-state and time-resolved PL measurements were performed based on a multifunctional spectrometer with L-geometry of excitation-detection pathways (FluoTime 300, PicoQuant). A Xenon arc lamp and a PMT were used as the excitation sources and detectors for PL experiments. The PL-QY value can be obtained based on the spectrometer incorporated with an integrating sphere. Excitation wavelength. 360 nm. Range. 340–700 nm.

Crystal data for all compounds were collected at 295 K using a Nonius Kappa CCD diffractometer with graphite monochromated Mo-Kα radiation (λ = 0.71073 Å). The data sets were integrated with the Denzo SMN package and corrected for Lorentz, polarization, and absorption effects (SORTAV). Structures were solved by direct methods using the SIR97 system of programs and refined anisotropically by using full-matrix least-squares for all non-hydrogen atoms and hydrogen atoms included on their calculated positions, riding on their carrier atoms; except the N–H hydrogens forming intramolecular hydrogen bonds, which were refined isotropically. All calculations were performed by using SHELXL 2014/6, PARST and PLATON implemented in WINGX system of programs. Visualisation of structures was done with MERCURY 2020.2.0.

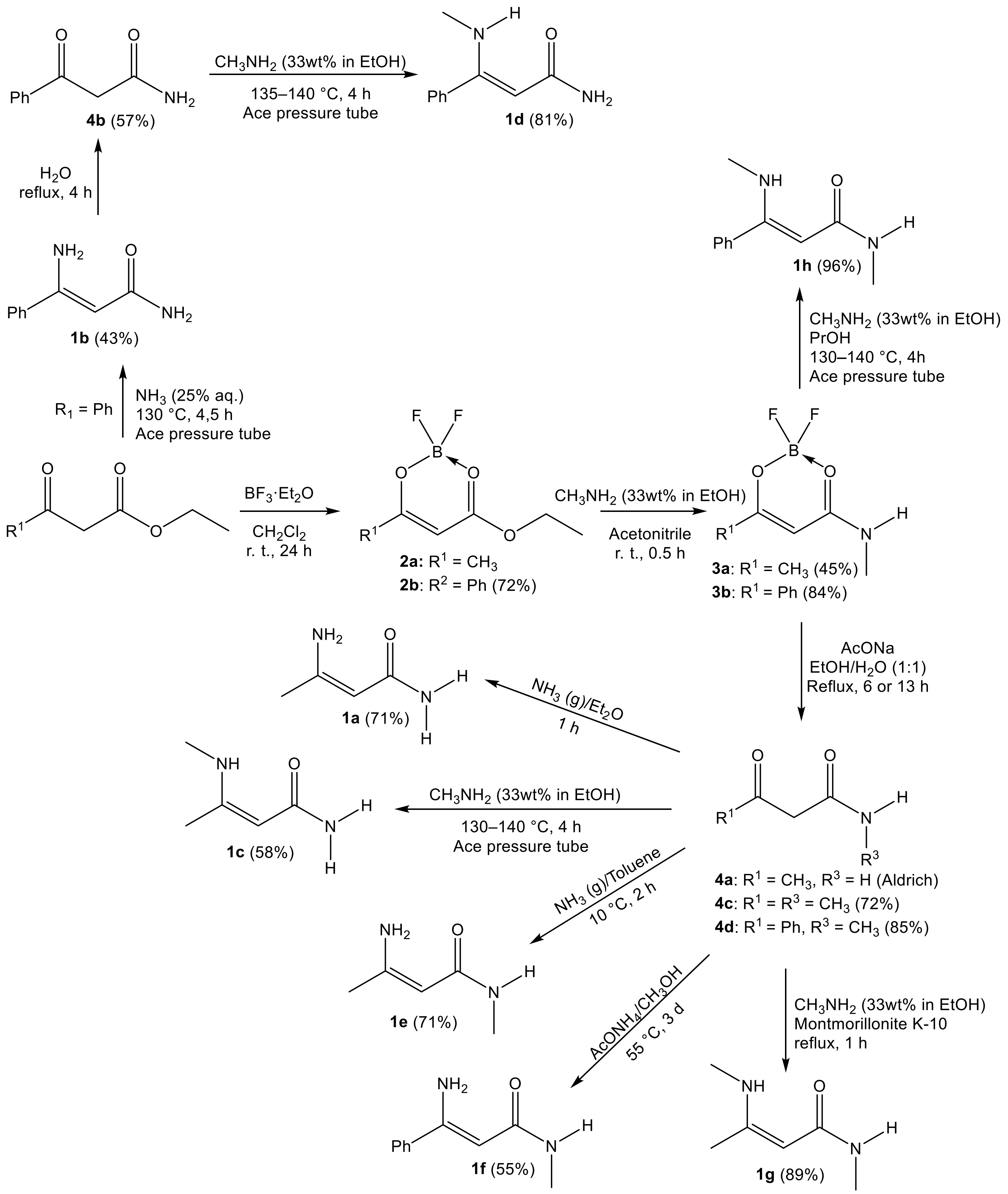

Scheme S1. Preparation of starting compounds.

2. NMR spectra

2.1 Triazaborinone 5a

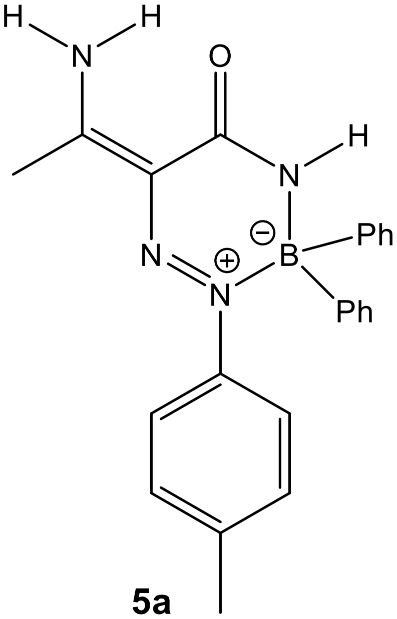

5a

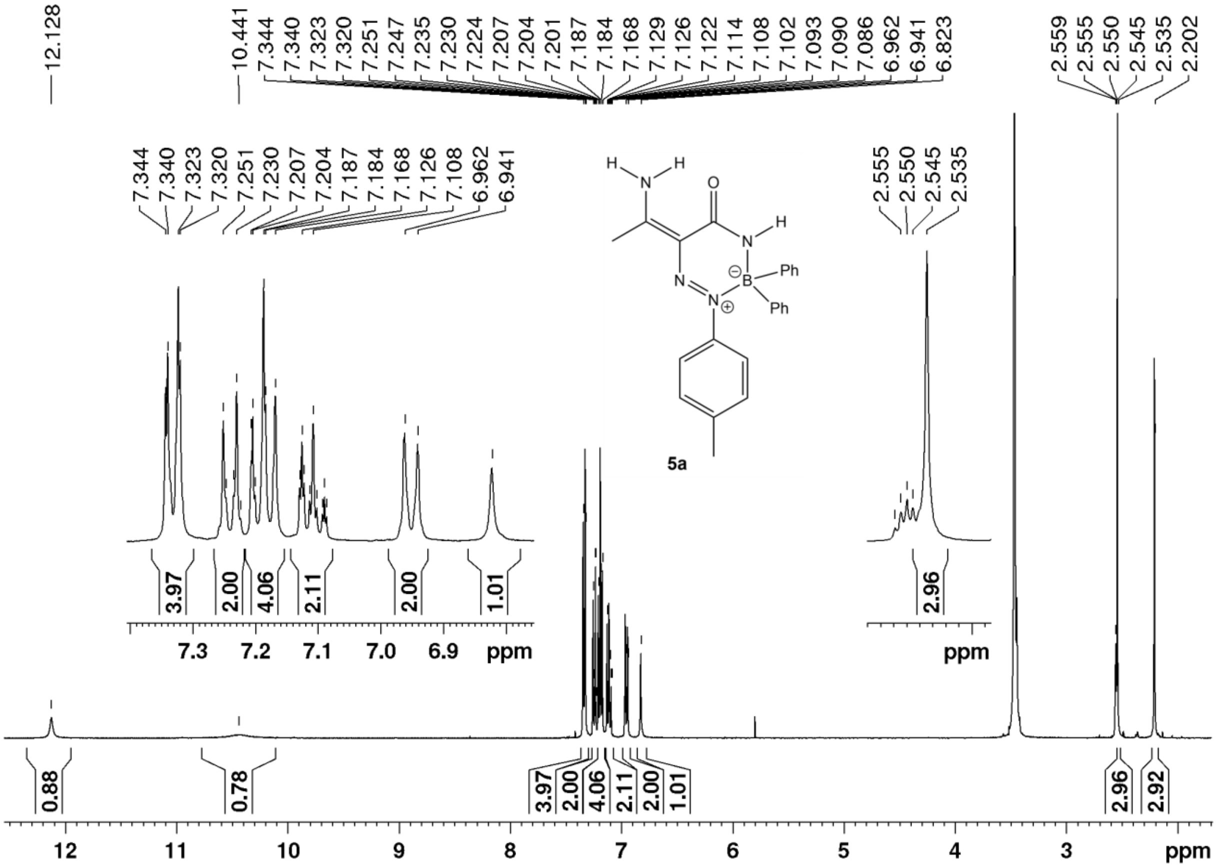

Figure S1. 400 MHz ^{1}H NMR of compound **5a** in DMSO-d_6

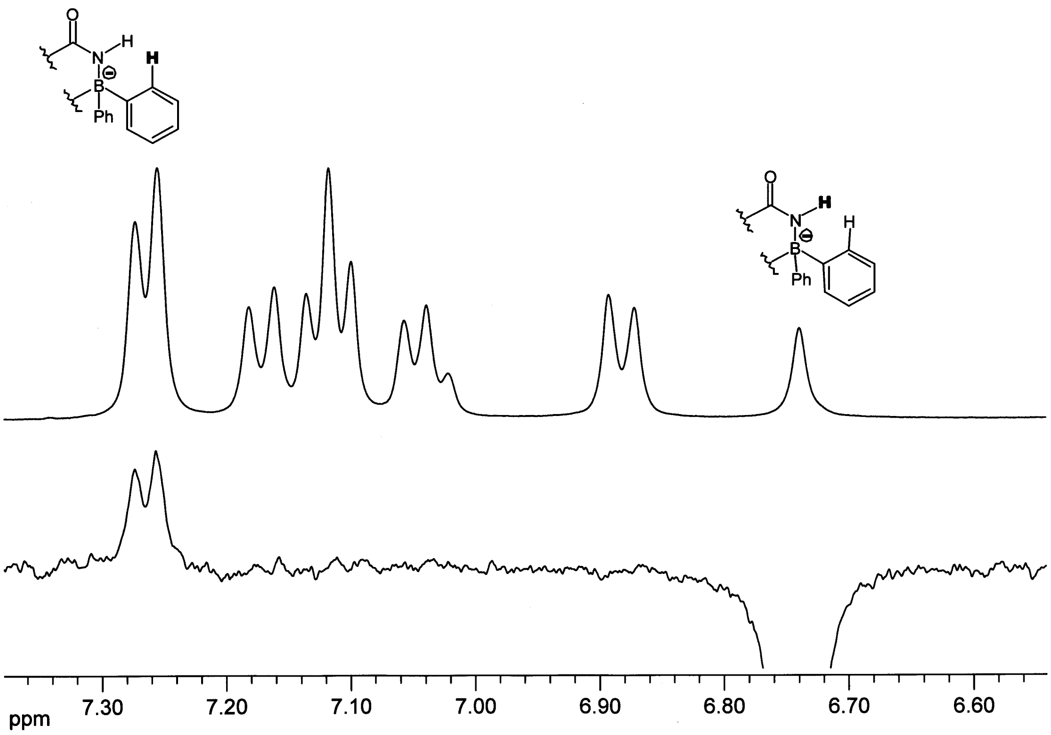

Figure S2. 400 MHz 1D-NOESY (mixing time d8 = 800 ms, relaxation delay d1 = 2s) of compound **5a** in DMSO-d_6

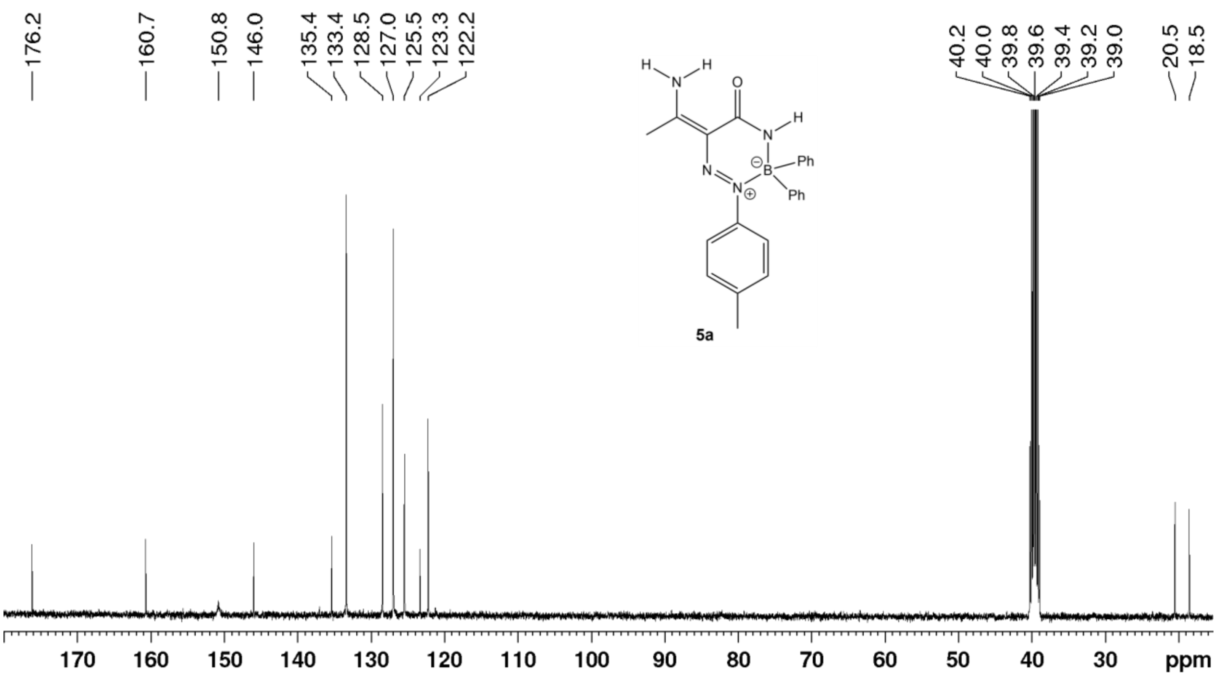

Figure S3. 100 MHz ^{13}C NMR of compound **5a** in DMSO-d_6

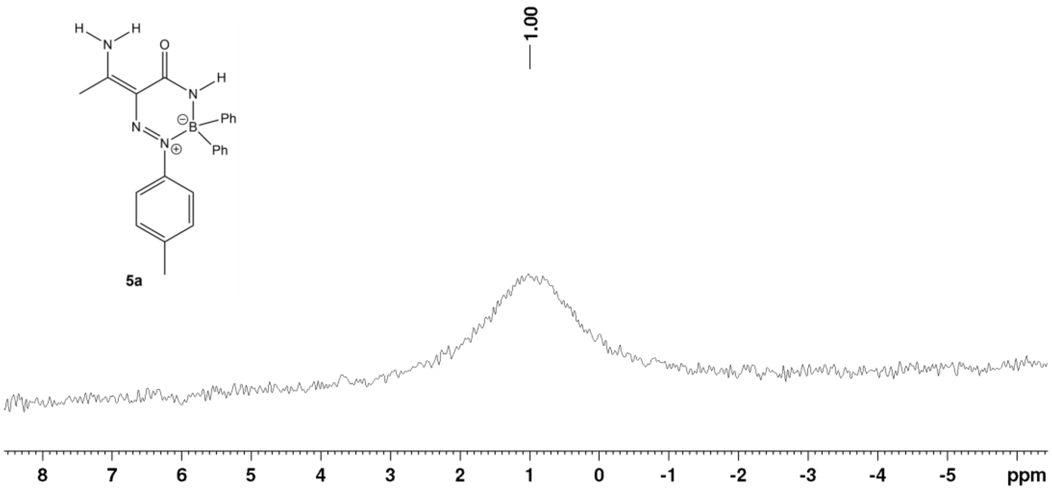

Figure S4. 160 MHz ^{11}B NMR of compound **5a** in $CDCl_3$

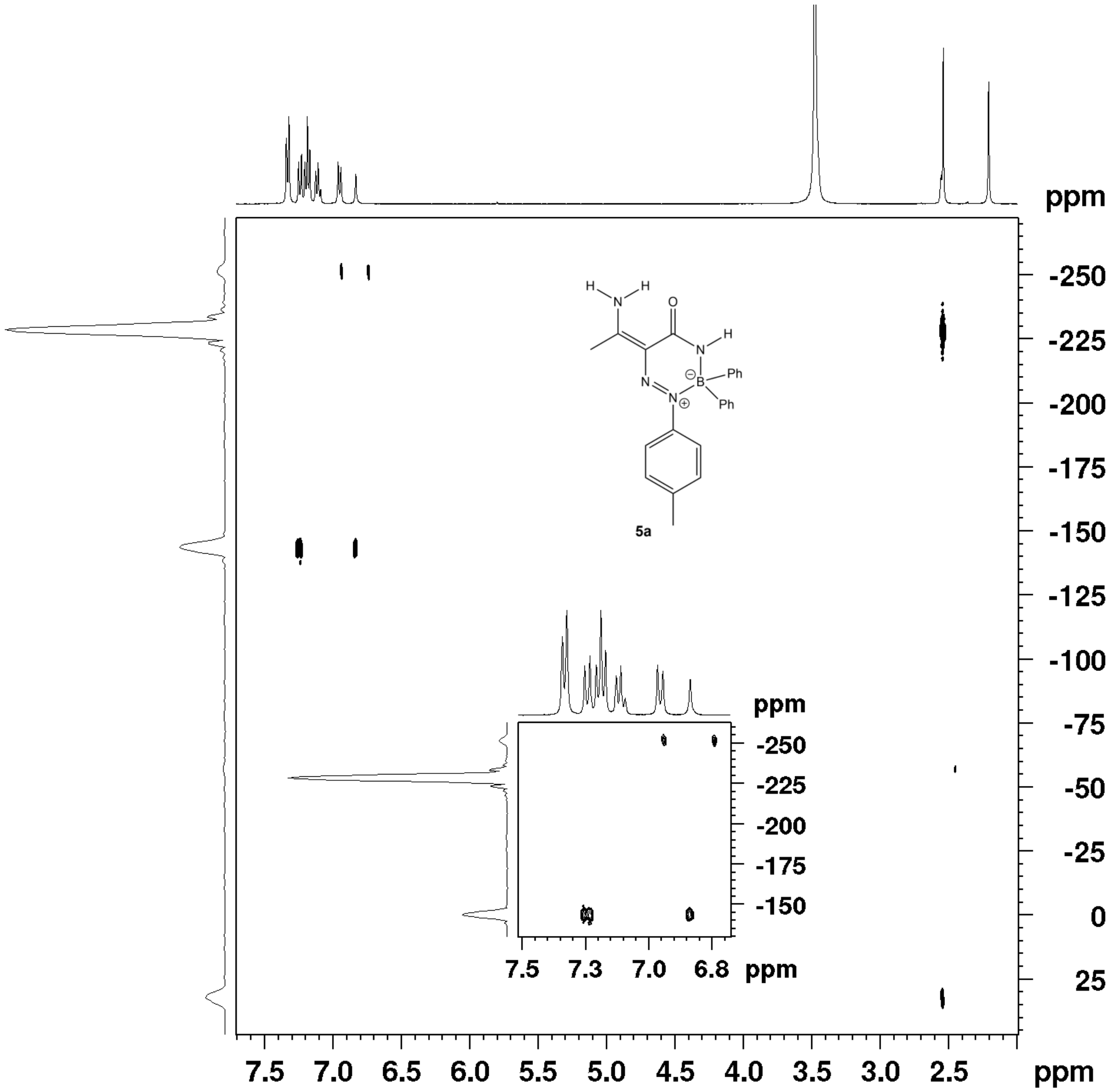

Figure S5. 500 MHz ^{1}H–^{15}N HMBC NMR of compound **5a** in DMSO-d_6

2.2 Triazaborinone 5b

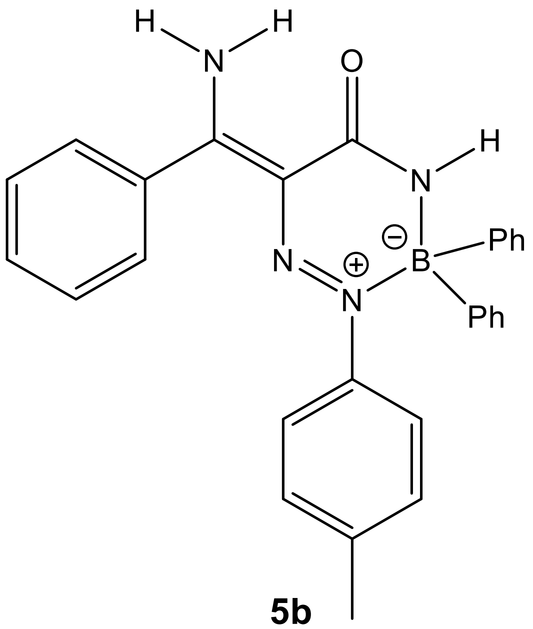

5b

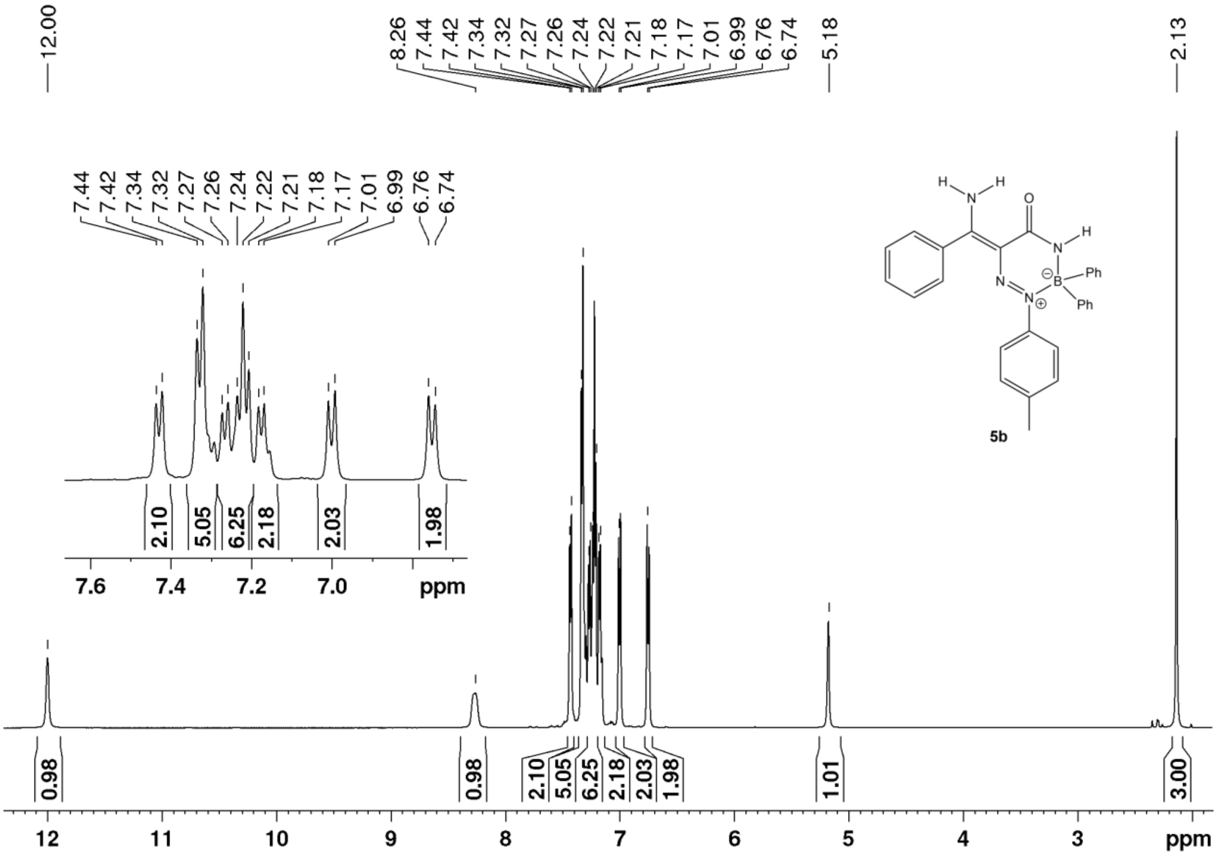

Figure S6. 500 MHz ^{1}H NMR of compound **5b** in $CDCl_3$

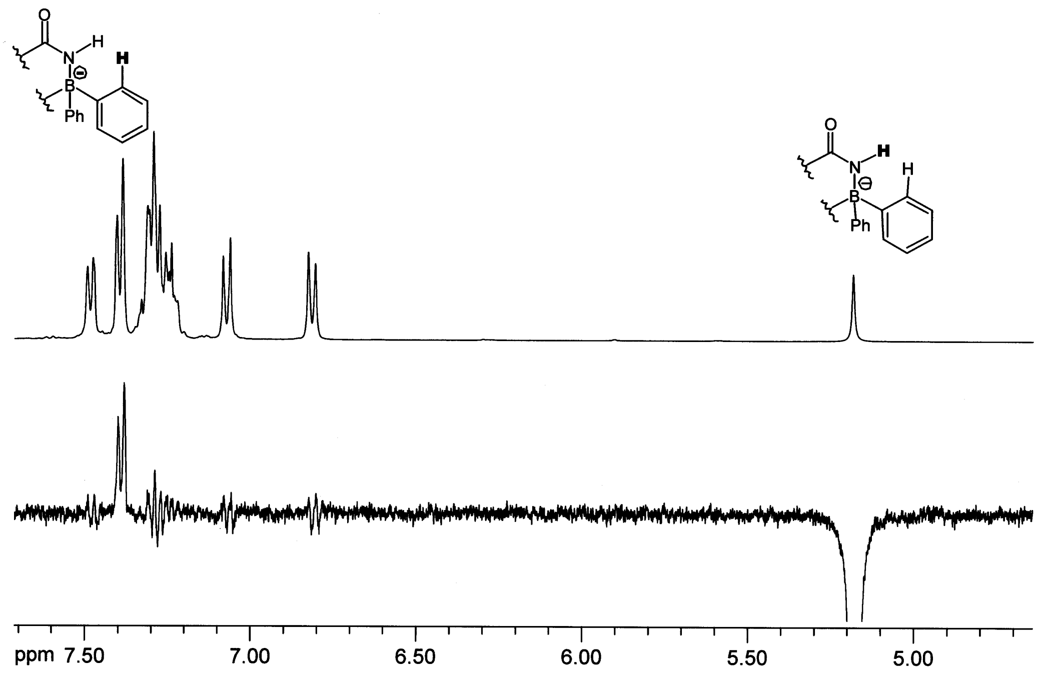

Figure S7. 400 MHz 1D-NOESY (mixing time d8 = 800 ms, relaxation delay d1 = 2s) of compound **5b** in $CDCl_3$

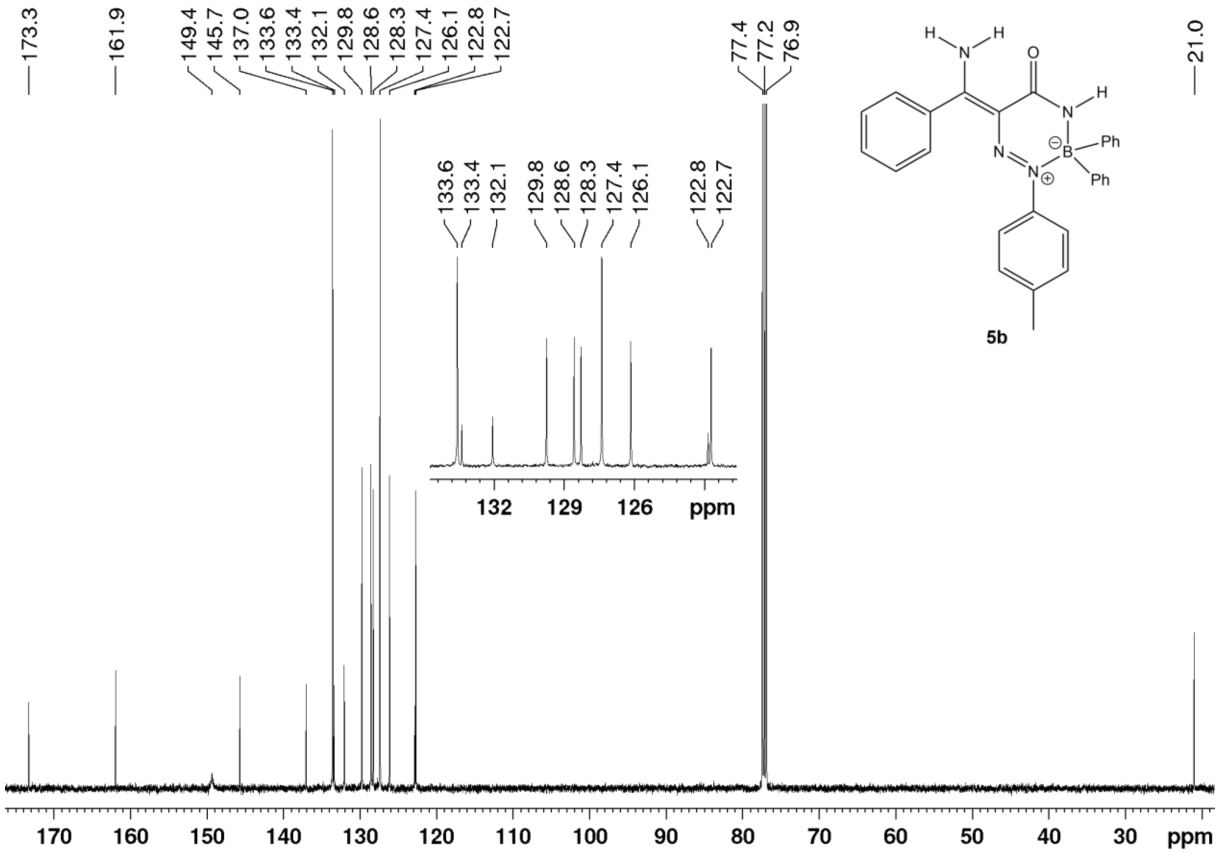

Figure S8. 125 MHz ^{13}C NMR of compound **5b** in $CDCl_3$

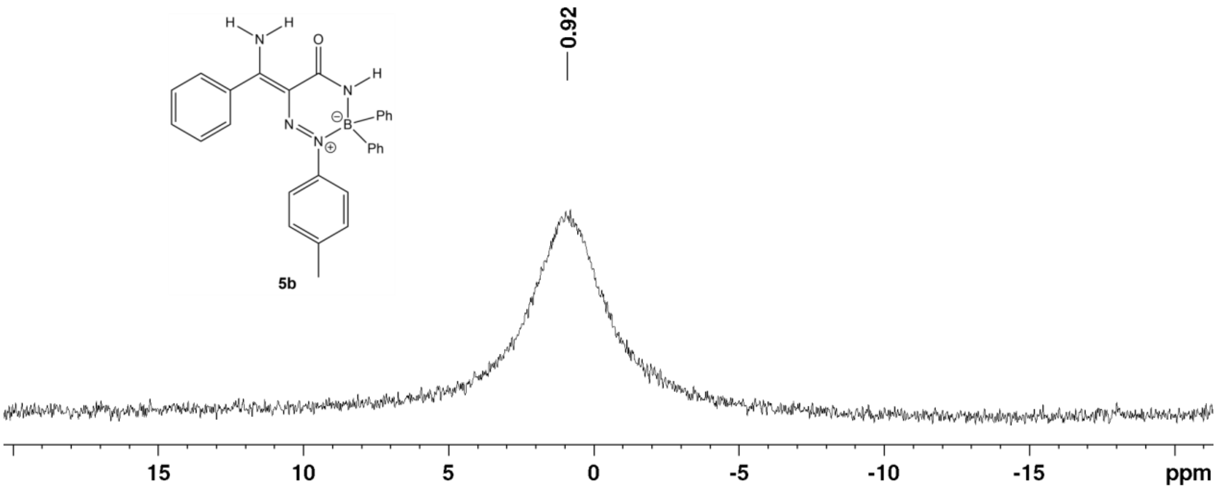

Figure S9. 128 MHz ^{11}B NMR of compound **5a** in $CDCl_3$

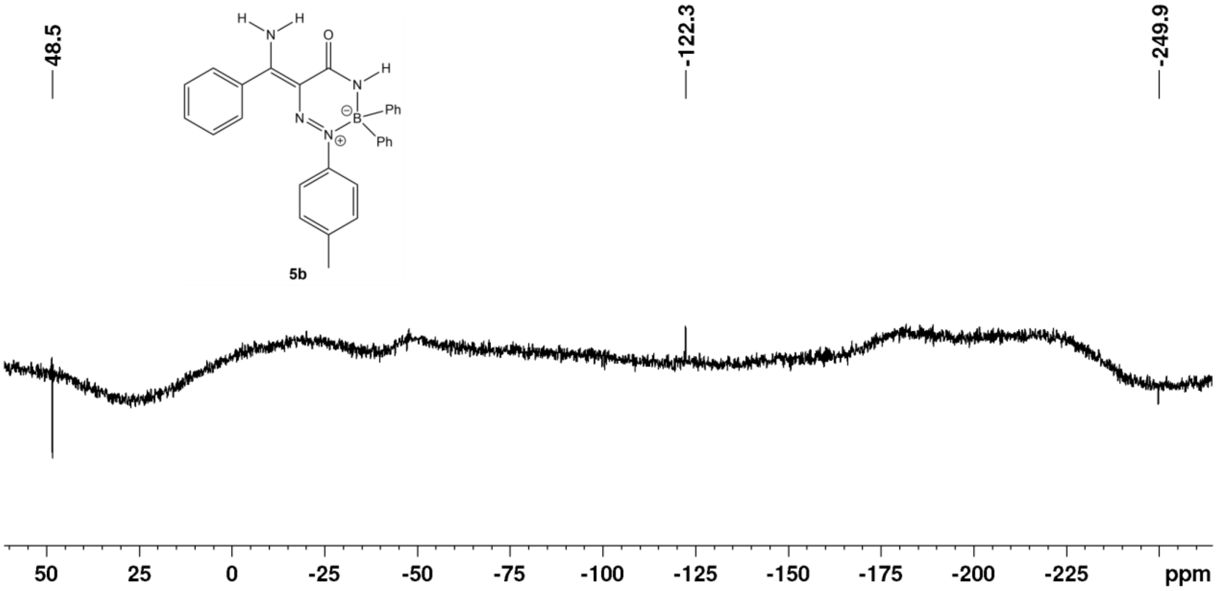

Figure S10. 50 MHz ^{15}N NMR of compound **5a** in DMSO-d_6

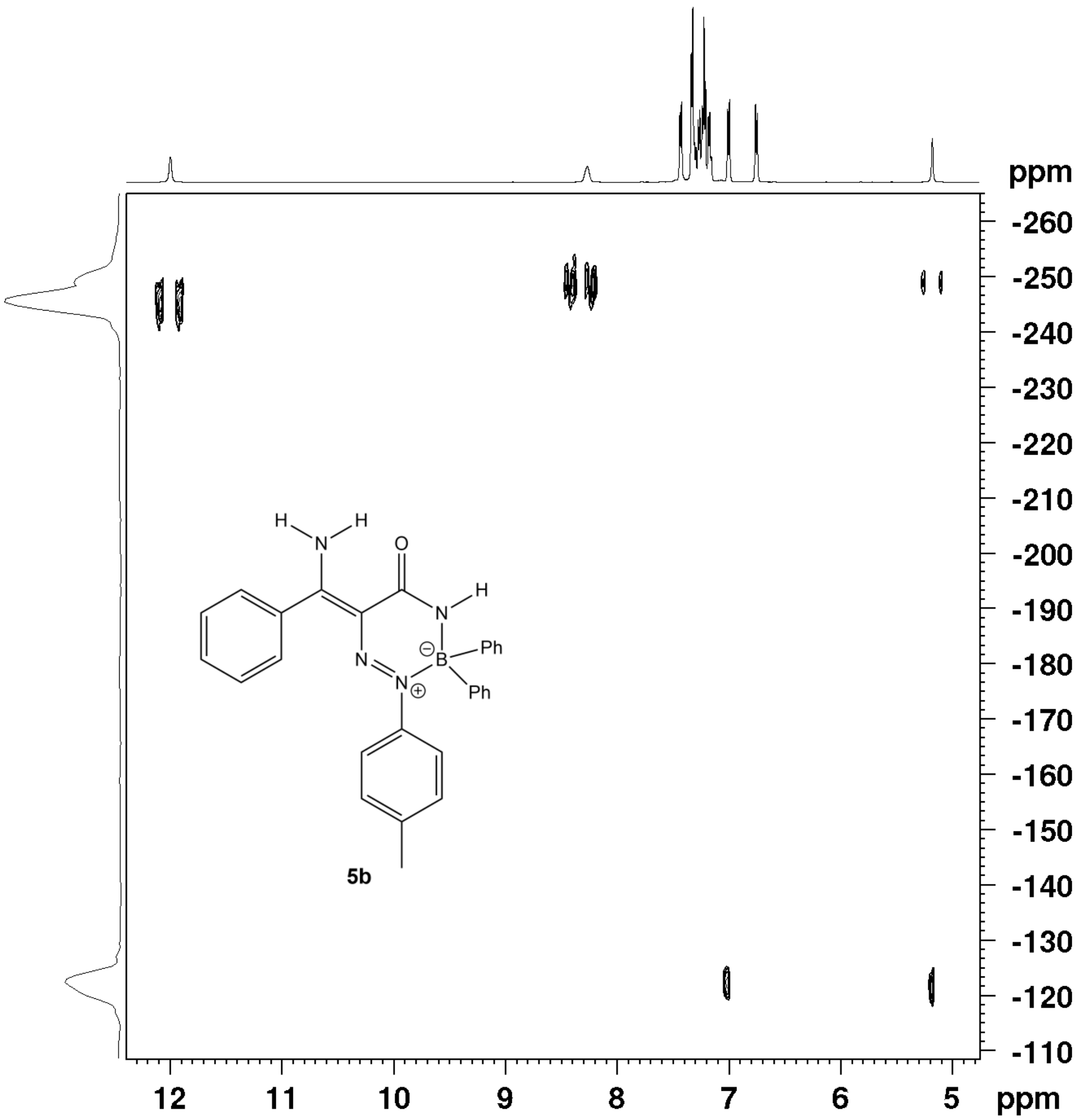

Figure S11. 500 MHz ^{1}H–^{15}N HMBC NMR of compound **5b** in $CDCl_3$

2.3 Triazaborinone 5c

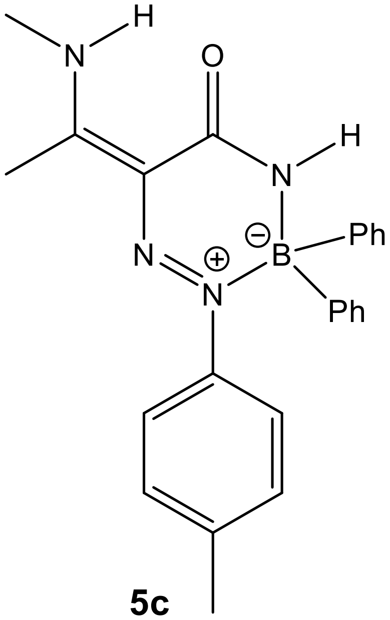

5c

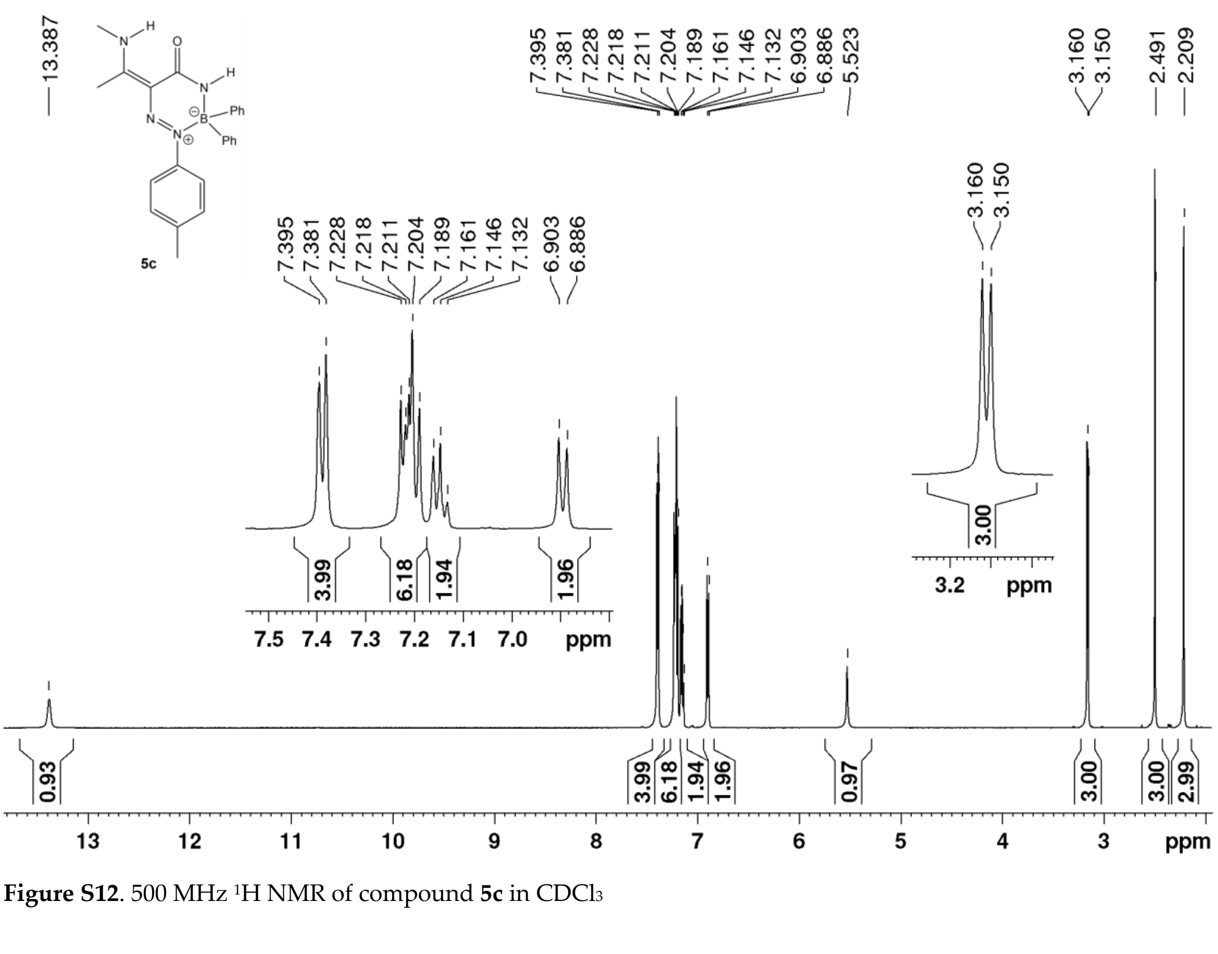

Figure S12. 500 MHz ^{1}H NMR of compound **5c** in $CDCl_3$

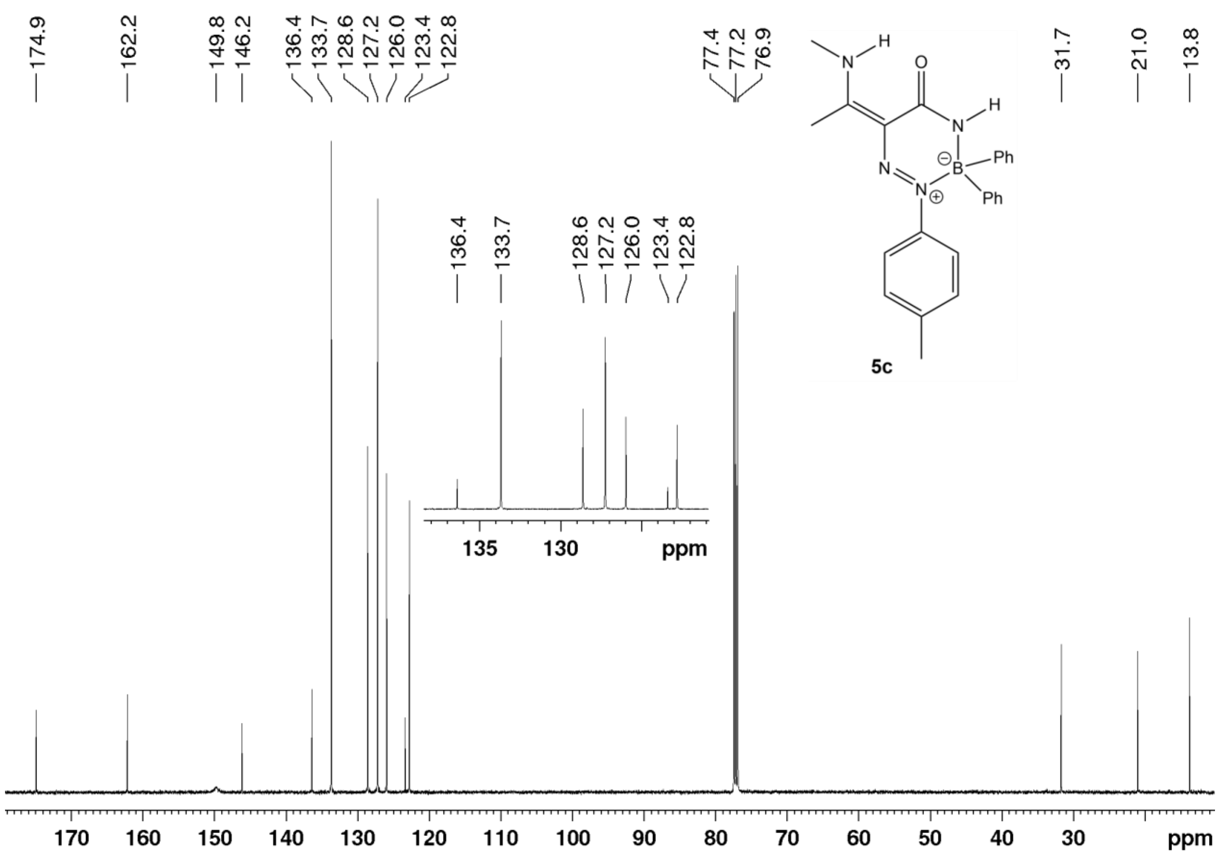

Figure S13. 125 MHz ^{13}C NMR of compound **5c** in $CDCl_3$

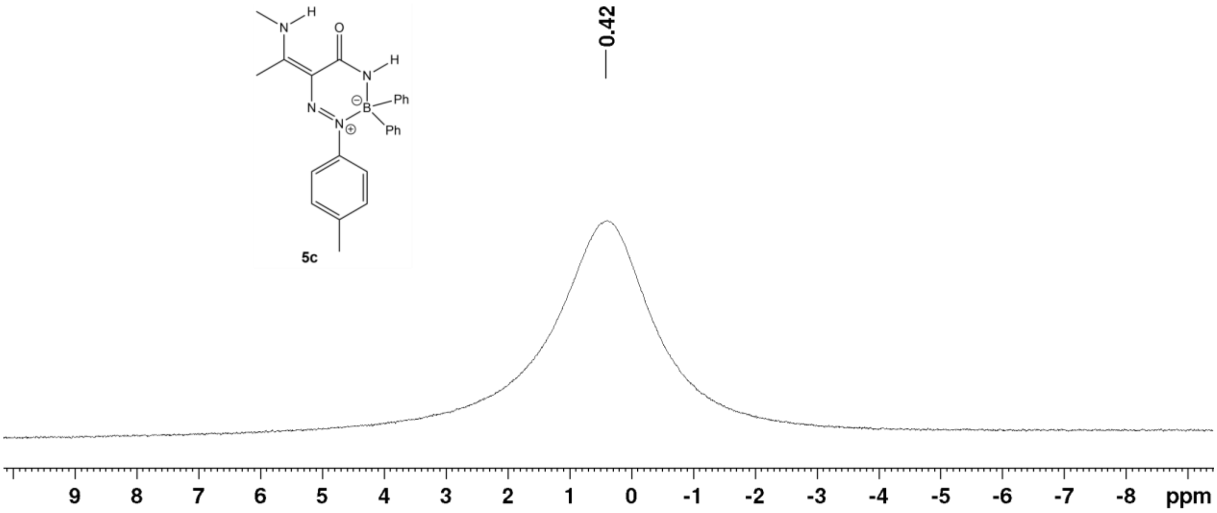

Figure S14. 160 MHz ^{11}B NMR of compound **5c** in $CDCl_3$

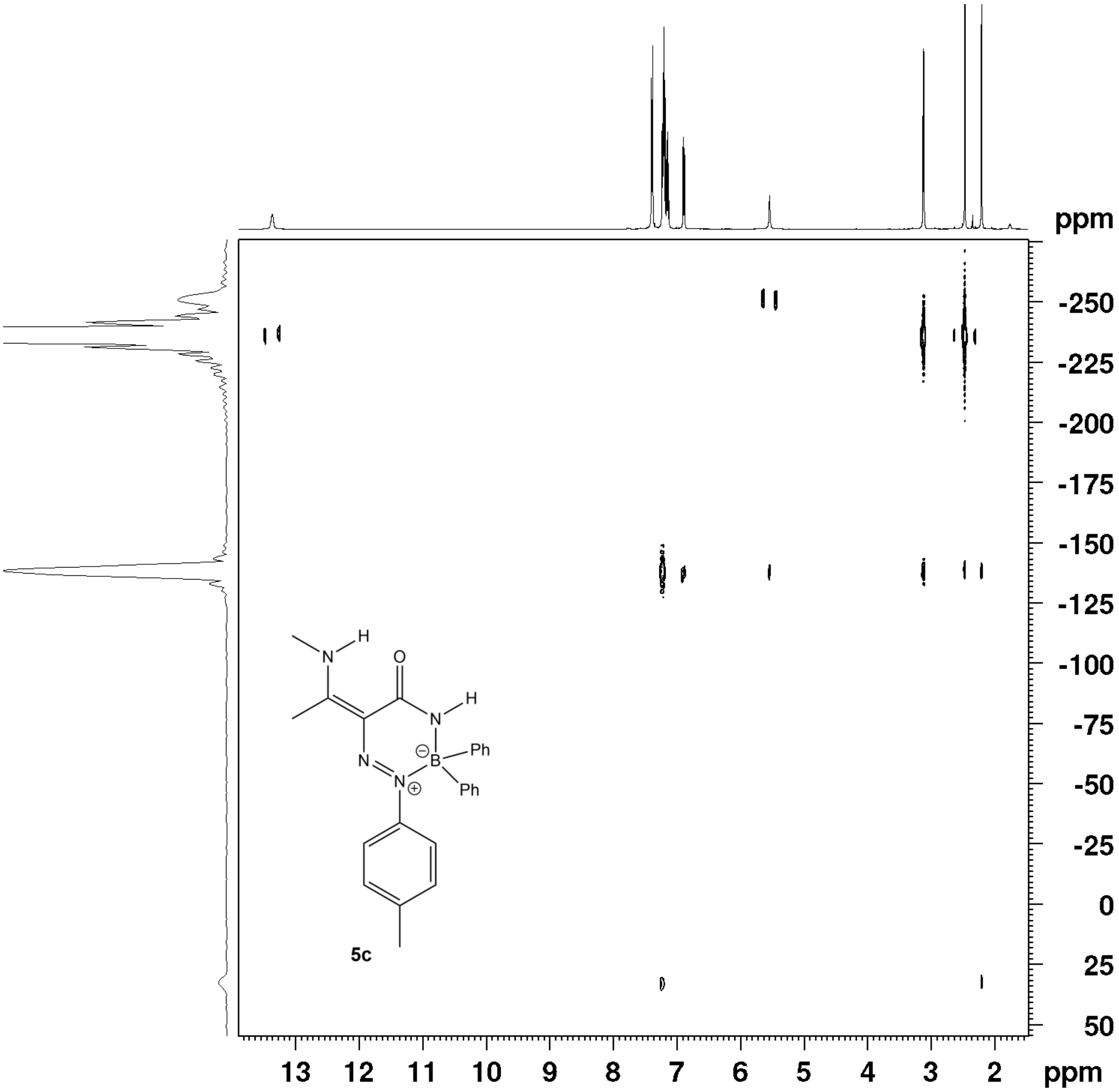

Figure S15. 500 MHz ¹H–¹⁵N HMBC NMR of compound **5c** in CDCl₃

2.4 Triazaborinone 5d

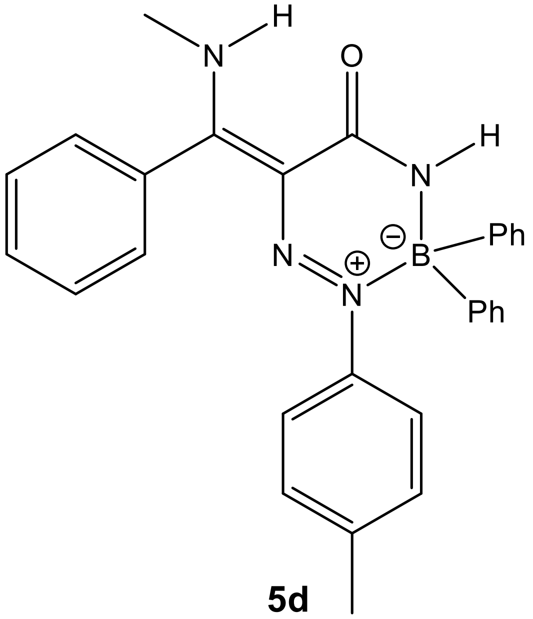

5d

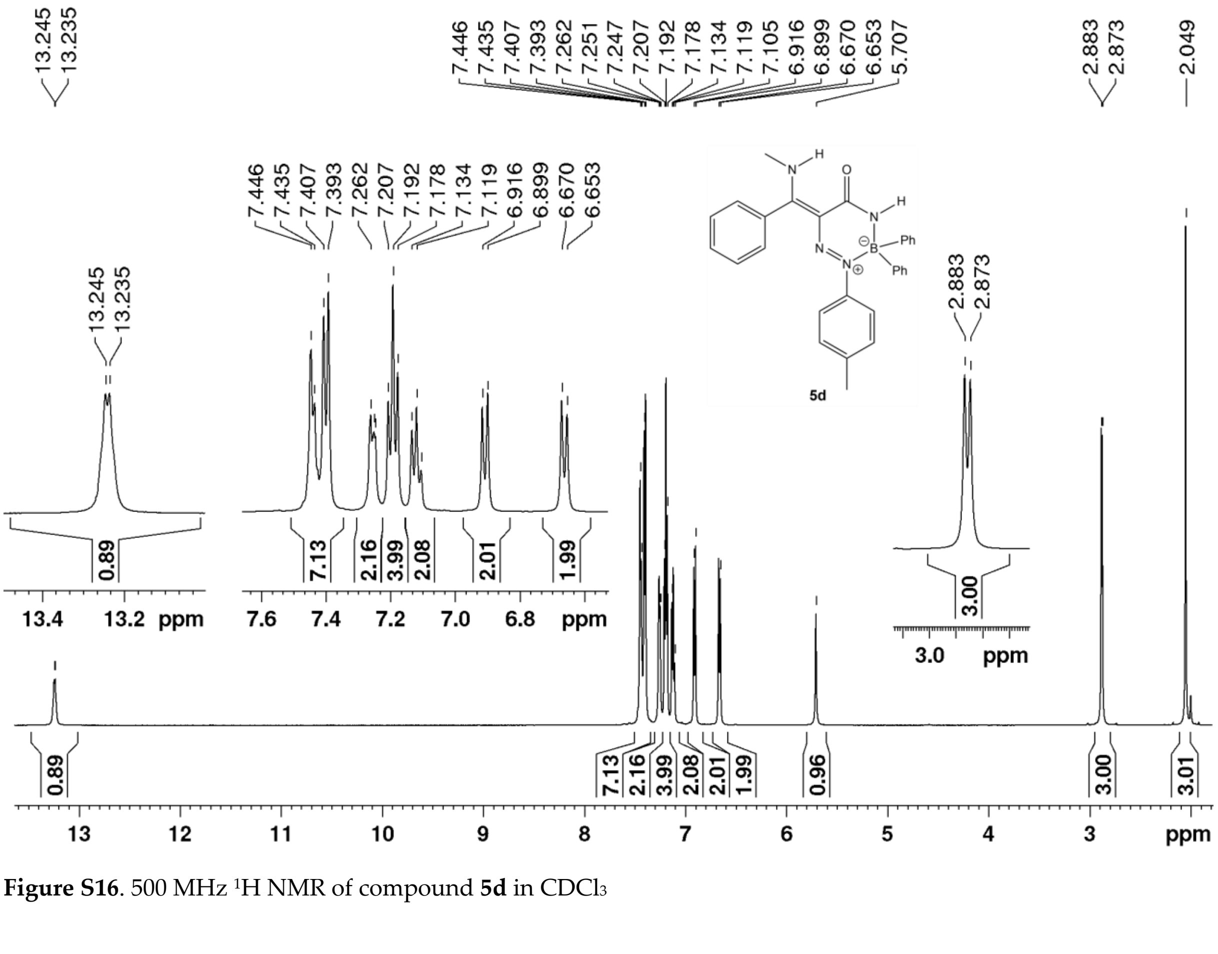

Figure S16. 500 MHz ^1H NMR of compound **5d** in $CDCl_3$

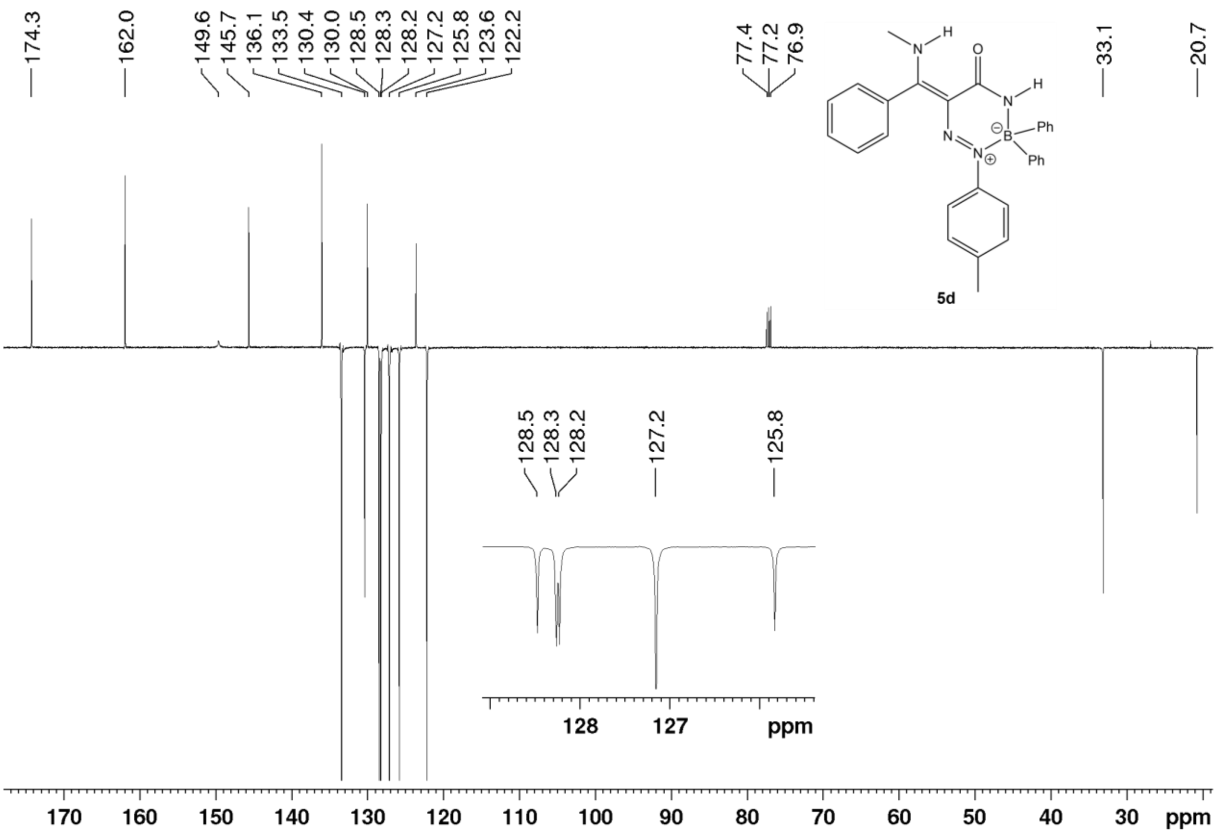

Figure S17. 125 MHz ^{13}C APT NMR of compound **5d** in $CDCl_3$

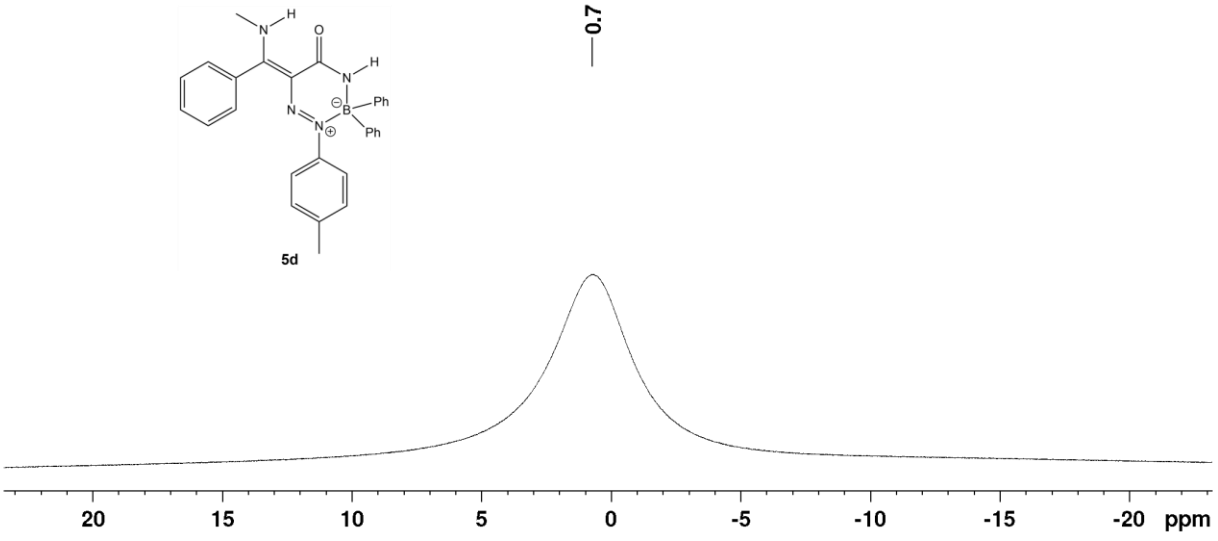

Figure S18. 160 MHz ^{11}B NMR of compound **5c** in $CDCl_3$

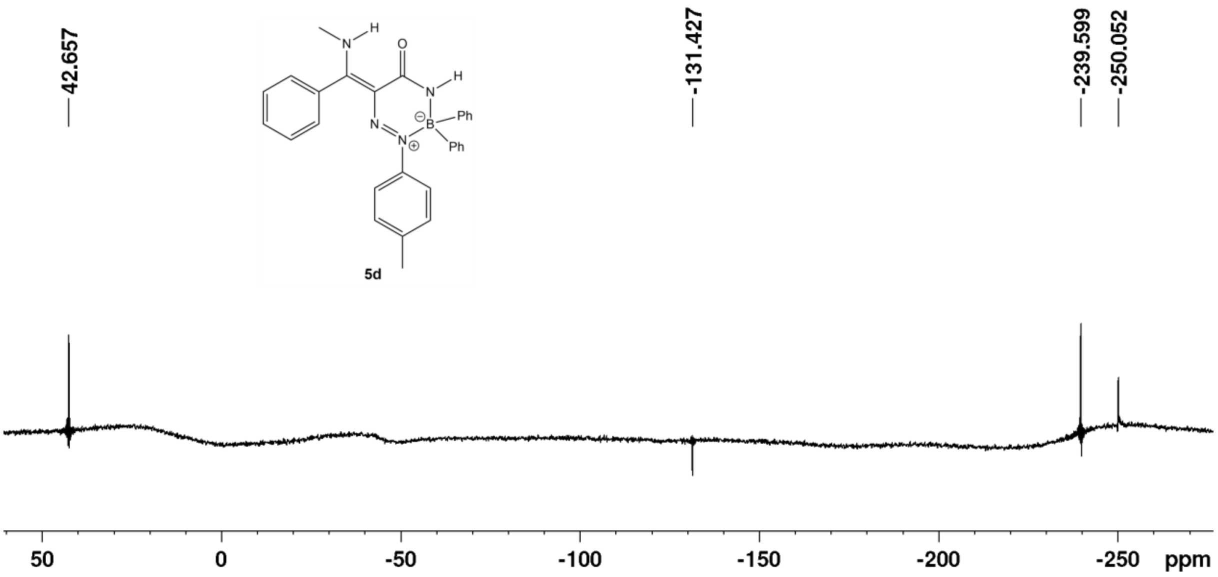

Figure S19. 50 MHz ^{15}N NMR of compound **5d** in $CDCl_3$

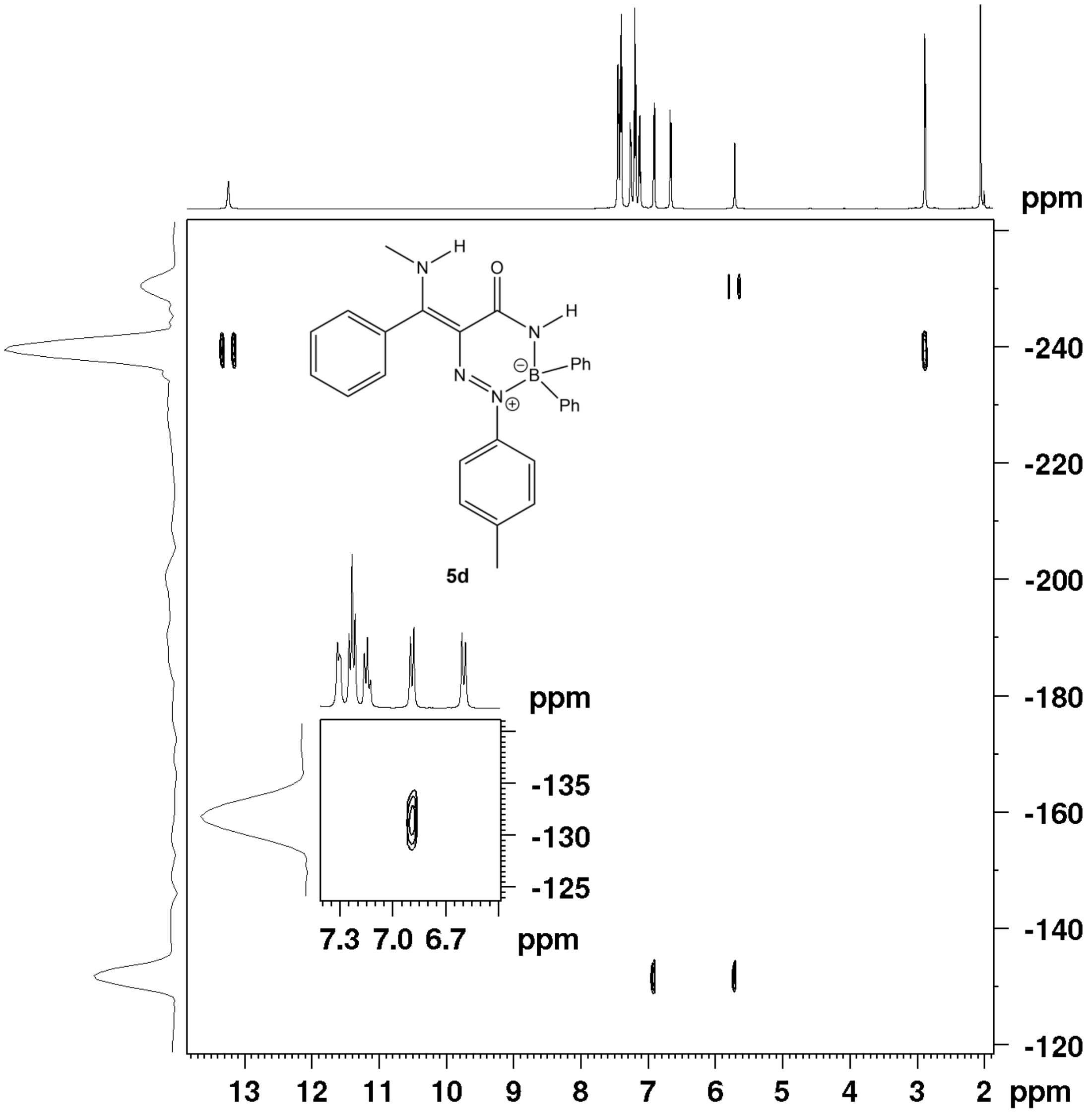

Figure S20. 500 MHz ^{1}H–^{15}N HMBC NMR of compound **5d** in $CDCl_3$

2.5 Triazaborinone 5g

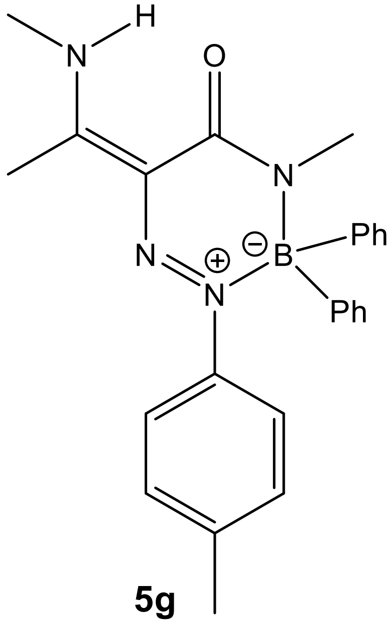

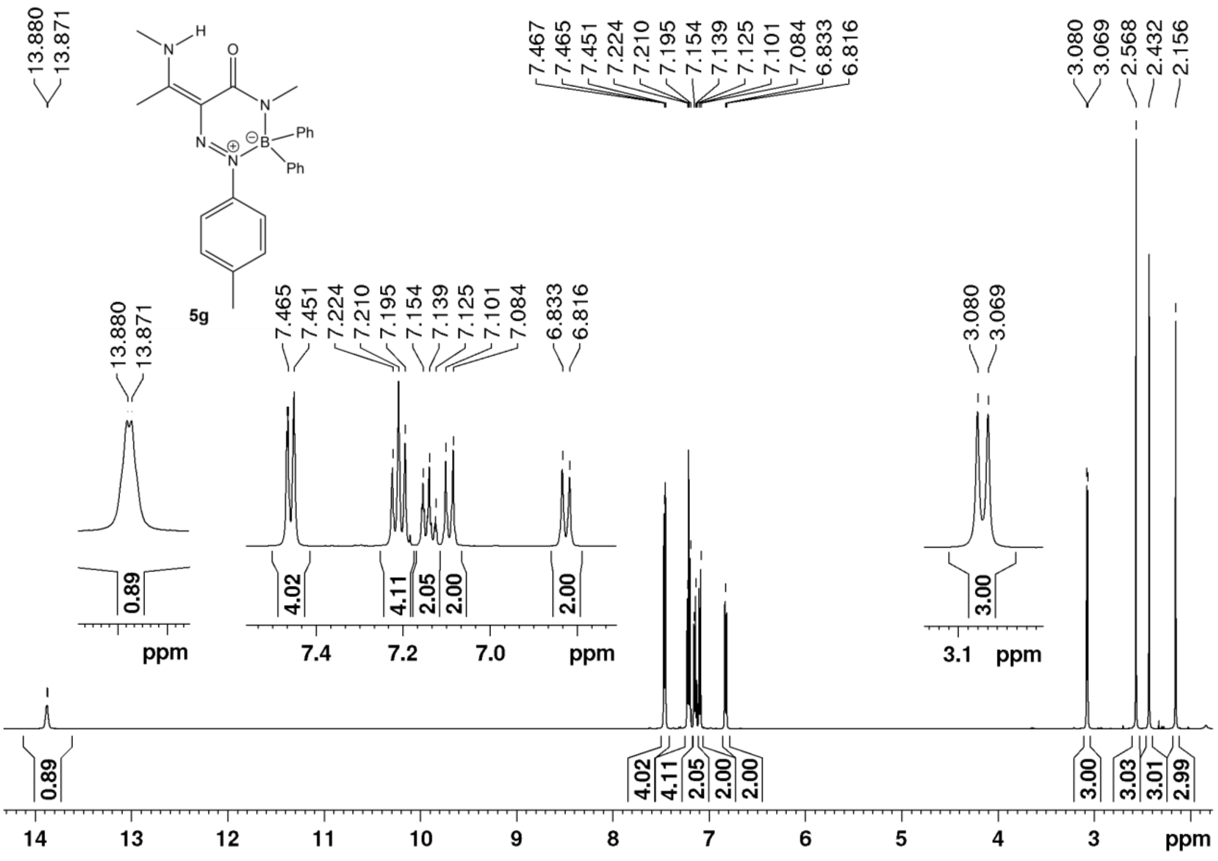

Figure S21. 500 MHz ^{1}H NMR of compound **5g** in $CDCl_3$

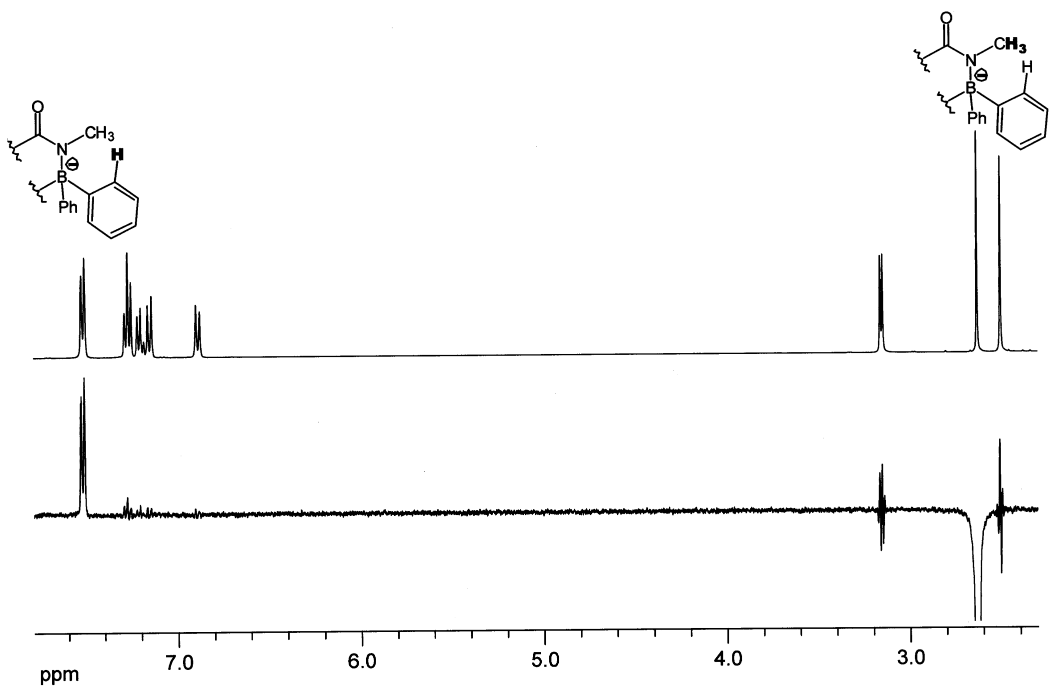

Figure S22. 400 MHz 1D-NOESY (mixing time d8 = 800 ms, relaxation delay d1 = 2s) of compound **5g** in $CDCl_3$

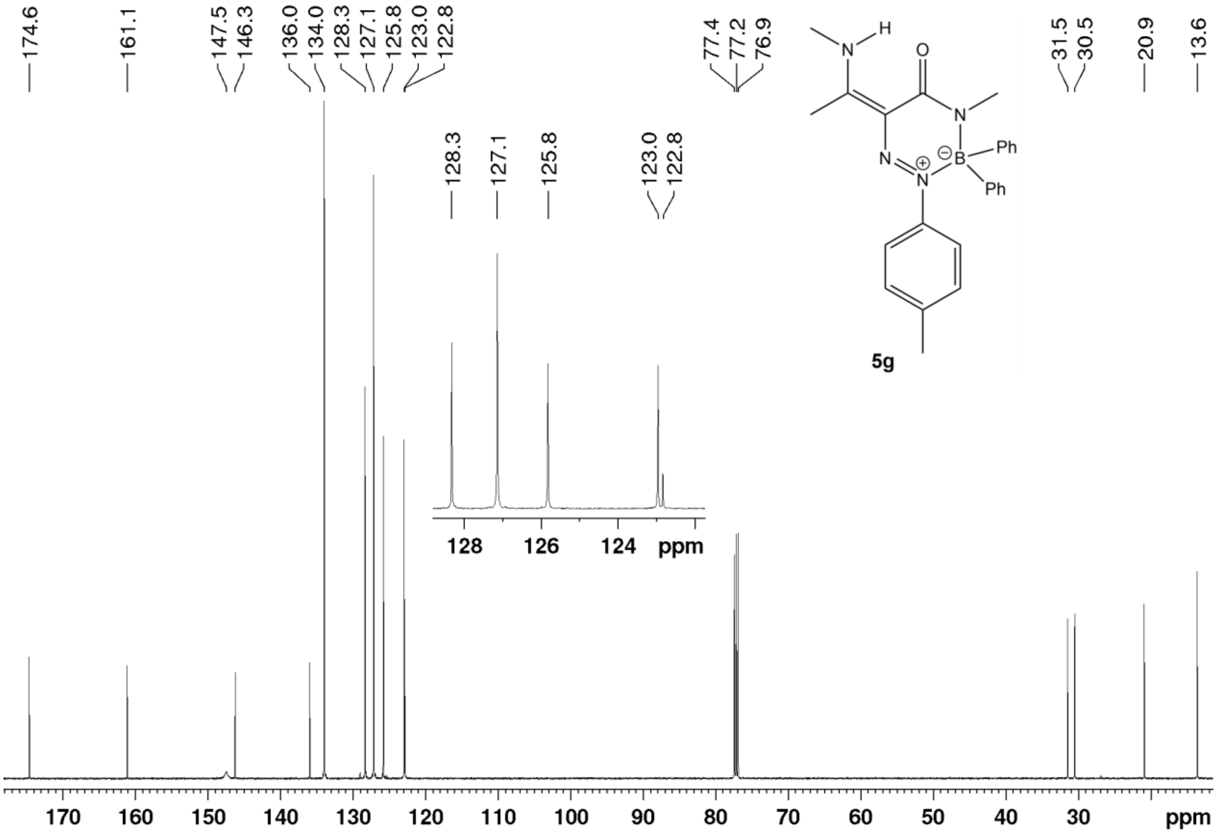

Figure S23. 125 MHz ^{13}C NMR of compound **5g** in $CDCl_3$

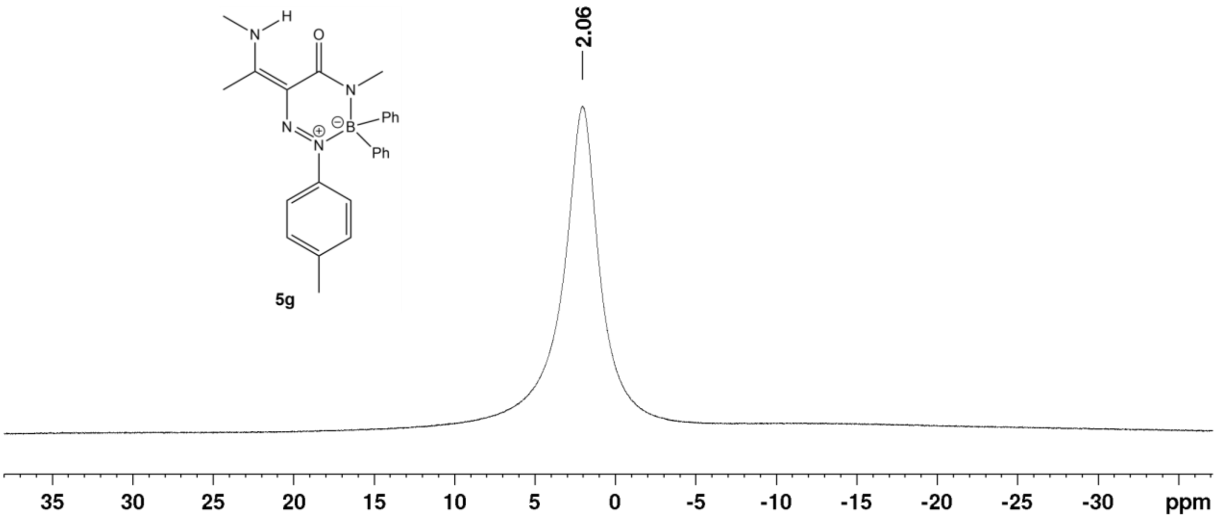

Figure S24. 160 MHz ^{11}B NMR of compound **5g** in $CDCl_3$

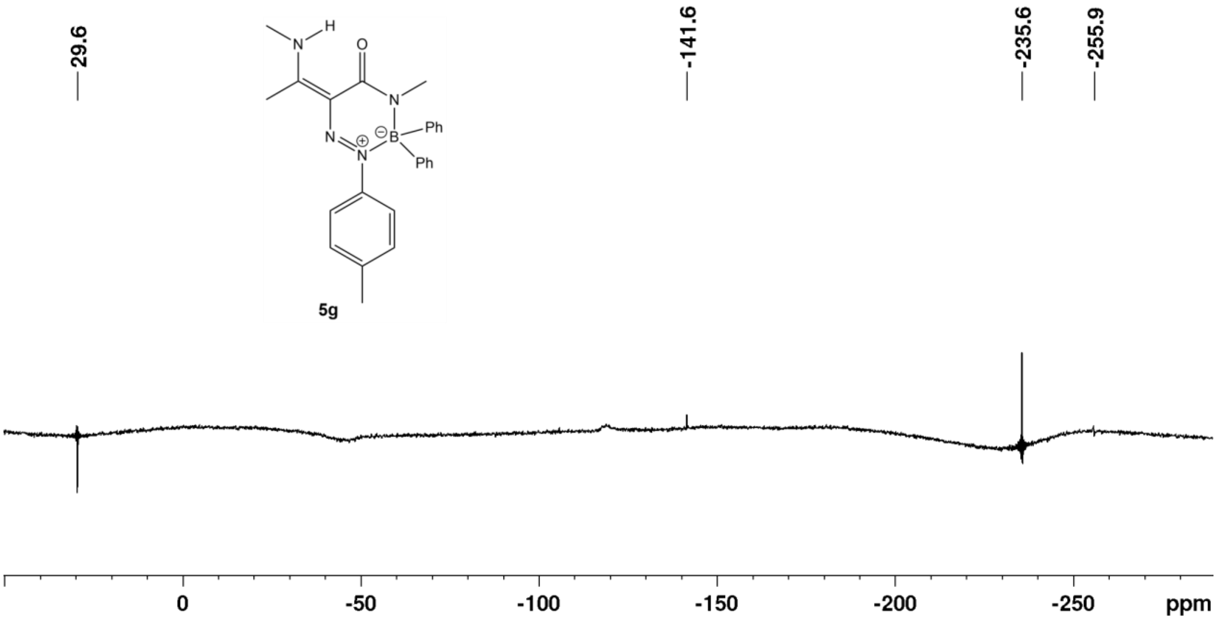

Figure S25. 50 MHz ^{15}N NMR of compound **5g** in $CDCl_3$

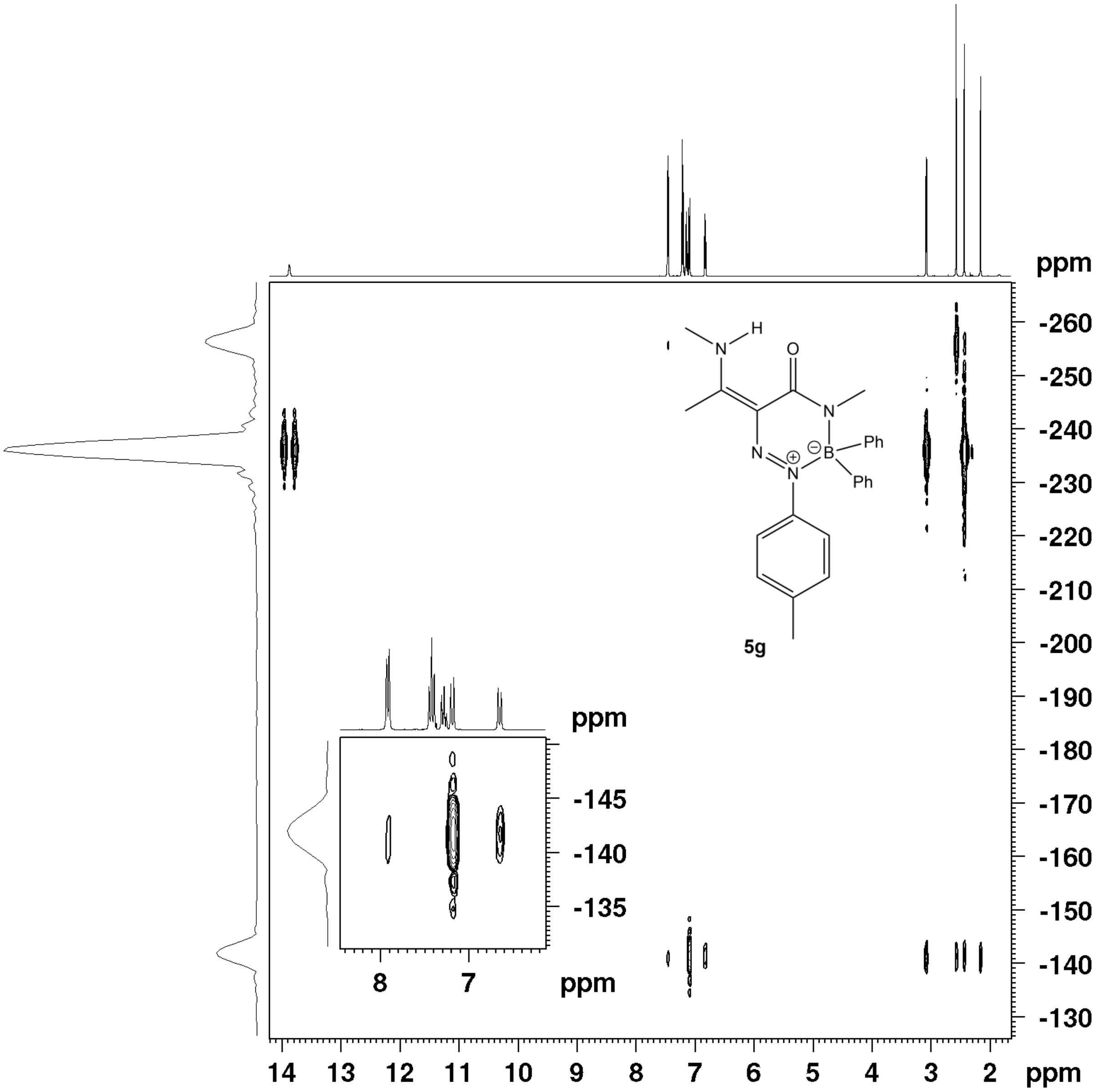

Figure S26. 500 MHz ^{1}H–^{15}N HMBC NMR of compound **5g** in $CDCl_3$

2.6 Triazaborinone 5h

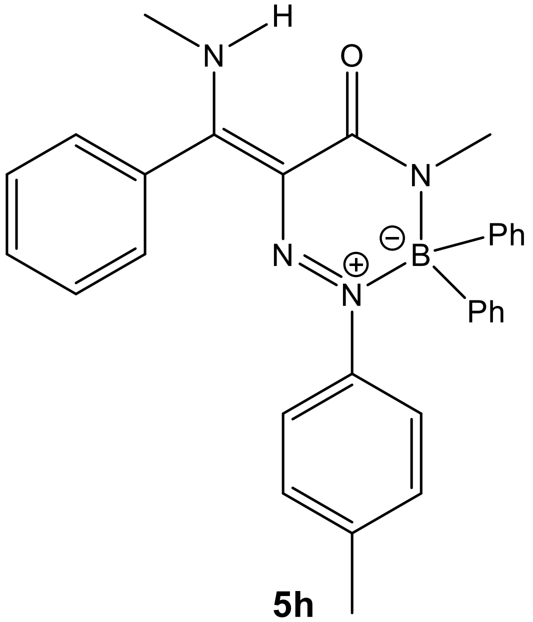

5h

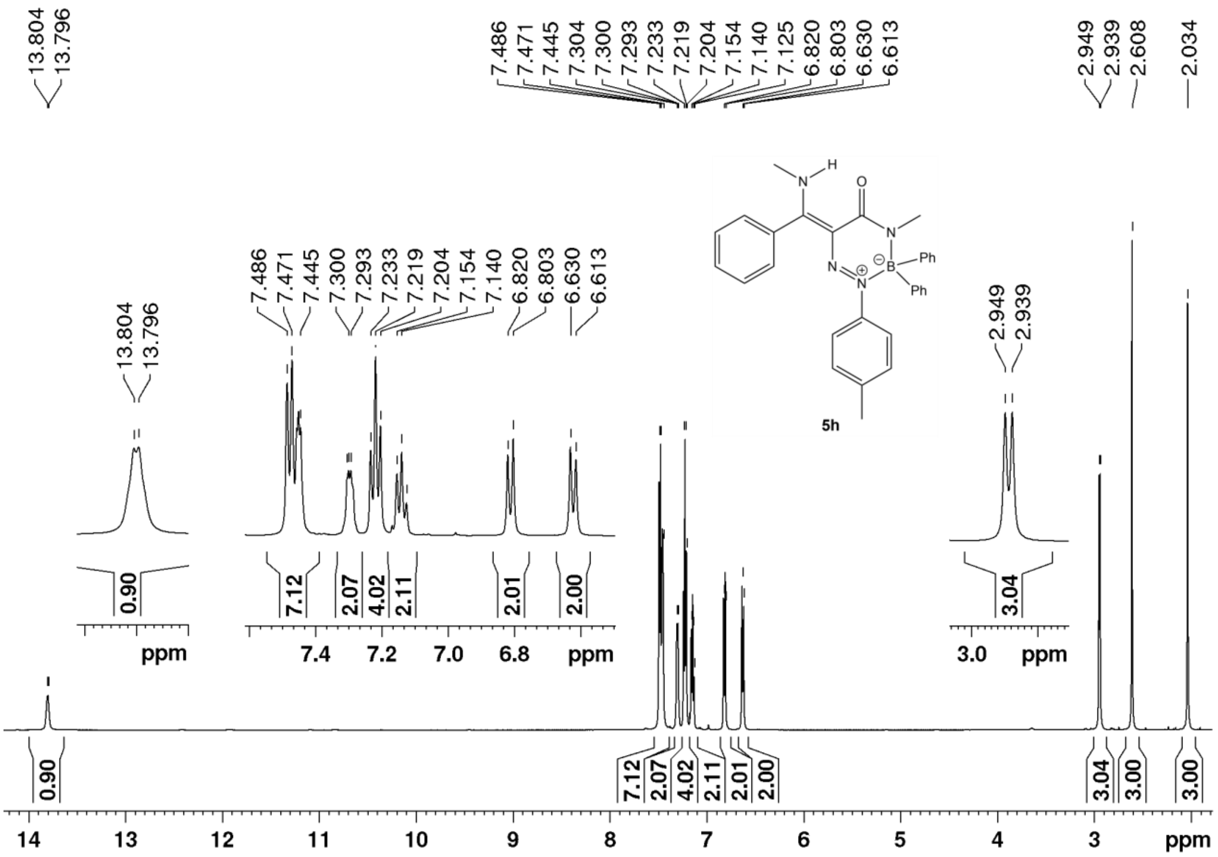

Figure S27. 500 MHz ^{1}H NMR of compound **5h** in $CDCl_3$

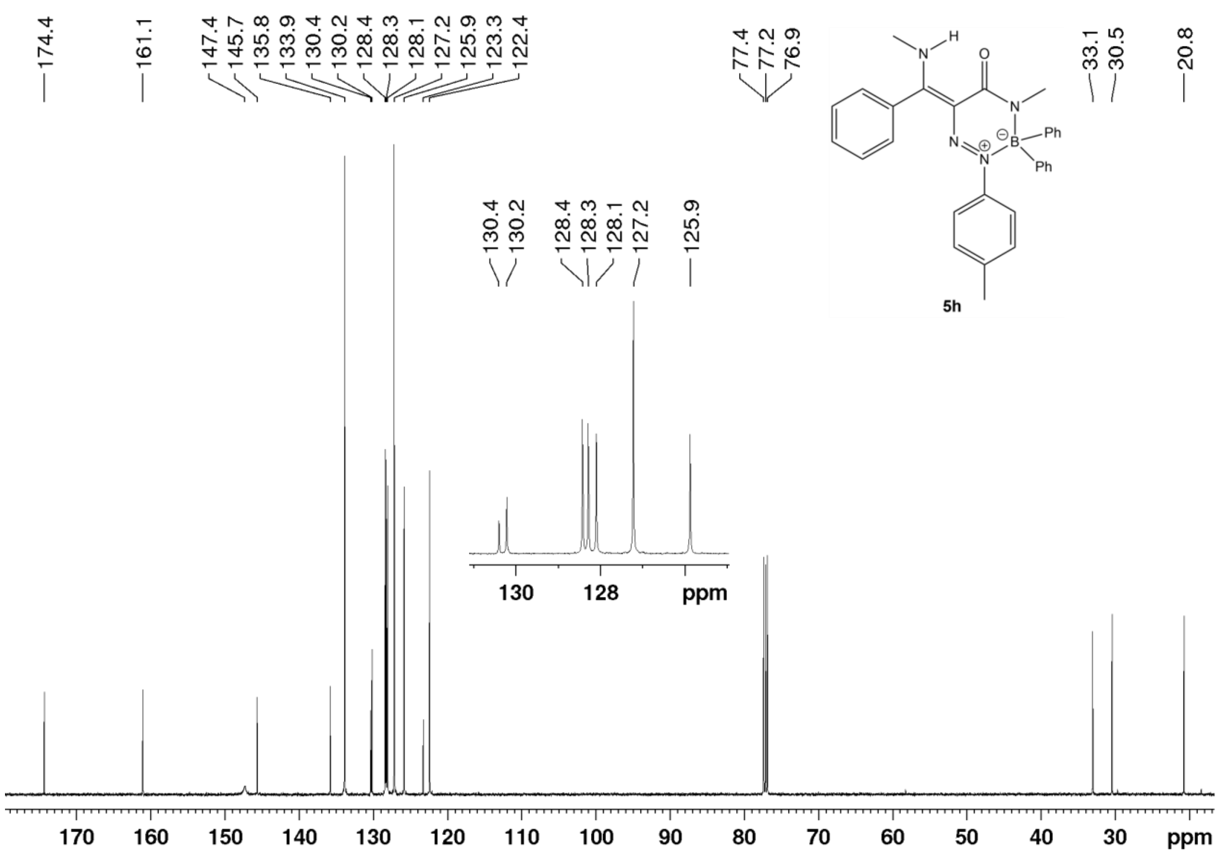

Figure S28. 125 MHz ^{13}C NMR of compound **5h** in $CDCl_3$

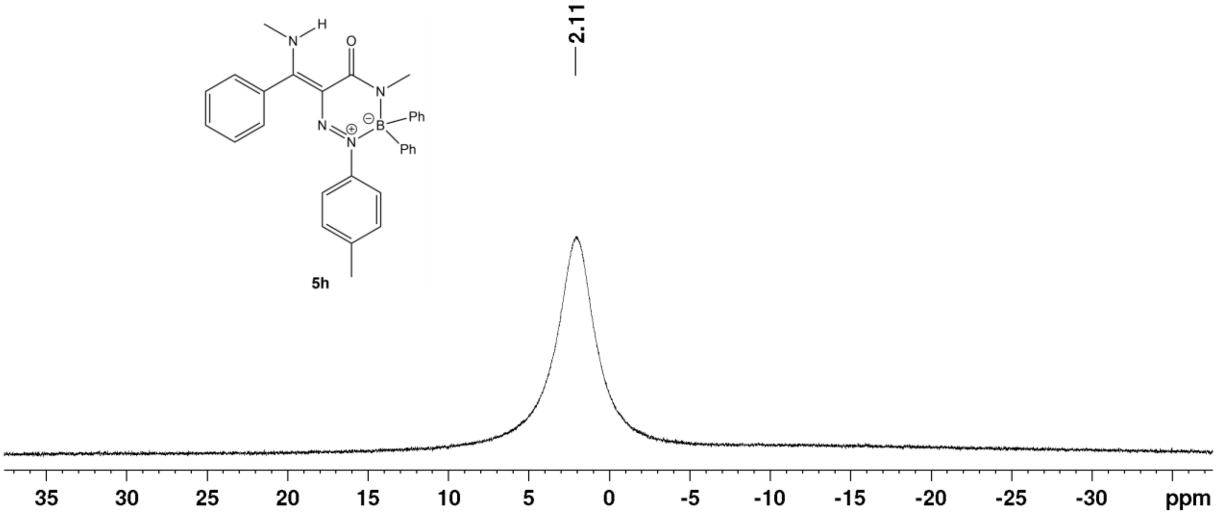

Figure S29. 160 MHz ^{11}B NMR of compound **5h** in $CDCl_3$

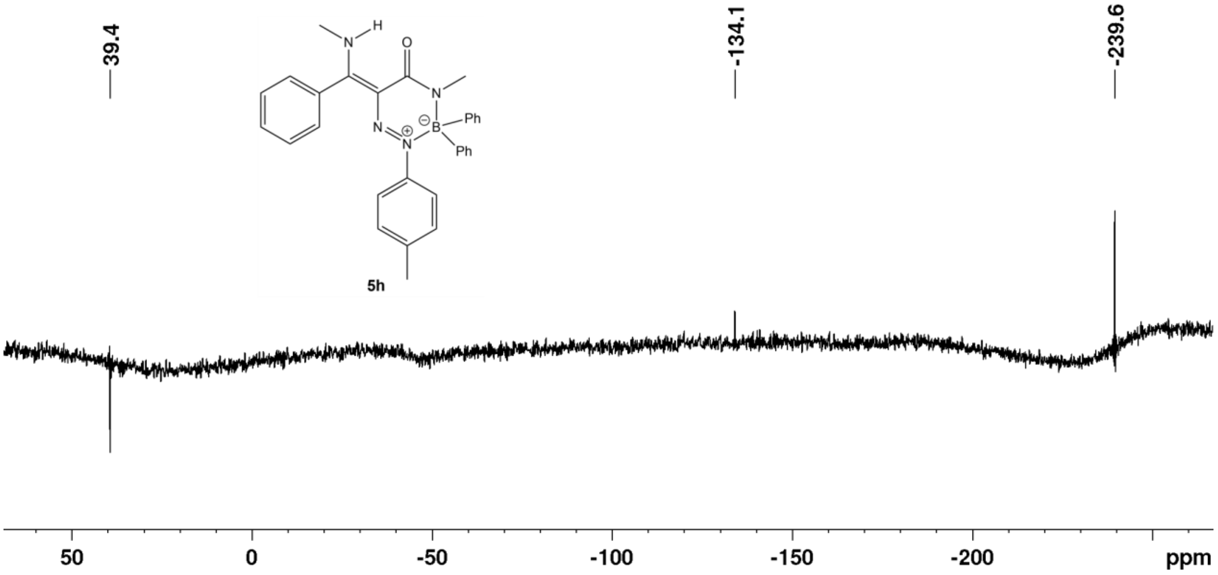

Figure S30. 50 MHz ^{15}N NMR of compound **5h** in $CDCl_3$

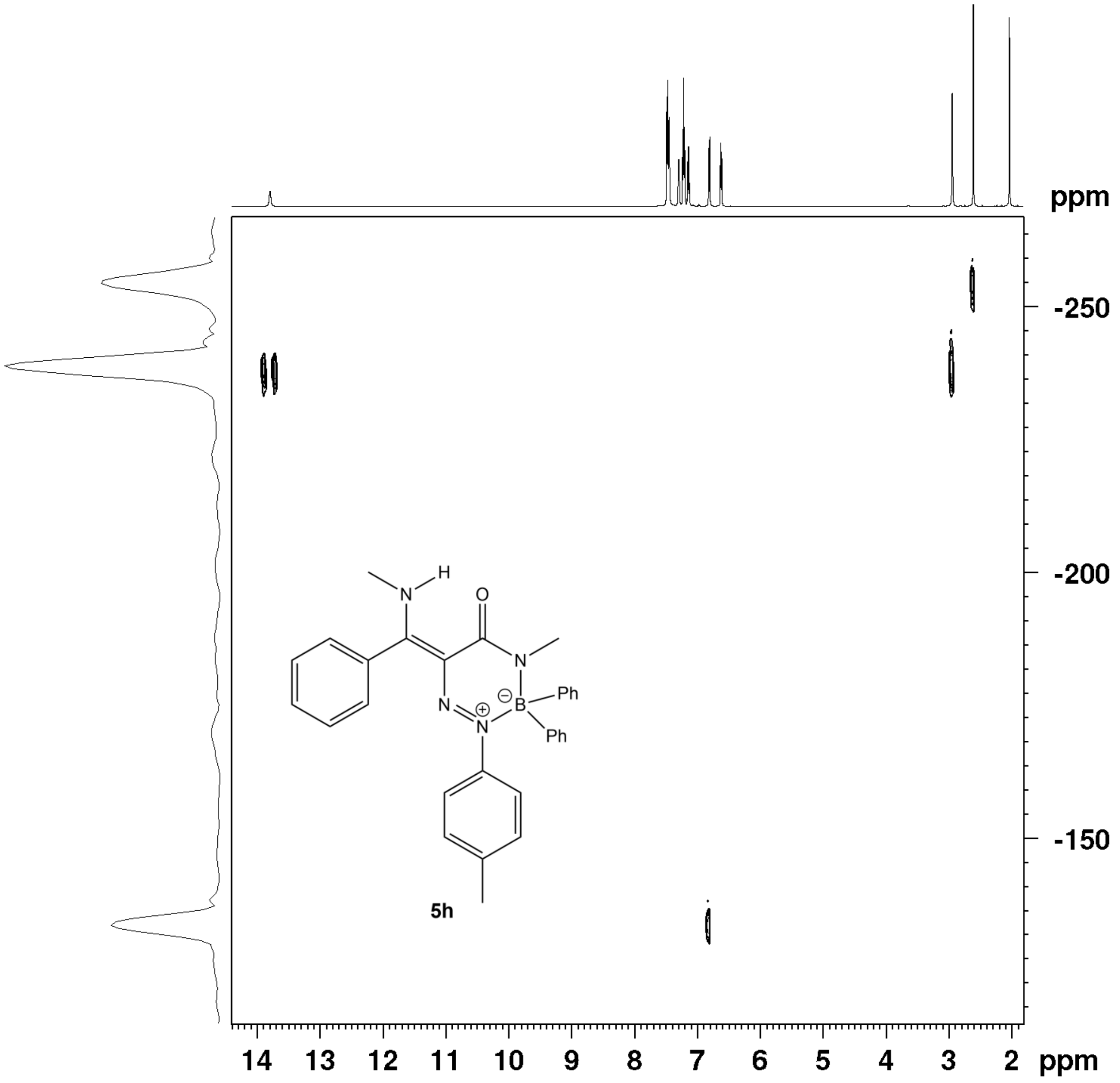

Figure S31. 500 MHz ^{1}H–^{15}N HMBC NMR of compound **5h** in $CDCl_3$

2.7 Oxazaborine 6a

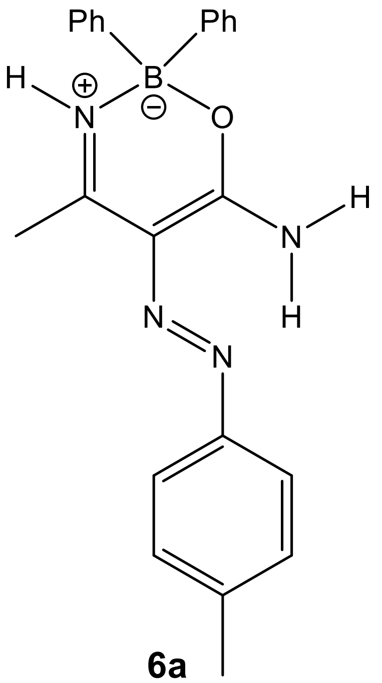

6a

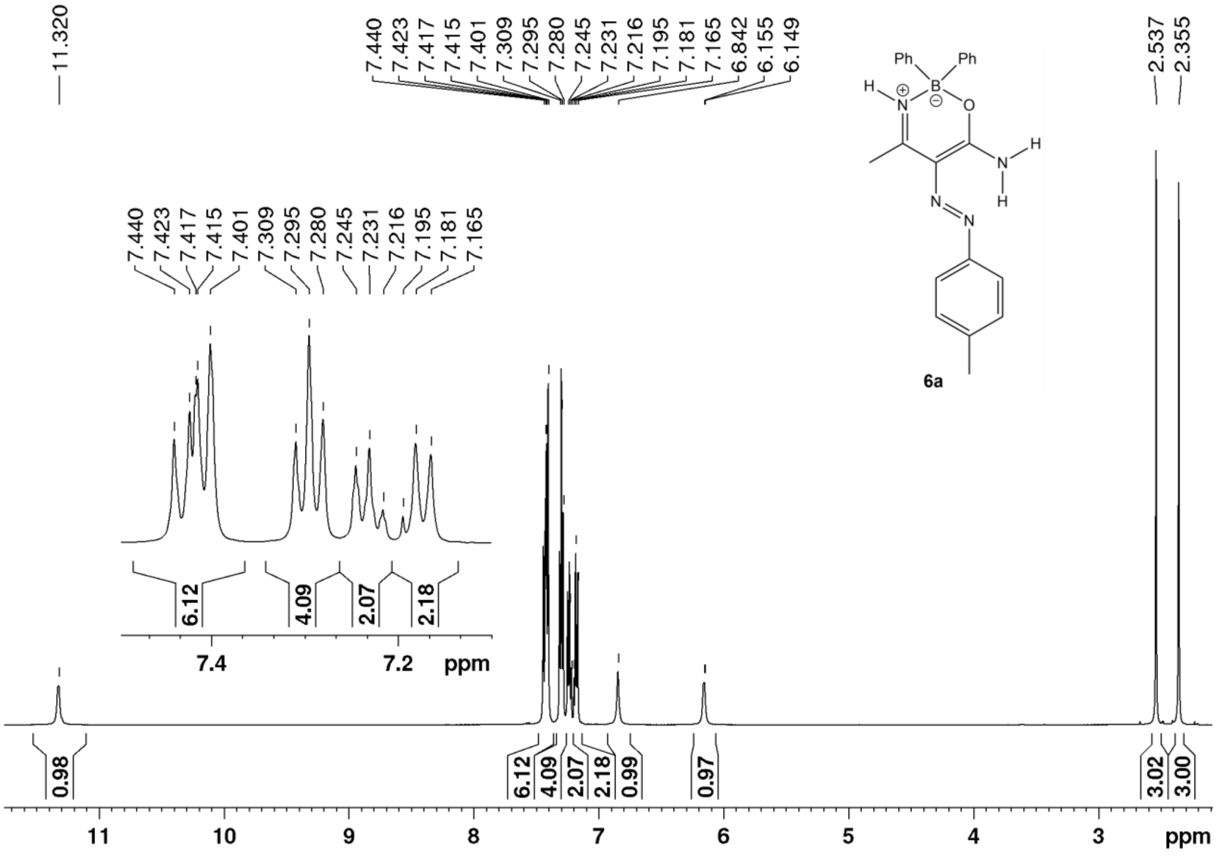

Figure S32. 500 MHz ^{1}H NMR of compound **6a** in $CDCl_3$

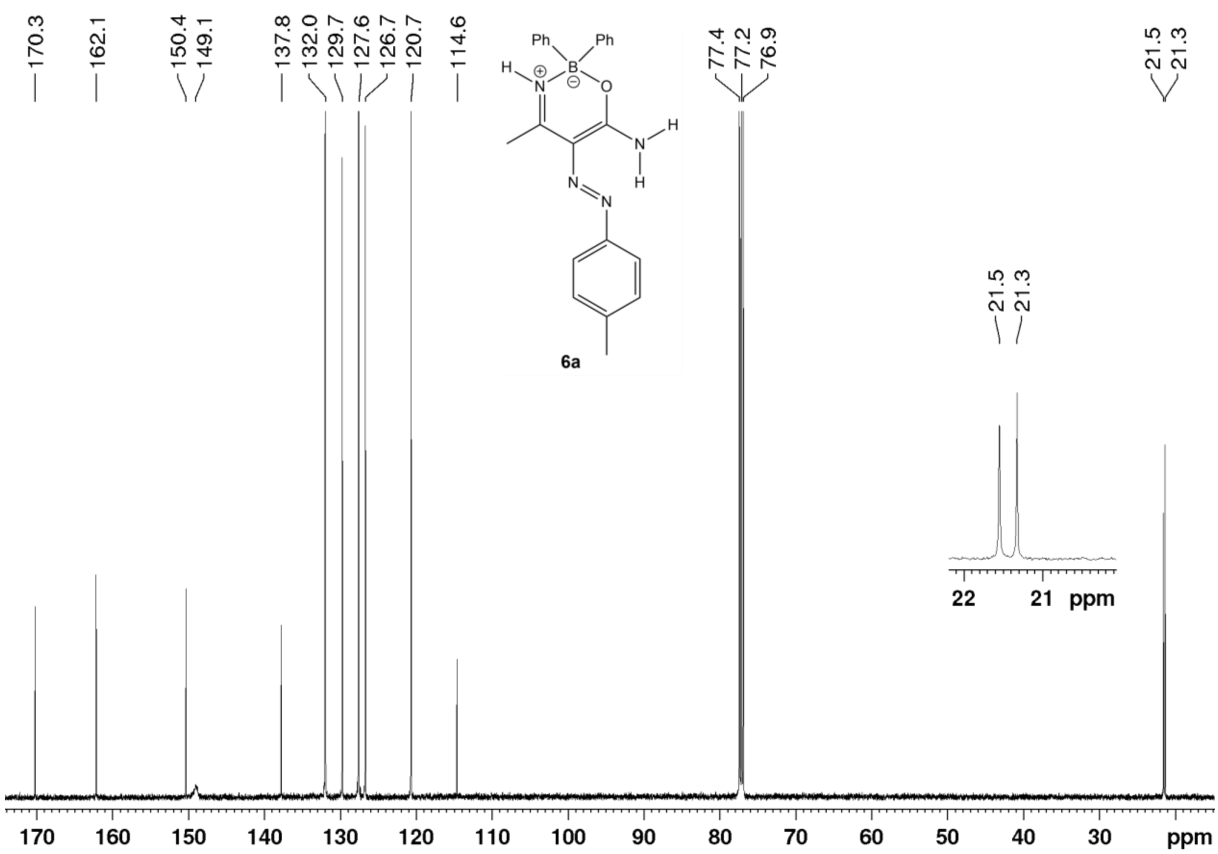

Figure S33. 125 MHz ^{13}C NMR of compound **6a** in $CDCl_3$

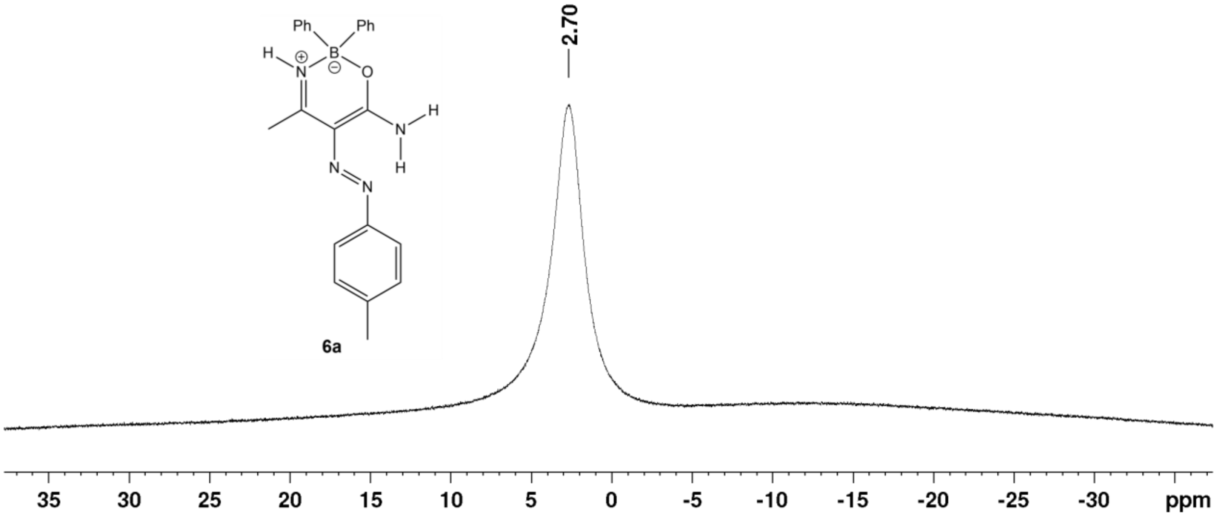

Figure S34. 160 MHz ^{11}B NMR of compound **6a** in $CDCl_3$

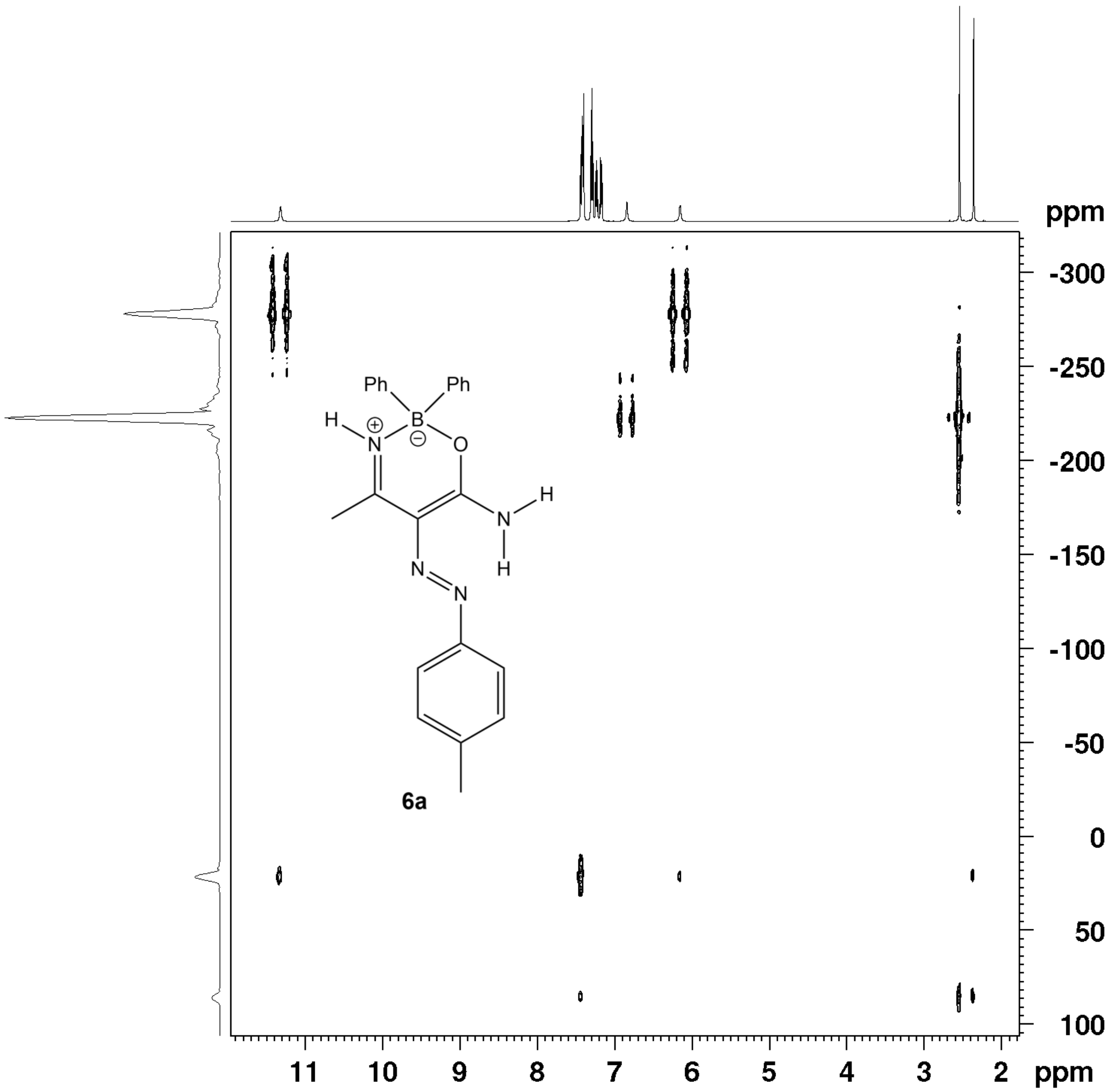

Figure S35. 500 MHz ^{1}H–^{15}N HMBC NMR of compound **6a** in $CDCl_3$

2.8 Oxazaborine 6e

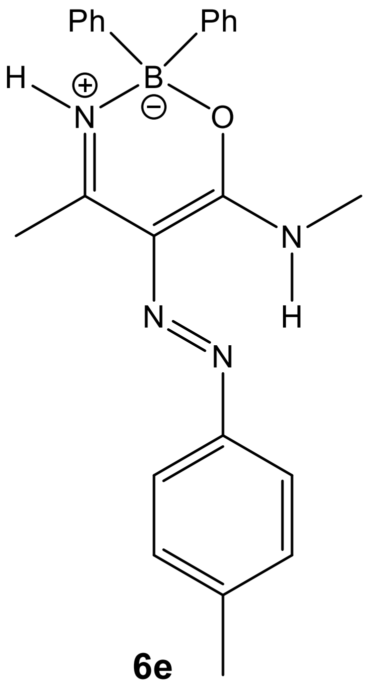

6e

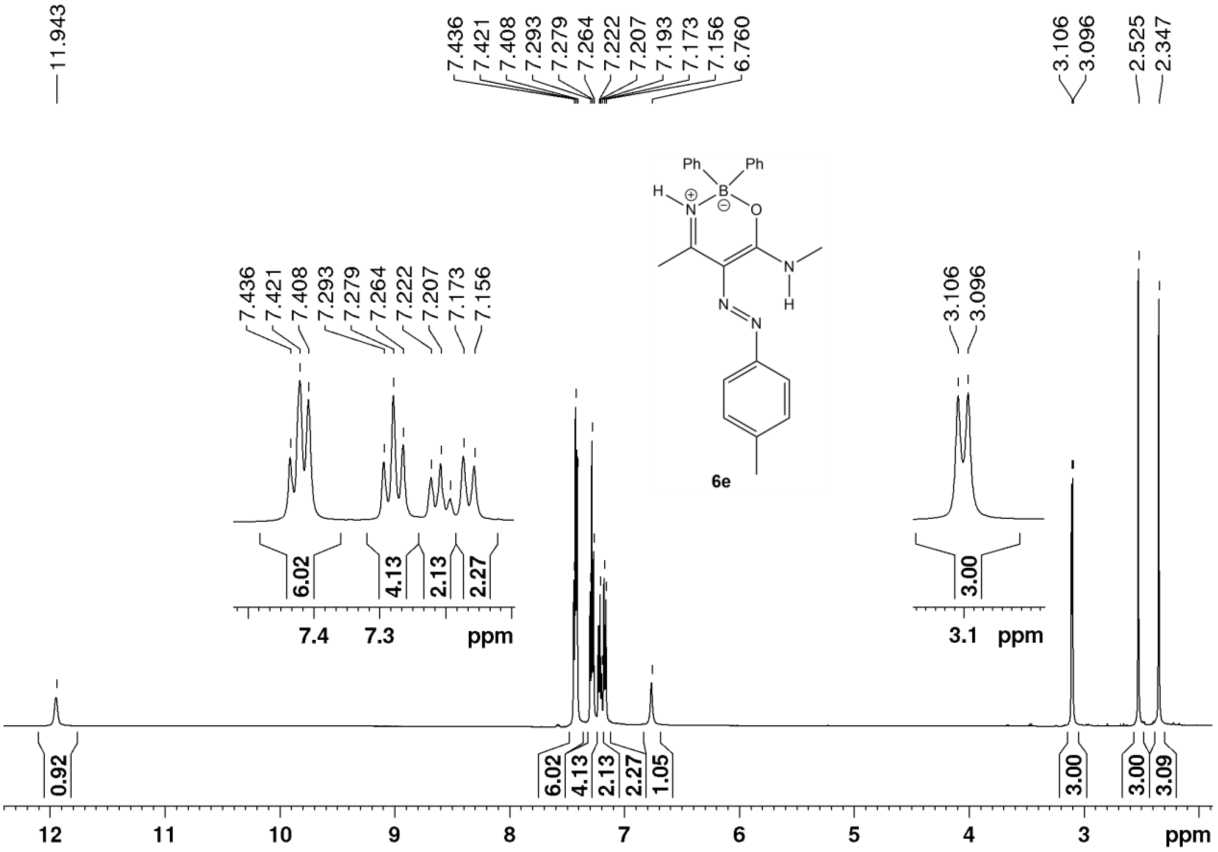

Figure S36. 500 MHz ^{1}H NMR of compound **6e** in $CDCl_3$

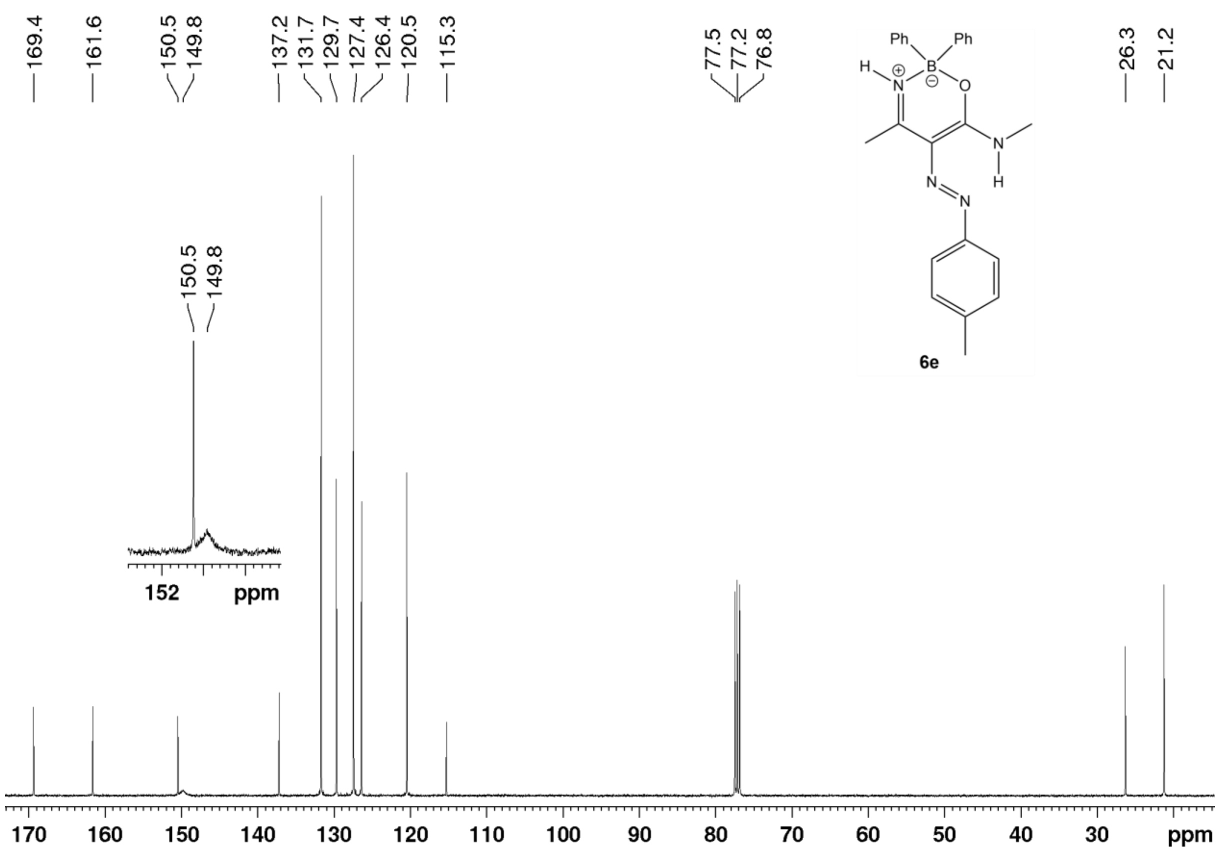

Figure S37. 100 MHz ^{13}C NMR of compound **6e** in $CDCl_3$

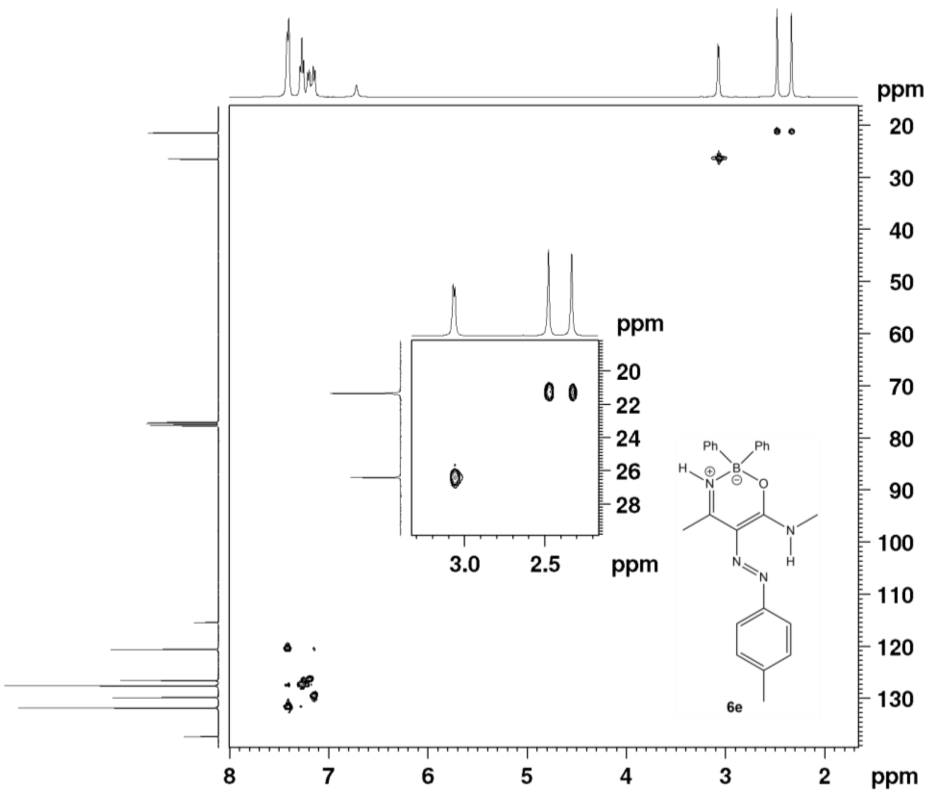

Figure S38. 100 MHz ^{1}H-^{13}C HSQC NMR of compound **6e** in $CDCl_3$

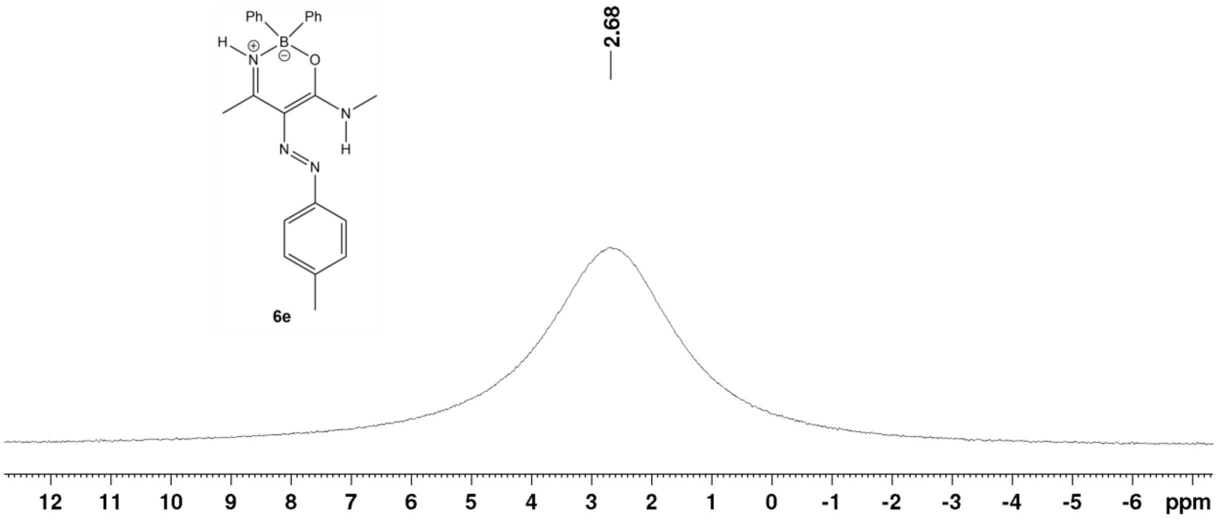

Figure S39. 160 MHz ^{11}B NMR of compound **6e** in $CDCl_3$

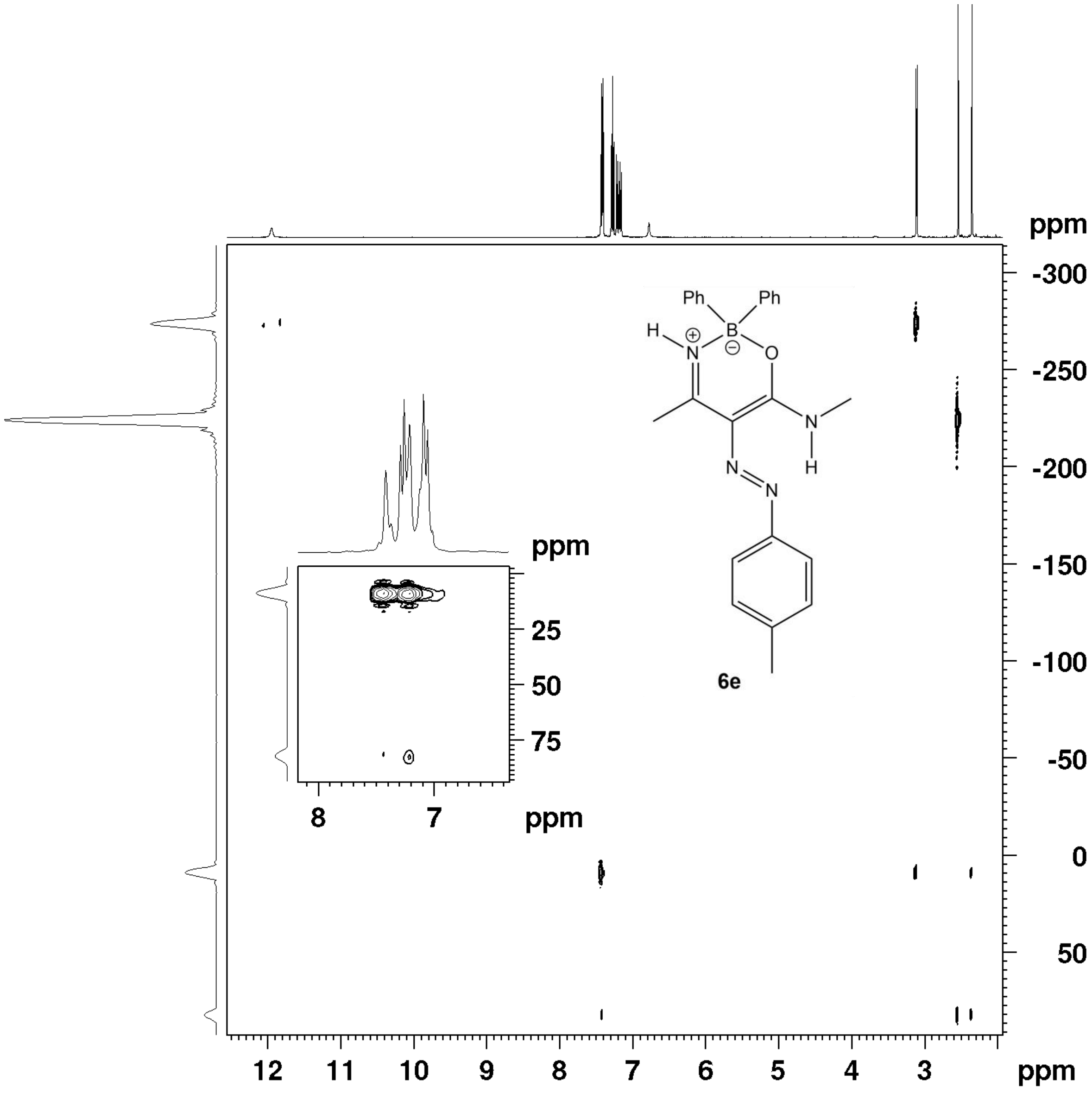

Figure S40. 500 MHz ^{1}H–^{15}N HMBC NMR of compound **6e** in $CDCl_3$

2.9 Oxazaborine 6f

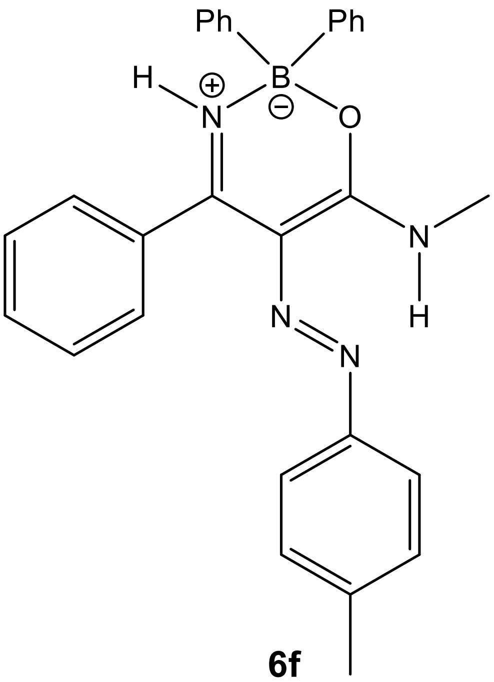

6f

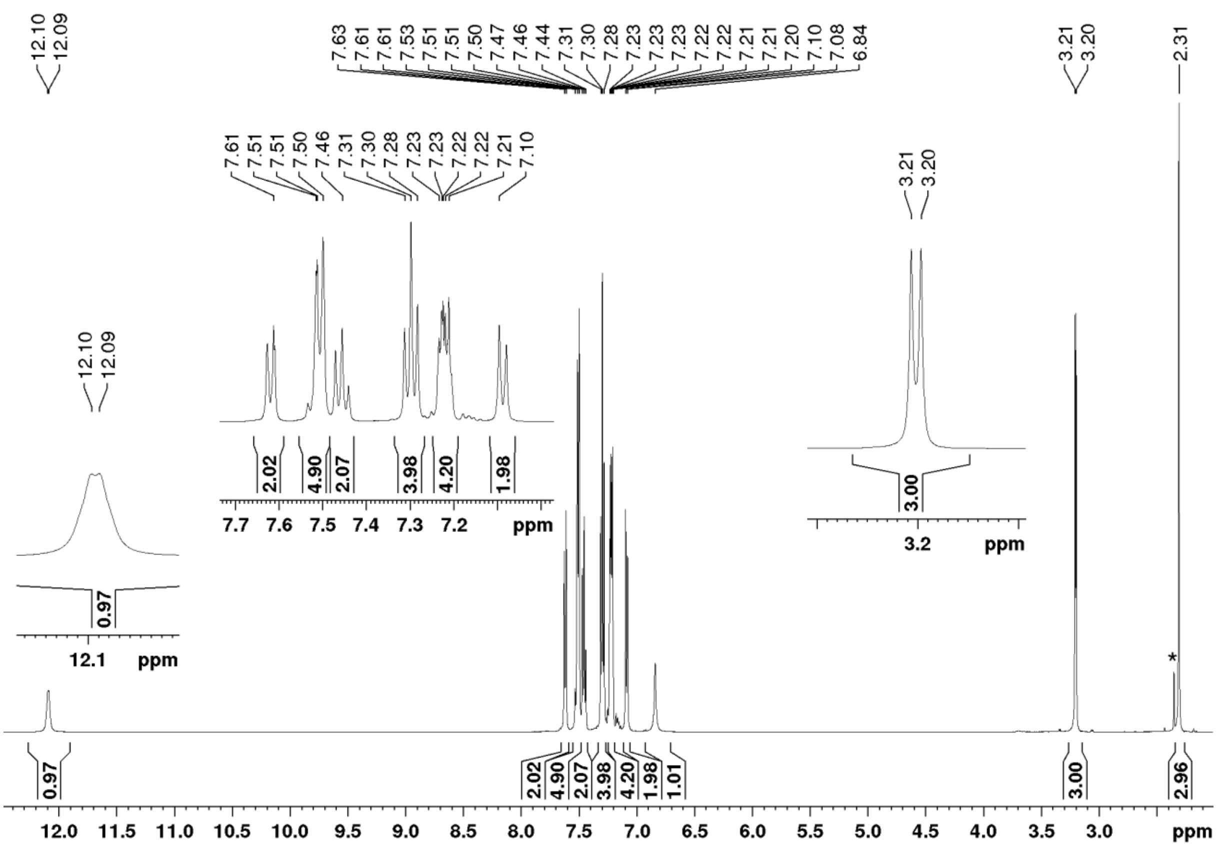

Figure S41. 500 MHz ^{1}H NMR of compound **6f** in $CDCl_3$ (Signal with asterix is $–CH_3$ from toluene.)

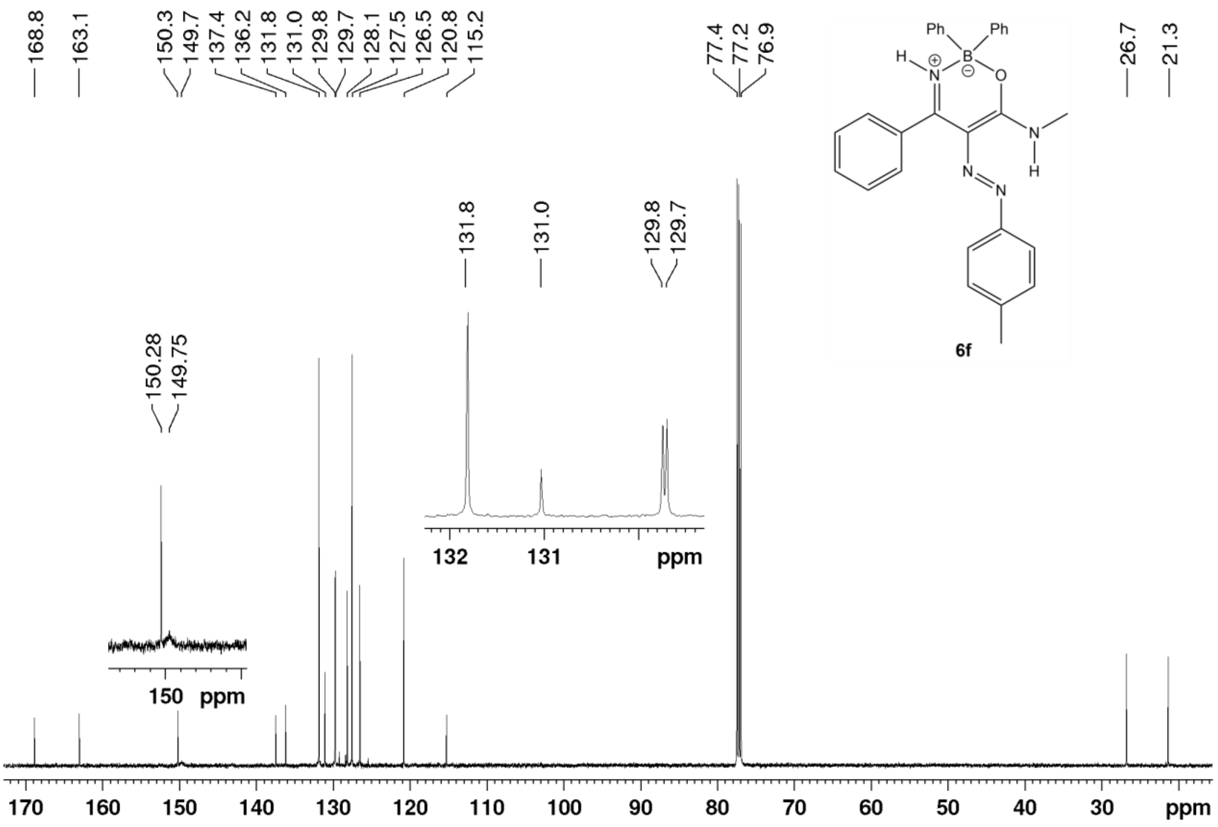

Figure S42. 125 MHz ^{13}C NMR of compound **6f** in $CDCl_3$

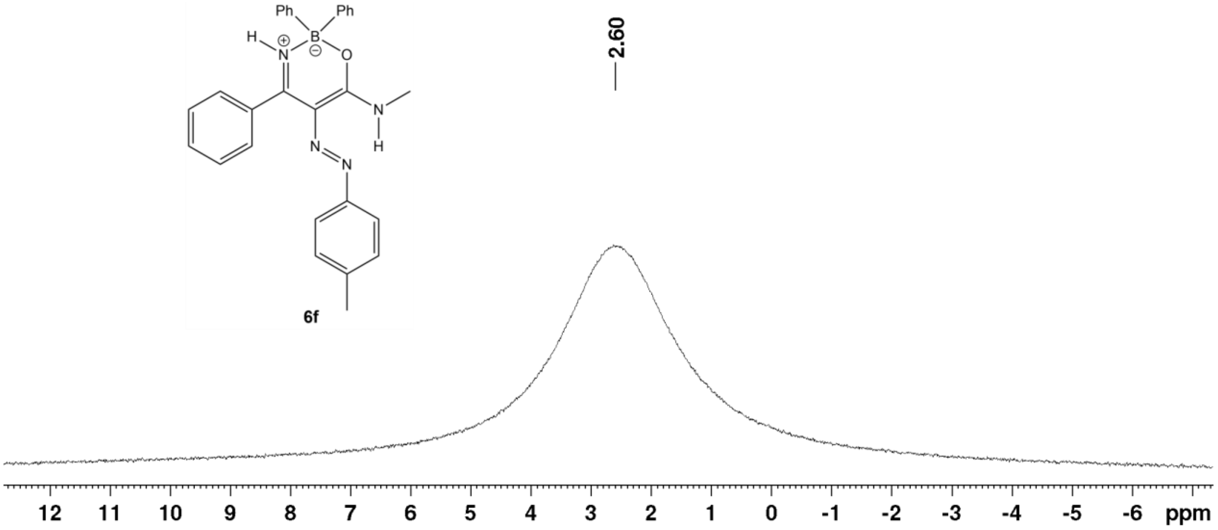

Figure S43. 160 MHz ^{11}B NMR of compound **6f** in $CDCl_3$

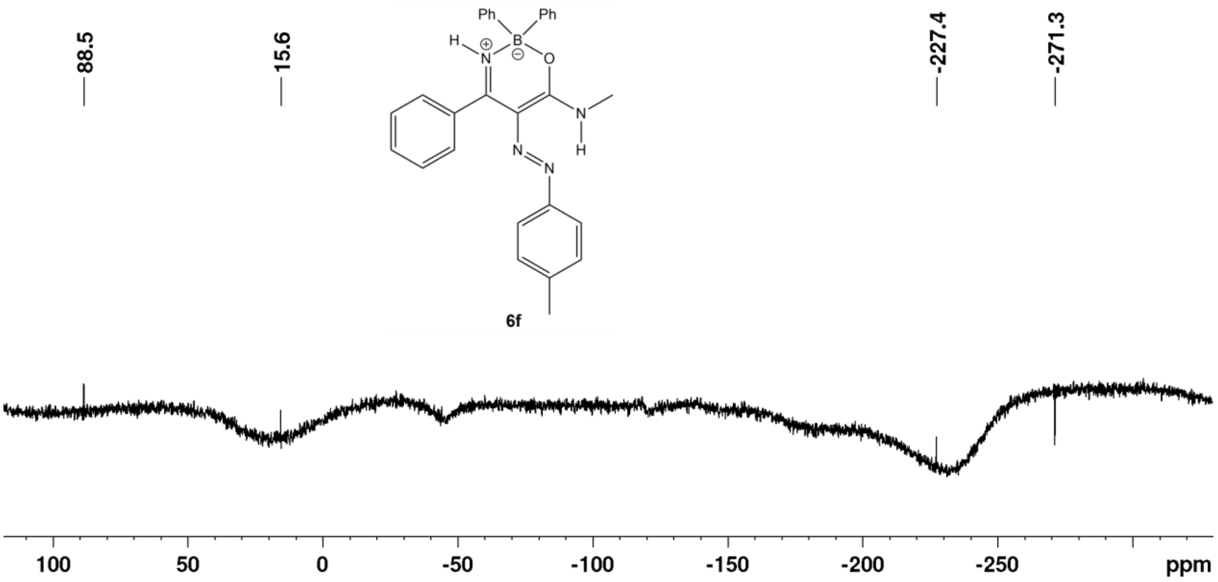

Figure S44. 50 MHz ^{15}N NMR of compound **6f** in $CDCl_3$

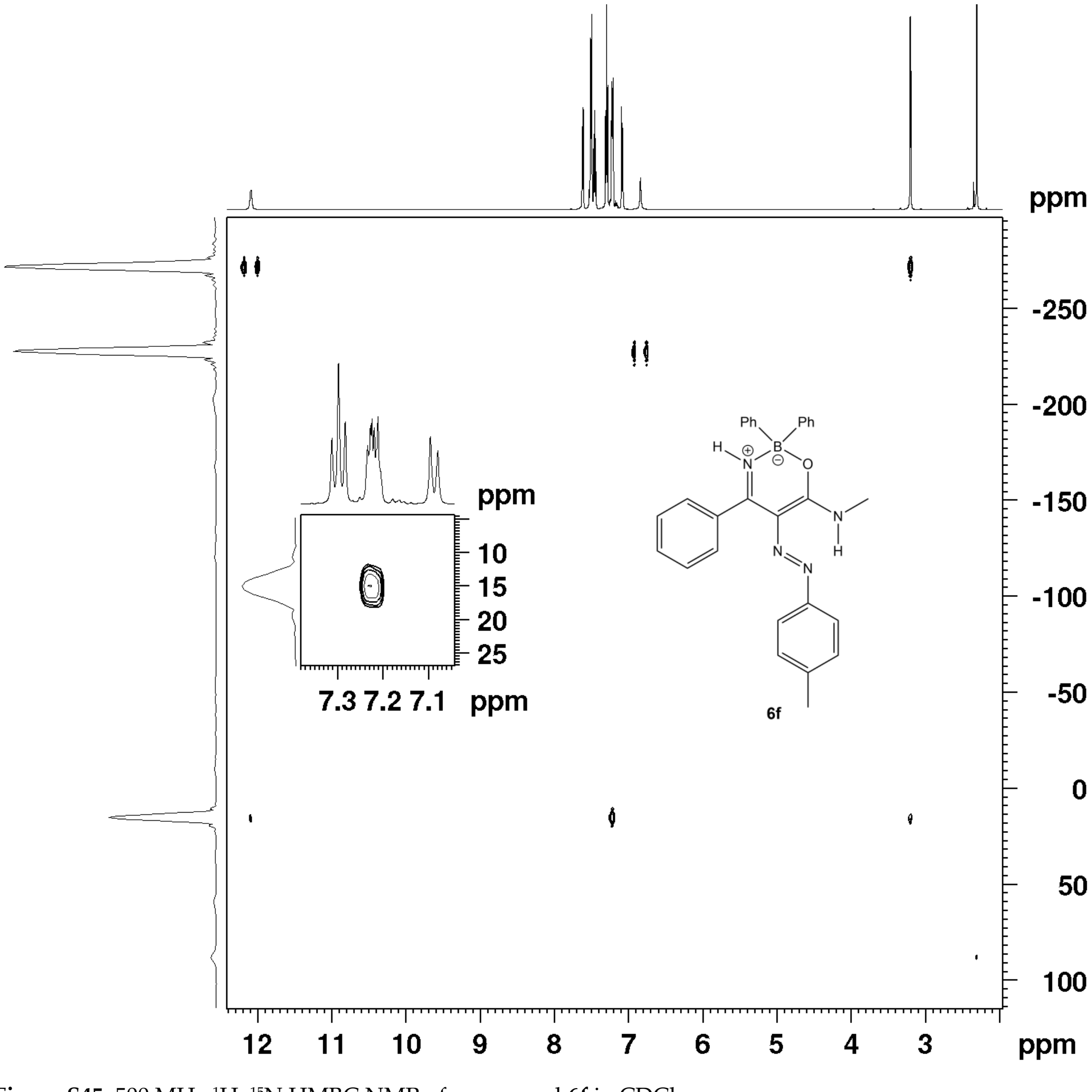

Figure S45. 500 MHz ^{1}H–^{15}N HMBC NMR of compound **6f** in $CDCl_3$

2.10 Oxazaborine 6g

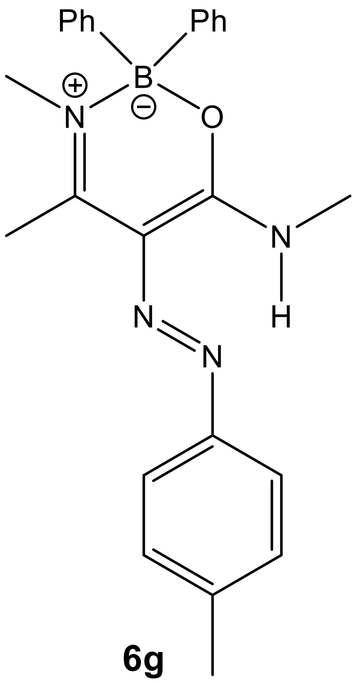

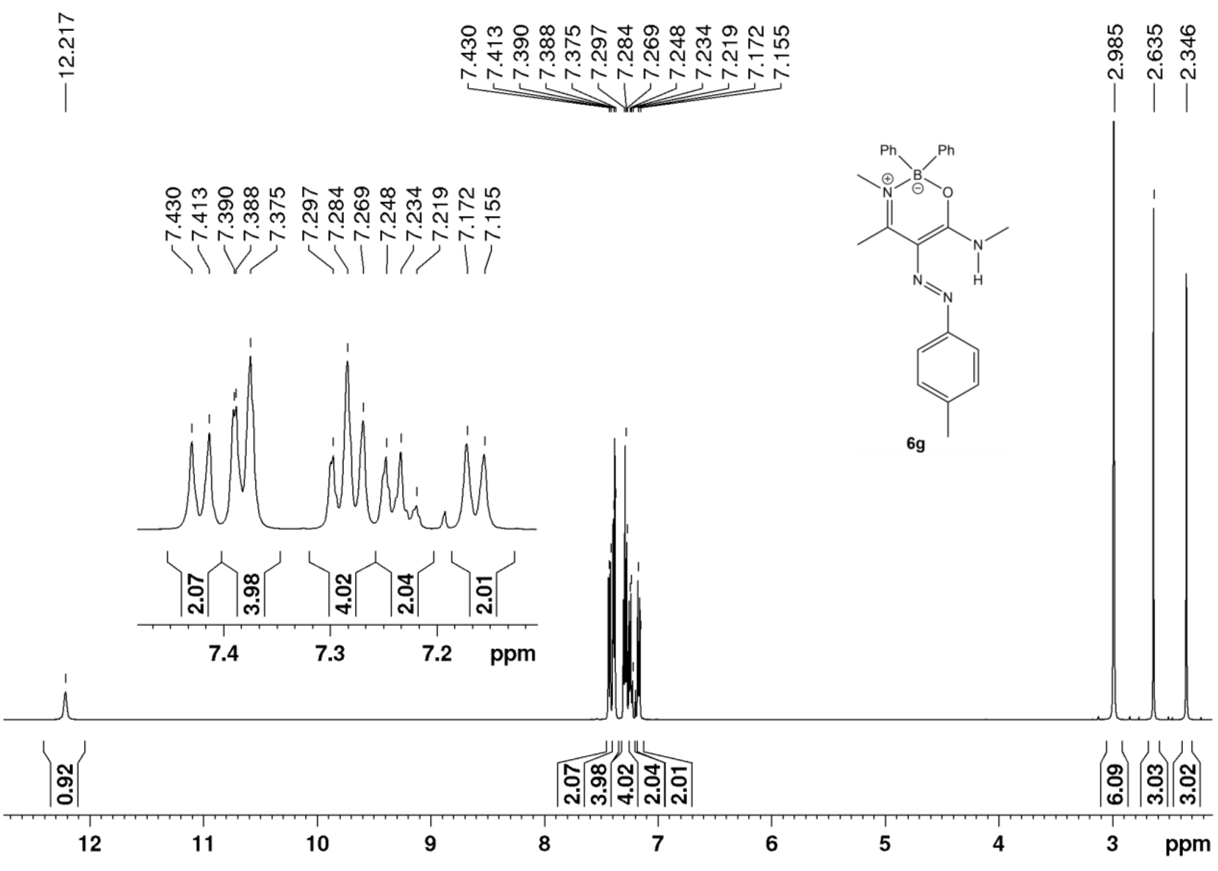

Figure S46. 500 MHz ^{1}H NMR of compound **6g** in $CDCl_3$

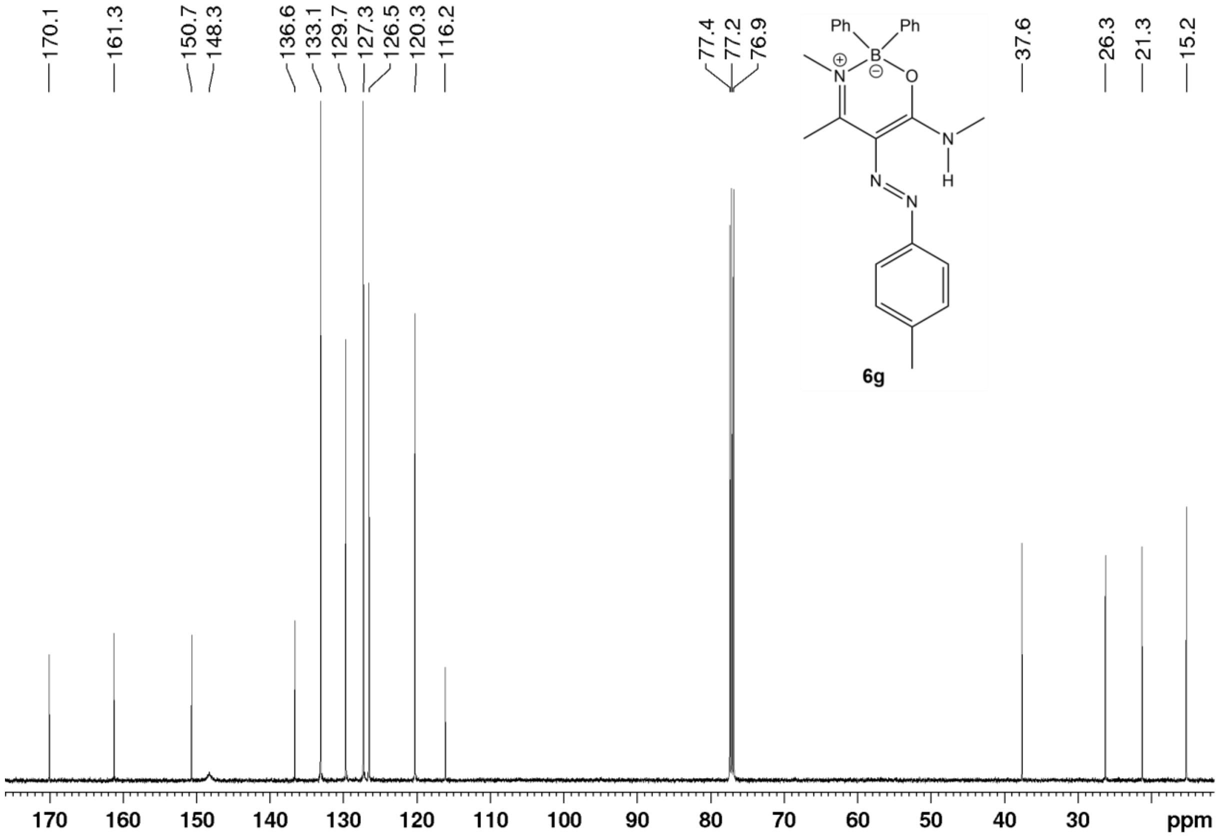

Figure S47. 125 MHz ^{13}C NMR of compound **6g** in $CDCl_3$

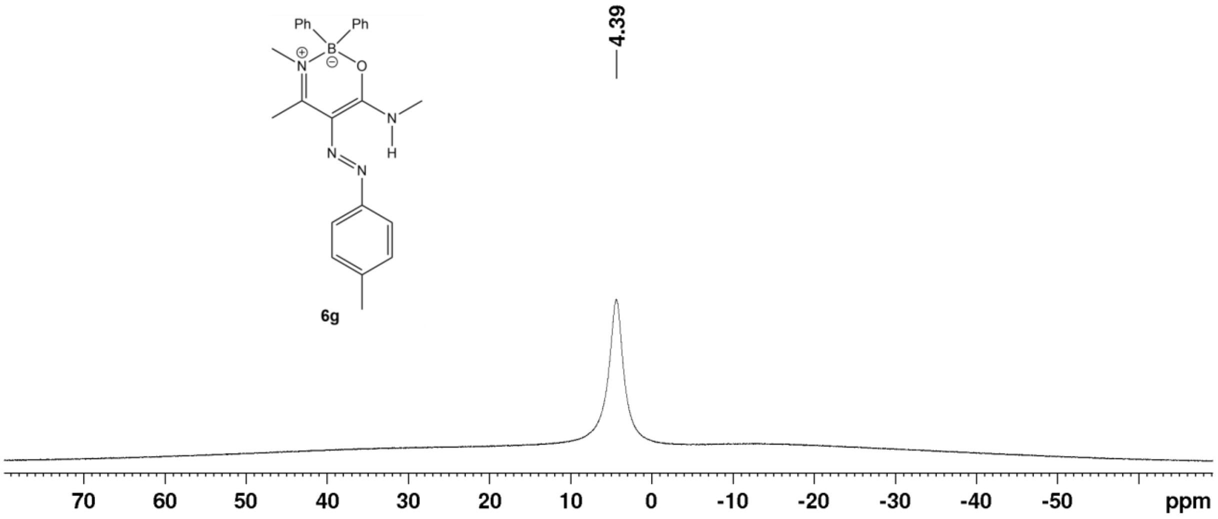

Figure S48. 160 MHz ^{11}B NMR of compound **6g** in $CDCl_3$

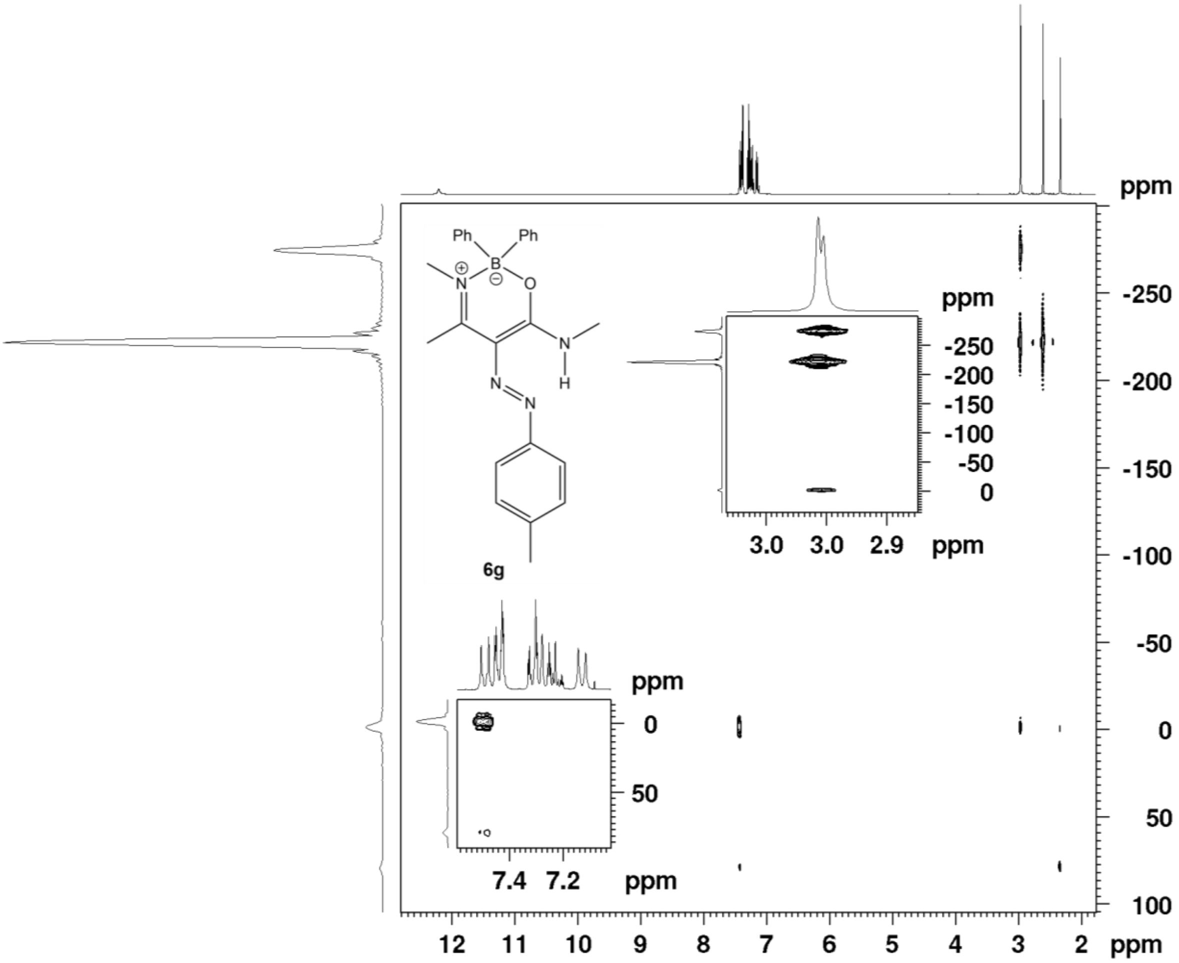

Figure S49. 400 MHz ^{1}H–^{15}N HMBC NMR of compound **6g** in $CDCl_3$

2.11 Oxazaborine 6h

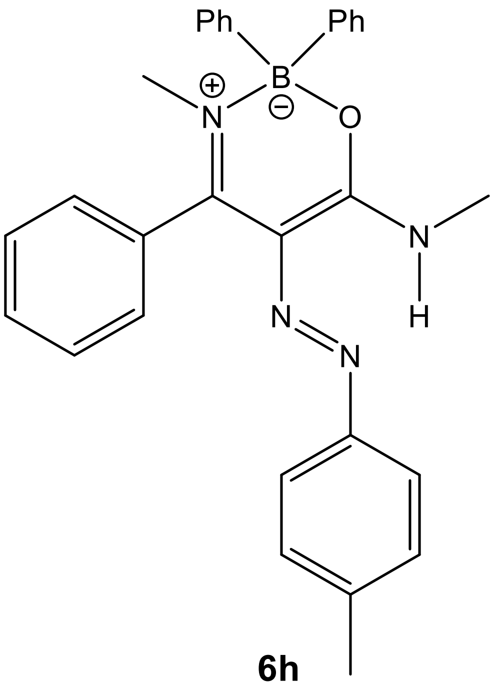

6h

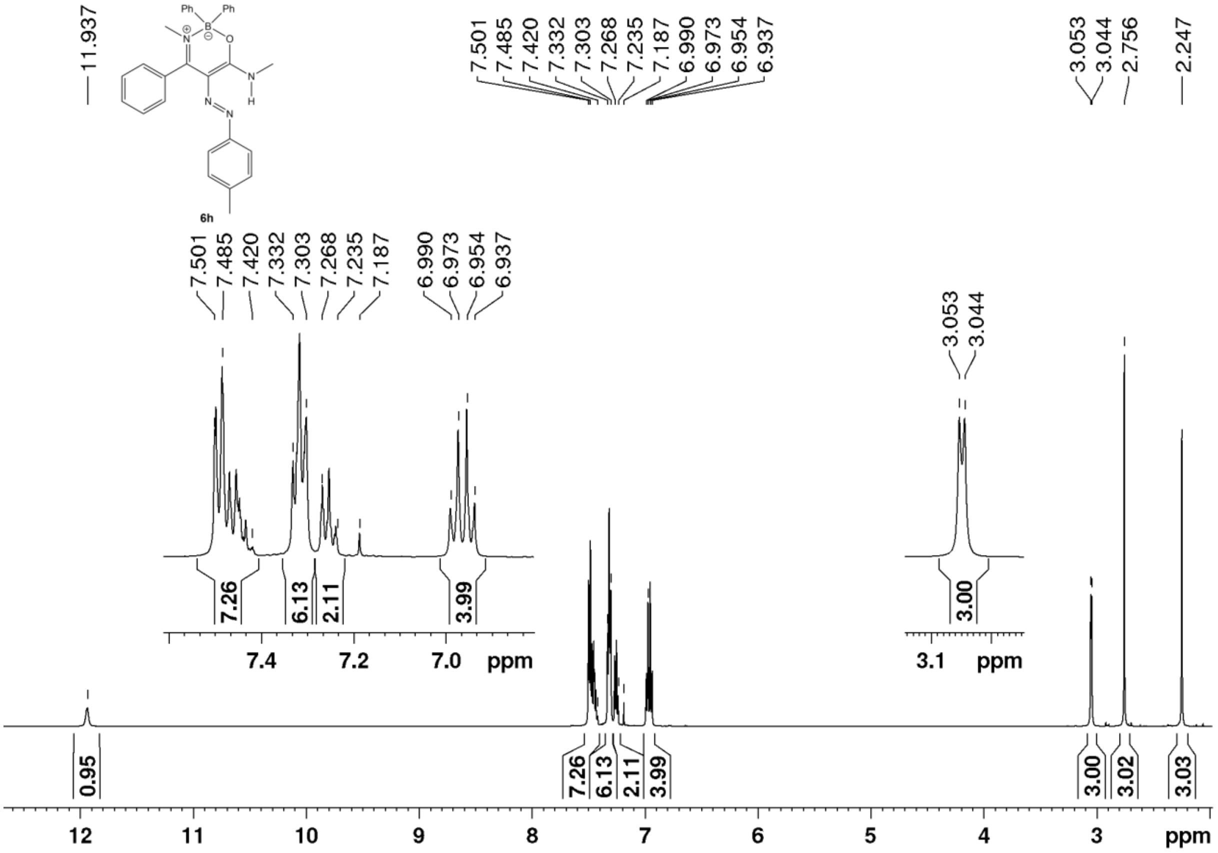

Figure S50. 500 MHz ^1H NMR of compound **6h** in $CDCl_3$

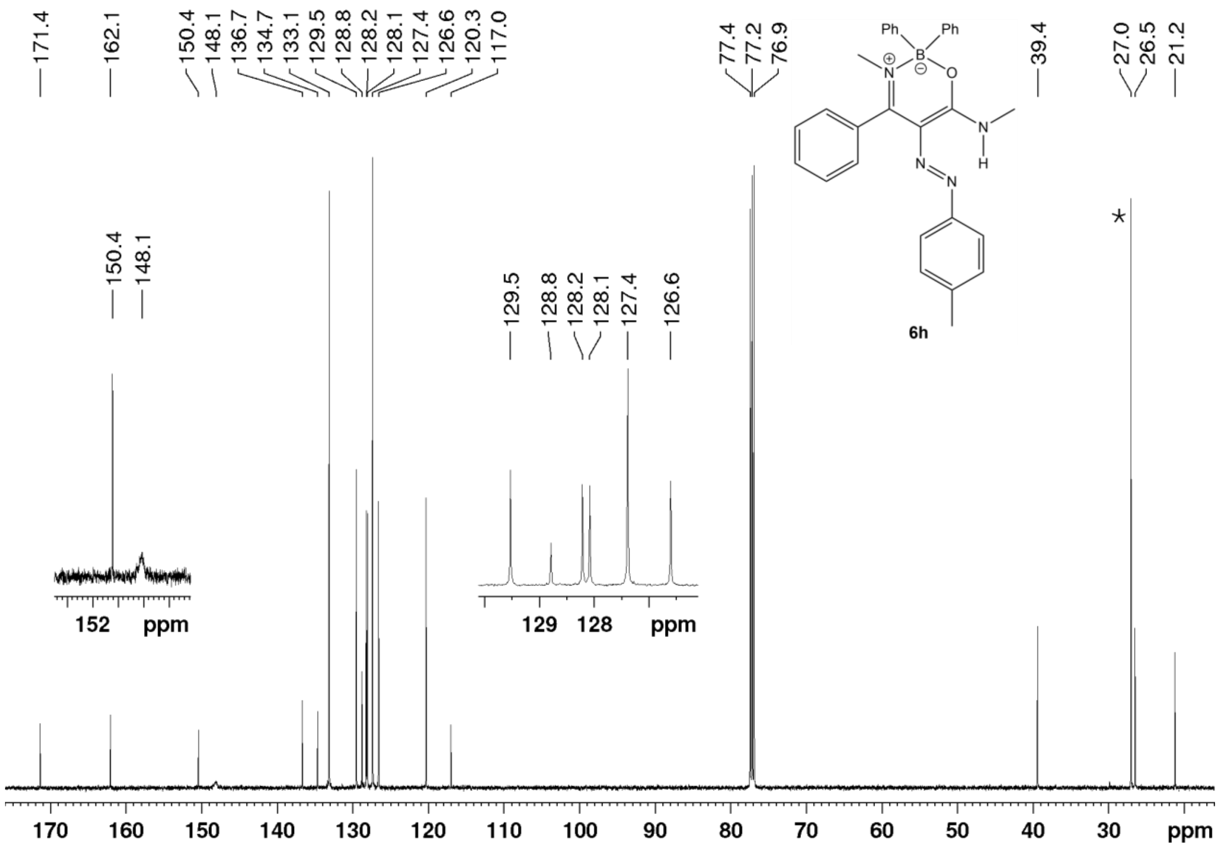

Figure S51. 125 MHz ^{13}C NMR of compound **6h** in $CDCl_3$ (*Cyclohexane)

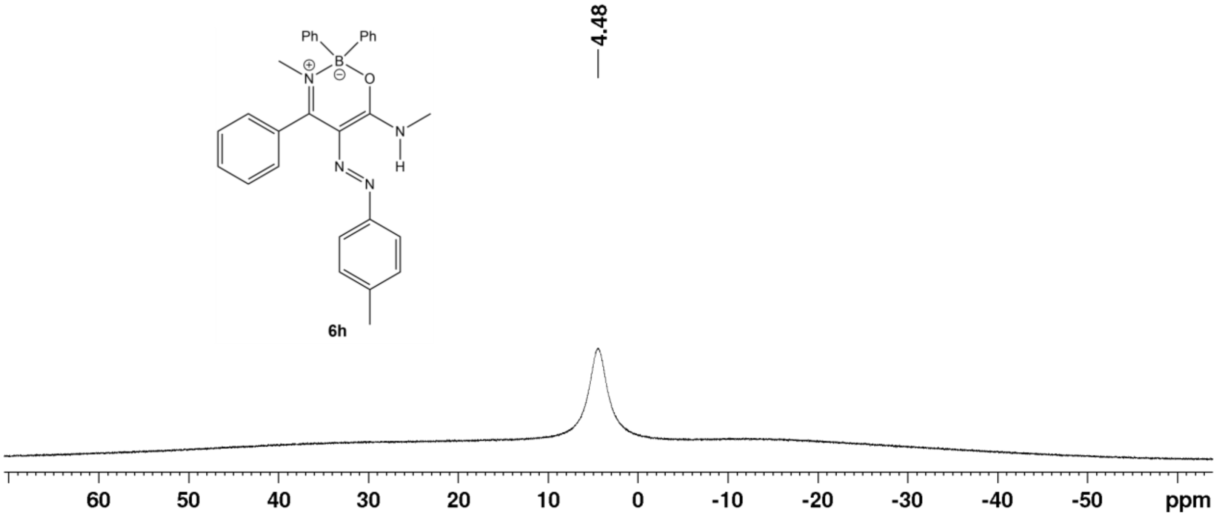

Figure S52. 160 MHz ^{11}B NMR of compound **6h** in $CDCl_3$

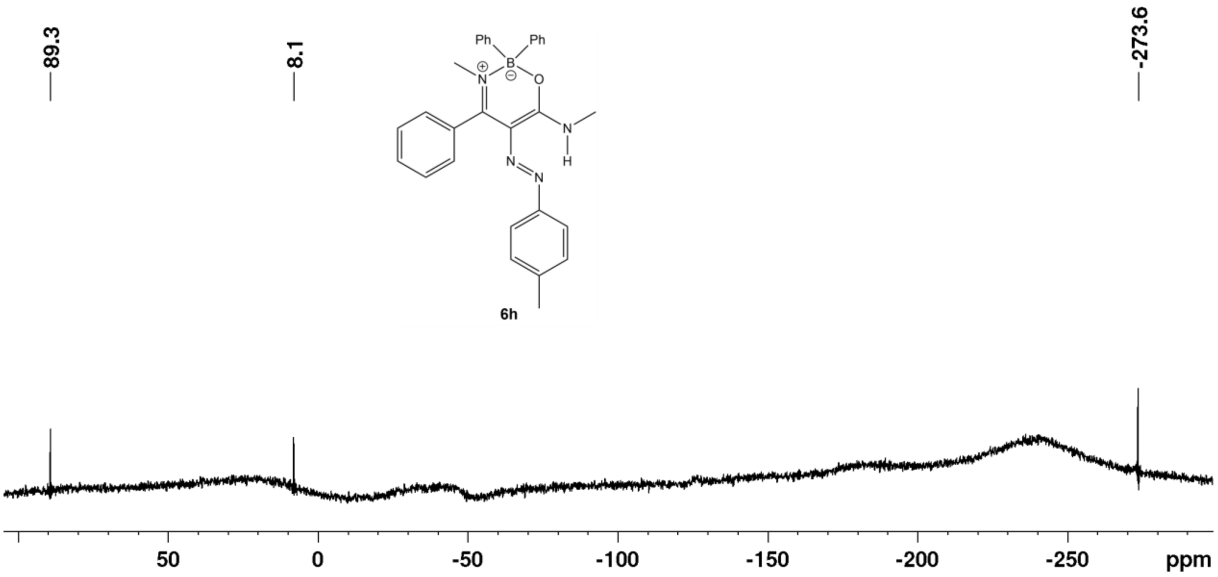

Figure S53. 50 MHz ^{15}N NMR of compound **6h** in $CDCl_3$

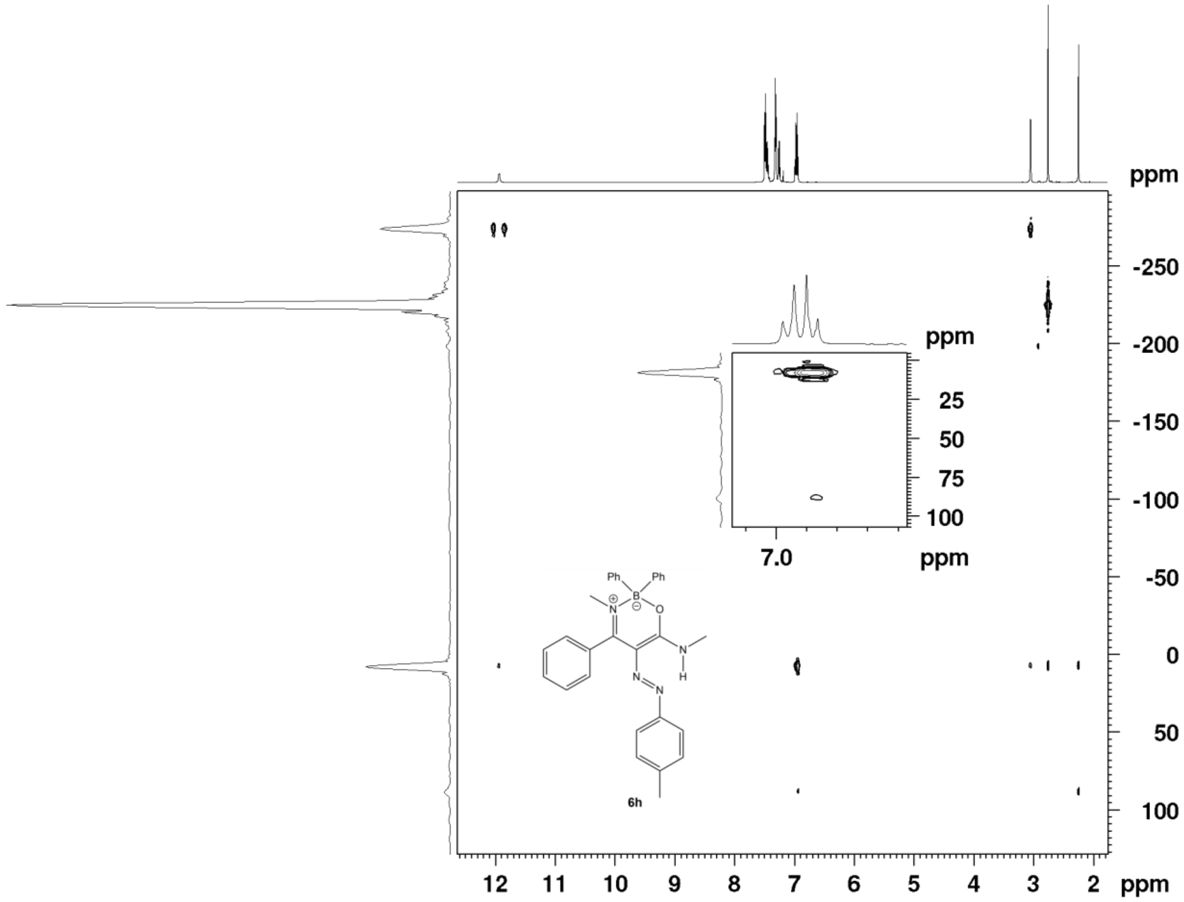

Figure S54. 500 MHz ^1H–^{15}N HMBC NMR of compound **6h** in $CDCl_3$

2.12 Diazaborinone 7a

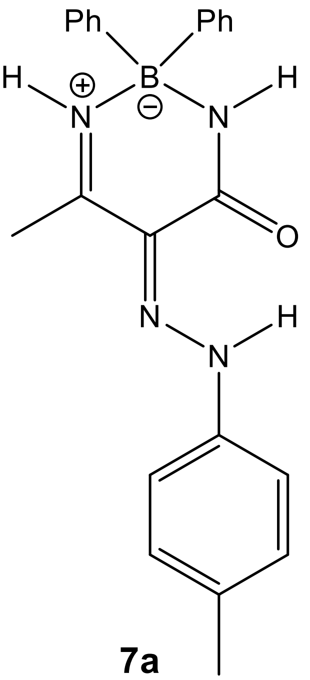

7a

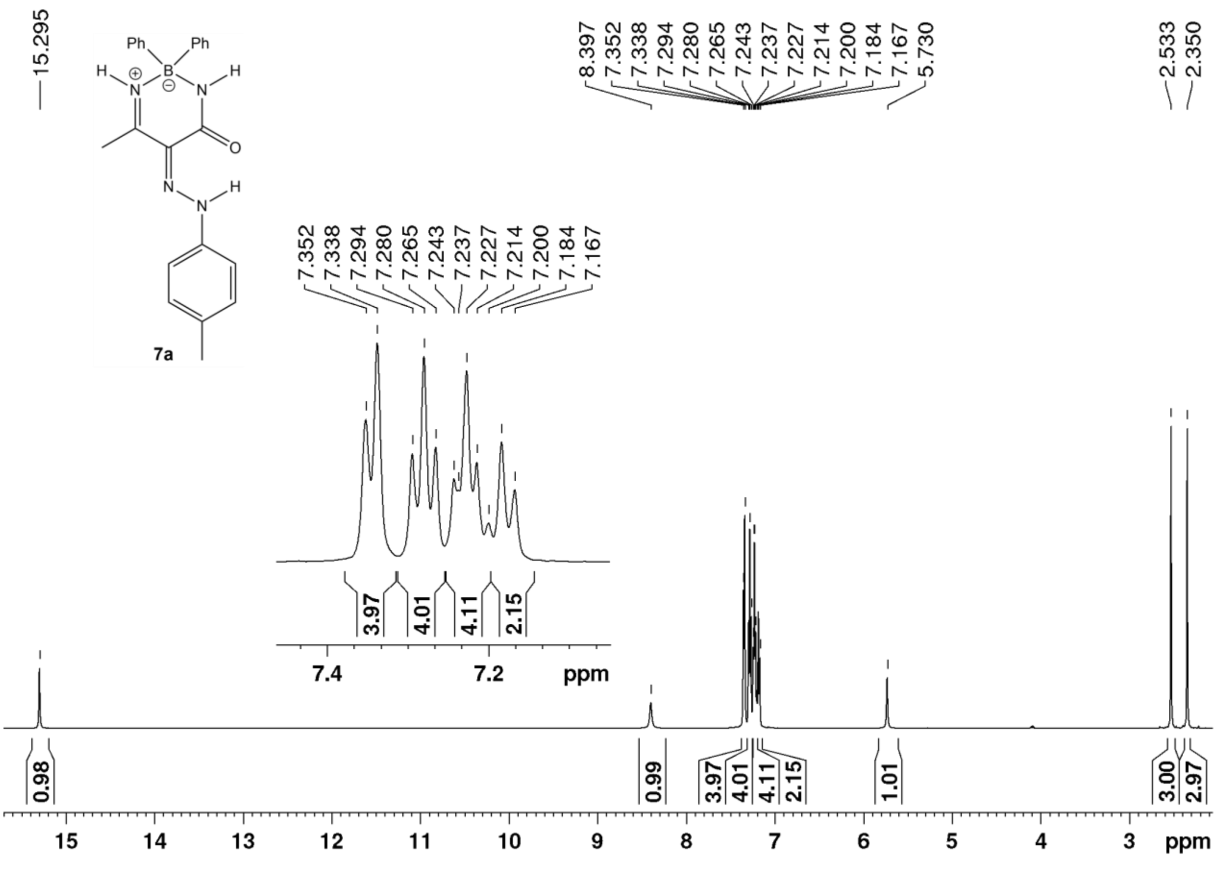

Figure S55. 500 MHz ^{1}H NMR of compound **7a** in $CDCl_3$

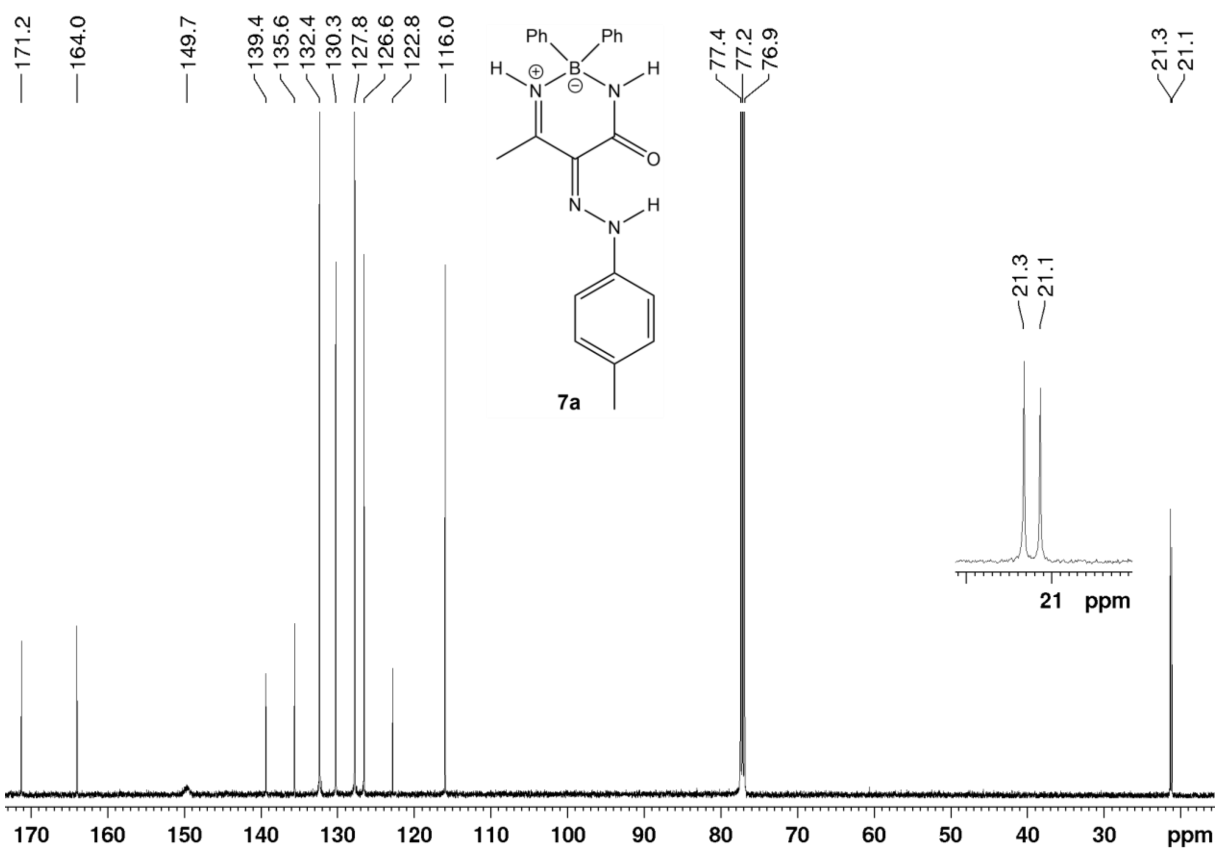

Figure S56. 125 MHz ^{13}C NMR of compound **7a** in $CDCl_3$

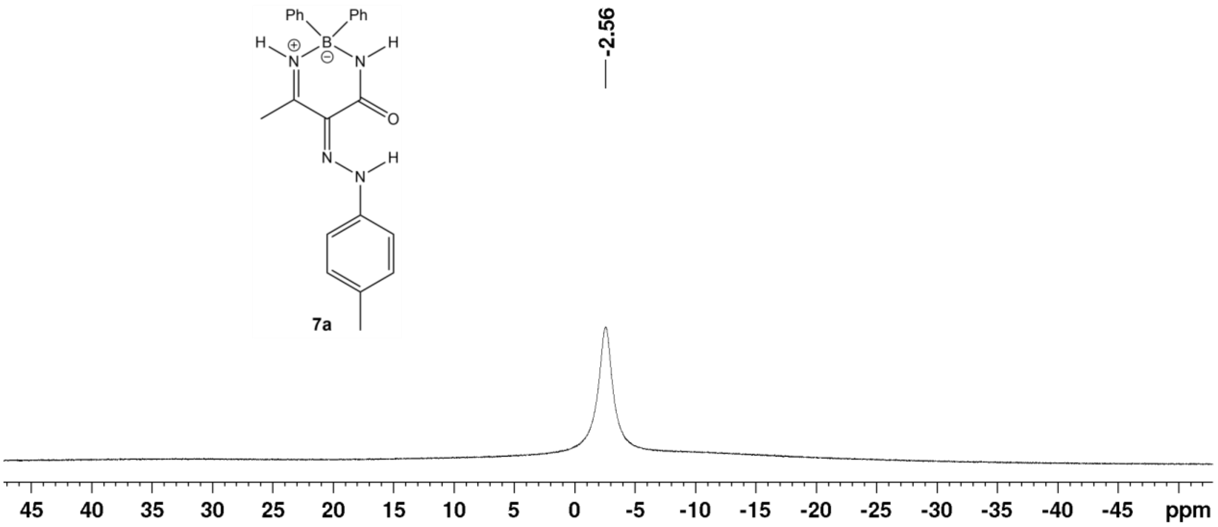

Figure S57. 160 MHz ^{11}B NMR of compound **7a** in $CDCl_3$

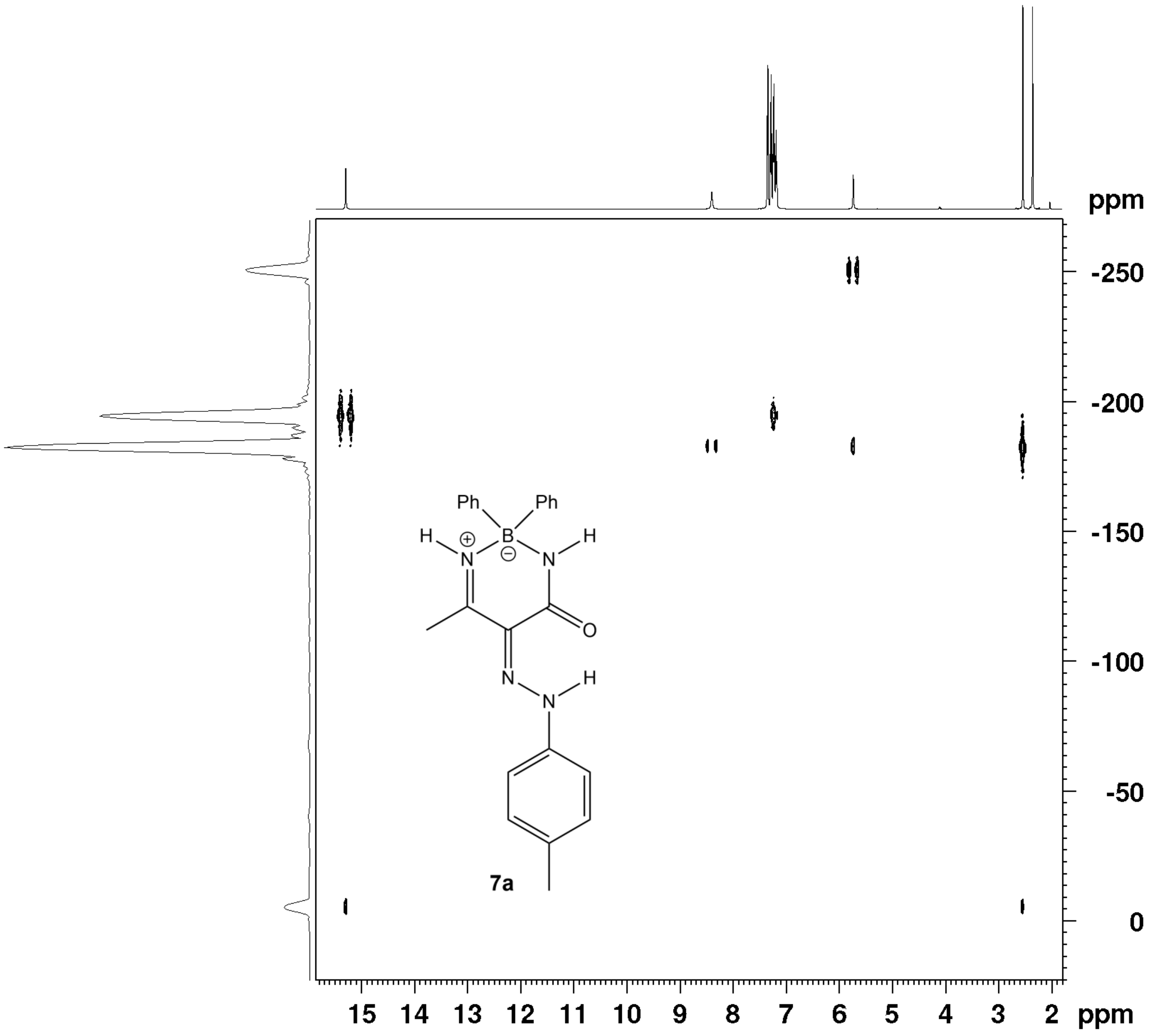

Figure S58. 500 MHz ^{1}H–^{15}N HMBC NMR of compound **7a** in $CDCl_3$

2.13 Diazaborinone 7b

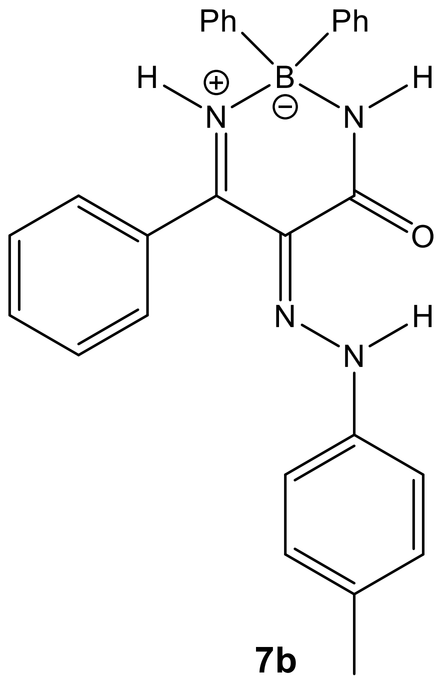

7b

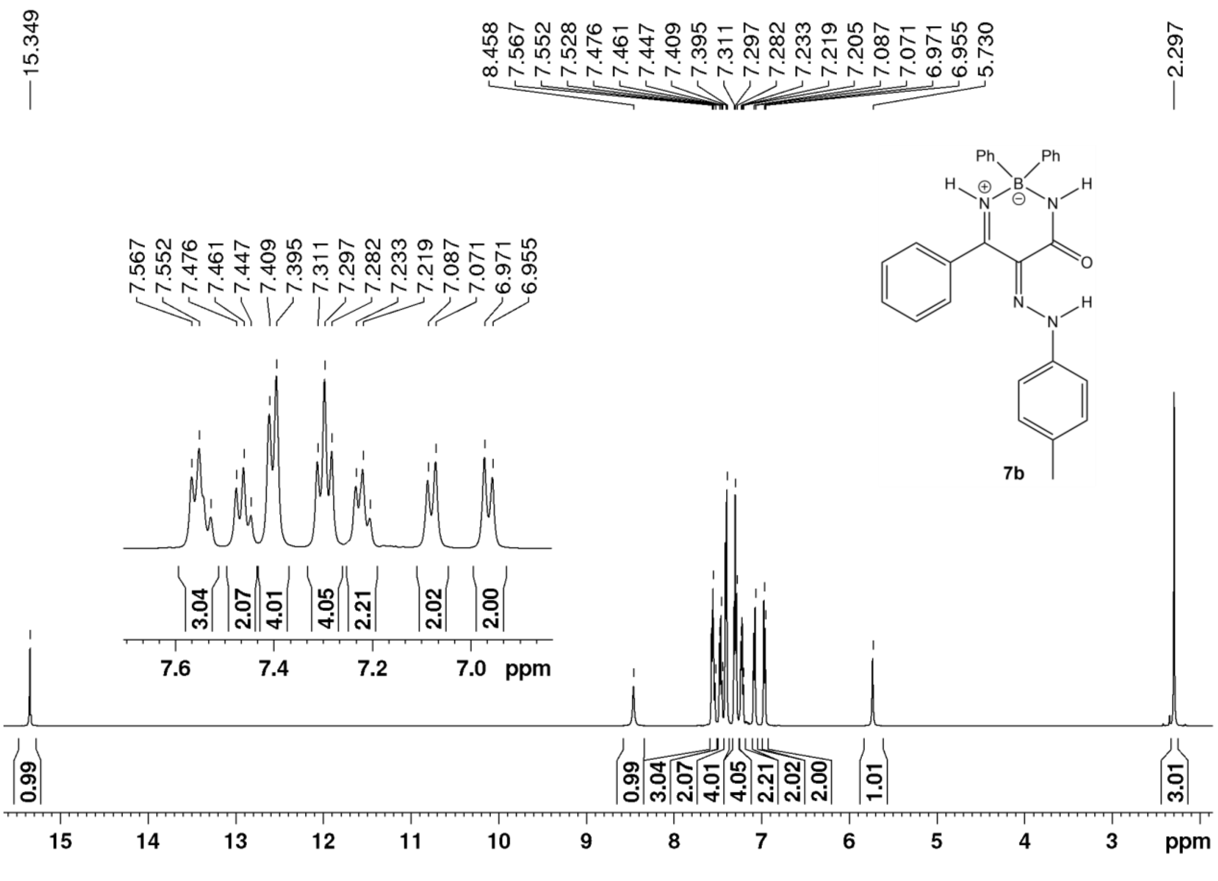

Figure S59. 500 MHz ^{1}H NMR of compound **7b** in $CDCl_3$

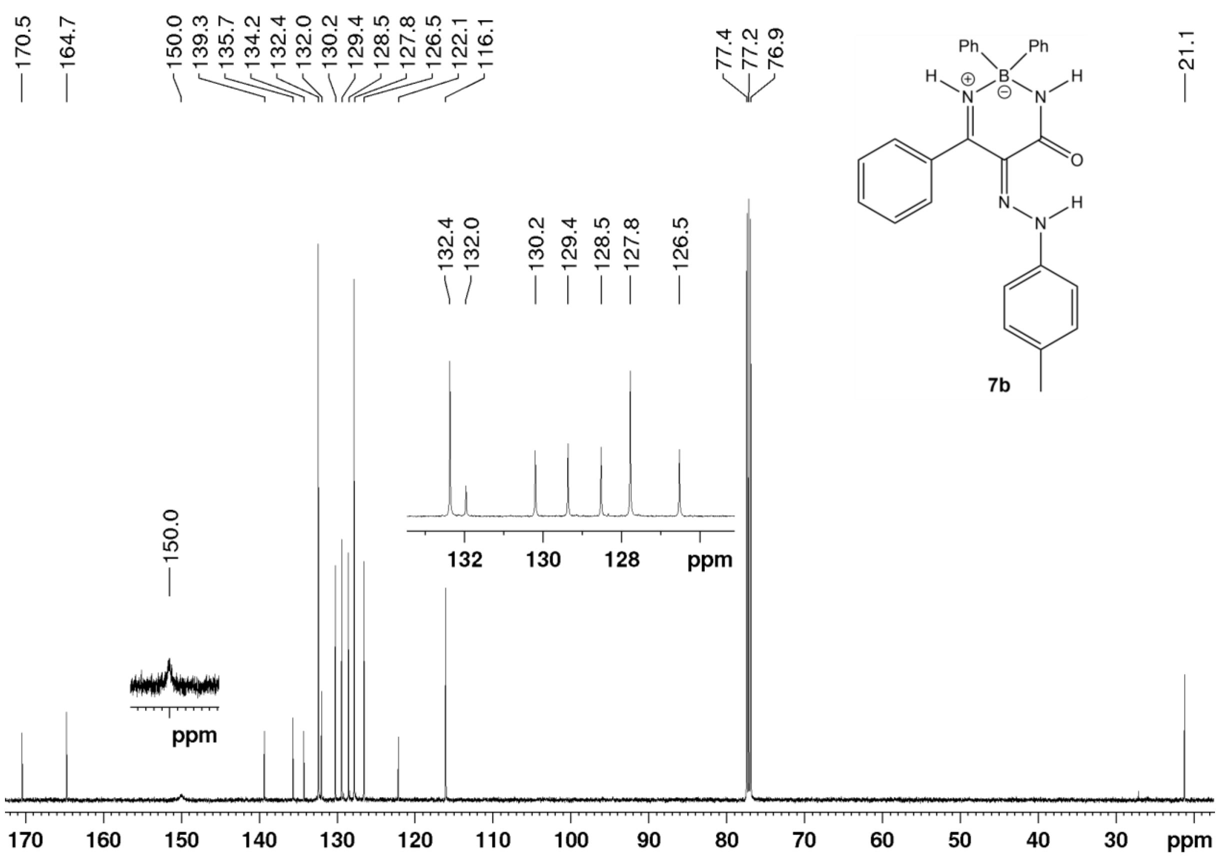

Figure S60. 125 MHz ^{13}C NMR of compound **7b** in $CDCl_3$

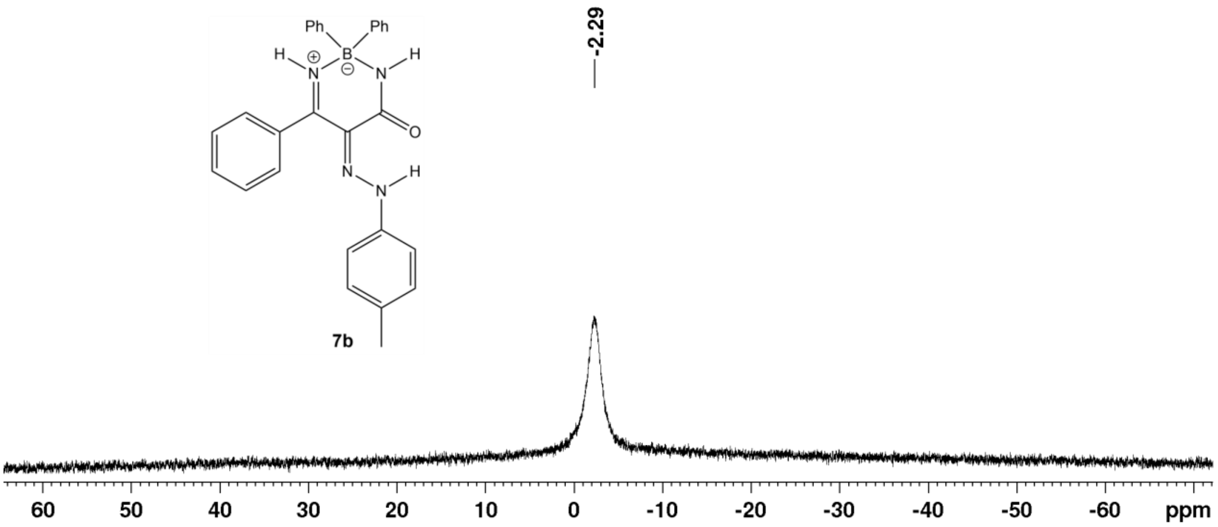

Figure S61. 160 MHz ^{11}B NMR of compound **7b** in $CDCl_3$

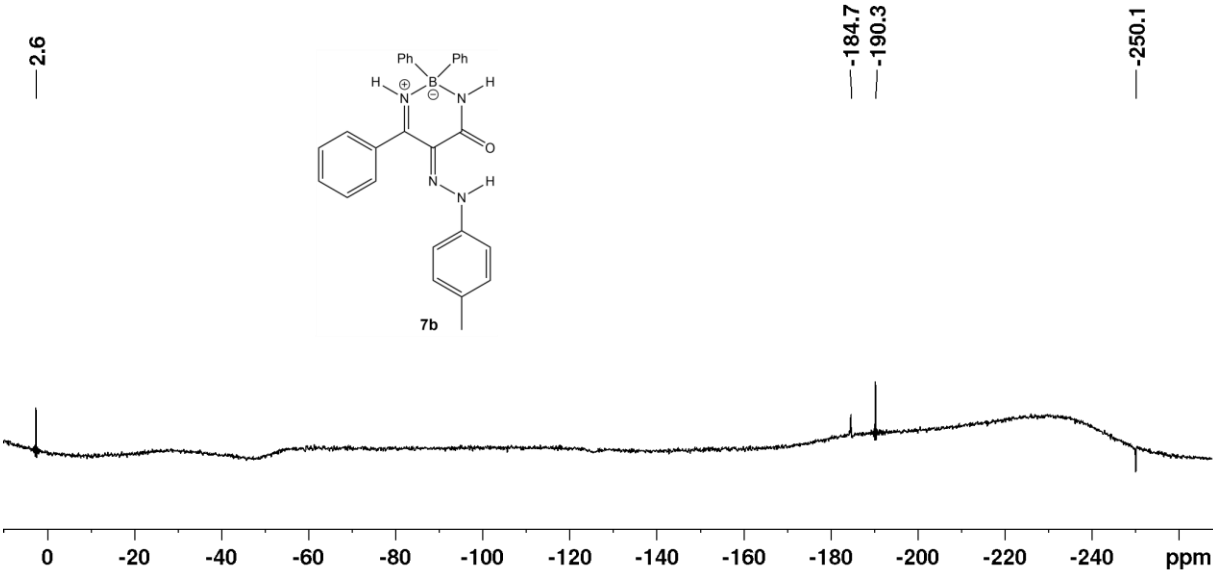

Figure S62. 50 MHz ^{15}N NMR of compound **7b** in $CDCl_3$

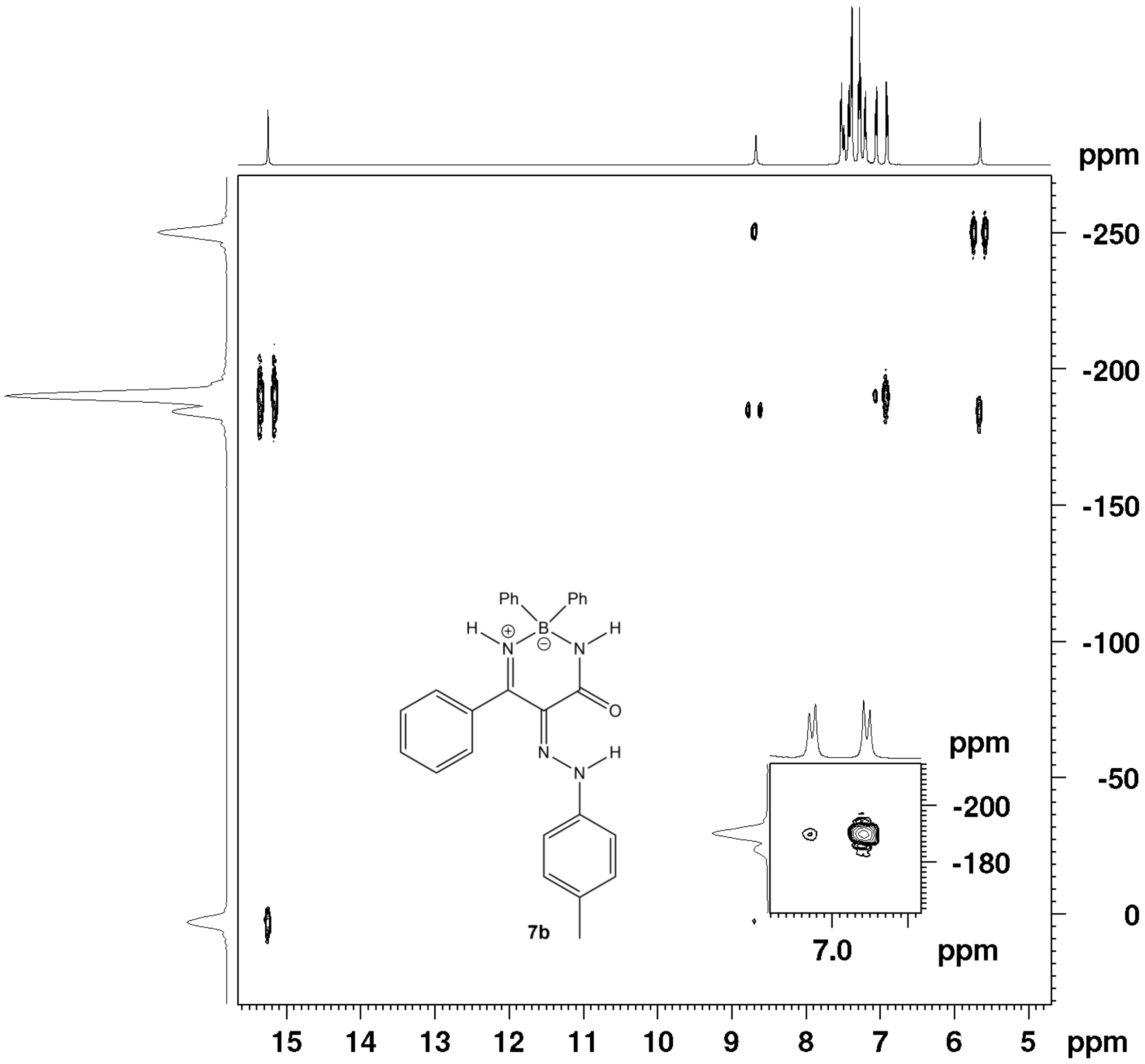

Figure S63. 500 MHz ^{1}H–^{15}N HMBC NMR of compound **7b** in $CDCl_3$

2.14 Diazaborinone 7c

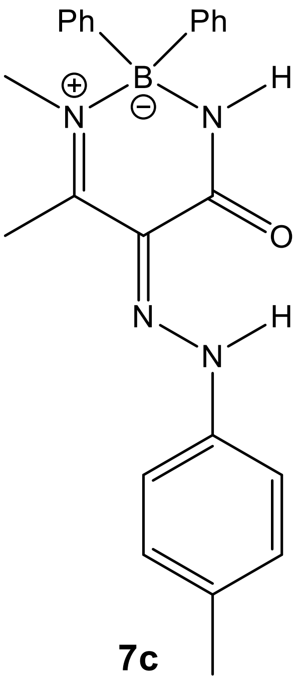

7c

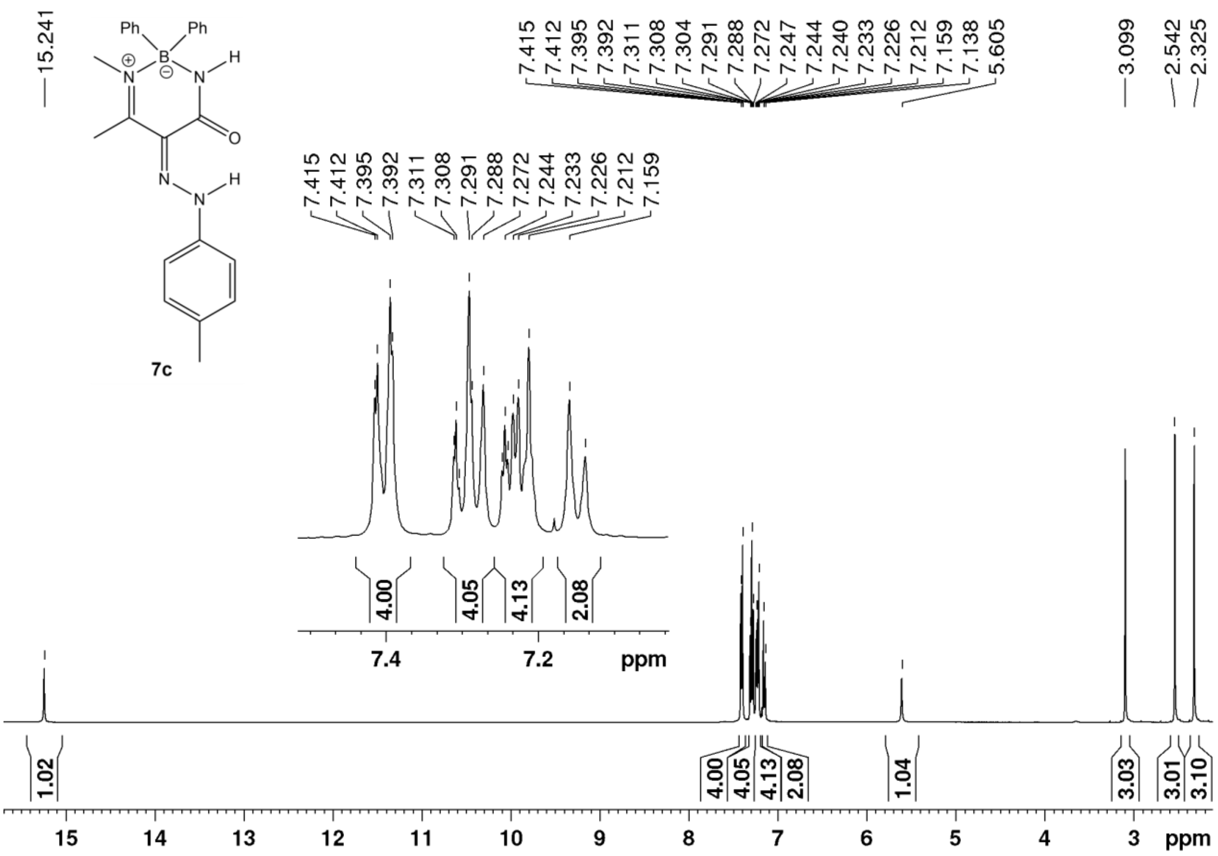

Figure S64. 400 MHz ^{1}H NMR of compound **7c** in $CDCl_3$

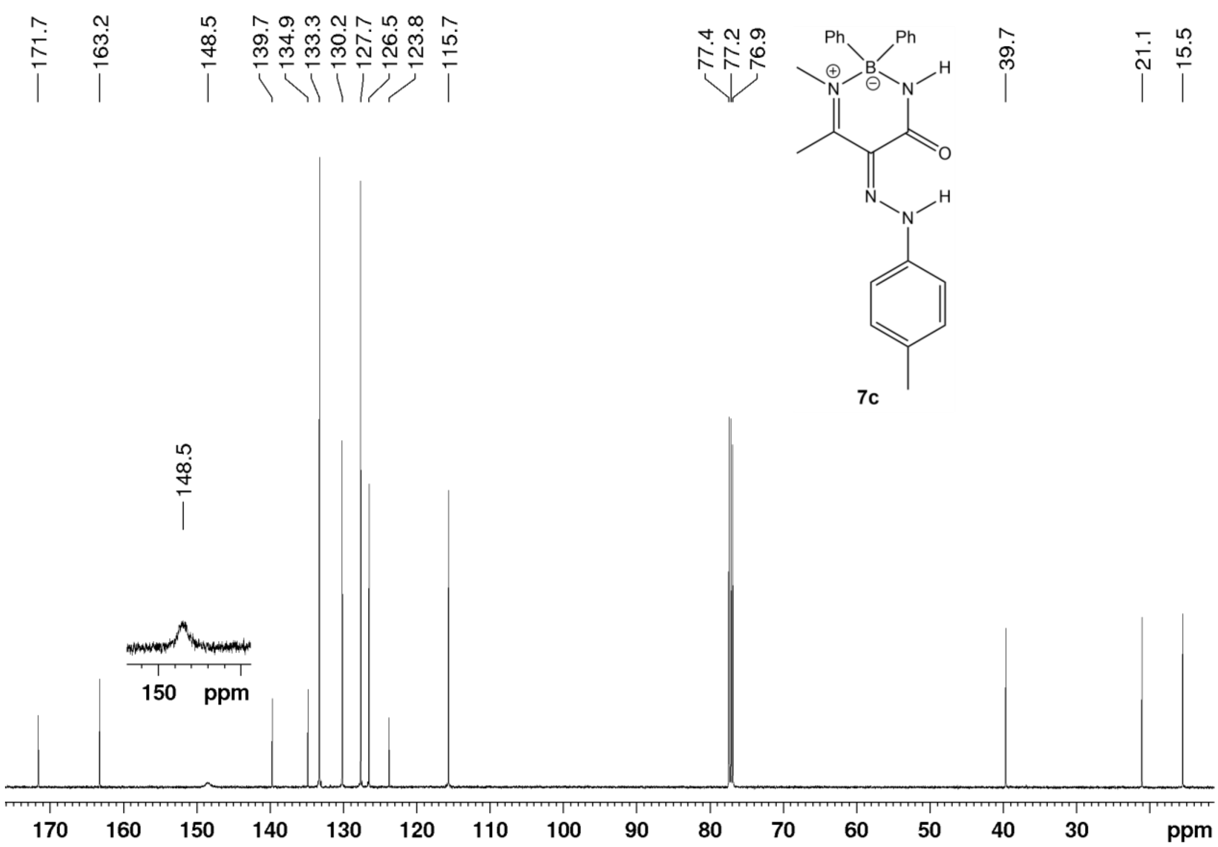

Figure S65. 125 MHz ^{13}C NMR of compound **7c** in $CDCl_3$

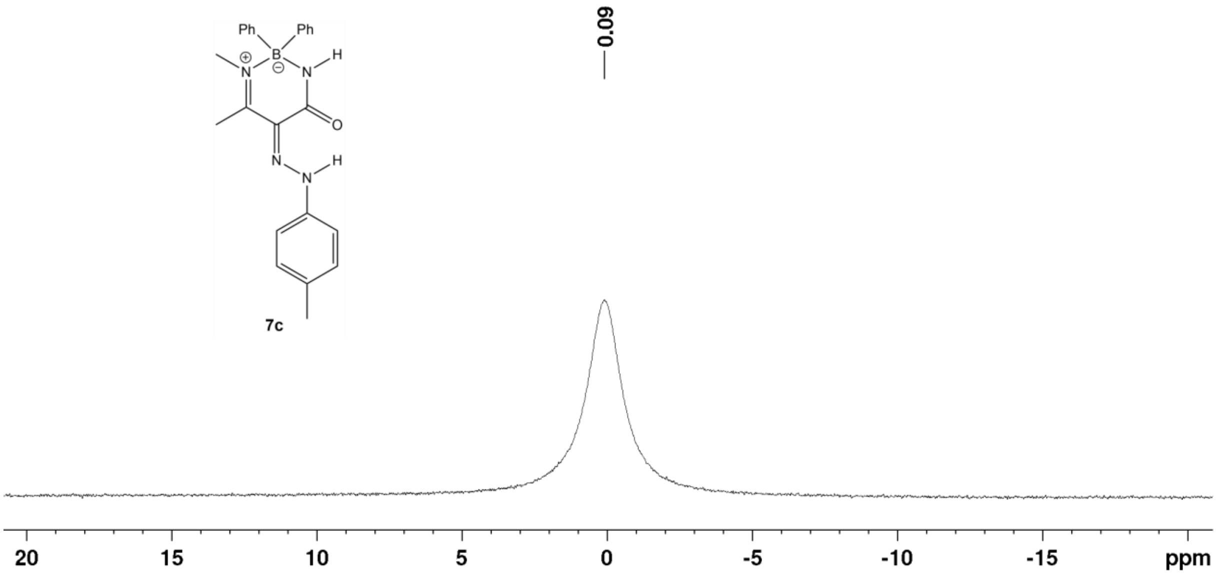

Figure S66. 160 MHz ^{11}B NMR of compound **7c** in $CDCl_3$

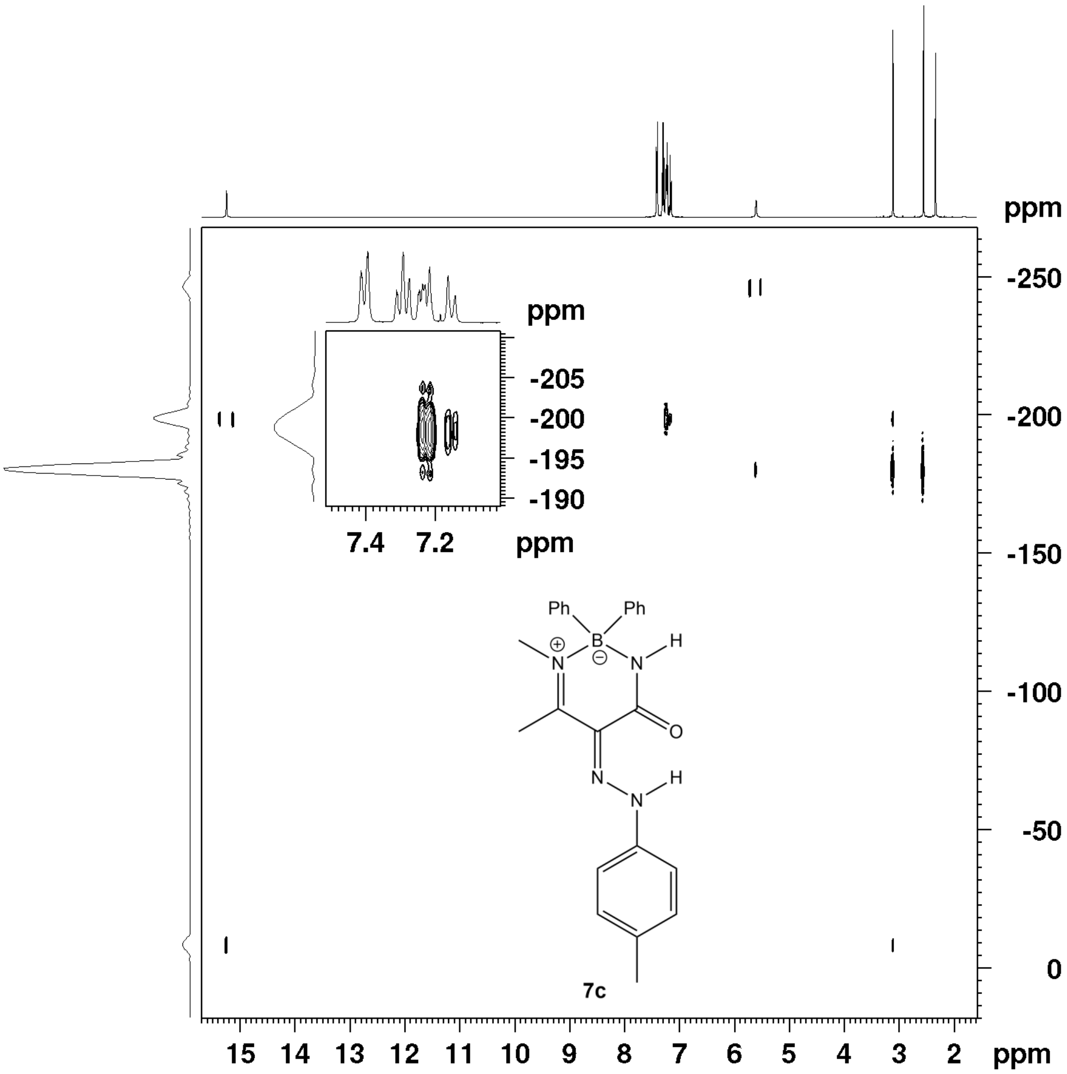

Figure S67. 500 MHz ¹H–¹⁵N HMBC NMR of compound **7c** in CDCl3

2.15 Diazaborinone 7d

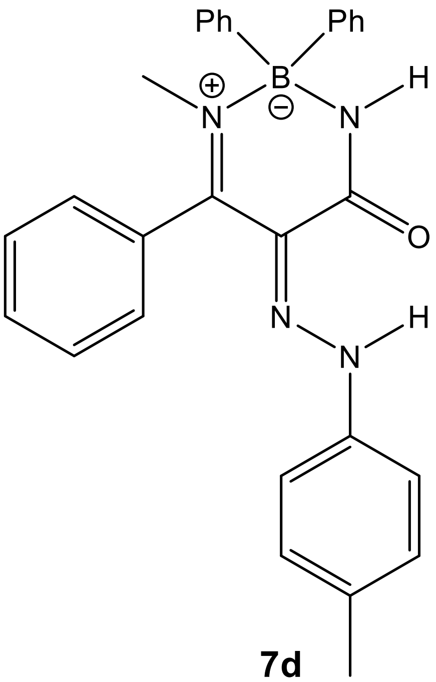

7d

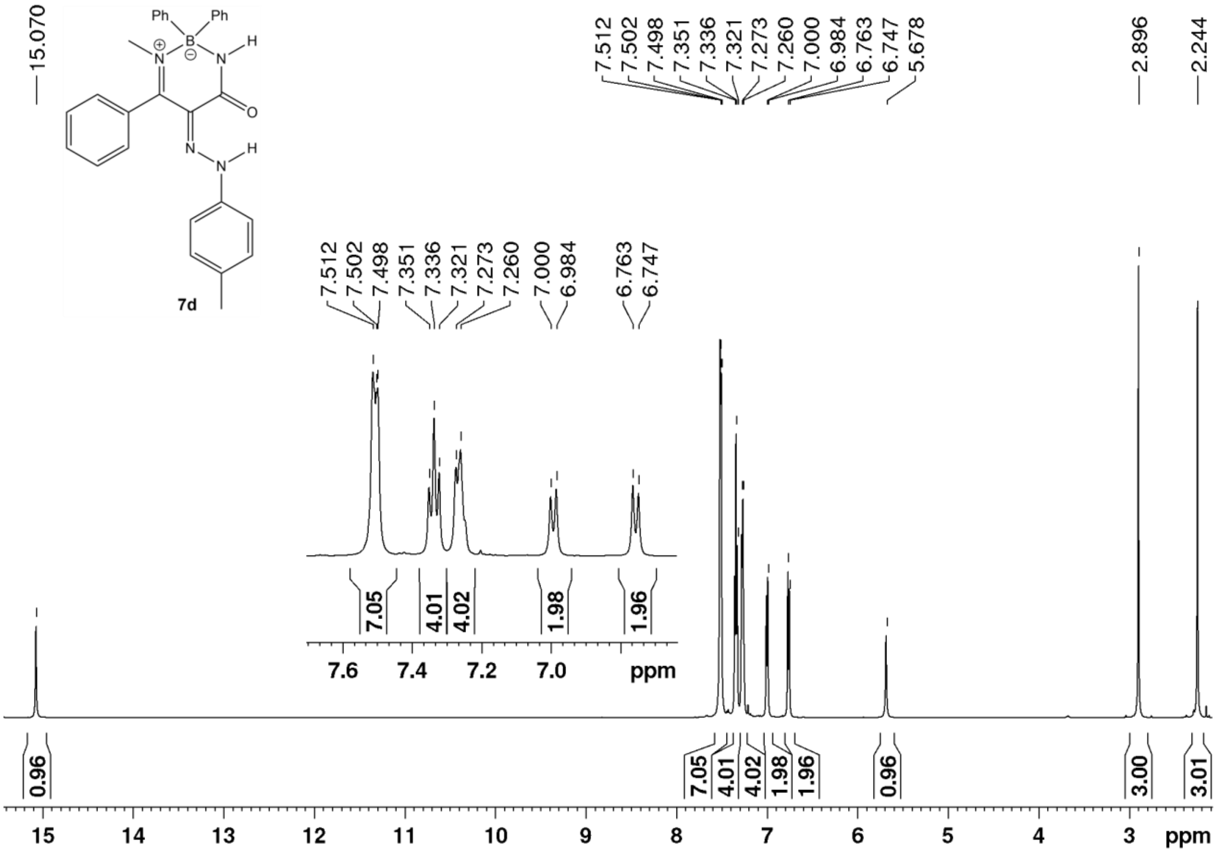

Figure S68. 500 MHz ^{1}H NMR of compound **7d** in $CDCl_3$

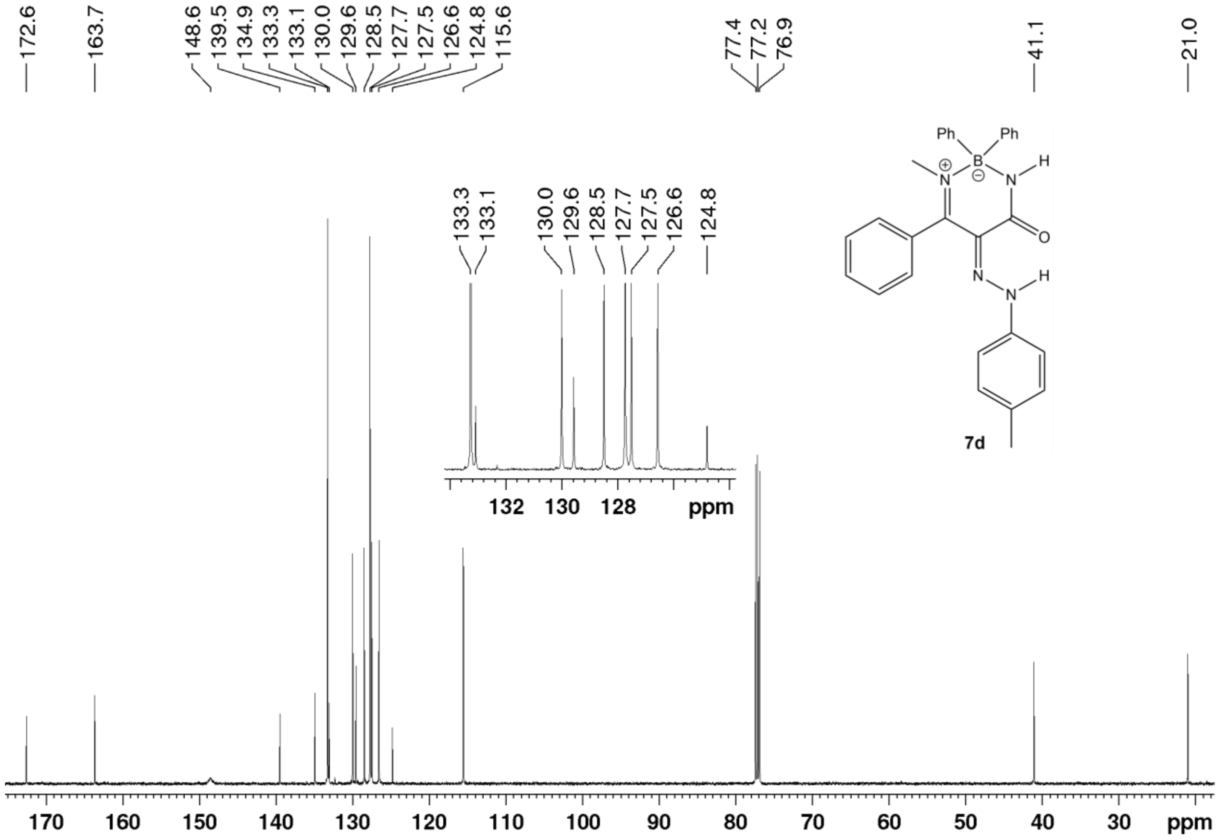

Figure S69. 125 MHz ^{13}C NMR of compound **7d** in $CDCl_3$

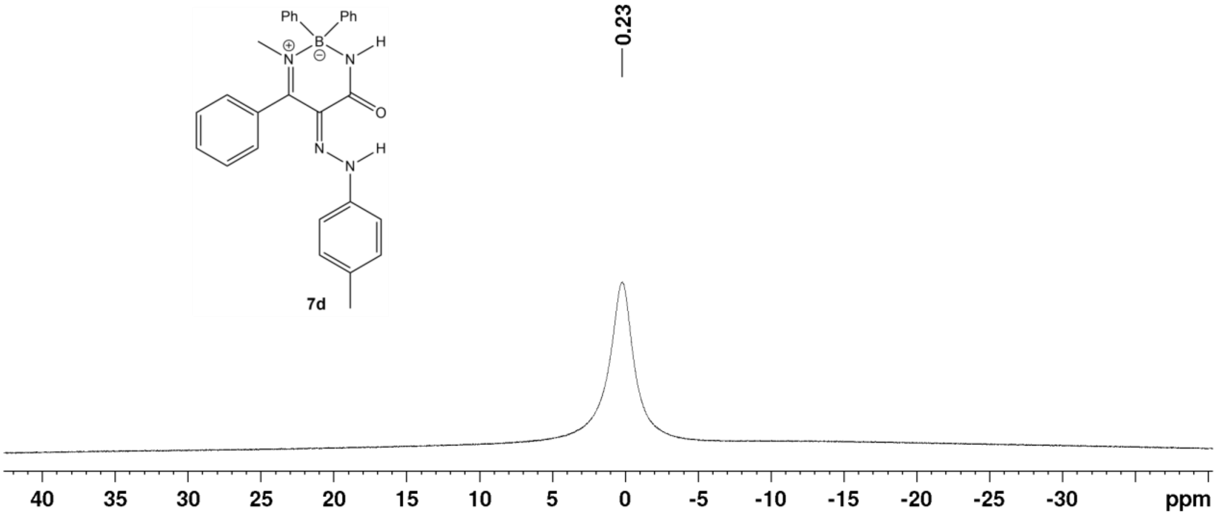

Figure S70. 160 MHz ^{11}B NMR of compound **7d** in $CDCl_3$

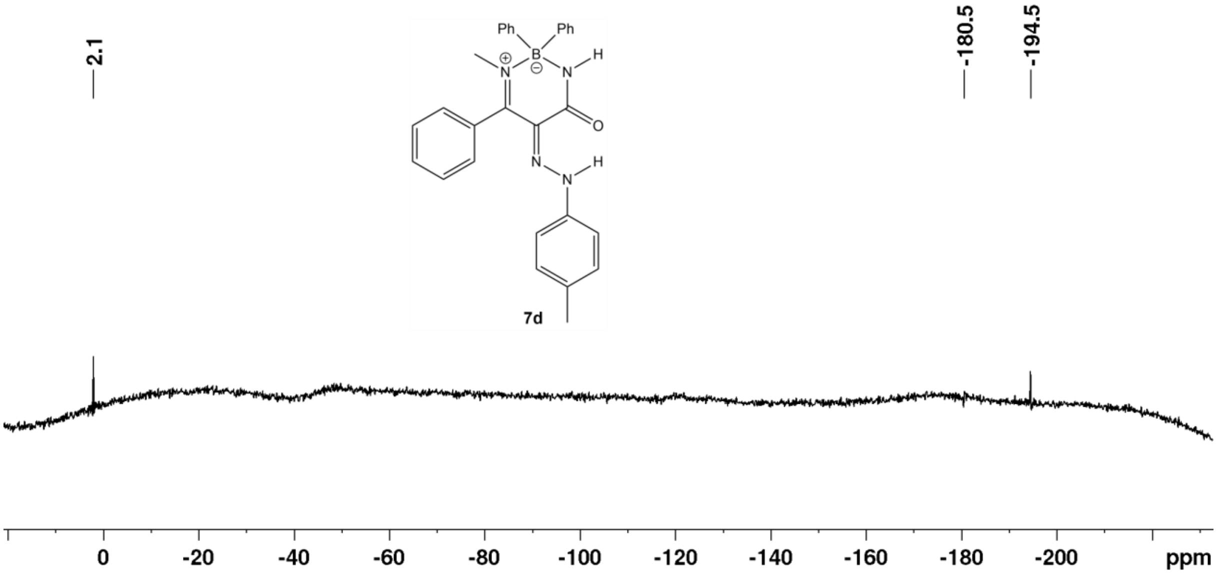

Figure S71. 50 MHz ^{15}N NMR of compound **7d** in $CDCl_3$

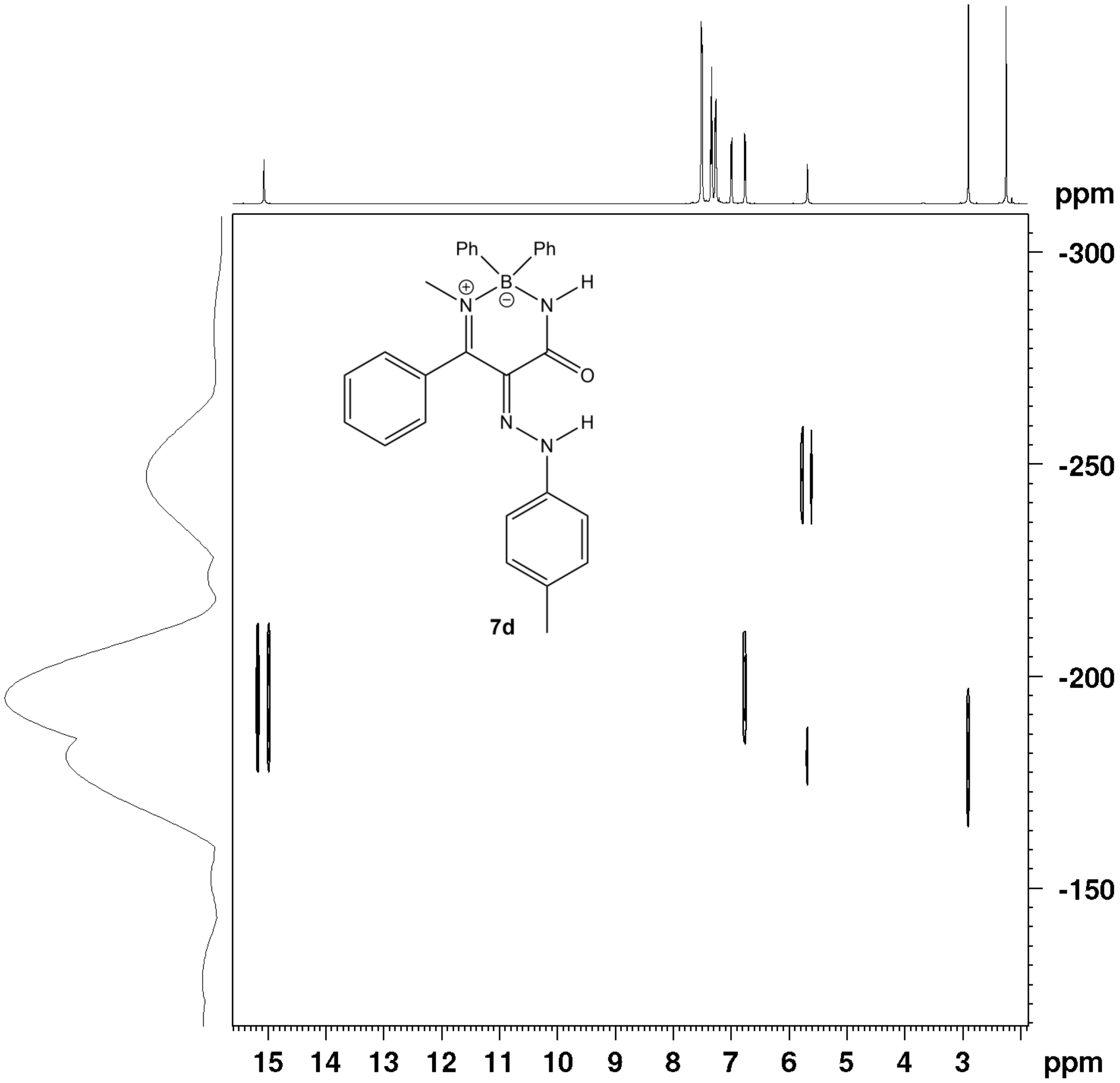

Figure S72. 500 MHz ^{1}H–^{15}N HMBC NMR of compound **7d** in $CDCl_3$

2.16 Diazaborinone 7e

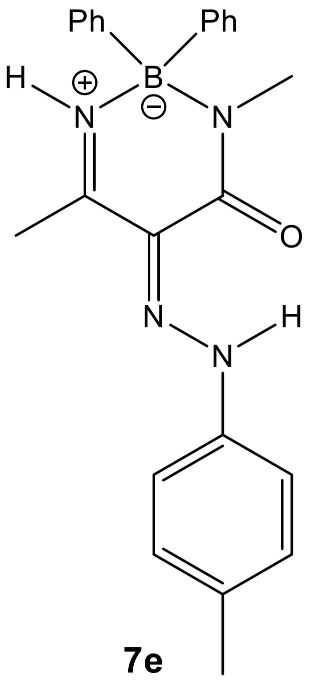

7e

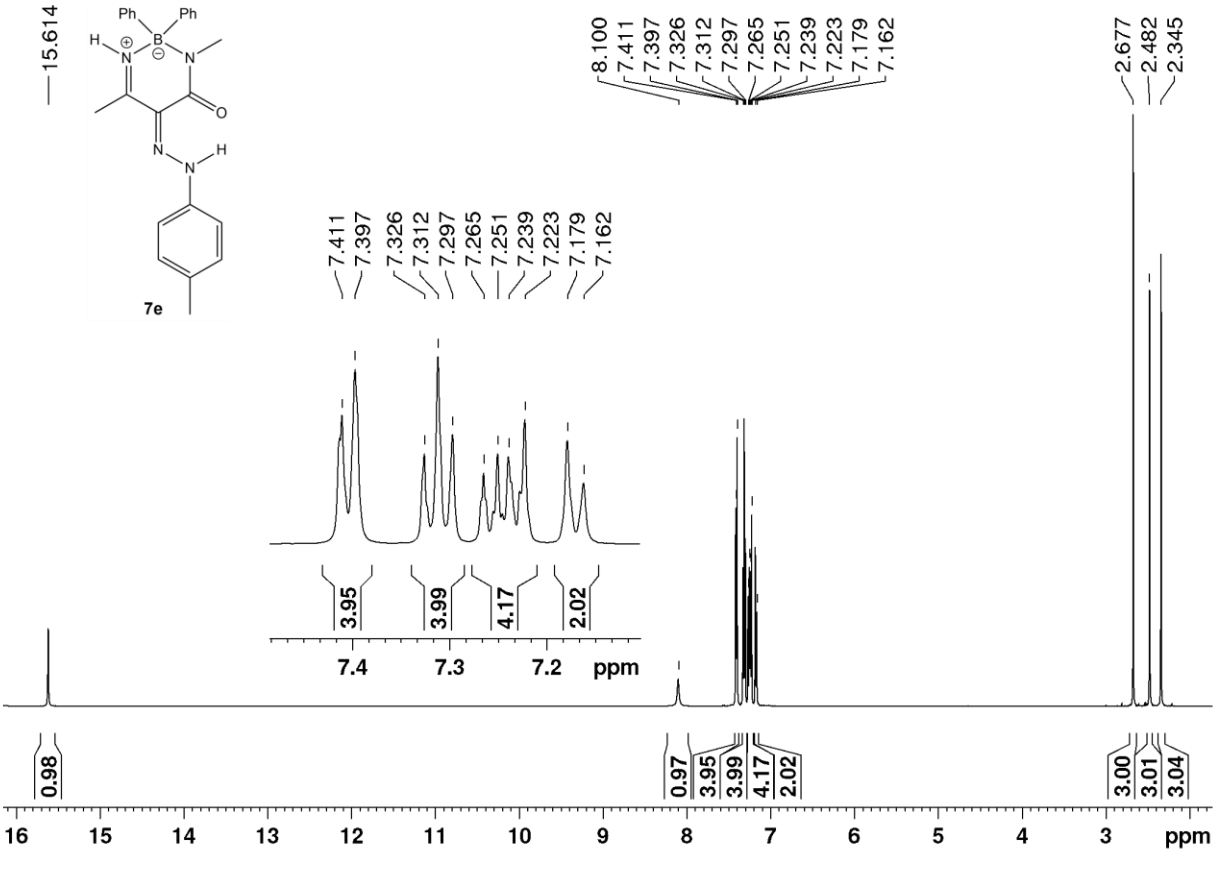

Figure S73. 500 MHz ^{1}H NMR of compound **7e** in $CDCl_3$

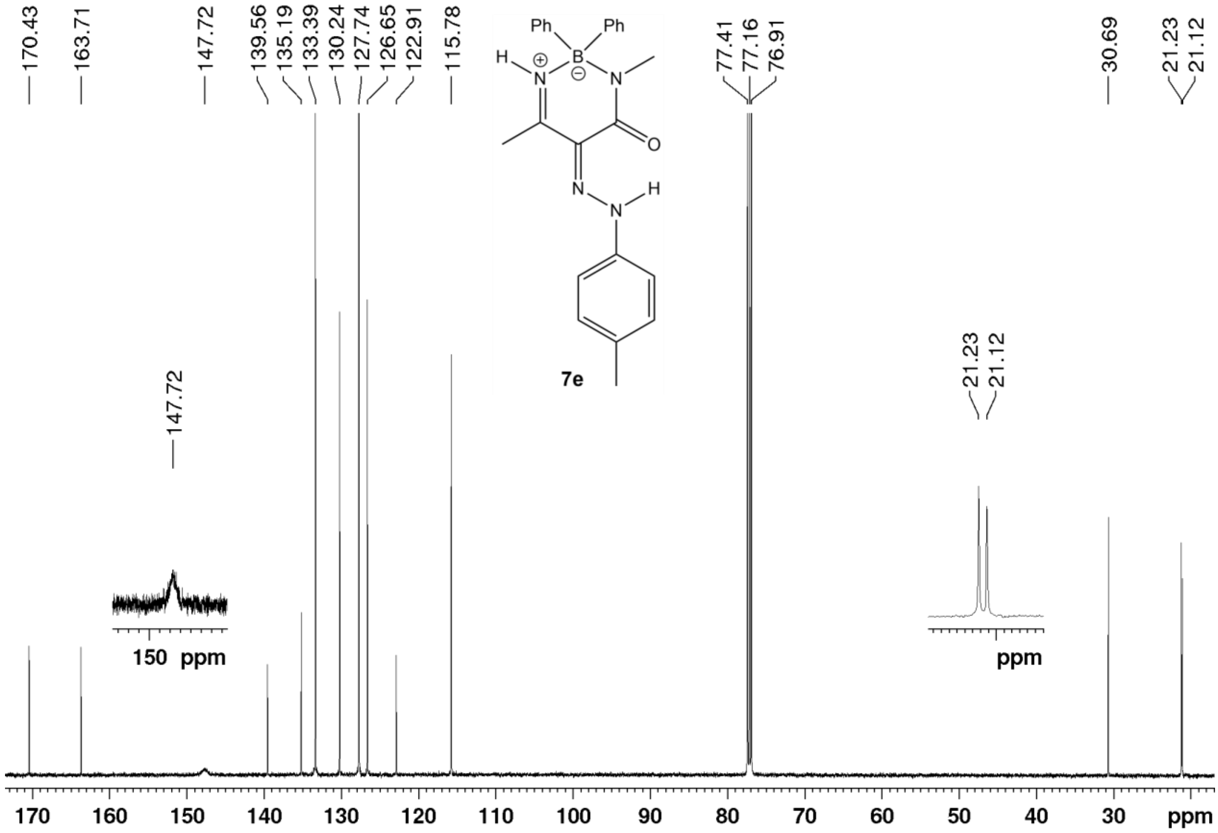

Figure S74. 125 MHz ^{13}C NMR of compound **7e** in $CDCl_3$

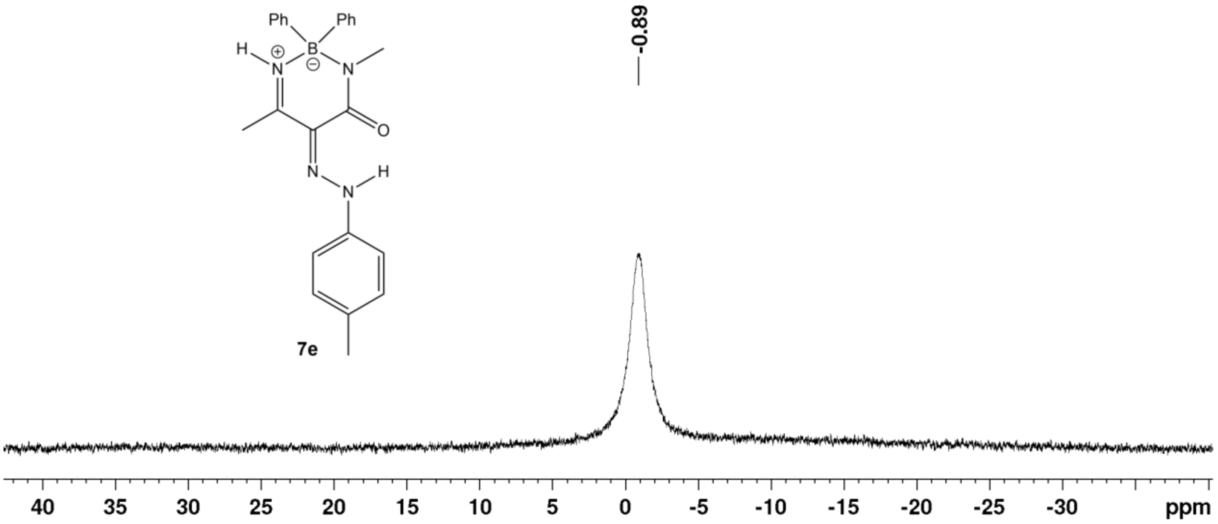

Figure S75. 160 MHz ^{11}B NMR of compound **7e** in $CDCl_3$

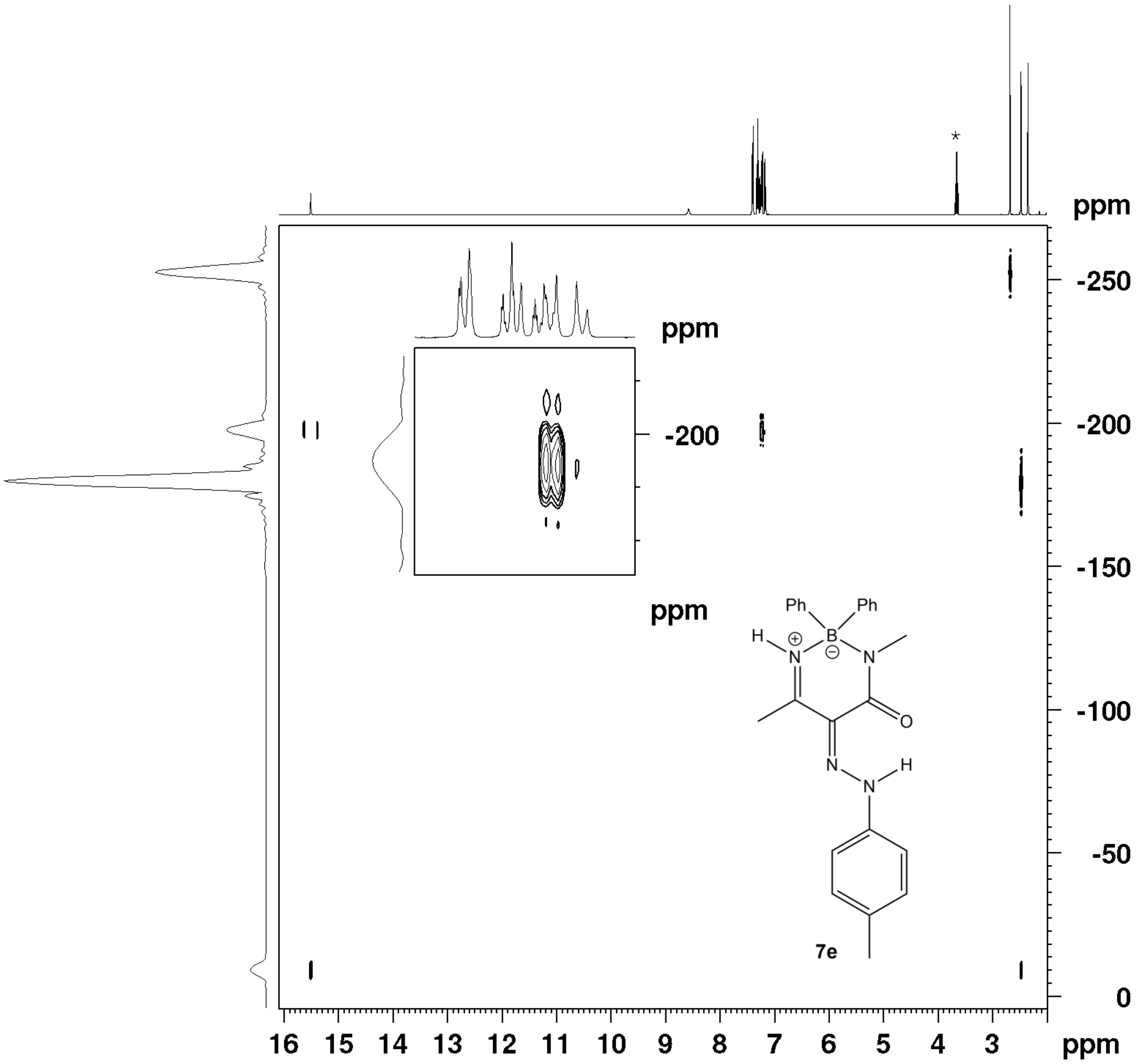

Figure S76. 400 MHz ^1H–^{15}N HMBC NMR of compound **7e** in $CDCl_3$ (*CH_2 from ethanol)

2.17 Diazaborinone 7f

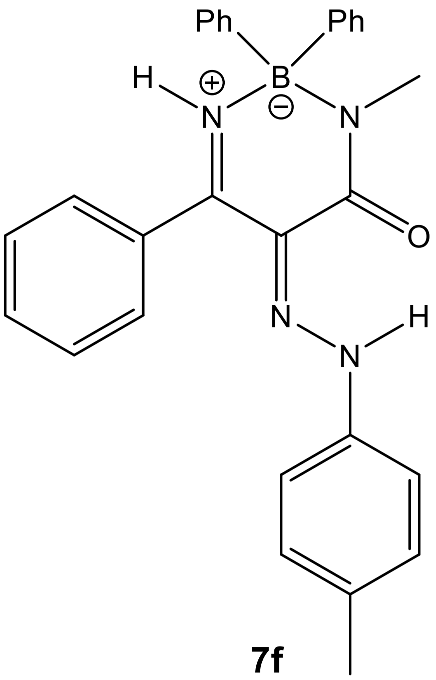

7f

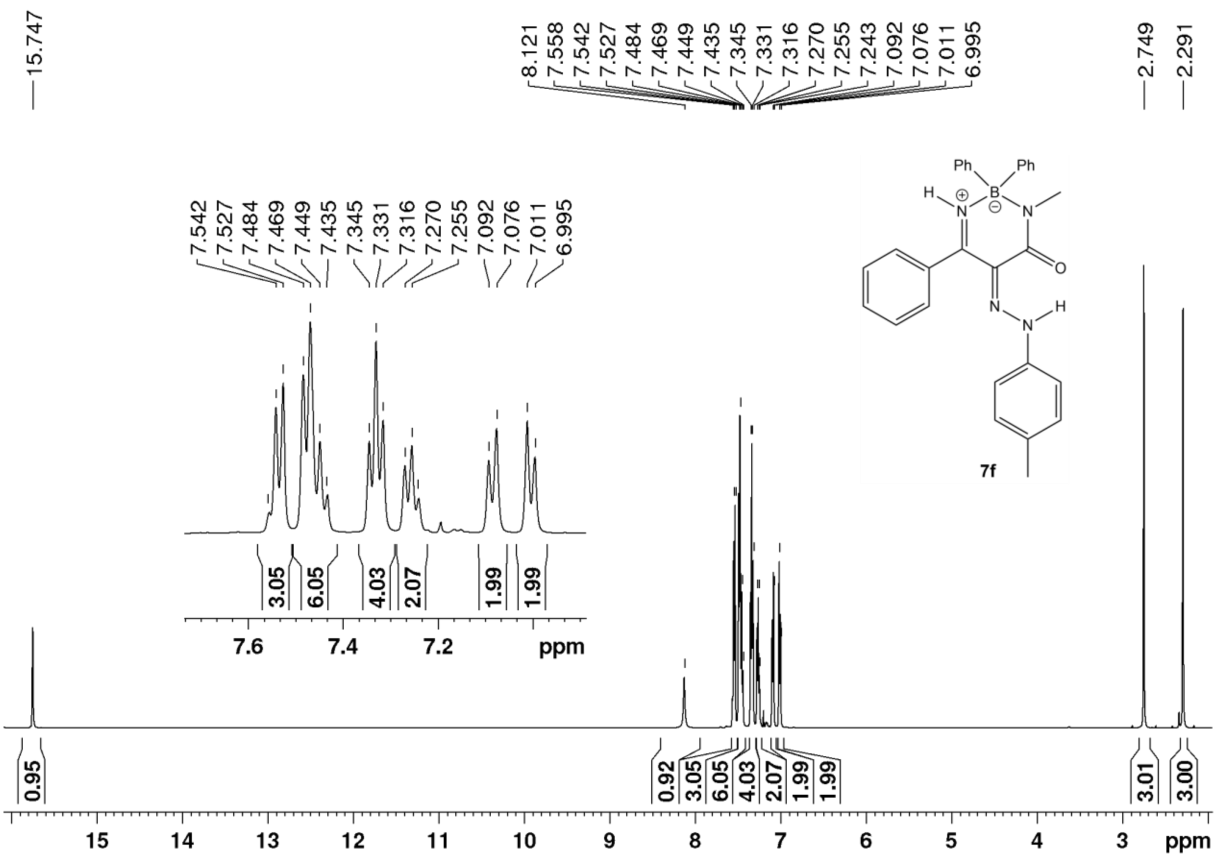

Figure S77. 500 MHz ^{1}H NMR of compound **7f** in $CDCl_3$

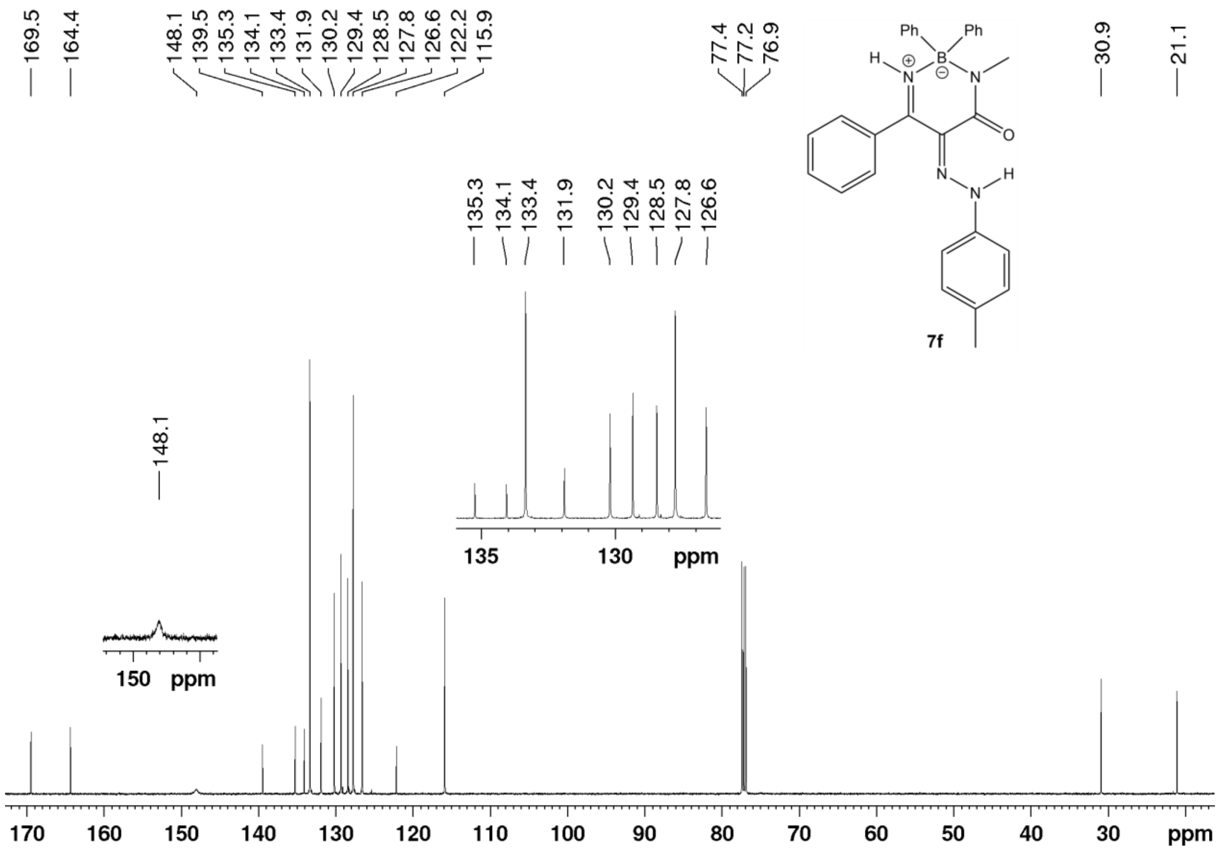

Figure S78. 125 MHz ^{13}C NMR of compound **7f** in $CDCl_3$

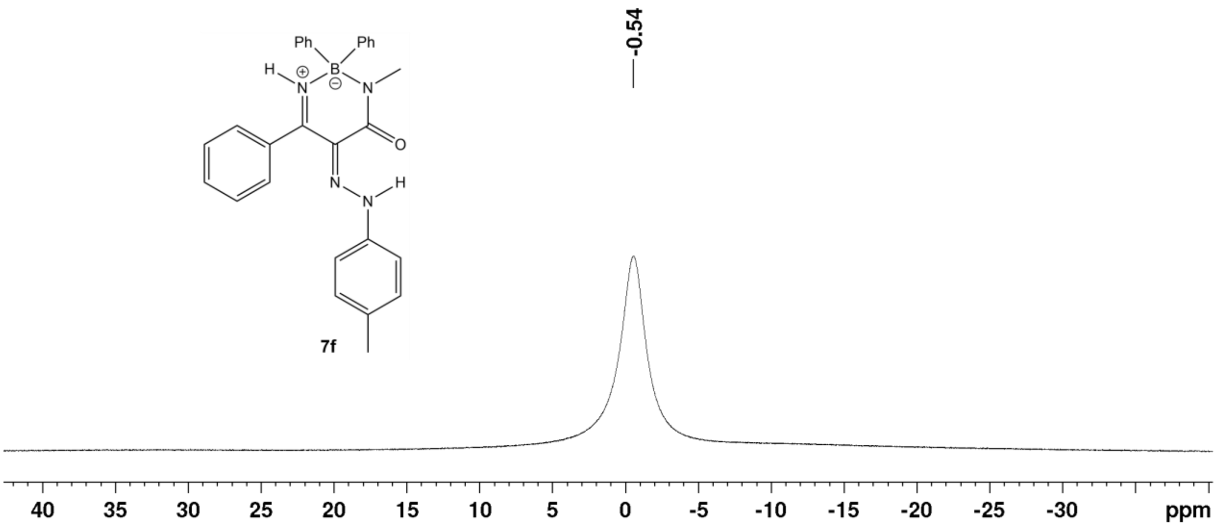

Figure S79. 160 MHz ^{11}B NMR of compound **7f** in $CDCl_3$

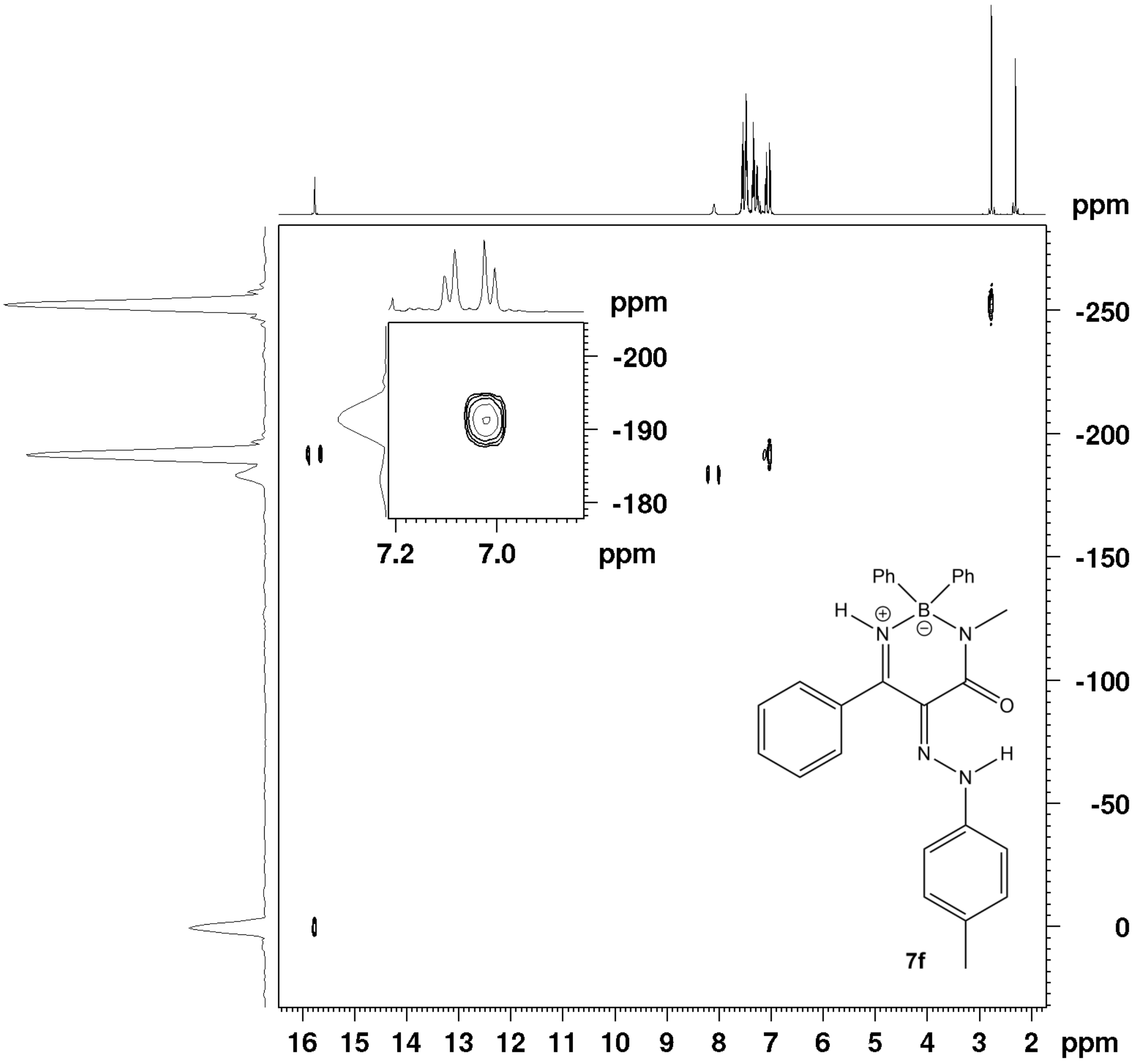

Figure S80. 400 MHz ^{1}H–^{15}N HMBC NMR of compound **7f** in $CDCl_3$

2.18 Diazaborinone 7g

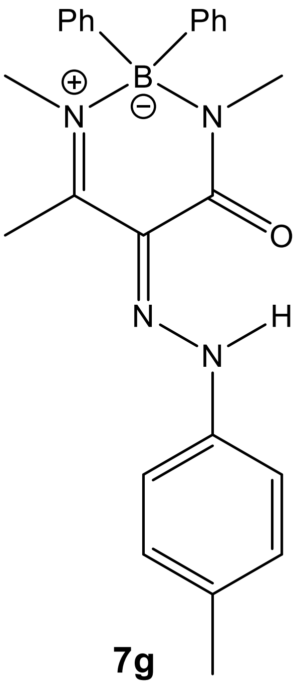

7g

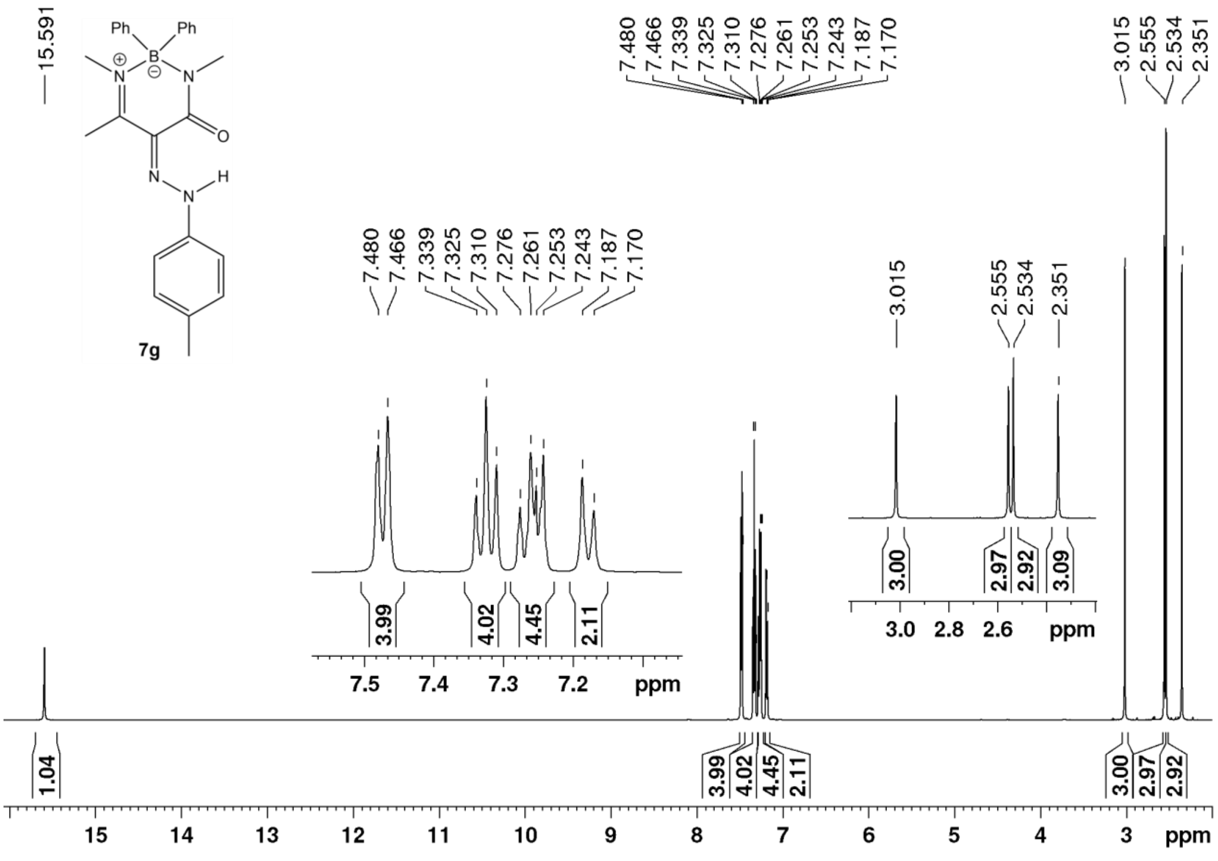

Figure S81. 500 MHz ^{1}H NMR of compound **7g** in $CDCl_3$

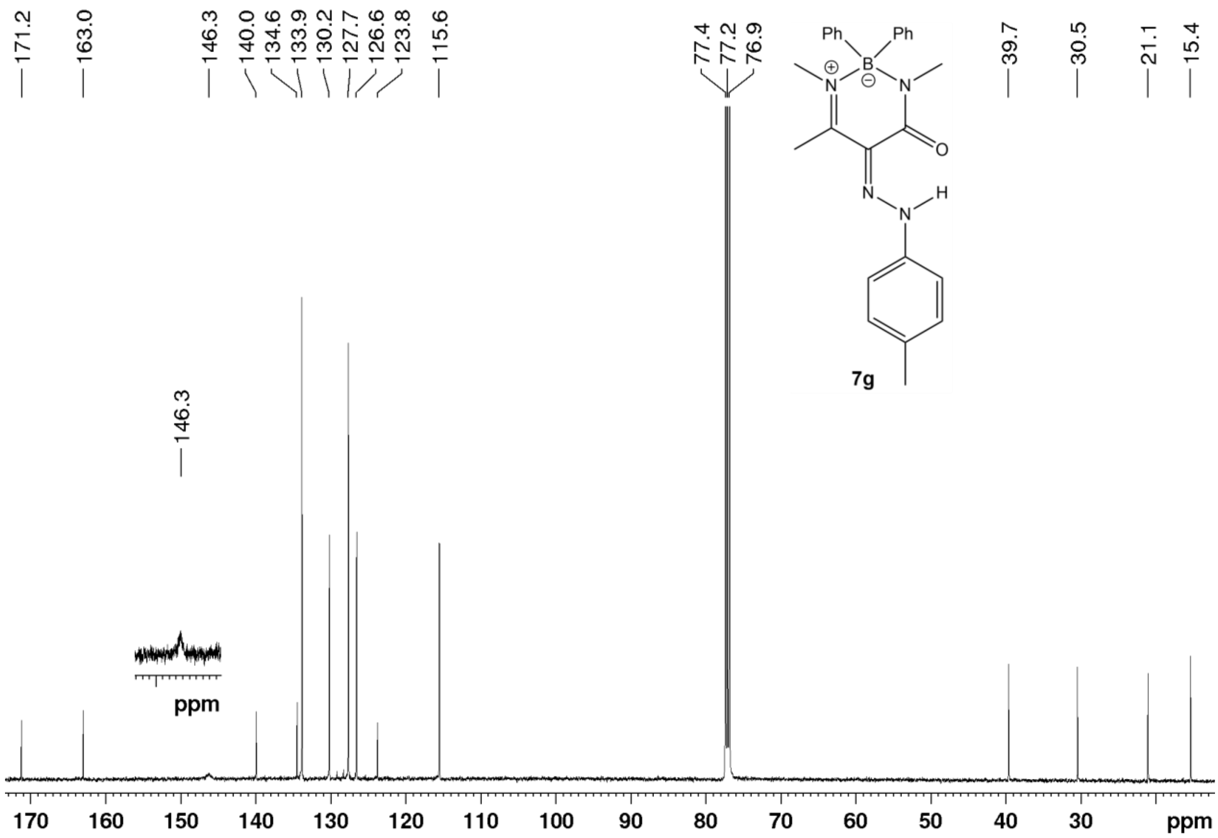

Figure S82. 125 MHz ^{13}C NMR of compound **7g** in $CDCl_3$

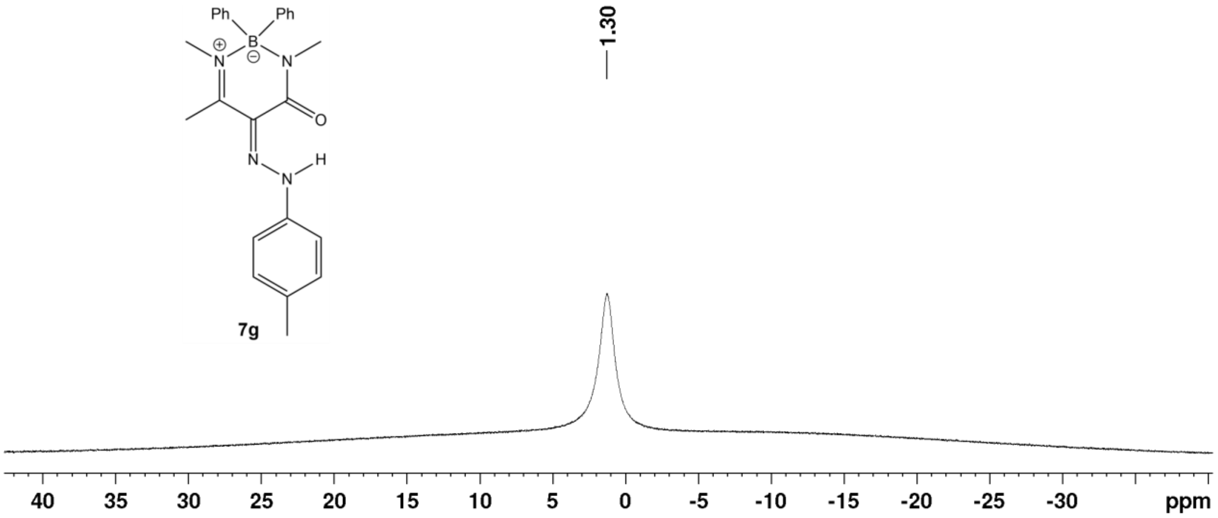

Figure S83. 160 MHz ^{11}B NMR of compound **7g** in $CDCl_3$

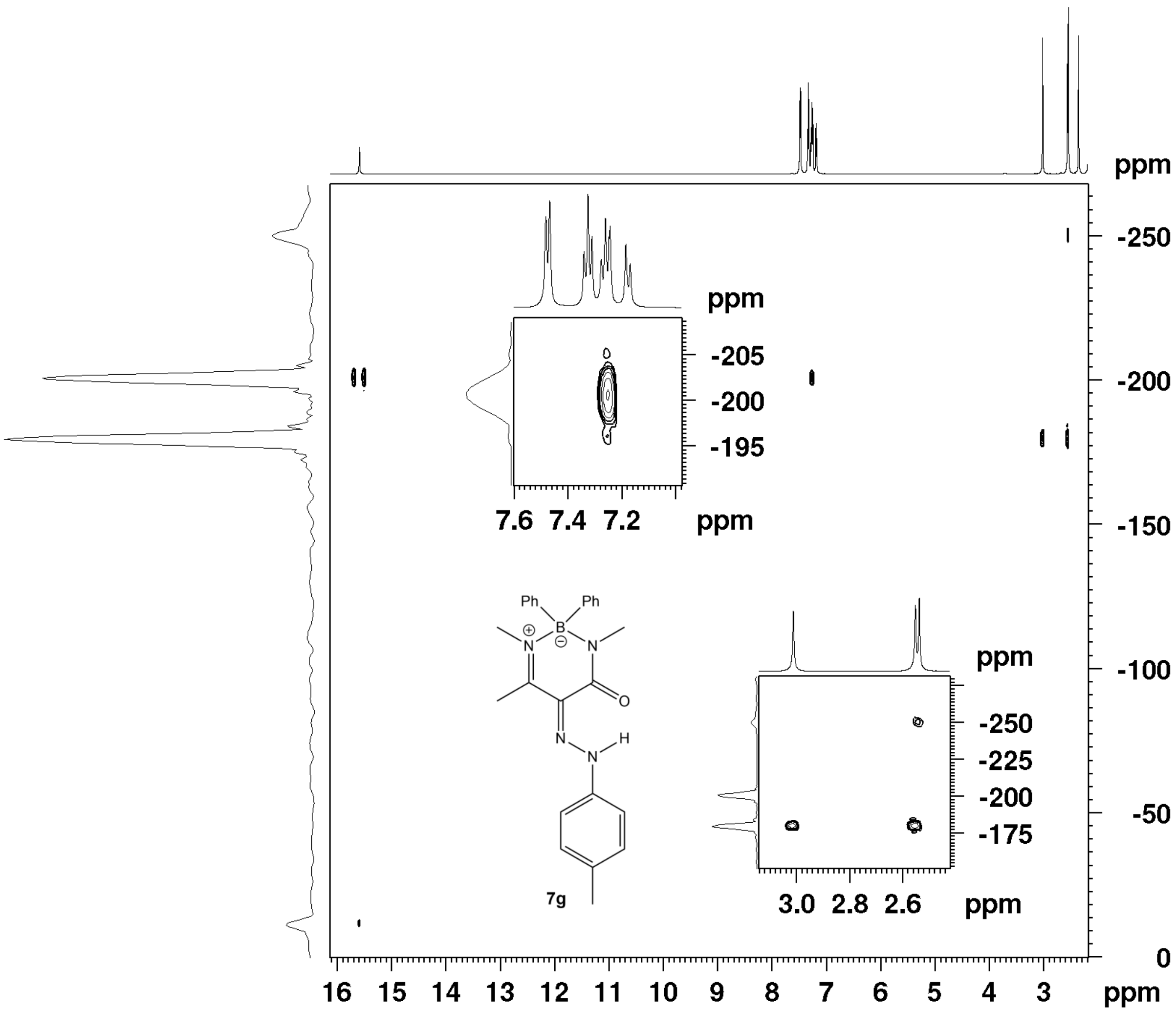

Figure S84. 500 MHz ¹H–¹⁵N HMBC NMR of compound **7g** in $CDCl_3$

2.19 Diazaborinone 7h

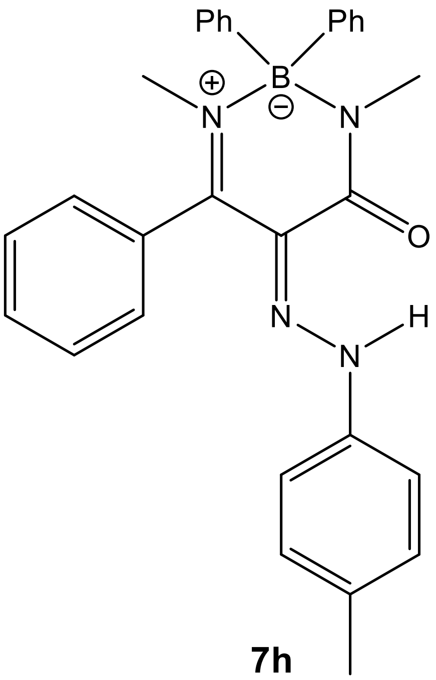

7h

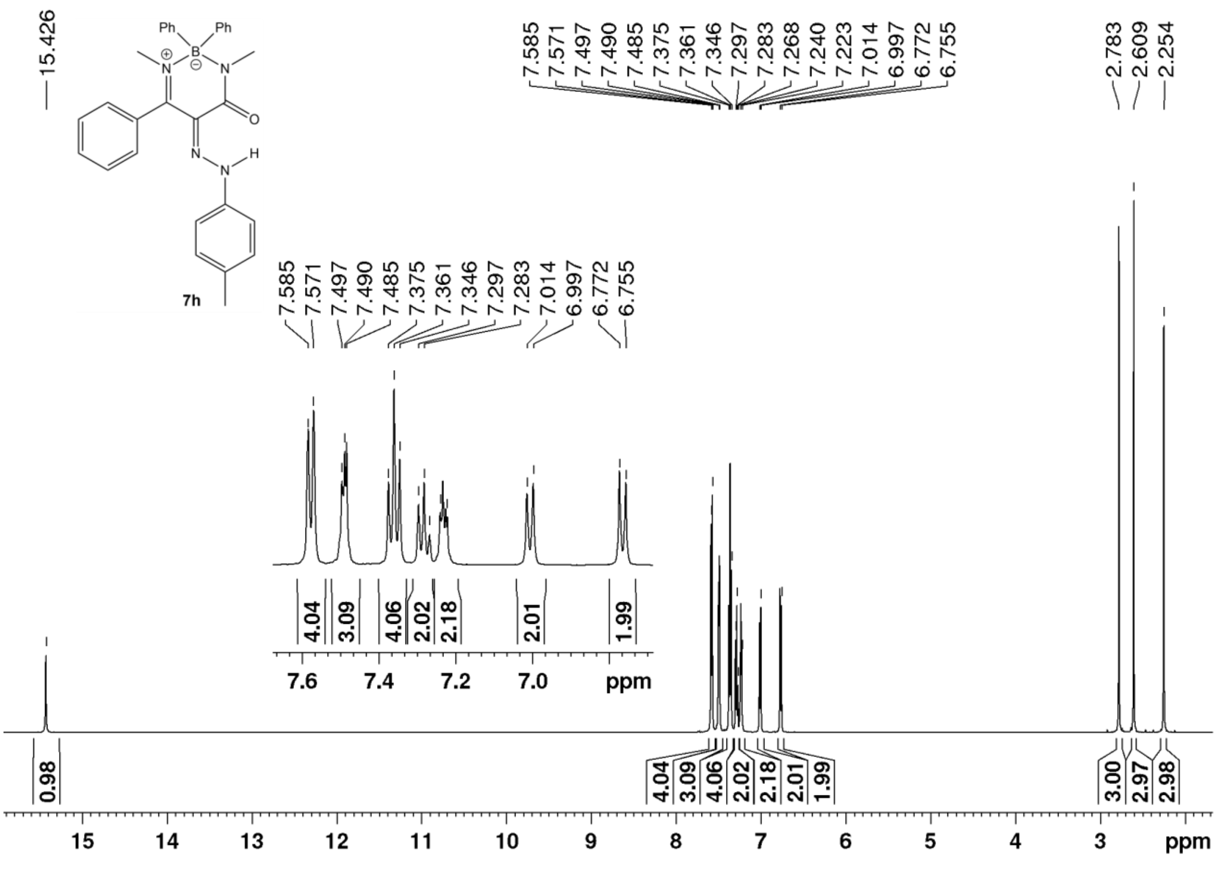

Figure S85. 500 MHz ^{1}H NMR of compound **7h** in $CDCl_3$

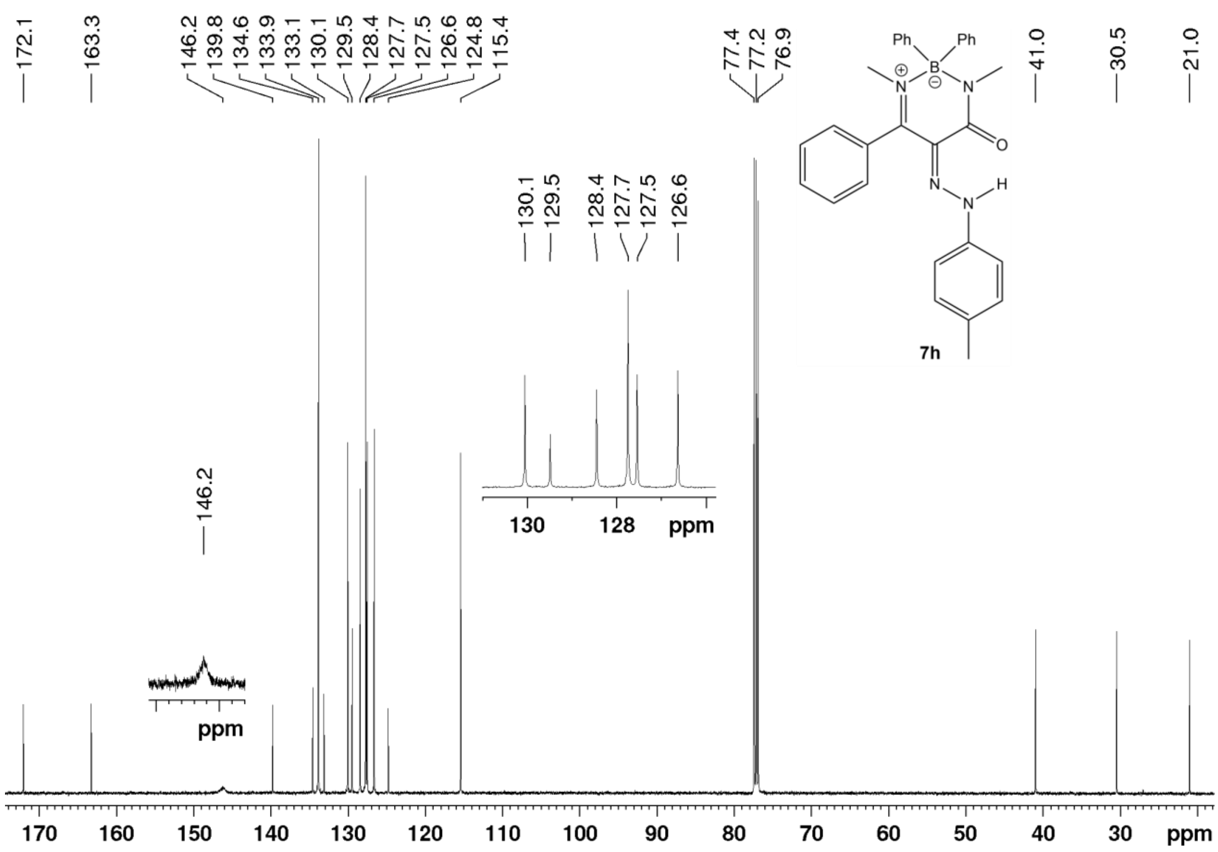

Figure S86. 125 MHz ^{13}C NMR of compound **7h** in $CDCl_3$

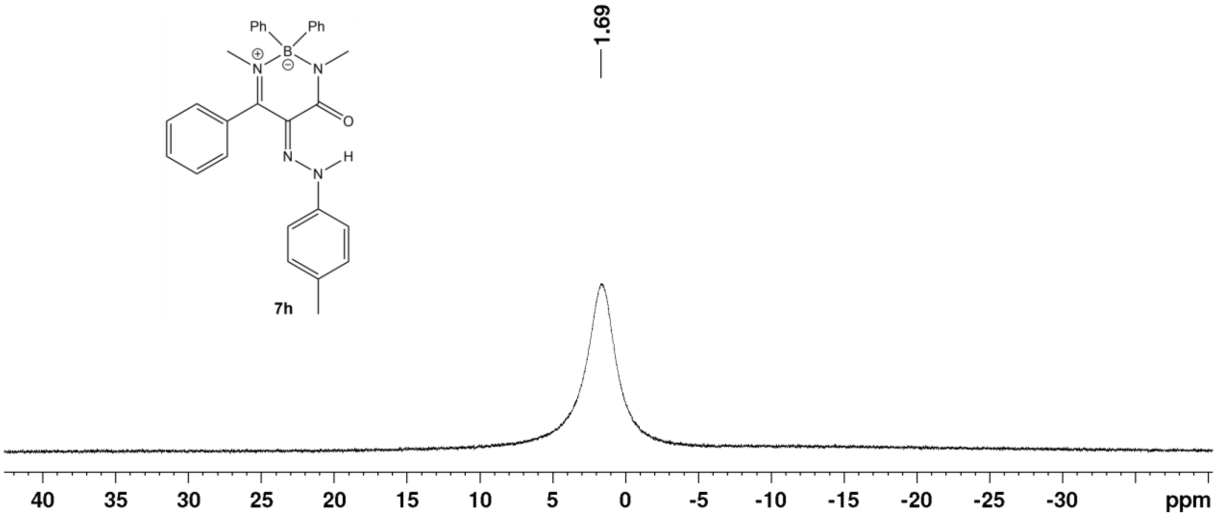

Figure S87. 160 MHz ^{11}B NMR of compound **7h** in $CDCl_3$

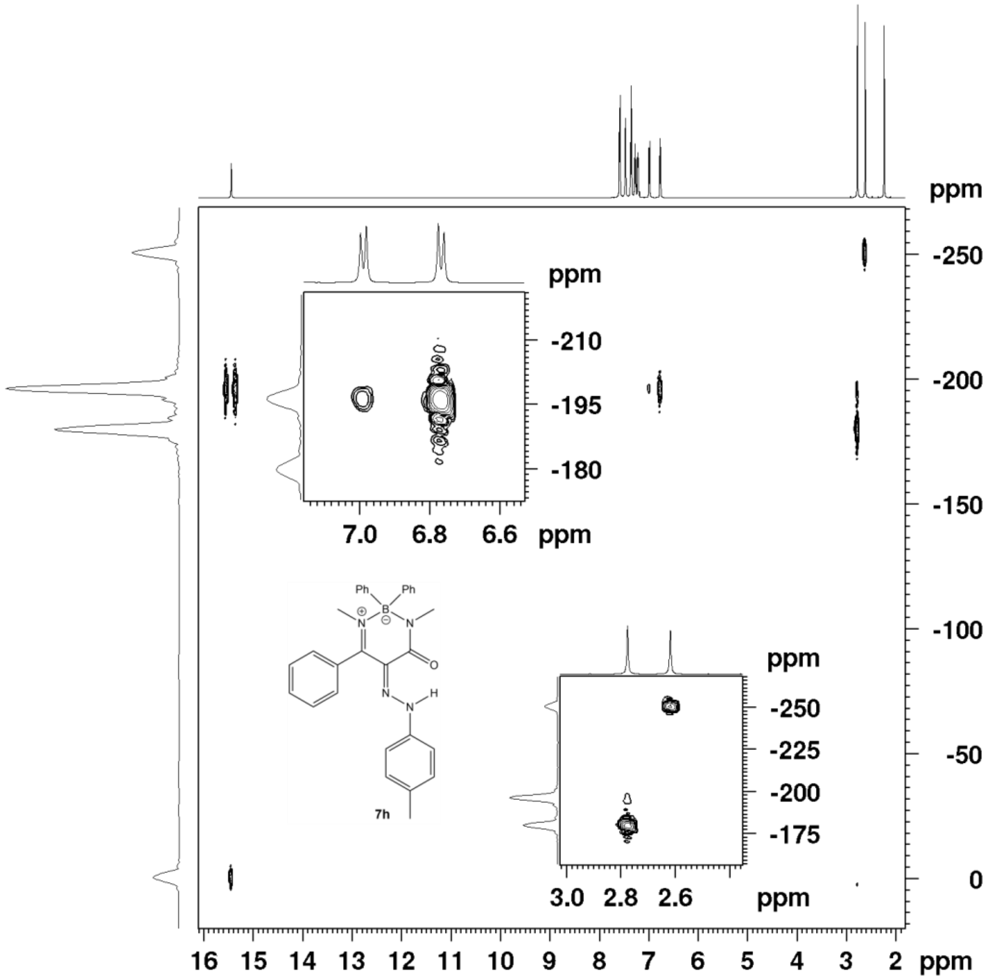

Figure S88. 500 MHz ^{1}H–^{15}N HMBC NMR of compound **7h** in $CDCl_3$

2.20 Triazaborine 8e

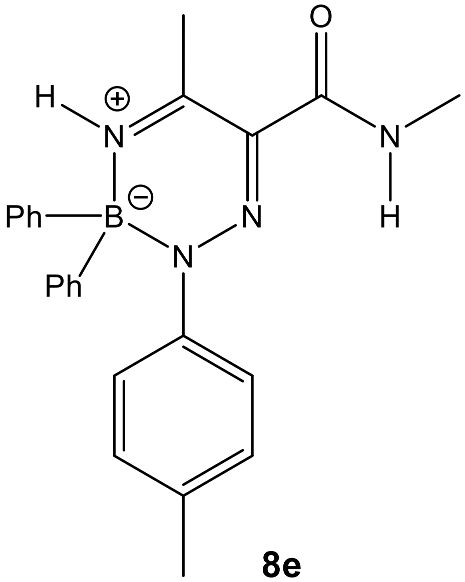

8e

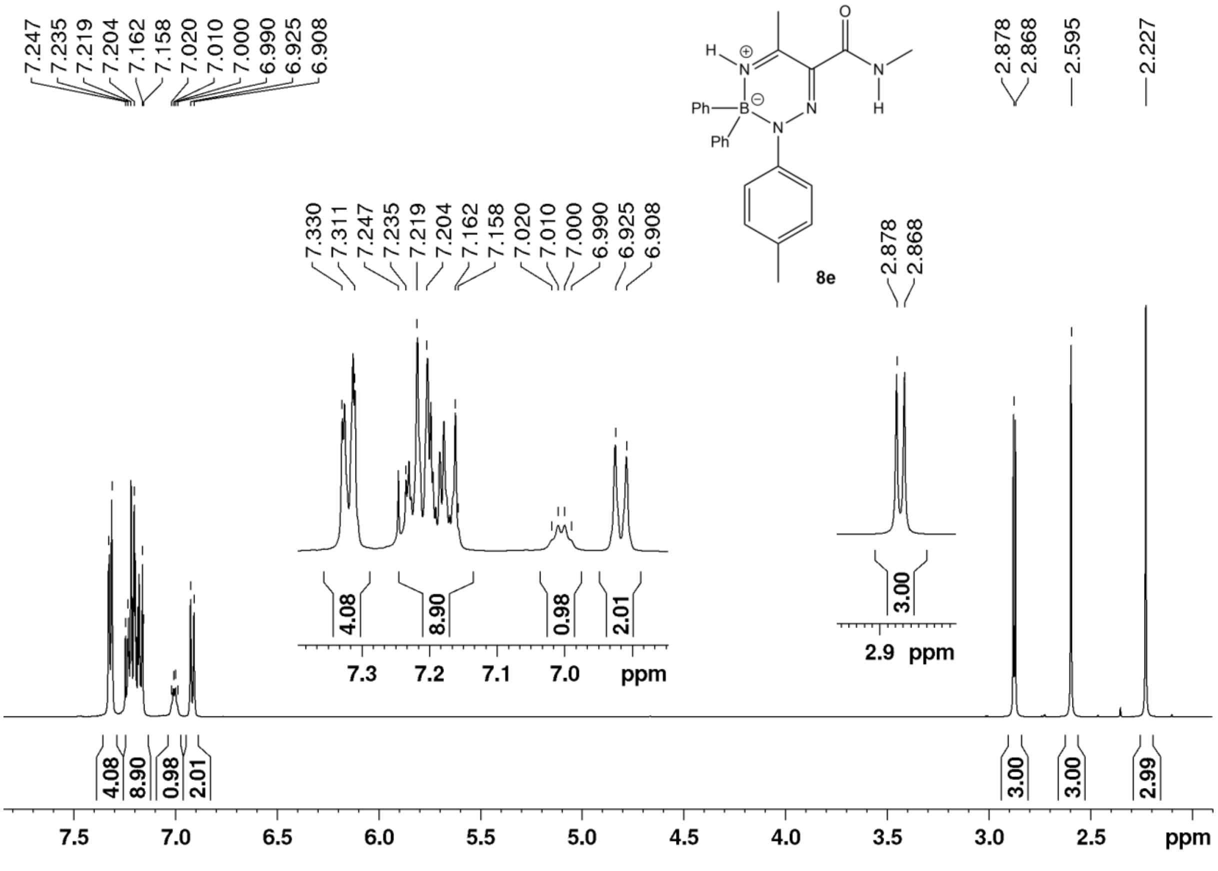

Figure S89. 500 MHz ^{1}H NMR of compound **8e** in $CDCl_3$

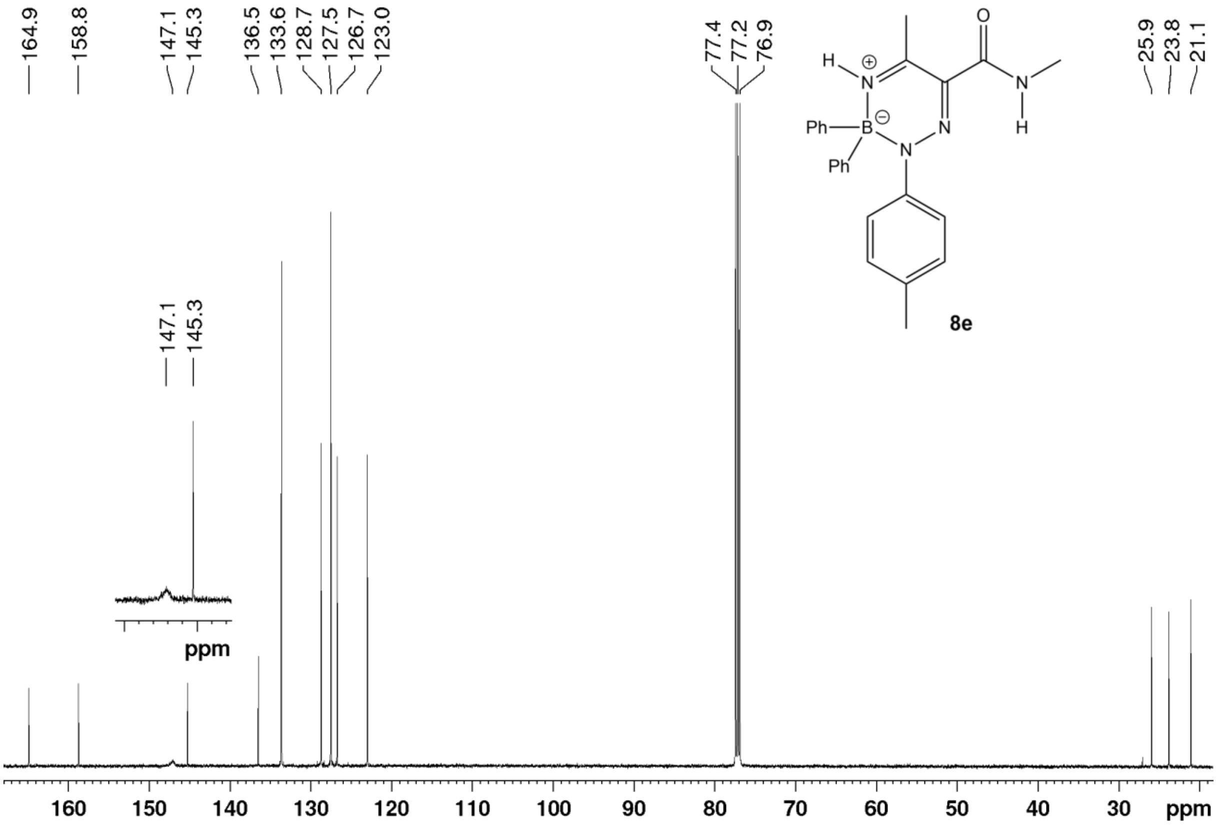

Figure S90. 125 MHz ^{13}C NMR of compound **8e** in $CDCl_3$

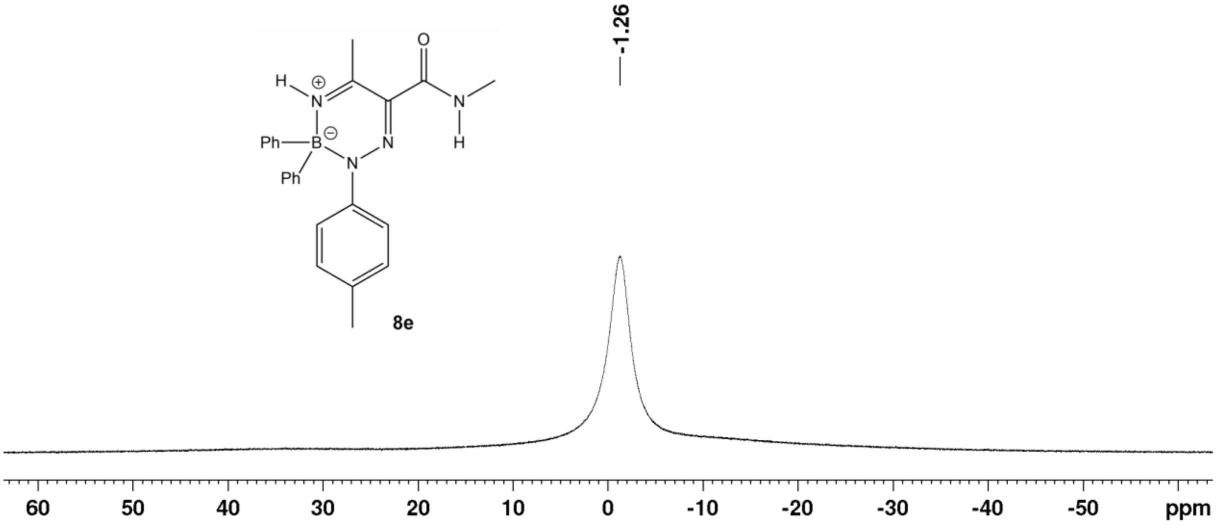

Figure S91. 160 MHz ^{11}B NMR of compound **8e** in $CDCl_3$

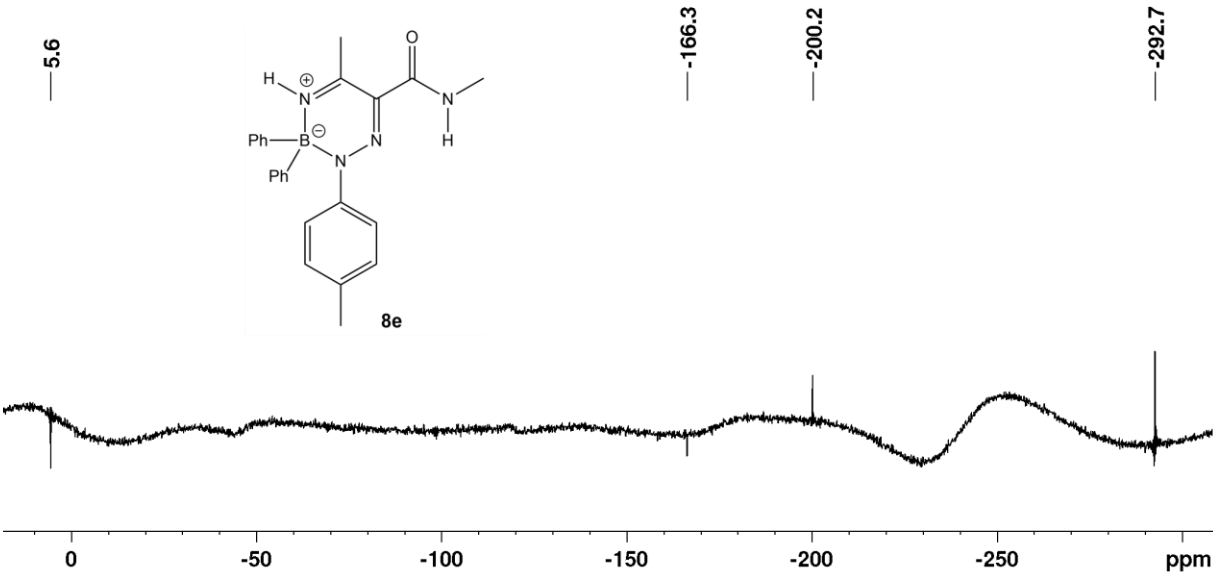

Figure S92. 50 MHz ^{15}N NMR of compound **8e** in $CDCl_3$

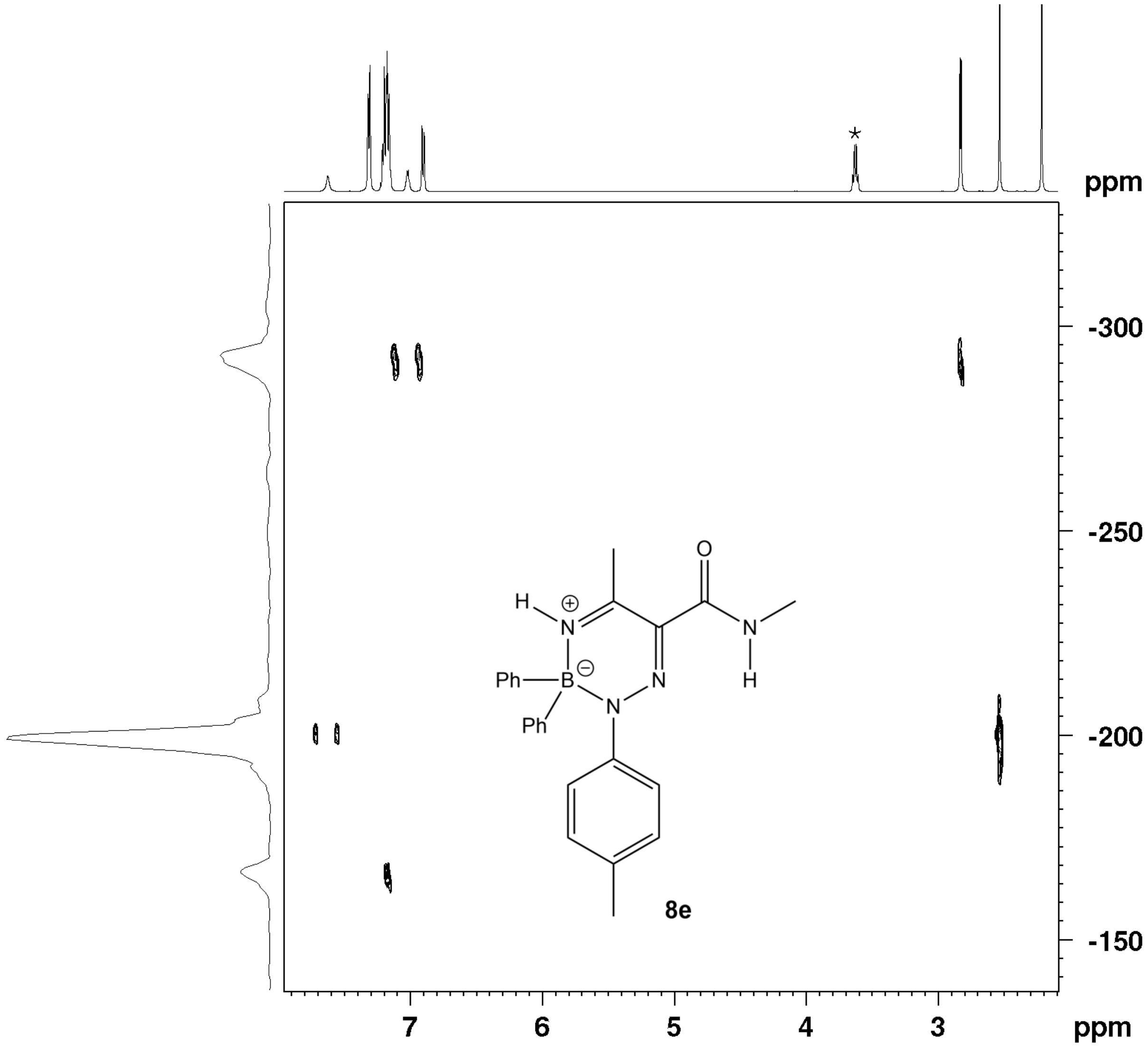

Figure S93. 500 MHz ^{1}H–^{15}N HMBC NMR of compound **8e** in $CDCl_3$ (* CH_2 from ethanol)

2.21 Triazaborine 8g

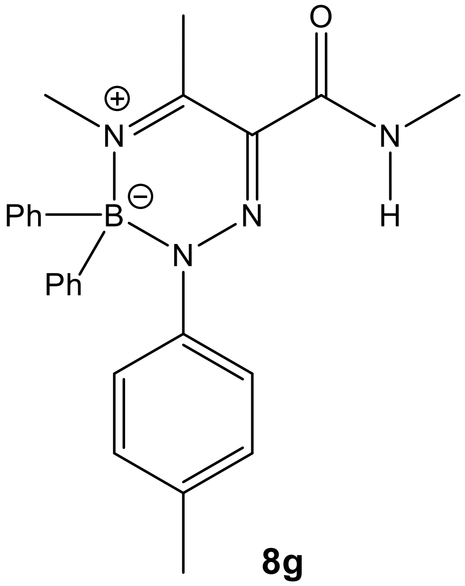

8g

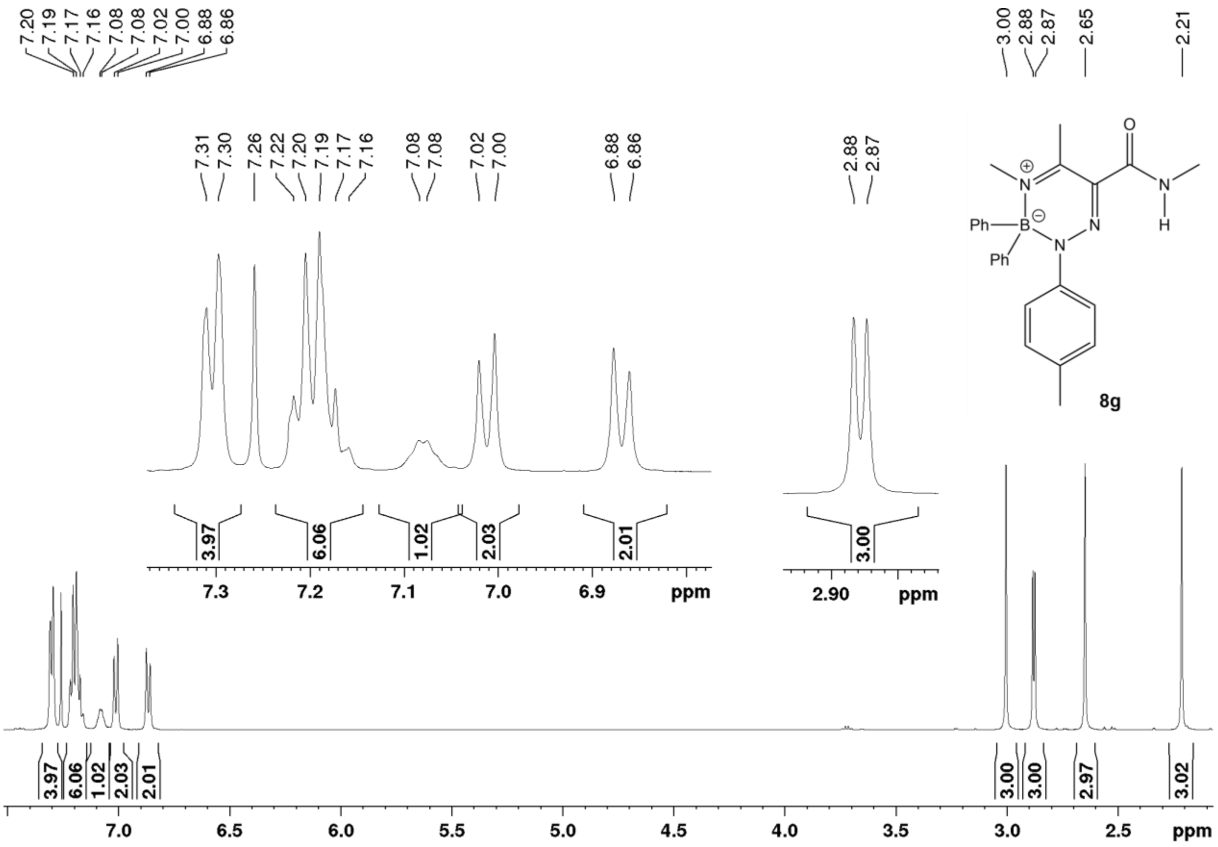

Figure S94. 500 MHz ^{1}H NMR of compound **8g** in $CDCl_3$

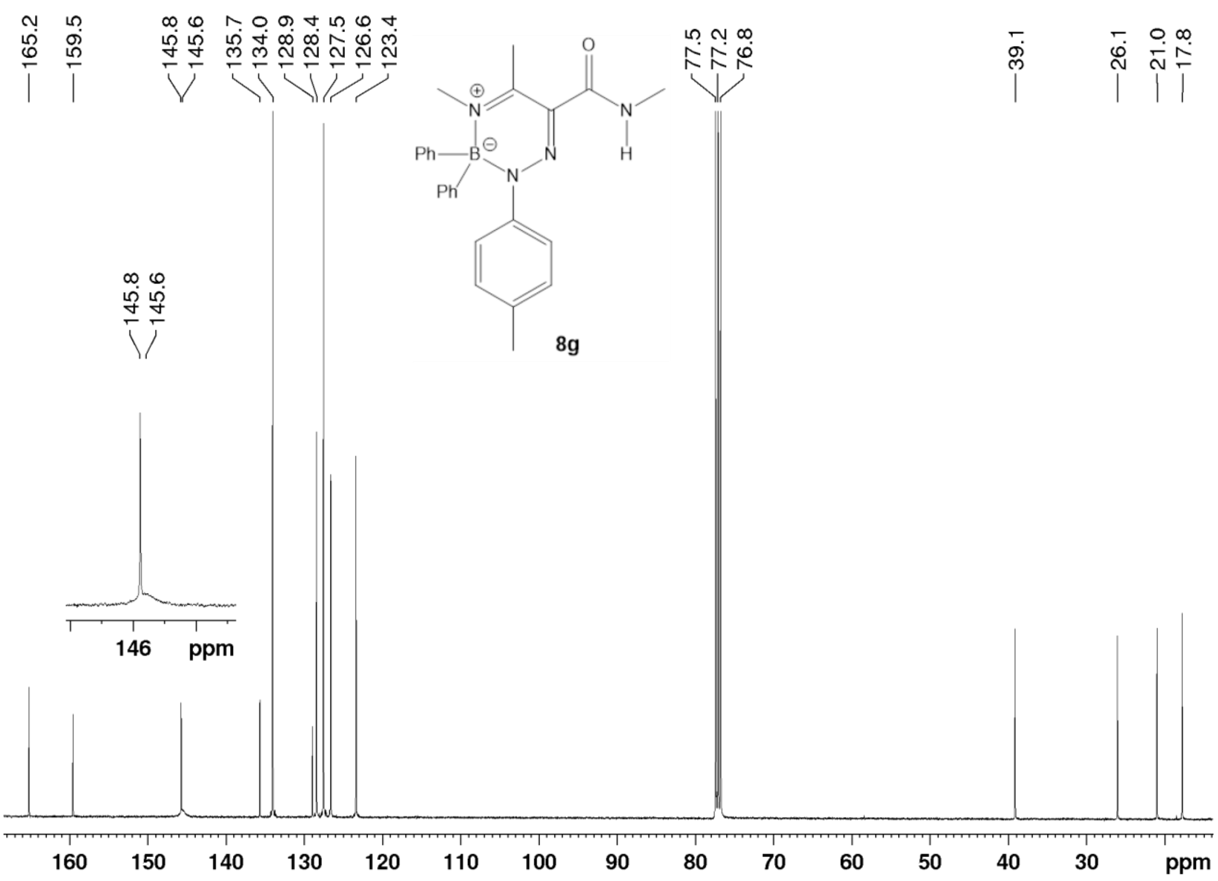

Figure S95. 100 MHz ^{13}C NMR of compound **8g** in $CDCl_3$

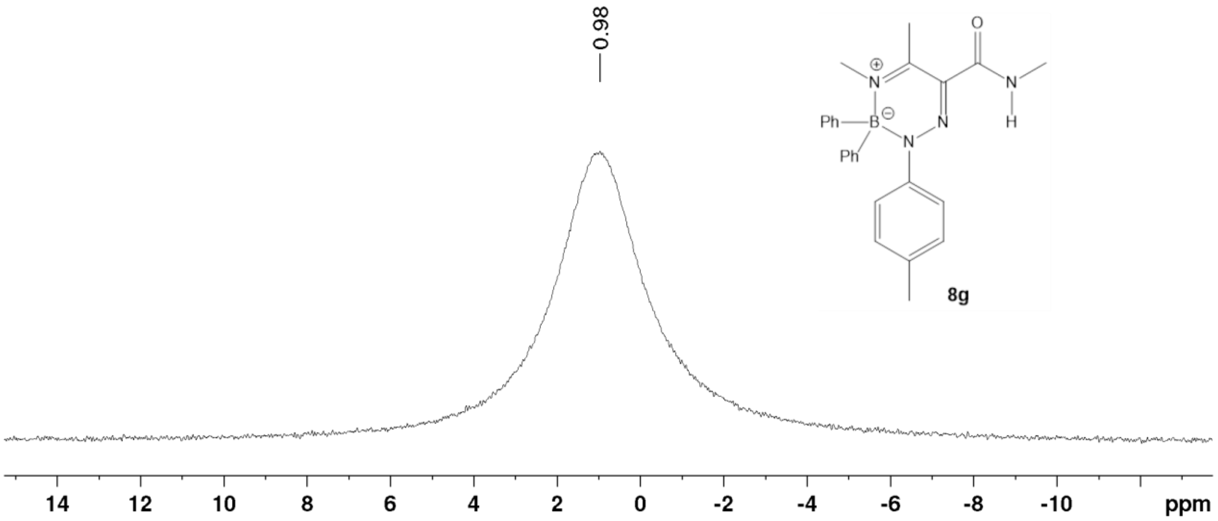

Figure S96. 128 MHz ^{11}B NMR of compound **8g** in $CDCl_3$

2.22 Triazaborine 8h

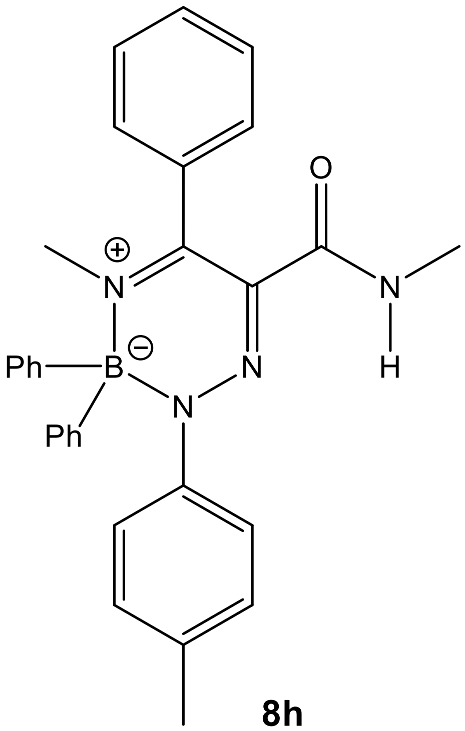

8h

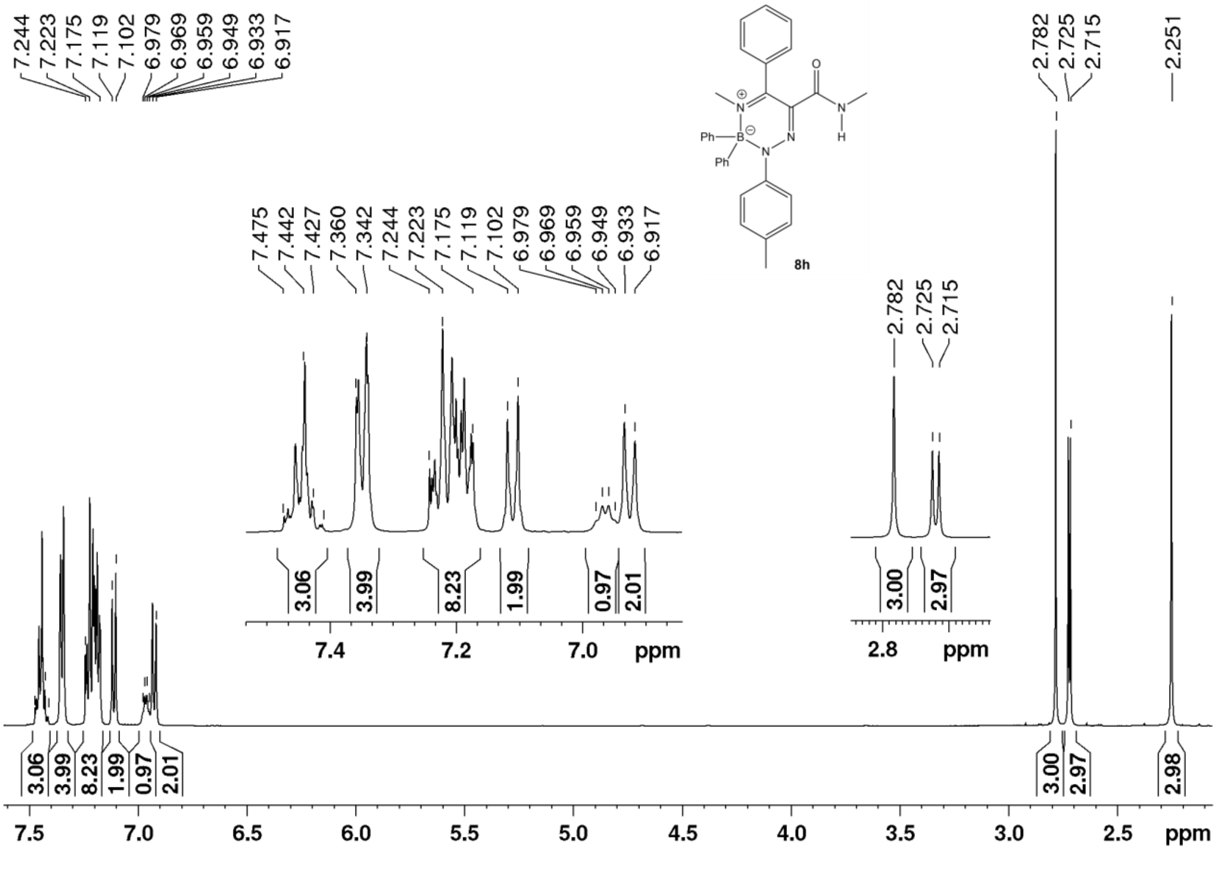

Figure S97. 500 MHz ^{1}H NMR of compound **8h** in $CDCl_3$

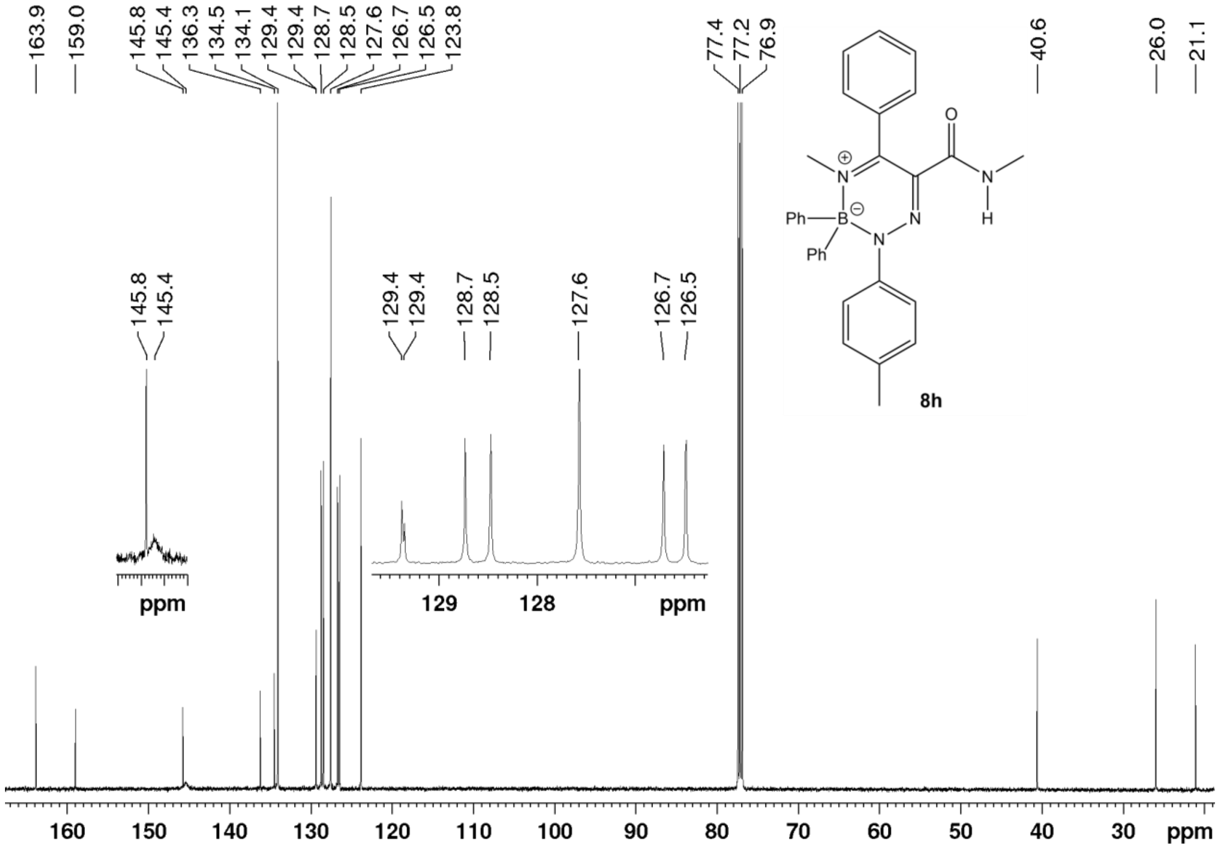

Figure S98. 125 MHz ^{13}C NMR of compound **8h** in $CDCl_3$

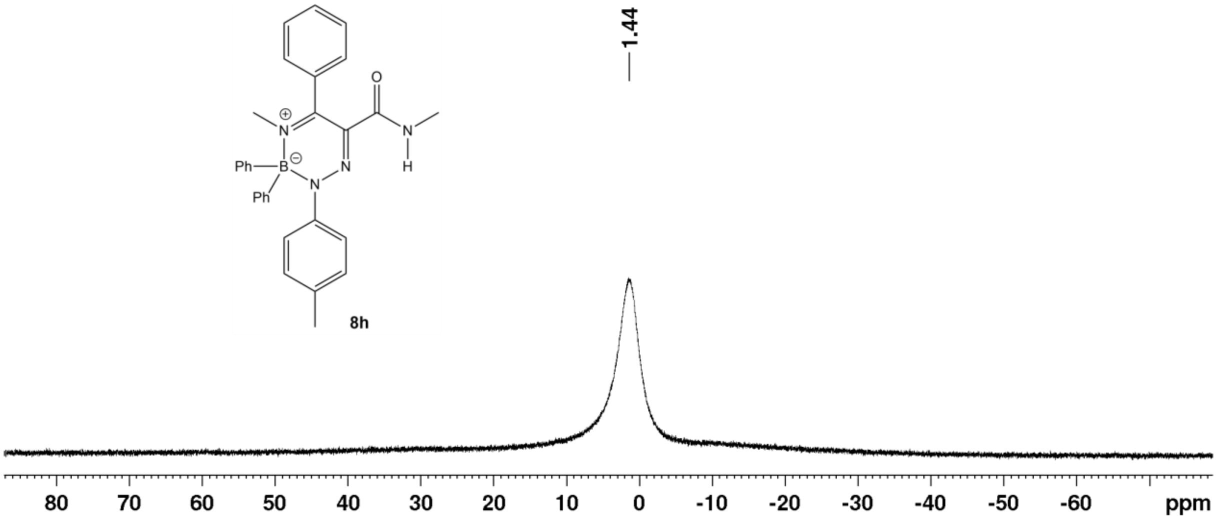

Figure S99. 160 MHz ^{11}B NMR of compound **8h** in $CDCl_3$

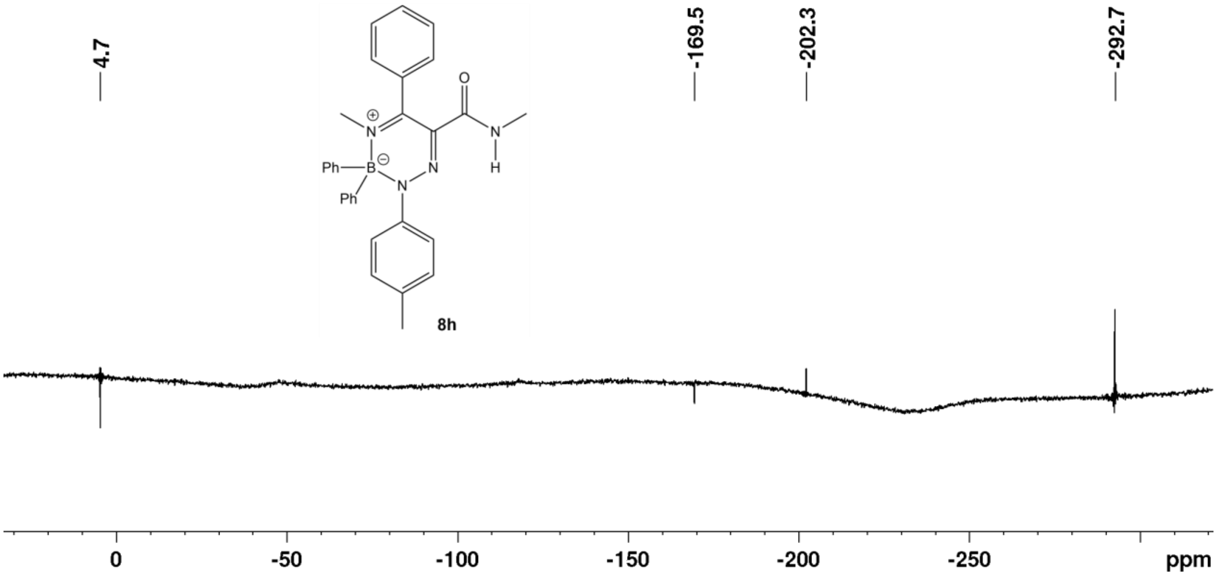

Figure S100. 50 MHz ^{15}N NMR of compound **8h** in $CDCl_3$

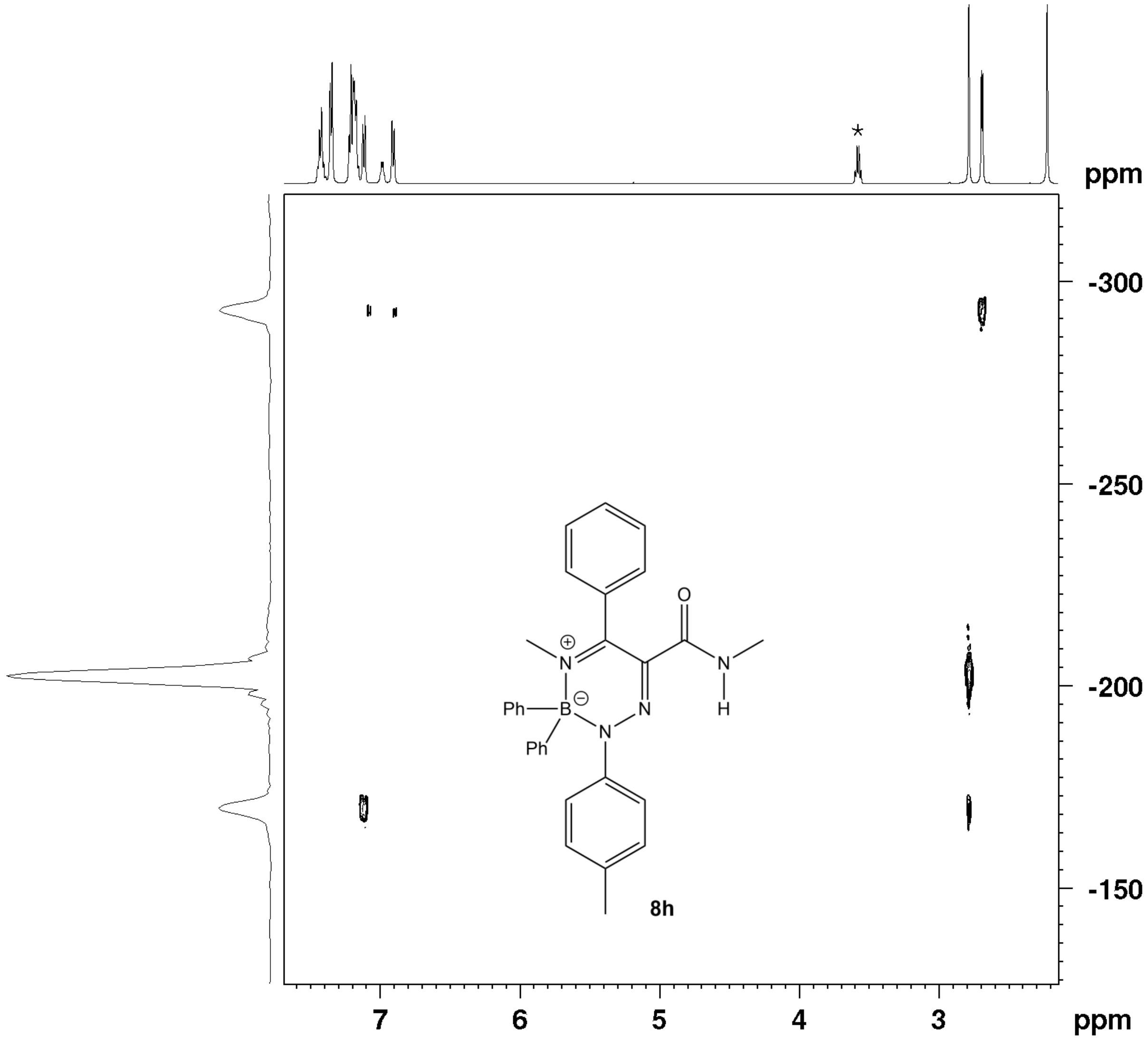

Figure S101. 500 MHz ^1H–^{15}N HMBC NMR of compound 8h in $CDCl_3$ (* CH_2 from ethanol)

2.23 Oxazaborine–Triazaborine 9g

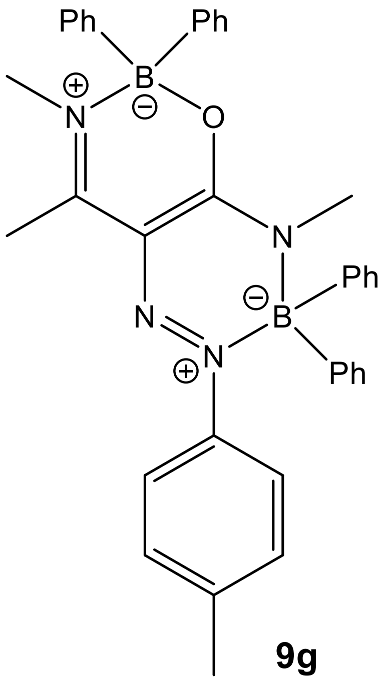

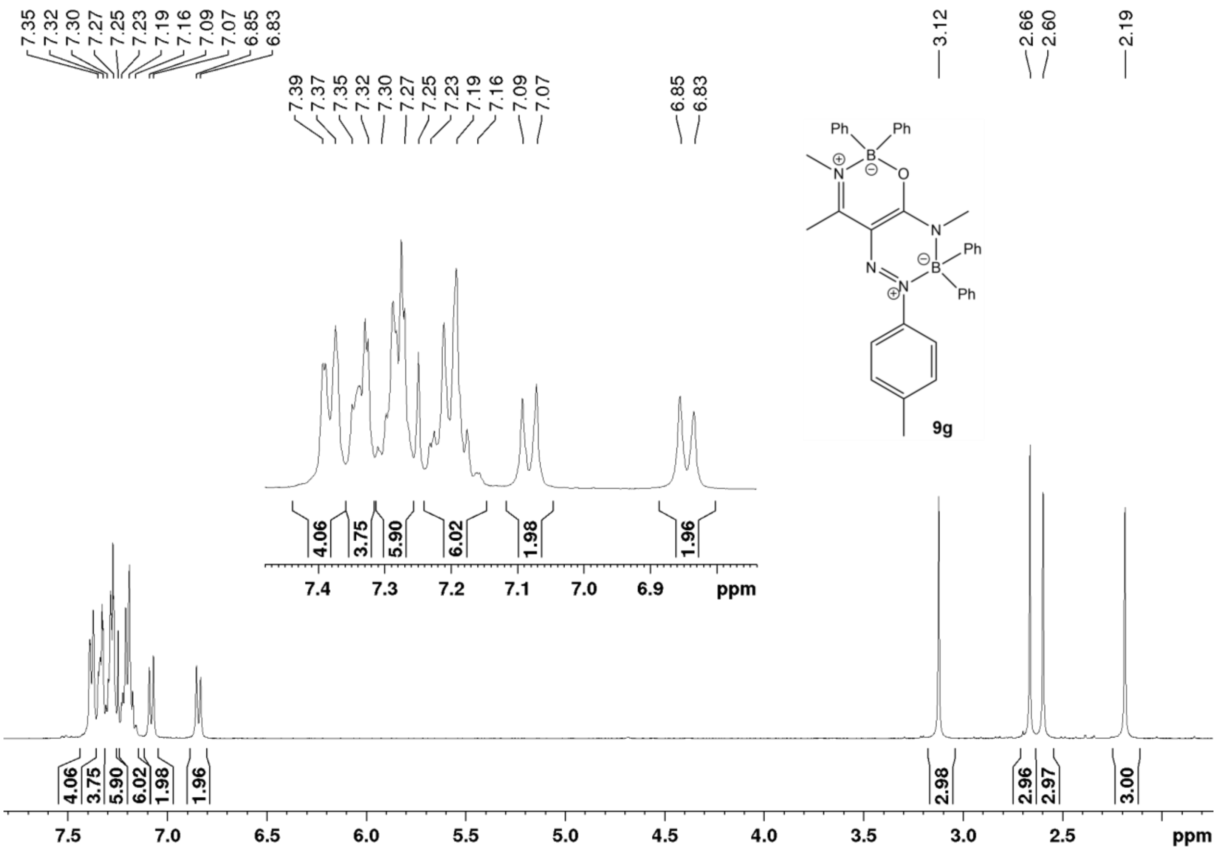

Figure S102. 400 MHz ^{1}H NMR of compound **9g** in $CDCl_3$

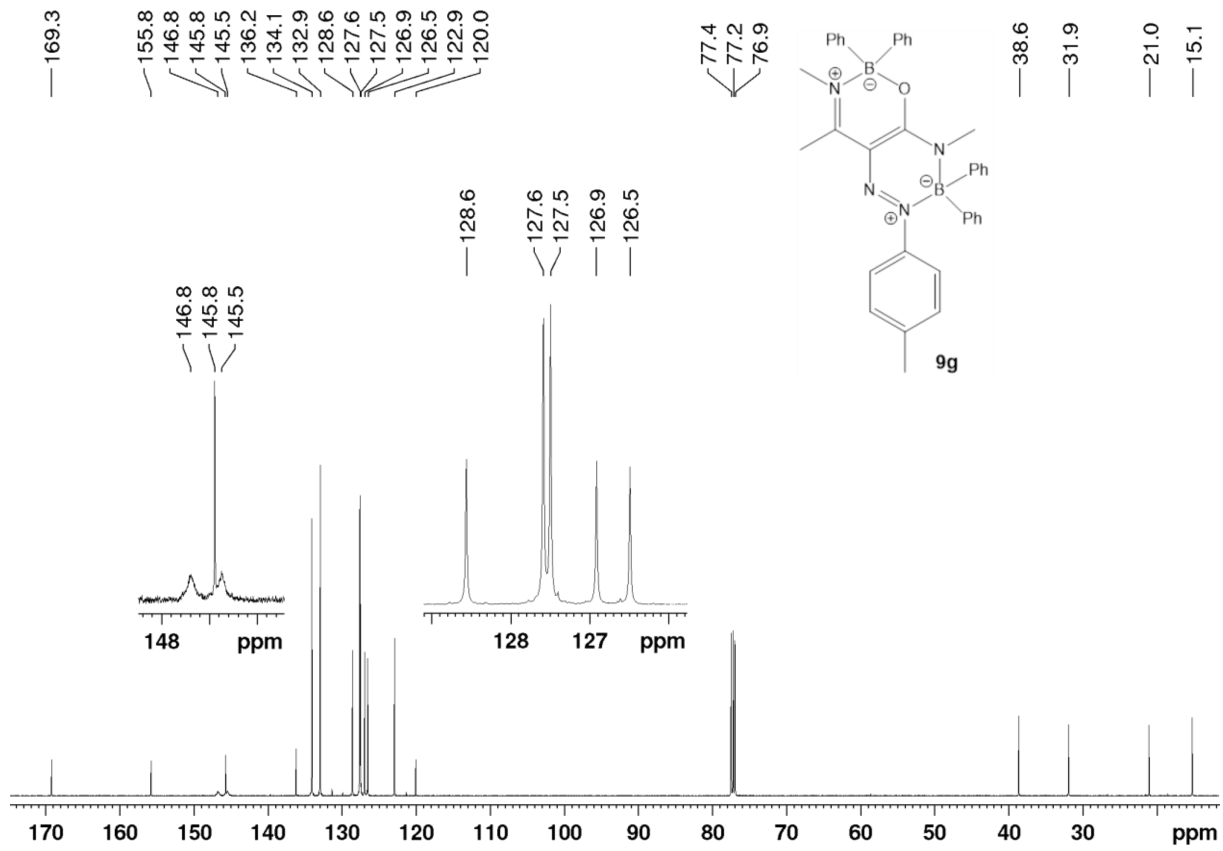

Figure S103. 125 MHz ^{13}C NMR of compound **9g** in $CDCl_3$

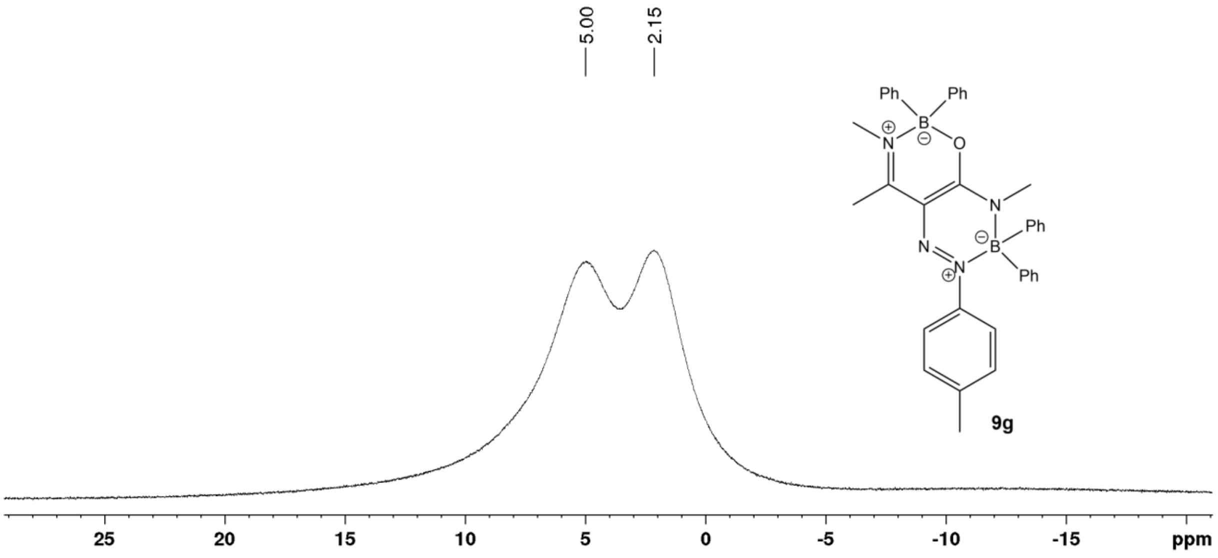

Figure S104. 160 MHz ^{11}B NMR of compound **9g** in $CDCl_3$

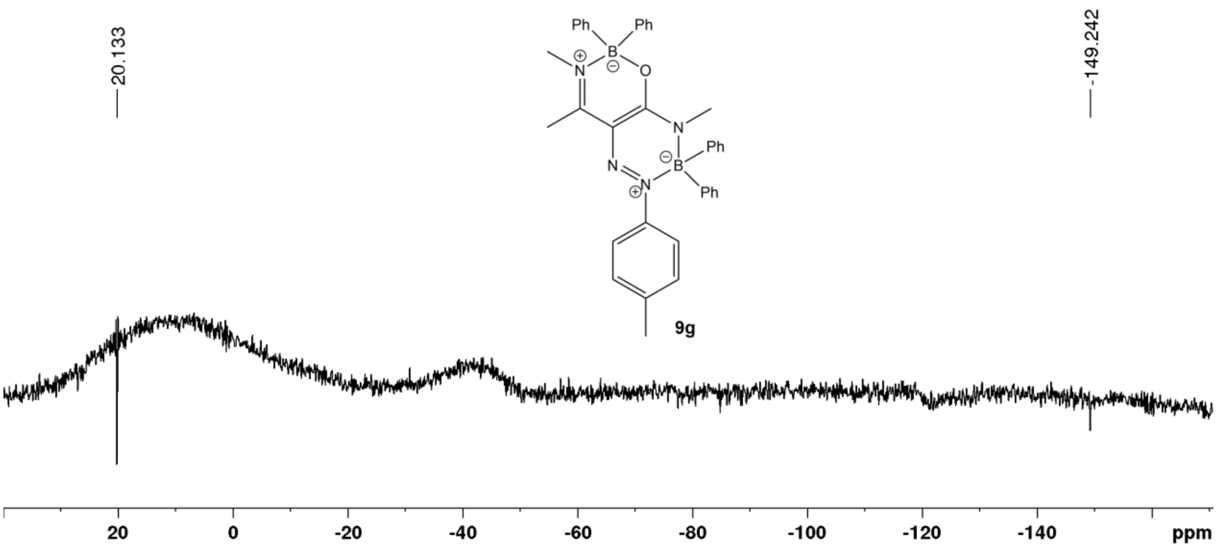

Figure S105. 50 MHz ^{15}N NMR of compound **9g** in $CDCl_3$

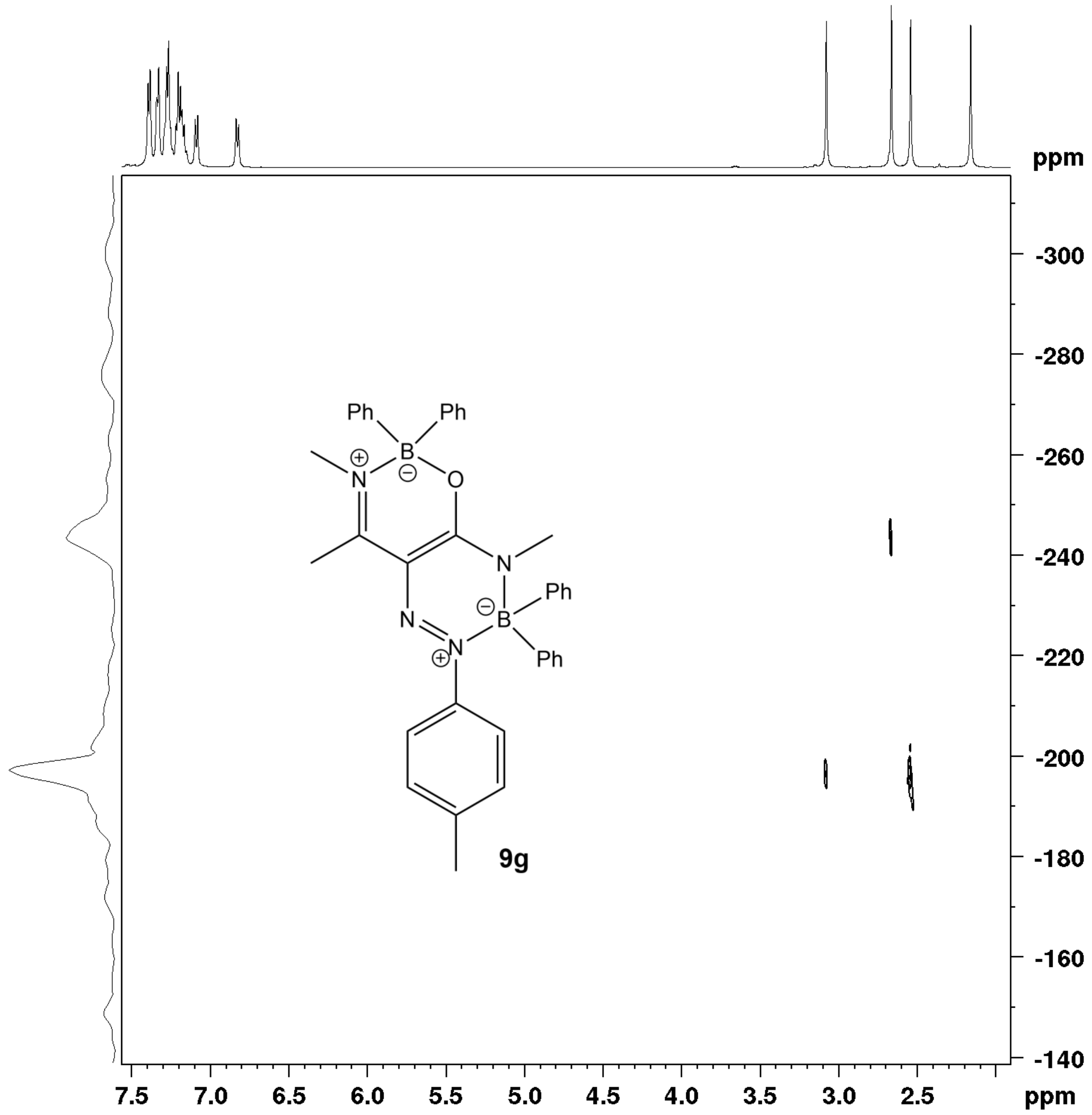

Figure S106. 500 MHz ^{1}H–^{15}N HMBC NMR of compound **9g** in $CDCl_3$

2.24 Oxazaborine–Triazaborine 9h

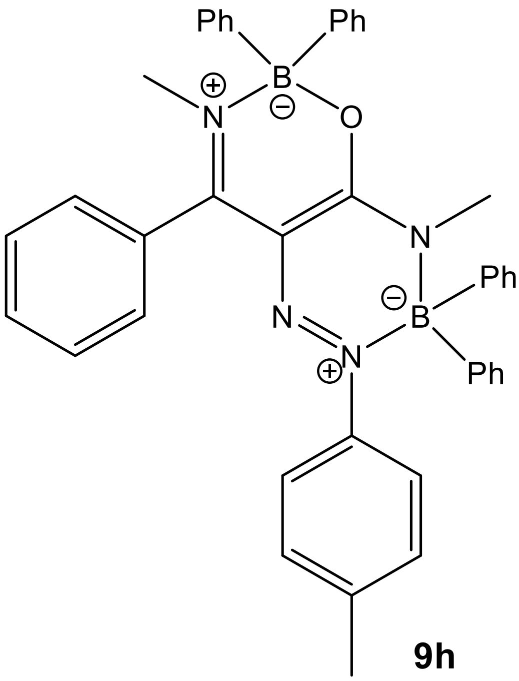

9h

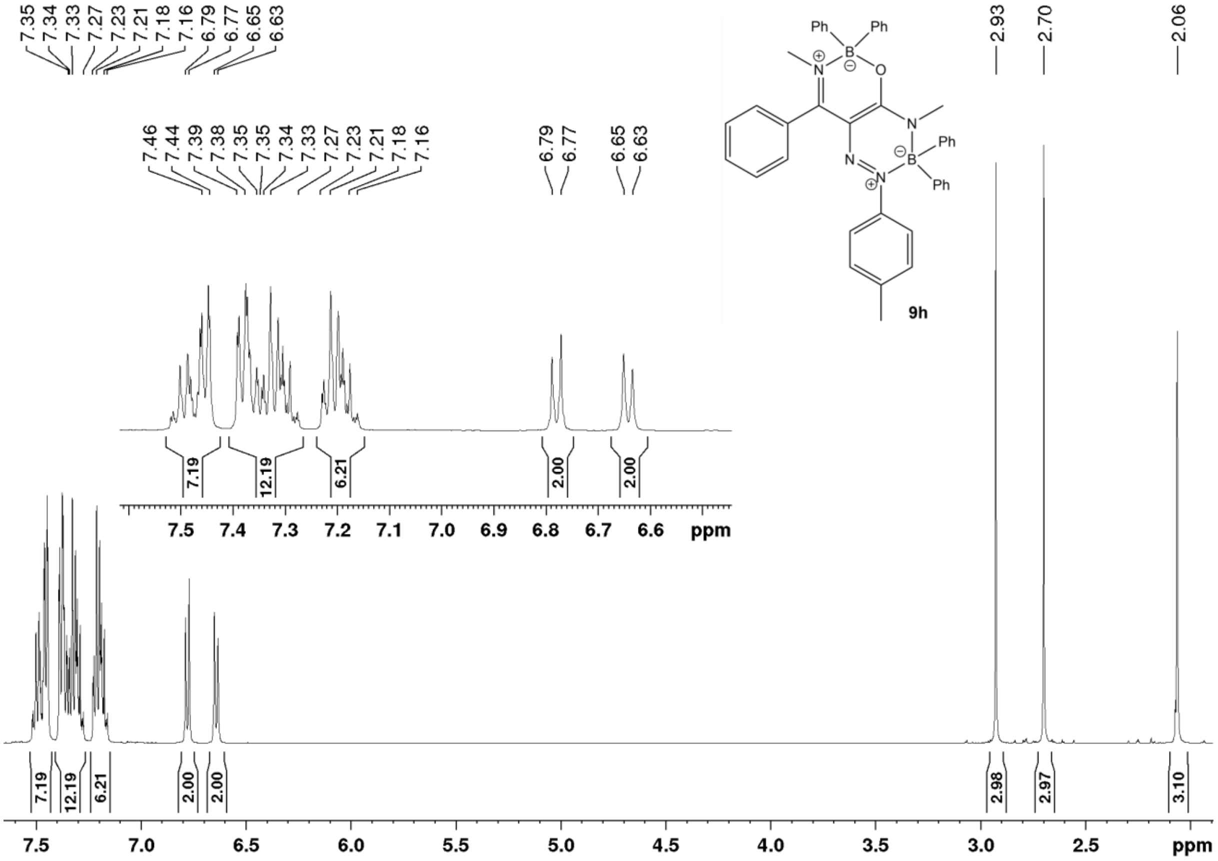

Figure S107. 500 MHz ^{1}H NMR of compound **9h** in $CDCl_3$

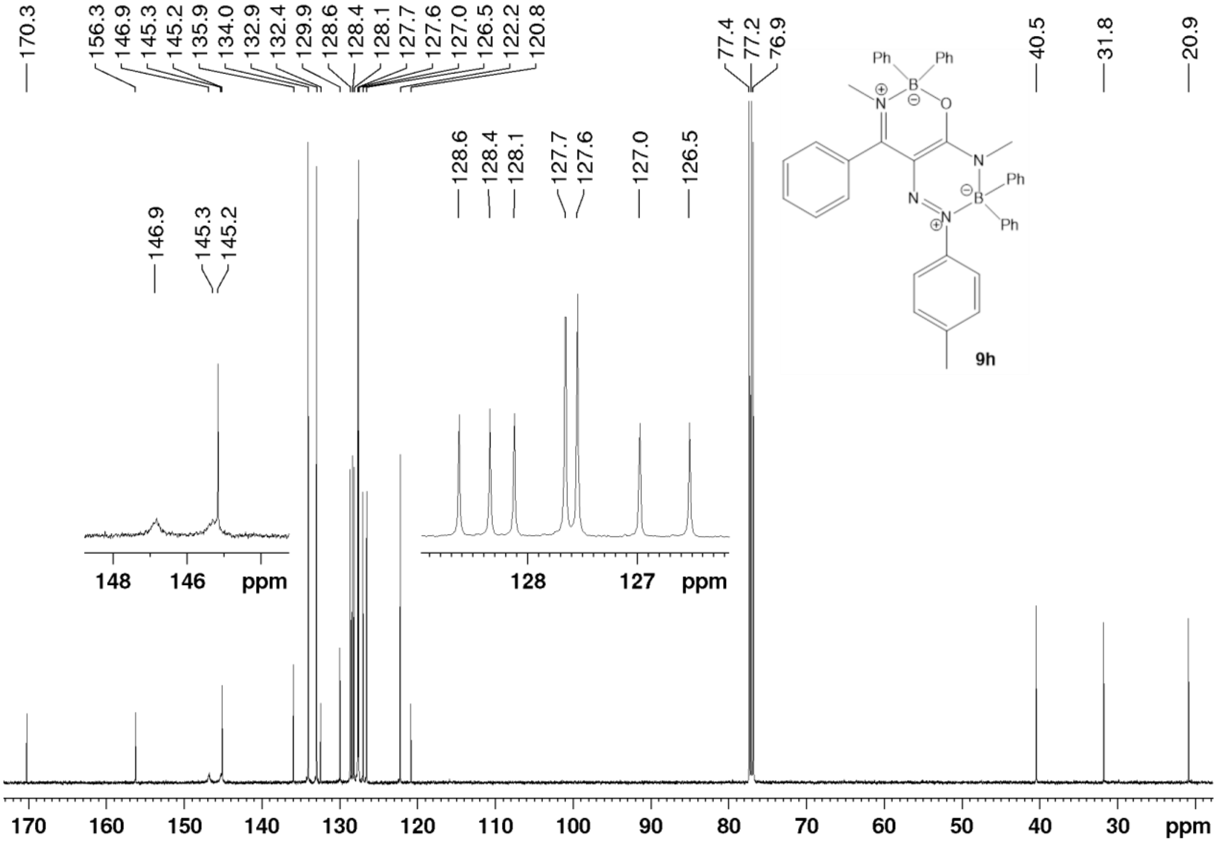

Figure S108. 125 MHz ^{13}C NMR of compound **9h** in $CDCl_3$

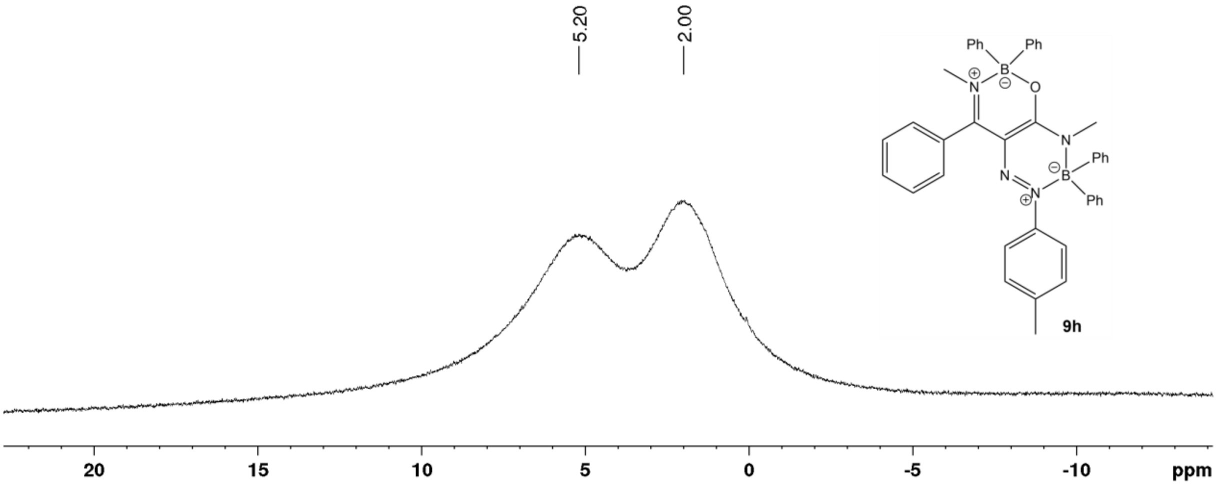

Figure S109. 160 MHz ^{11}B NMR of compound **9h** in $CDCl_3$

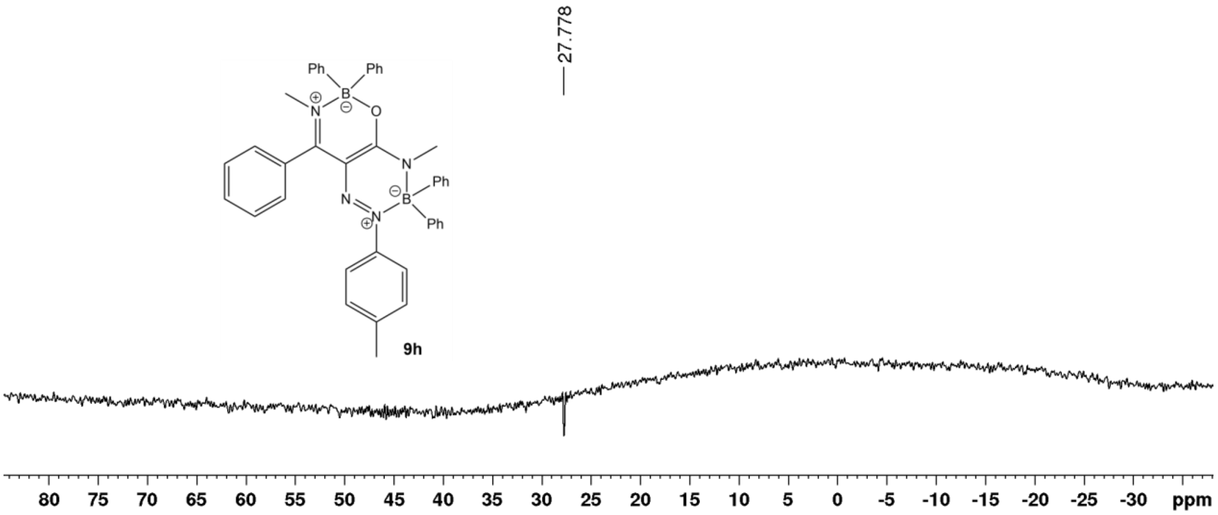

Figure S110. 50 MHz ^{15}N NMR of compound **9h** in $CDCl_3$

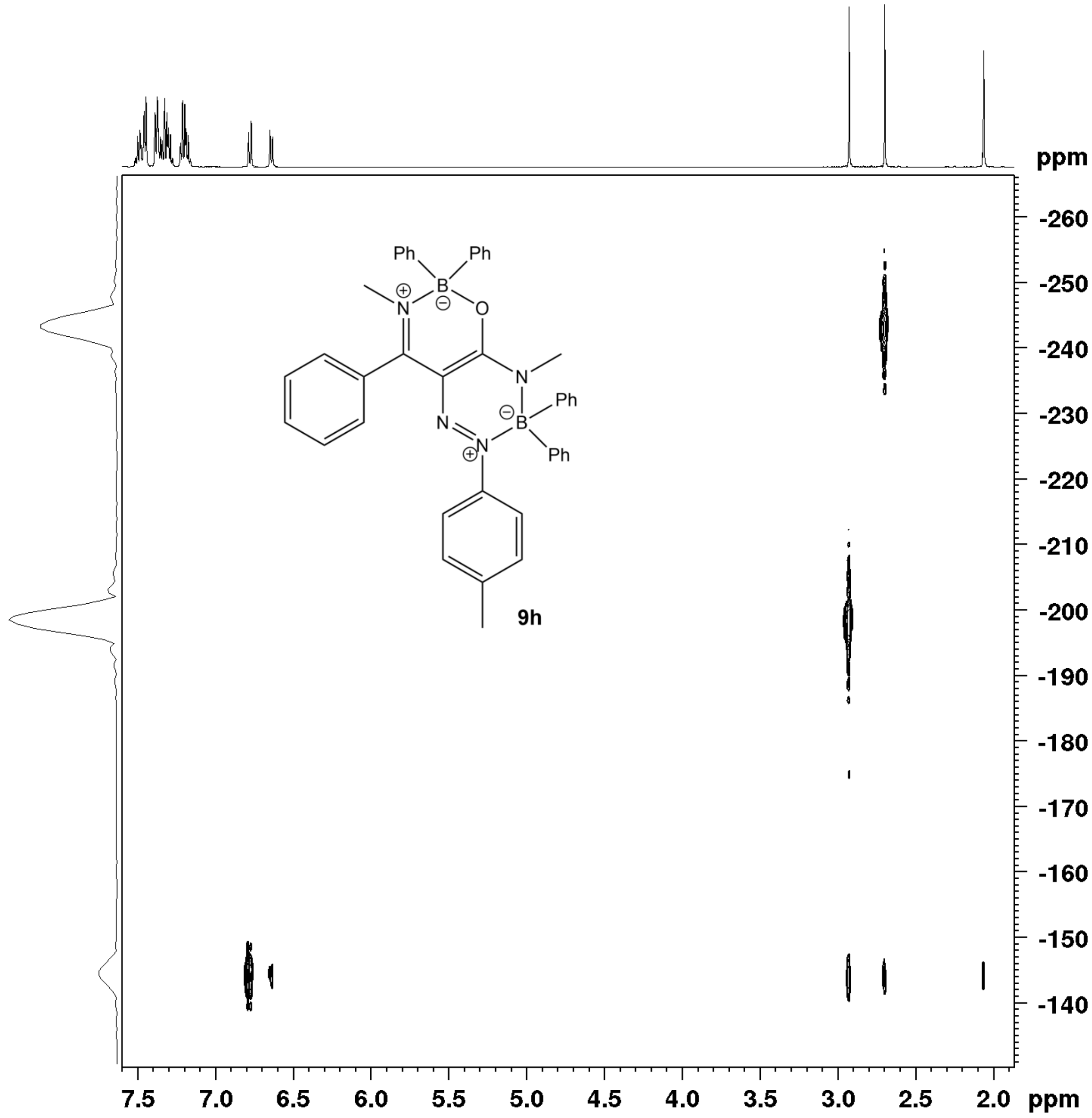

Figure S111. 500 MHz ^{1}H–^{15}N HMBC NMR of compound **9h** in $CDCl_3$

3. UV–Vis spectra

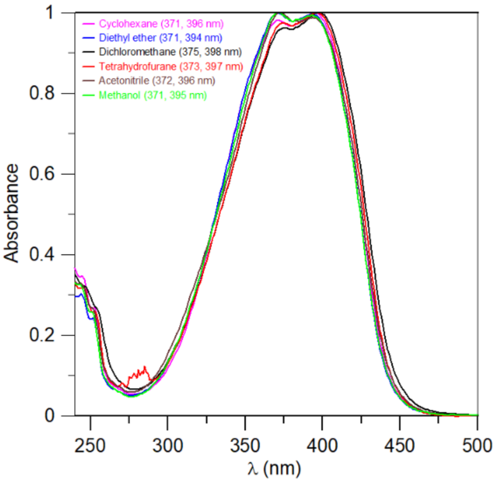

Figure S112. Normalized UV–VIS spectrum of oxazaborine **6g.**

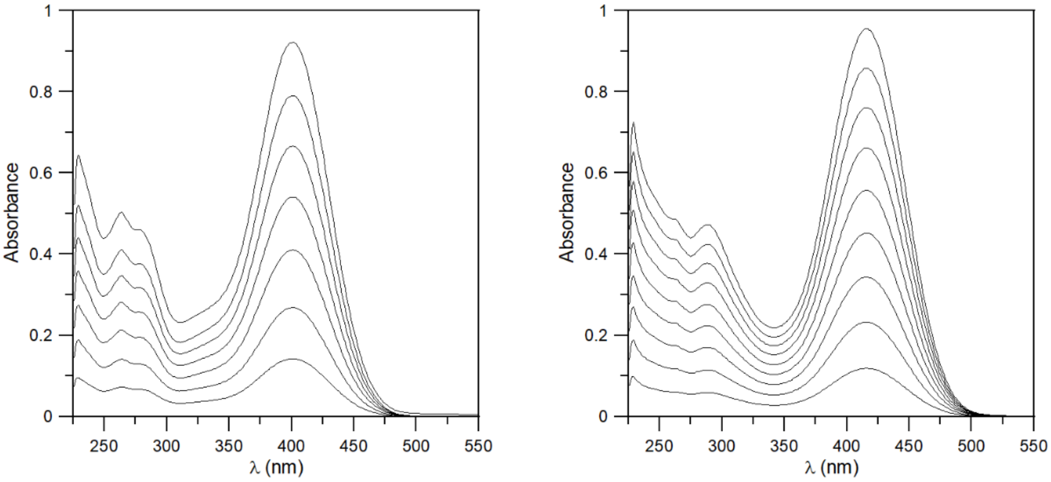

(**a**) (**b**)

Figure S113. UV–Vis spectrum in CH_2Cl_2. (**a**) triazaborinone **5a** and (**b**) triazaborinone **5b**.

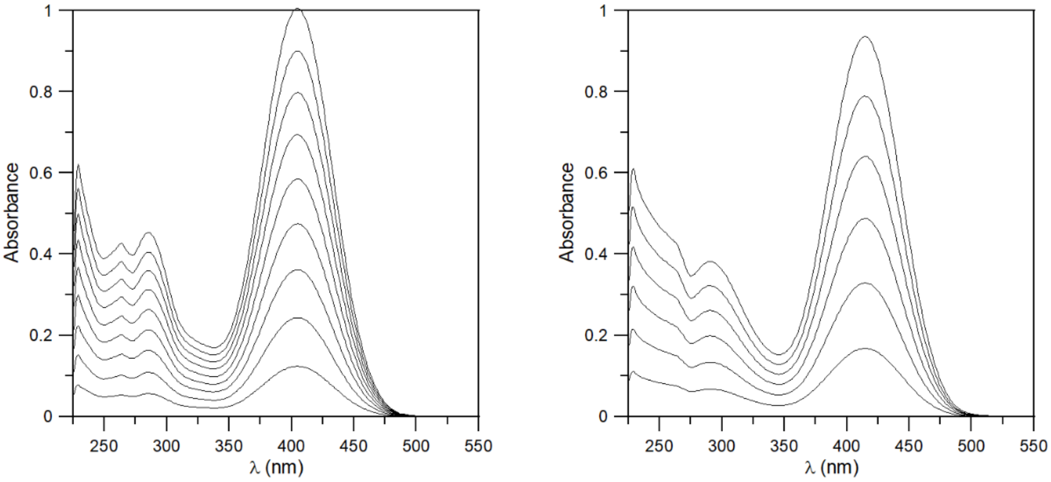

(**a**) (**b**)

Figure S114. UV–Vis spectrum in CH_2Cl_2. (**a**) triazaborinone **5c** and (**b**) triazaborinone **5d**.

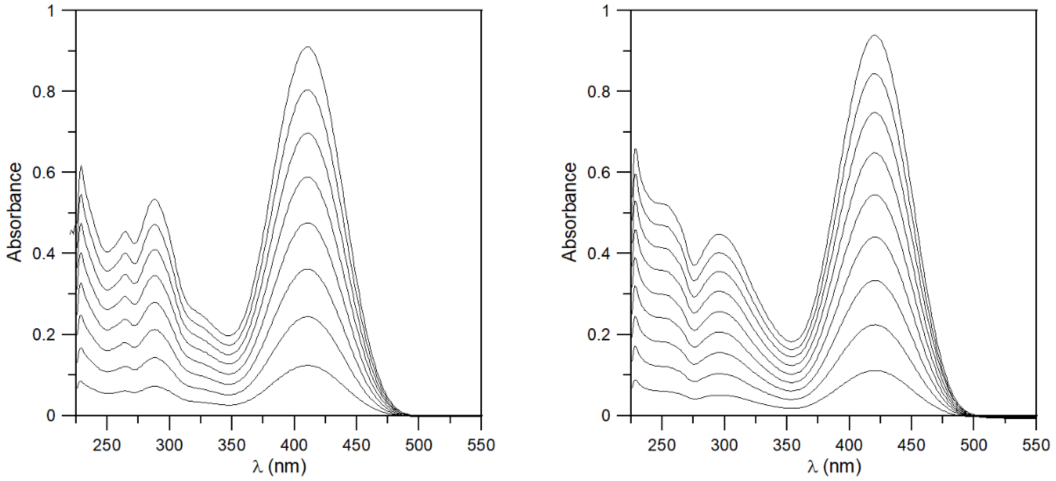

(**a**) (**b**)

Figure S115. UV–Vis spectrum in CH_2Cl_2. (**a**) triazaborinone **5g** and (**b**) triazaborinone **5h**.

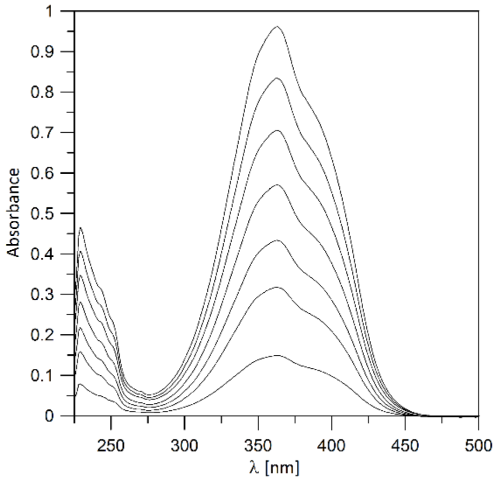

(**a**)

Figure S116. UV–Vis spectrum in CH_2Cl_2 oxazaborine **6a**.

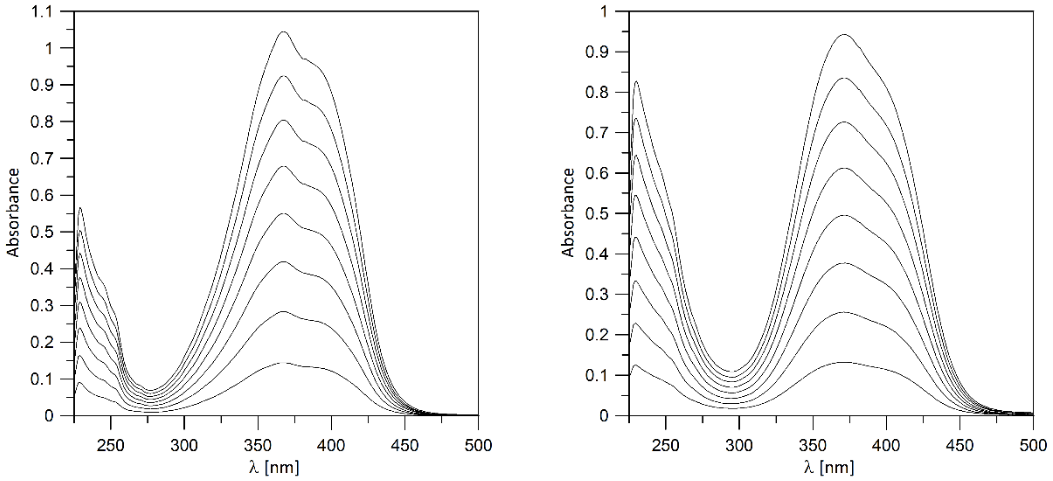

(**a**) (**b**)

Figure S117. UV–Vis spectrum in CH_2Cl_2. (**a**) oxazaborine **6e** and (**b**) oxazaborine **6f**.

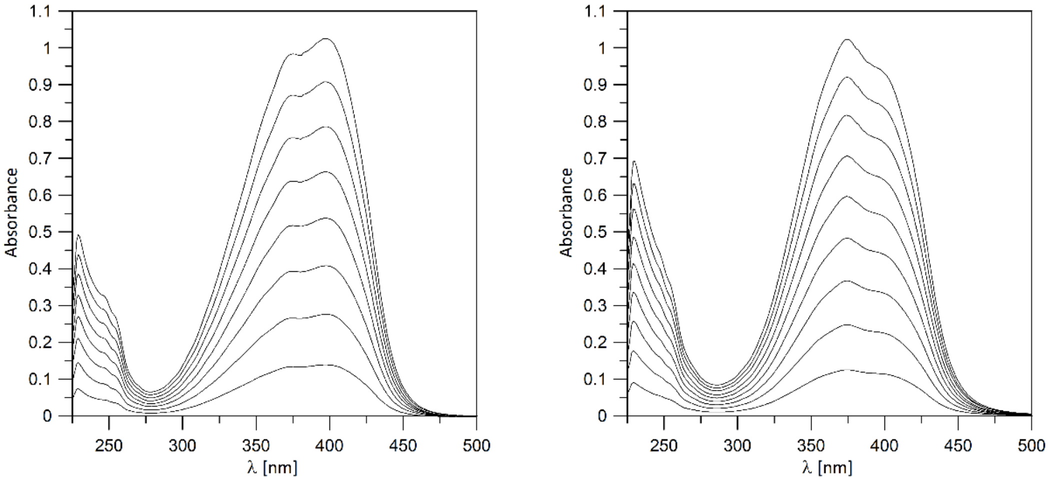

(**a**) (**b**)

Figure S118. UV–Vis spectrum in CH_2Cl_2. (**a**) oxazaborine **6g** and (**b**) oxazaborine **6h**.

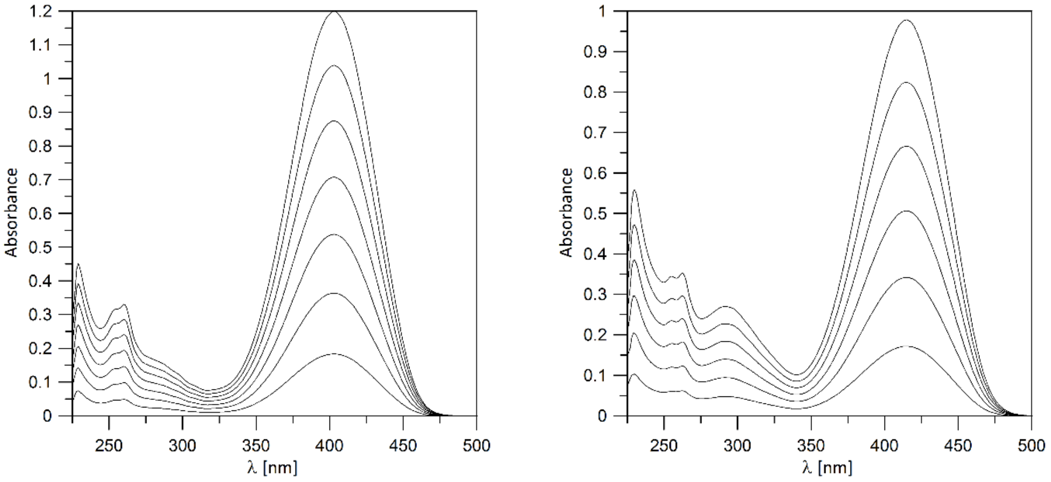

(**a**) (**b**)

Figure S119. UV–Vis spectrum in CH_2Cl_2. (**a**) diazaborine 7**a** and (**b**) diazaborinone **7b**.

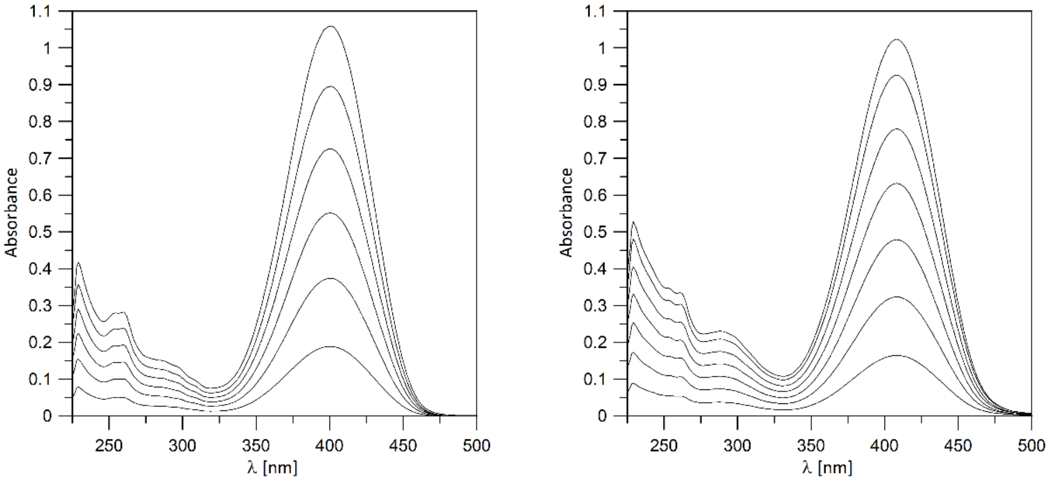

(**a**) (**b**)

Figure S120. UV–Vis spectrum in CH_2Cl_2. (**a**) diazaborine **7c** and (**b**) diazaborinone **7d**.

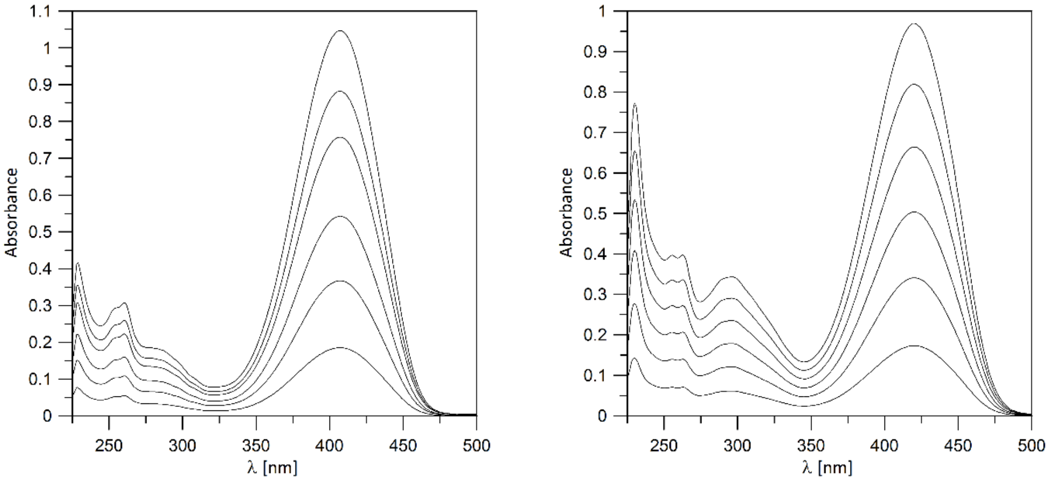

(**a**) (**b**)

Figure S121. UV–Vis spectrum in CH_2Cl_2. (**a**) diazaborine **7e** and (**b**) diazaborinone **7f**.

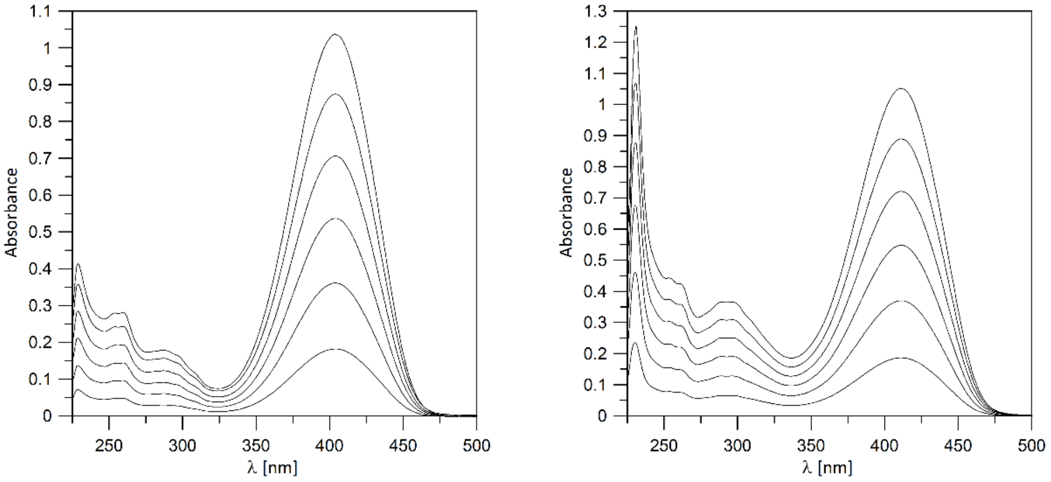

(**a**) (**b**)

Figure S122. UV–Vis spectrum in CH_2Cl_2. (**a**) diazaborine **7g** and (**b**) diazaborinone **7h**.

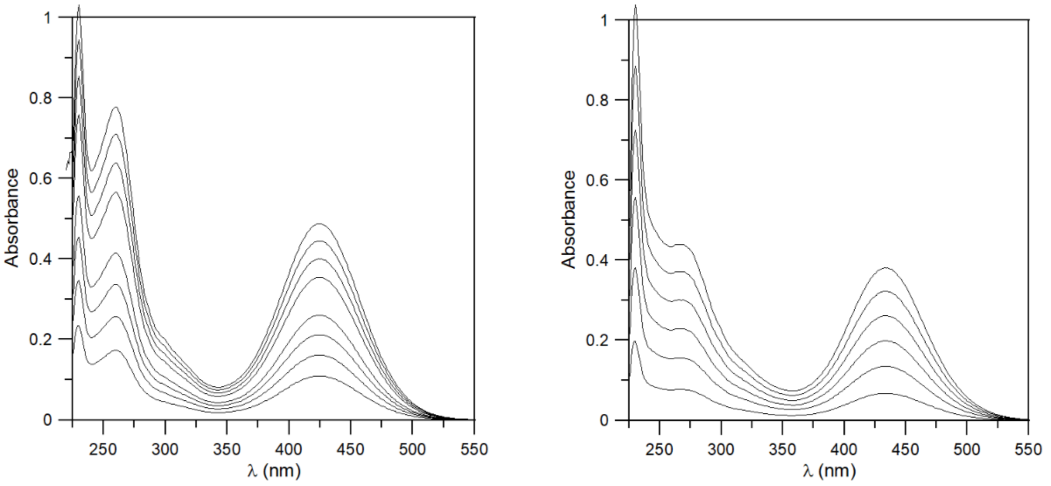

(**a**) (**b**)

Figure S123. UV–Vis spectrum in CH_2Cl_2. (**a**) triazaborine **8e** and (**b**) triazaborie **8h**.

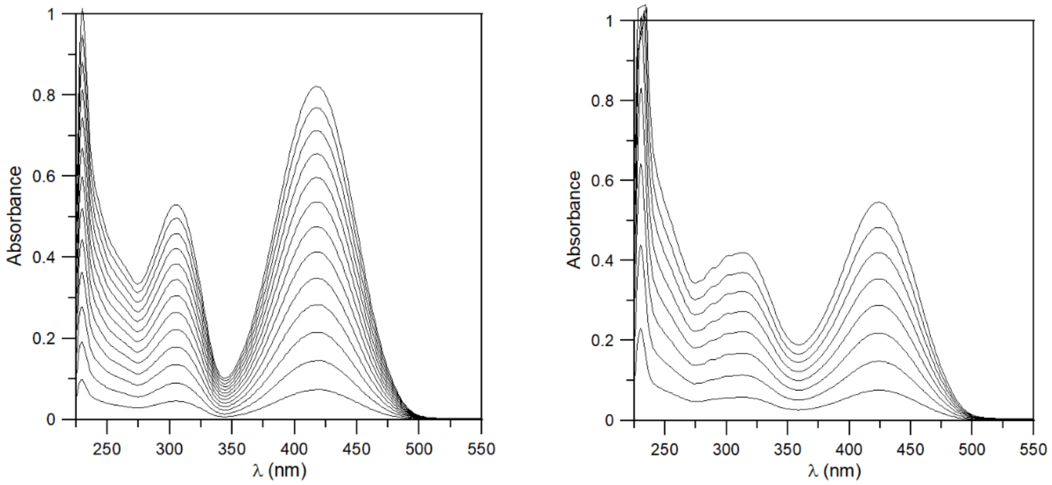

(**a**) (**b**)

Figure S124. UV–Vis spectrum in CH_2Cl_2. (**a**) oxazaborine–triazaborine **9g** and (**b**) oxazaborine–triazaborie **9h**.

Table S1. UV–Vis parameters of prepared heterocyclic compounds in CH_2Cl_2.

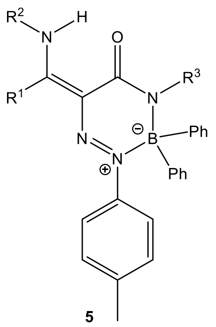

5

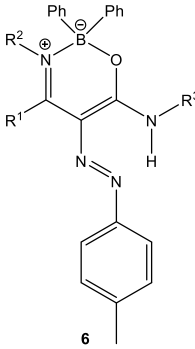

6

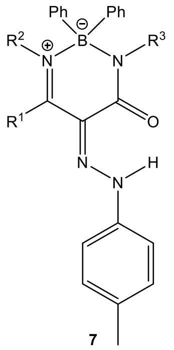

7

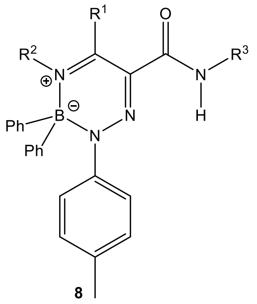

8

Compound	R^1	R^2	R^3	λ_{max} (nm)	ε $(l\cdot cm^{-1}\cdot mol^{-1})$
Triazaborinone **5a**	Me	H	H	401	21 908
Triazaborinone **5b**	Ph	H	H	416	25 105
Triazaborinone **5c**	Me	Me	H	405	26 250
Triazaborinone **5d**	Ph	Me	H	415	35 561
Triazaborinone **5g**	Me	Me	Me	411	26 641
Triazaborinone **5h**	Ph	Me	Me	421	24 867
Oxazaborine **6a**	Me	H	H	363	31 114
Oxazaborine **6e**	Me	H	Me	368	30 400
Oxazaborine **6f**	Ph	H	Me	371	27 476
Oxazaborine **6g**	Me	Me	Me	398	29 882
Oxazaborine **6h**	Ph	Me	Me	374	26 909
Diazaborinone **7a**	Me	H	H	403	39 358
Diazaborinone **7b**	Ph	H	H	415	37 224
Diazaborinone **7c**	Me	Me	H	400	40 031
Diazaborinone **7d**	Ph	Me	H	408	35 159
Diazaborinone **7e**	Me	H	Me	407	40 023
Diazaborinone **7f**	Ph	H	Me	420	36 707
Diazaborinone **7g**	Me	Me	Me	404	39 328
Diazaborinone **7h**	Ph	Me	Me	410	39 778
Triazaborine **8e**	Me	H	Me	424	11 714
Triazaborine **8g**	Me	Me	Me		
Triazaborine **8h**	Ph	Me	Me	434	14 541
Ozazaborine–triazaborine **9g**	Me	Me	Me	418	25 284
Ozazaborine–triazaborine **9h**	Ph	Me	Me	425	25 697

Table S2. UV–Vis parameters of triazaborine **5g**. λ_{max} (nm), ε (l·cm^{-1}mol^{-1})

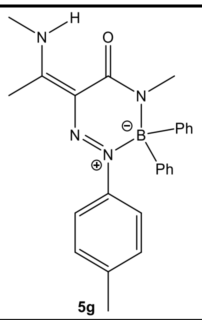

5g

Cyclohexane		Et_2O		CH_2Cl_2		THF		MeOH		Formamide	
λ_{max}	ε	λ_{max}	ε	λ_{max}	ε	λ_{max}	ε	λ_{max}	ε	λ_{max}	ε
412	21 450	409	22 509	411	26 641	409	22 676	405	22 371	408	6 292

Table S3. UV–Vis parameters of oxazaborine **6g**. λ_{max} (nm), ε (l·cm^{-1}mol^{-1}).

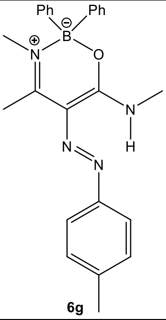

6g

Cyclohexane		Et_2O		CH_2Cl_2		THF		Acetonitril		Methanol	
λ_{max}	ε	λ_{max}	ε	λ_{max}	ε	λ_{max}	ε	λ_{max}	ε	λ_{max}	ε
395	28 600	394	27 789	398	29 882	396	29 174	394	26 841	393	27 196

Table S4. UV–Vis parameters of diazaborinone **7a**. λ_{max} (nm), ε (l·cm^{-1}mol^{-1}).

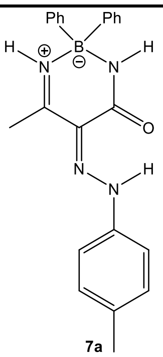

7a

CH_2Cl_2		THF		Acetonitril		Methanol	
λ_{max}	ε	λ_{max}	ε	λ_{max}	ε	λ_{max}	ε
403	39 358	398	38 398	395	38 592	392	19 064

4. Crystallographic data

Table S5. Crystal data.

Compound	5c	5d	5g	6a	6e	7f
Formula	$C_{24}H_{25}BN_4O$	$C_{29}H_{27}BN_4O \cdot 0.5\ C_7H_8$	$C_{25}H_{27}BN_4O$	$C_{23}H_{23}BN_4O$	$C_{24}H_{25}BN_4O$	$C_{29}H_{27}BN_4O$
M	396.29	504.42	410.31	382.26	396.29	458.36
System	Triclinic	Triclinic	Triclinic	Monoclinic	Monoclinic	Triclinic
Space Group	*P–1*	*P–1*	*P–1*	$P2_1/c$	$P2_1/n$	*P–1*
a/Å	9.7716(2)	9.5356(6)	9.1045(2)	10.0193(5)	14.3675(3)	6.3703(1)
b/Å	10.5152(2)	9.6497(4)	10.2052(3)	17.7580(12)	9.4464(2)	10.4288(2)
c/Å	12.2365(4)	16.2161(10)	12.4674(5)	24.0839(17)	16.5228(4)	19.0078(5)
α/°	82.6401(9)	96.902(3)	86.2535(9)	90	90	82.4168(10)
β/°	71.5304(10)	105.457(3)	75.3584(9)	92.7624(17)	105.1227(9)	89.1976(9)
γ/°	63.6440(11)	99.699(3)	88.8020(19)	90	90	80.3378(16)
U/Å^3	1068.47(5)	1396.05(14)	1118.36(6)	4280.1(5)	2164.84(8)	1233.93(5)
Z	2	2	2	8	4	2
D_c/g cm^{-3}	1.232	1.200	1.218	1.186	1.216	1.234
T/K	295	295	295	295	295	295
μ/cm^{-1}	0.77	0.73	0.75	0.74	0.76	0.76
θ_{min}–θ_{max}/°	3.9–27.8	2.9–28.0	3.7–27.0	2.0–24.0	3.4–27.0	1.1–27.8
Unique Refl.ns	4998	6708	4853	6451	4678	5793
R_{int}	0.017	0.049	0.022	0.084	0.021	0.021
Observed Refl.ns [I>2σ(I)]	3772	4215	3284	2577	3687	3827
R (Obs. Refl.ns)	0.0504	0.0677	0.0505	0.0746	0.0621	0.0474
wR (All Refl.ns)	0.1333	0.1653	0.1429	0.1934	0.1751	0.1310
S	1.042	1.026	1.024	1.045	1.031	1.025
$\Delta\varrho_{max}$; $\Delta\varrho_{min}$/e Å^{-3}	0.241; –0.195	0.362; –0.250	0.198; –0.207	0.158; –0.185	0.422; –0.220	0.185; –0.176
CCDC deposition number	2127610	2127611	2127612	2127613	2127614	2127615

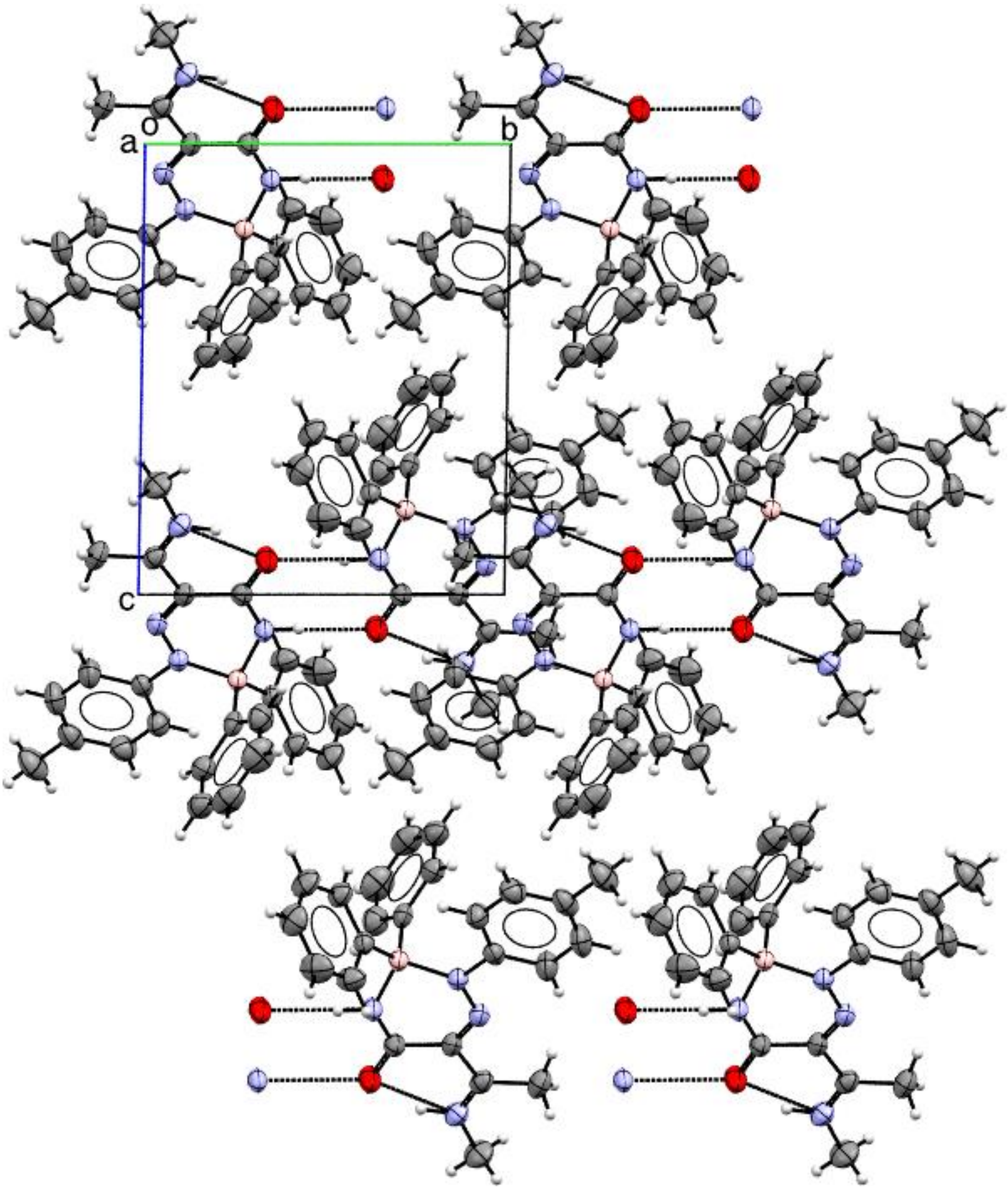

Figure S125. ORTEP view of the crystal structure of **5c** in the direction of the crystallographic axis *a*.

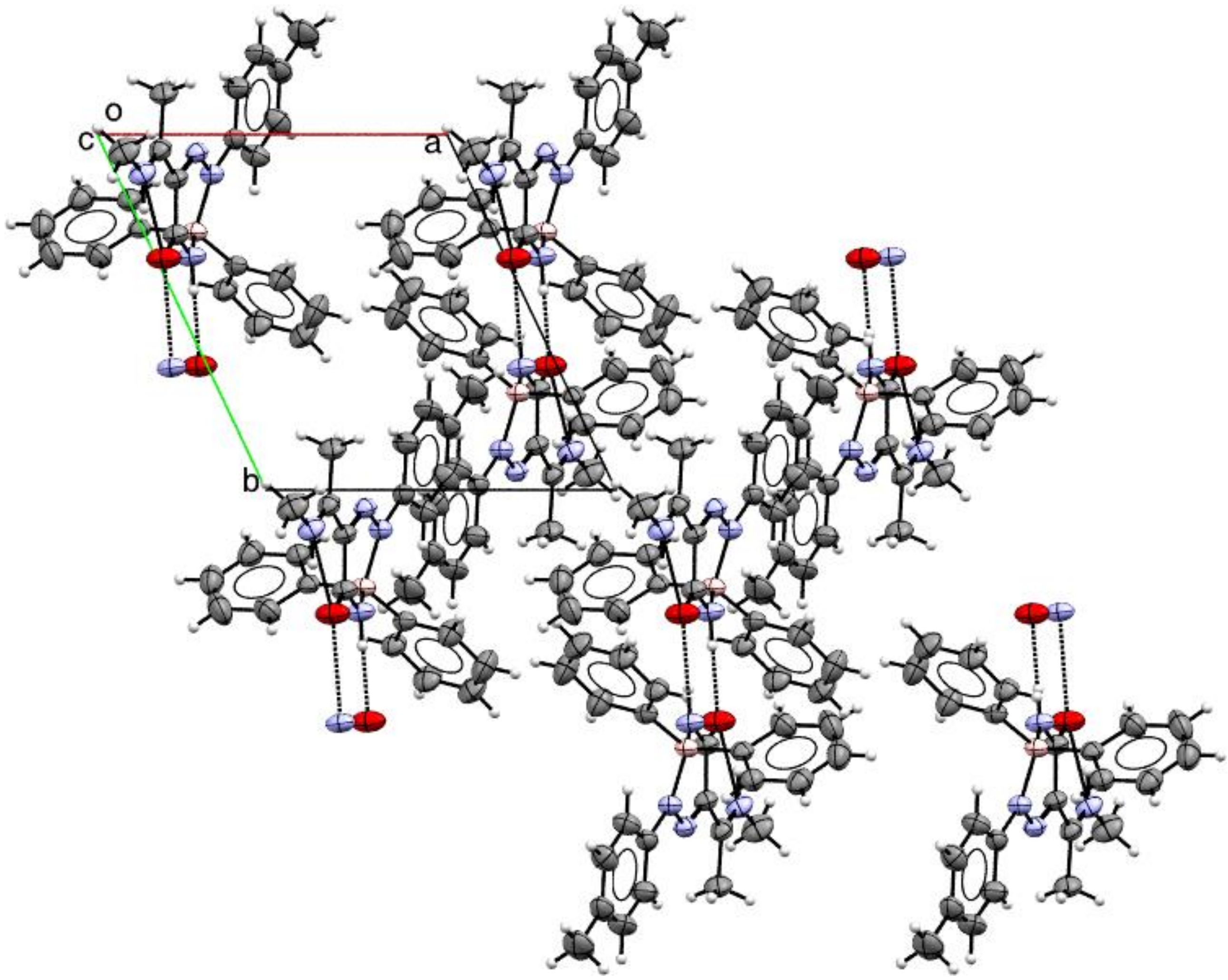

Figure S126. ORTEP view of the crystal structure of **5c** in the direction of the crystallographic axis *c*.

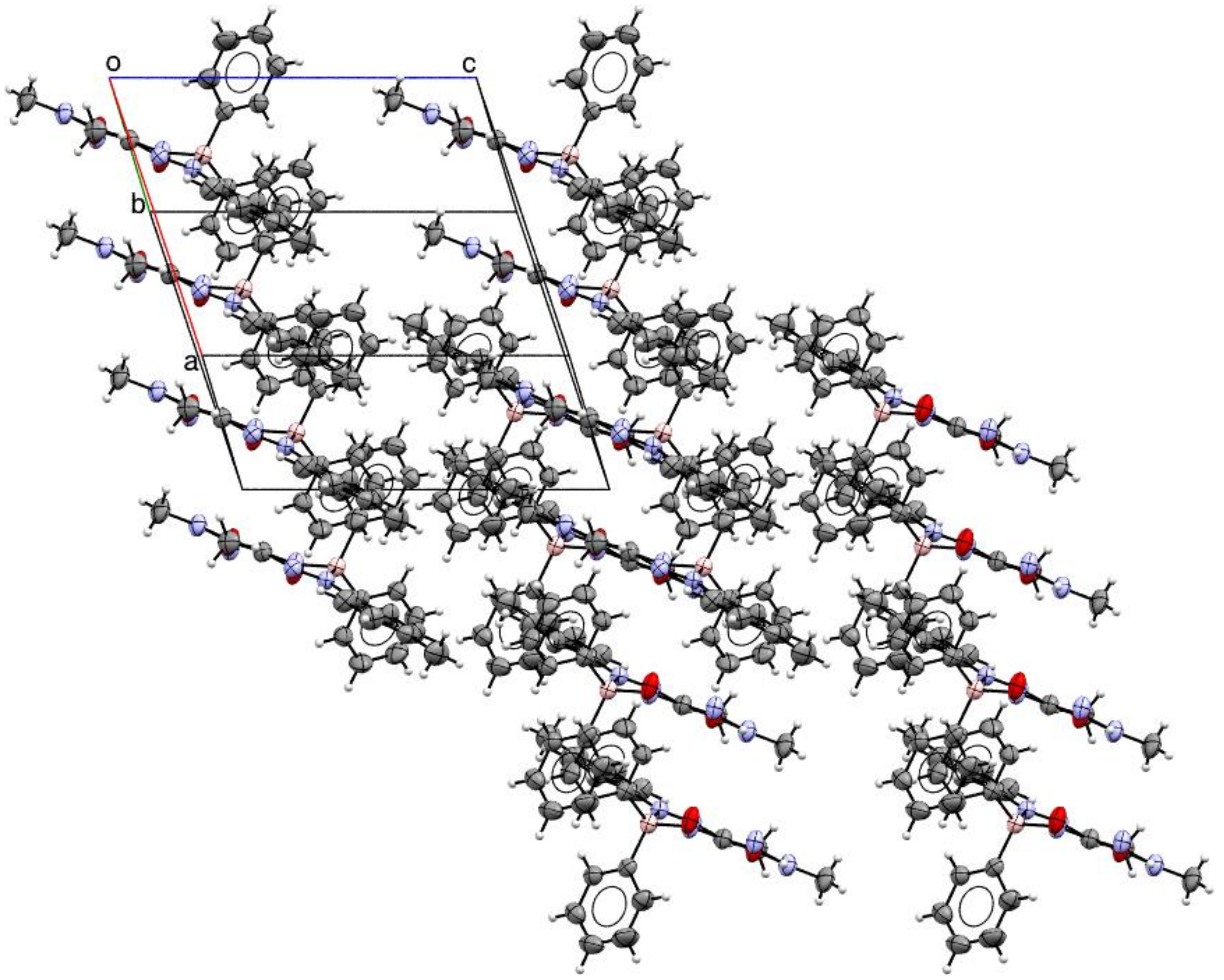

Figure S127. ORTEP view of the crystal structure of **5c** in the direction of the reciprocal cell axis *b**.

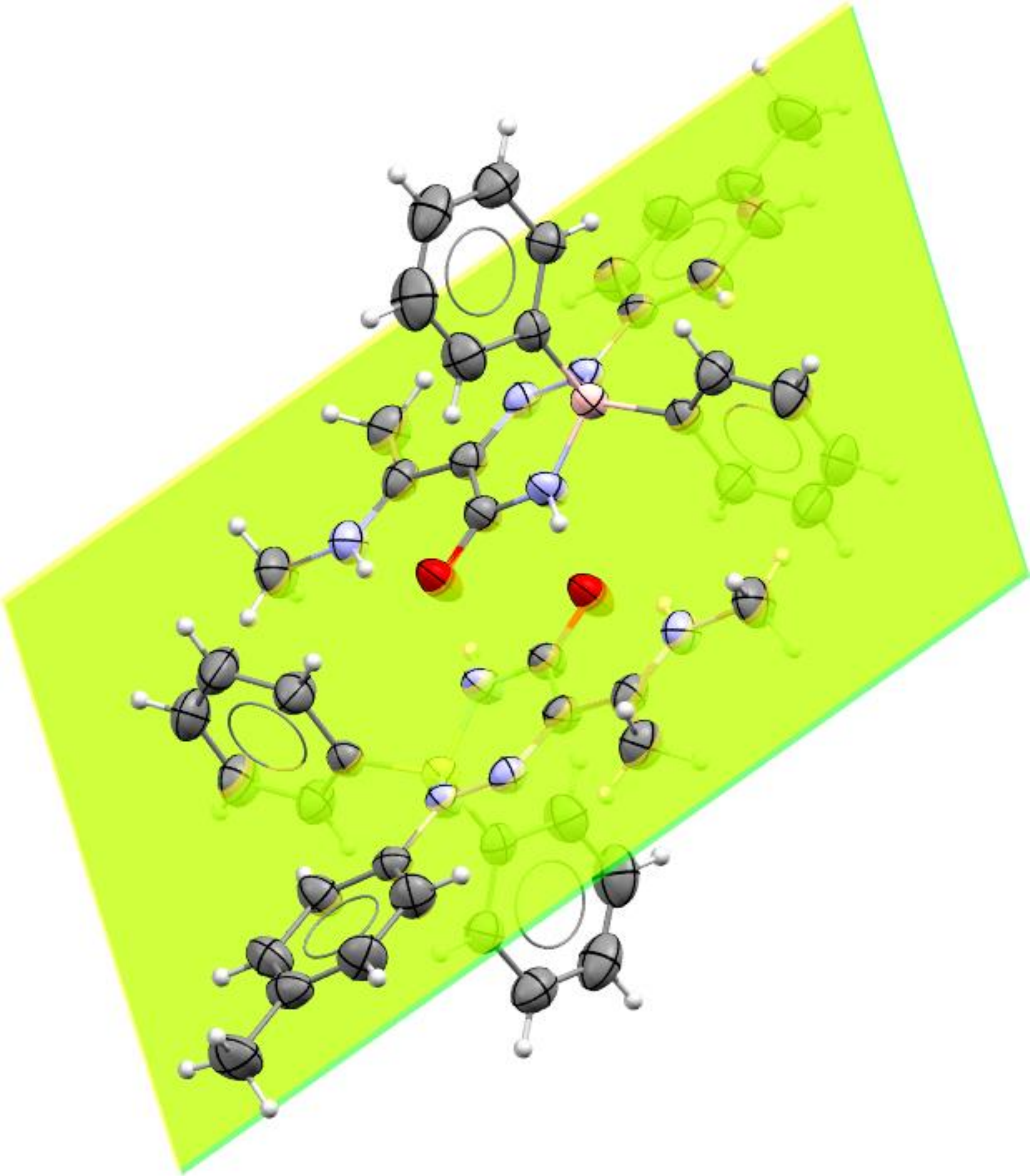

Figure S128. Triazaborine **5c**. The planes on which the double bonds C=N, C=N, and C=O lie.

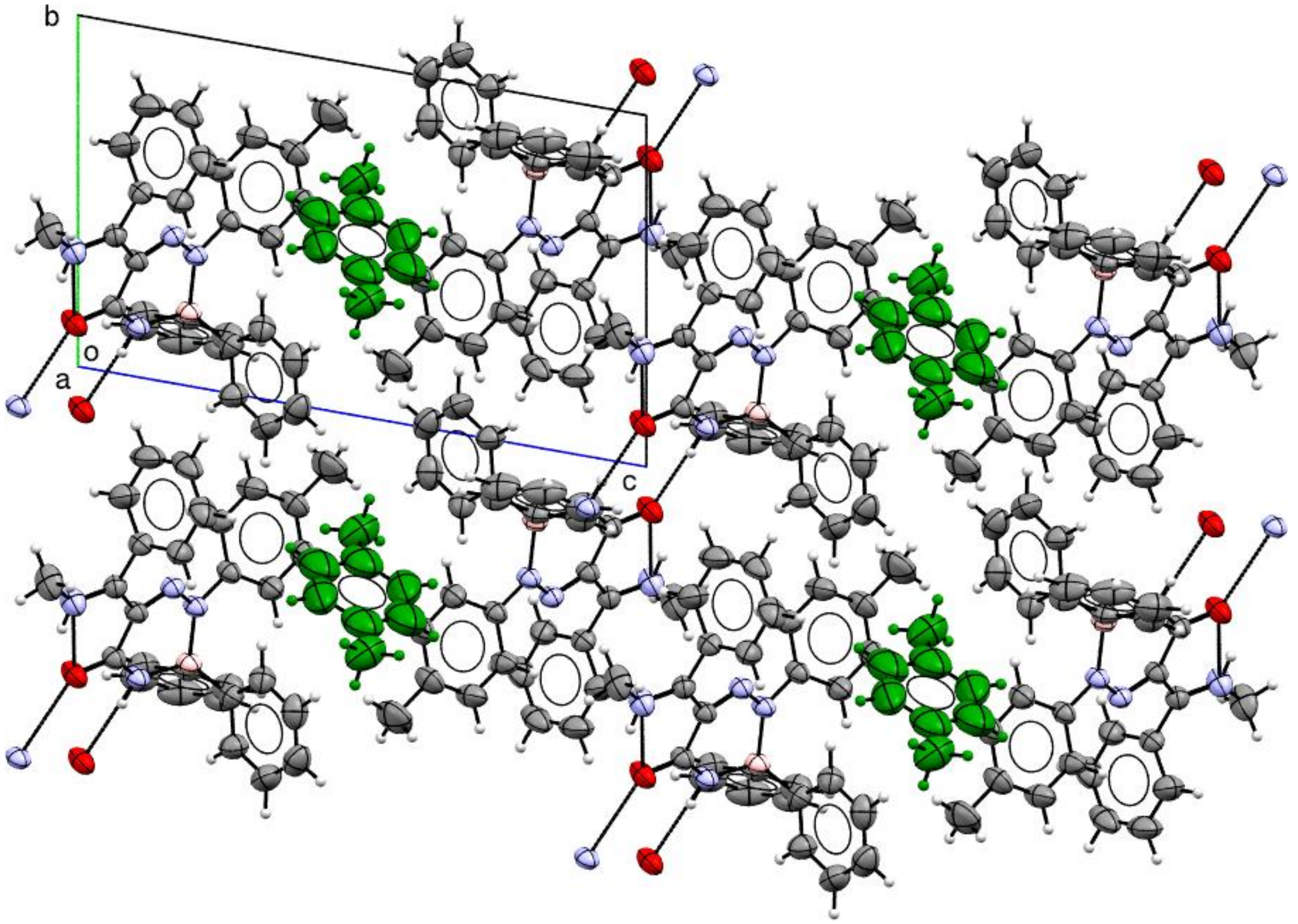

Figure S129. ORTEP view of the crystal packing of triazaborinone **5d** in the direction of the crystallographic axis *a*.

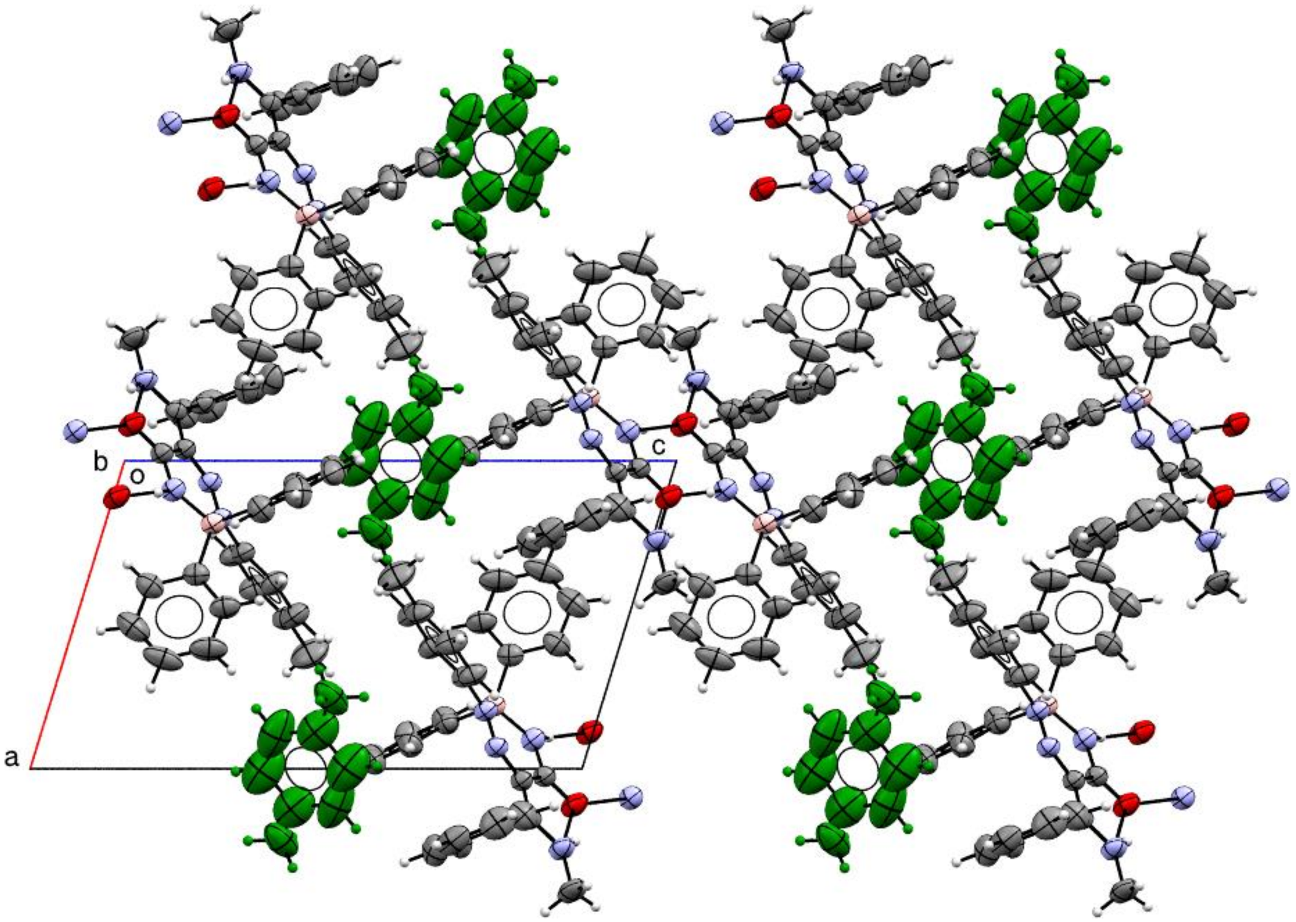

Figure S130. ORTEP view of the crystal packing of triazaborinone **5d** in the direction of the crystallographic axis *b*.

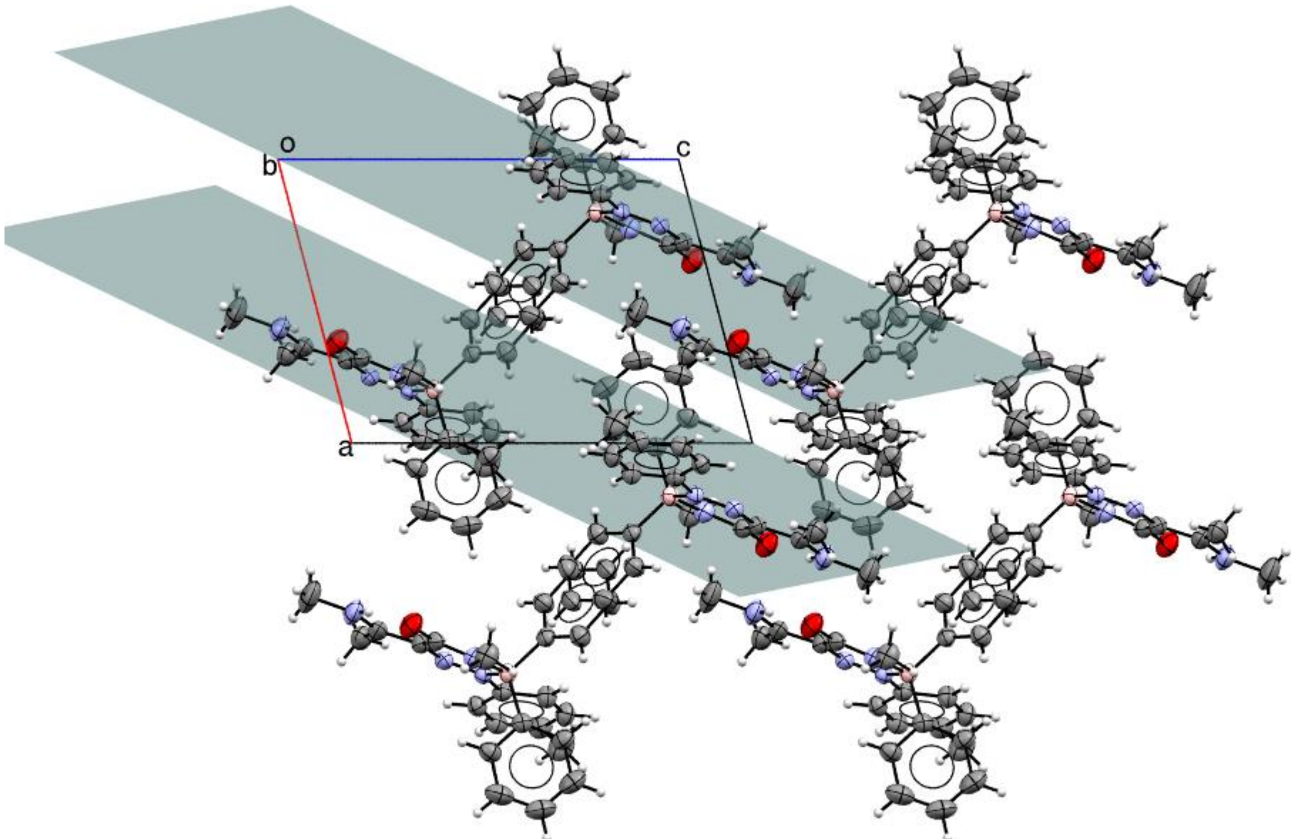

Figure S131. ORTEP view of the crystal structure of **5g** in the direction of the crystallographic axis *b*.

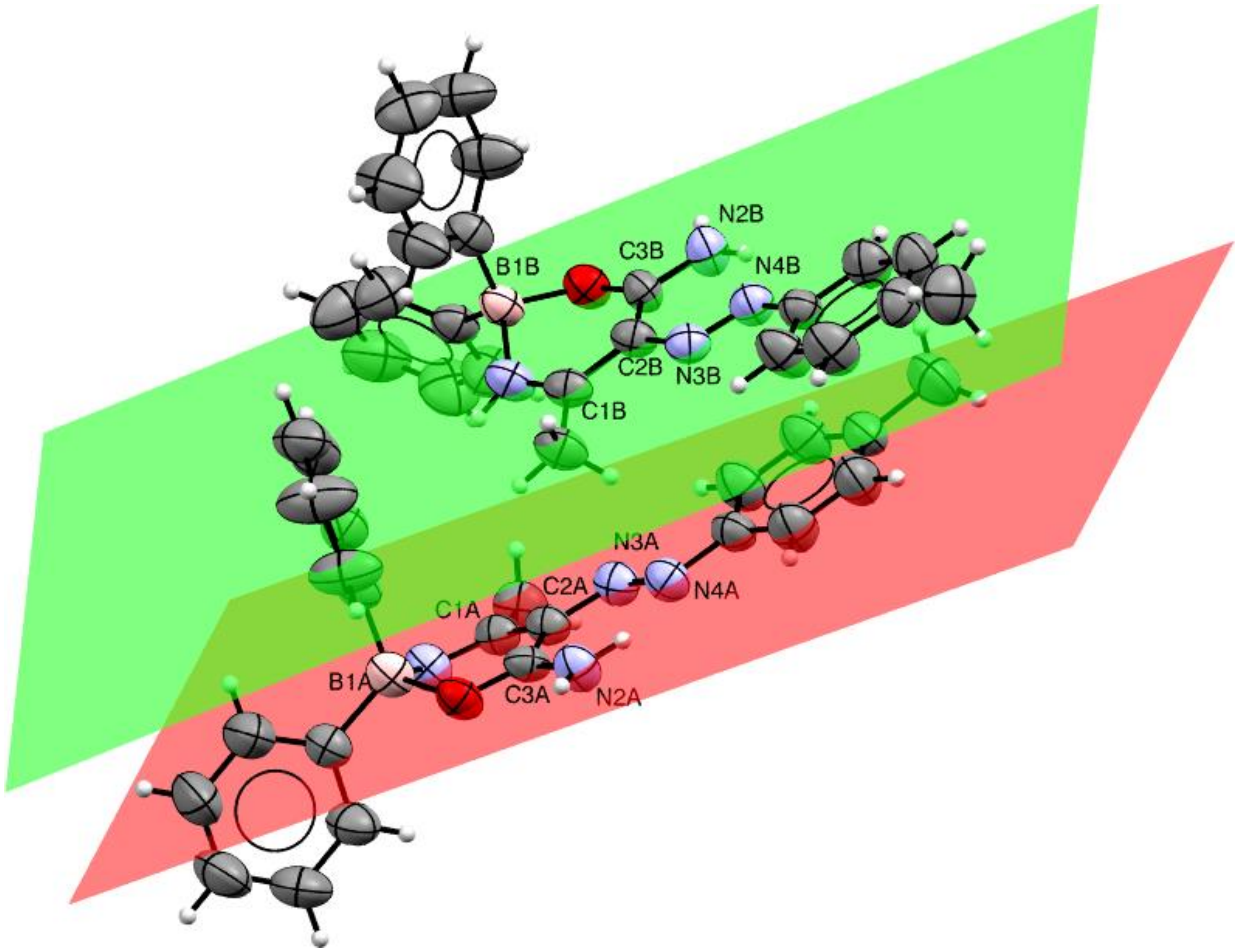

Figure S132. ORTEP diagram of the oxazaborine **6a** asymmetric unit.

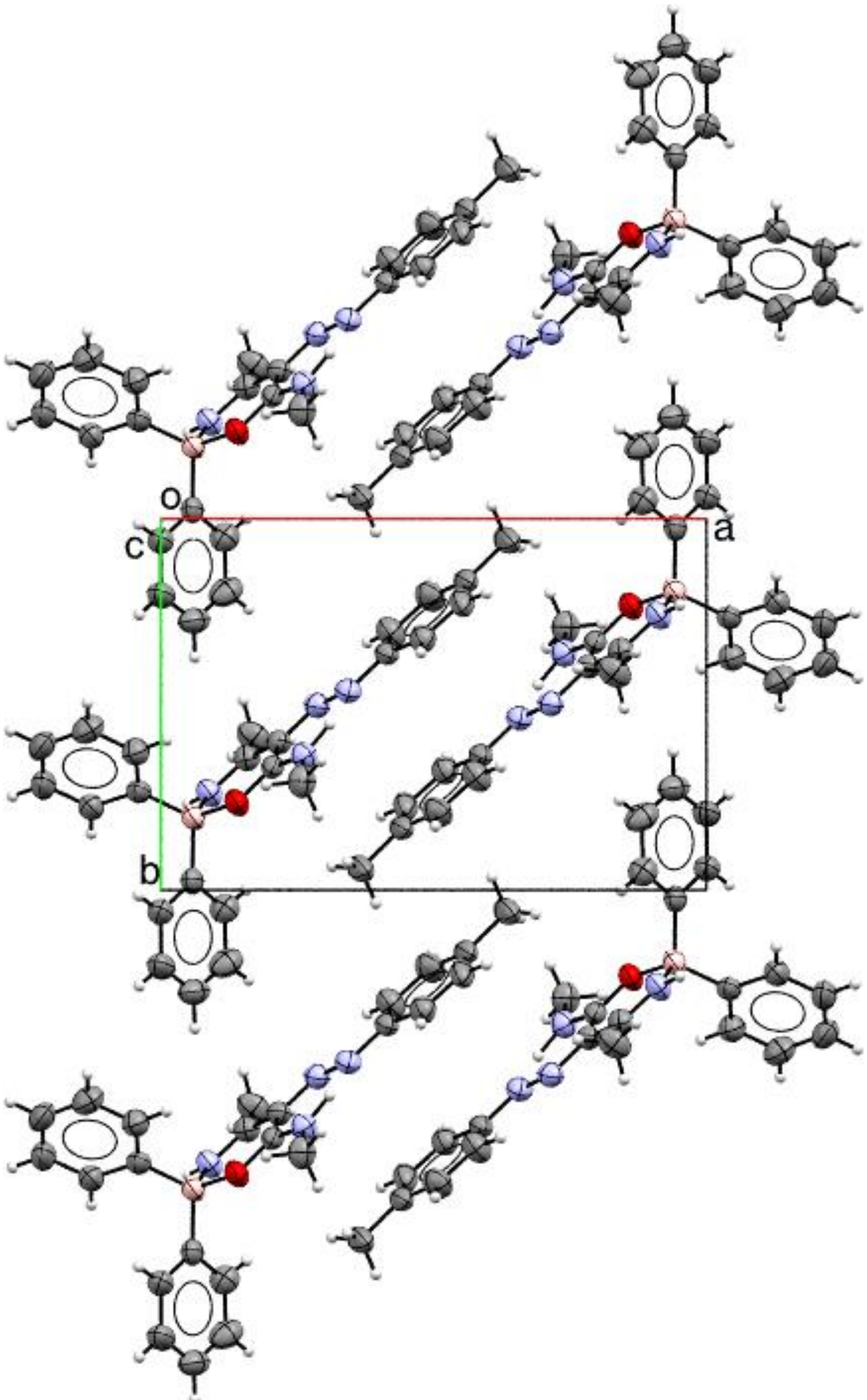

Figure S133. ORTEP view of the π–π stacking chain of **6e** in the direction of the crystallographic axis *c*.

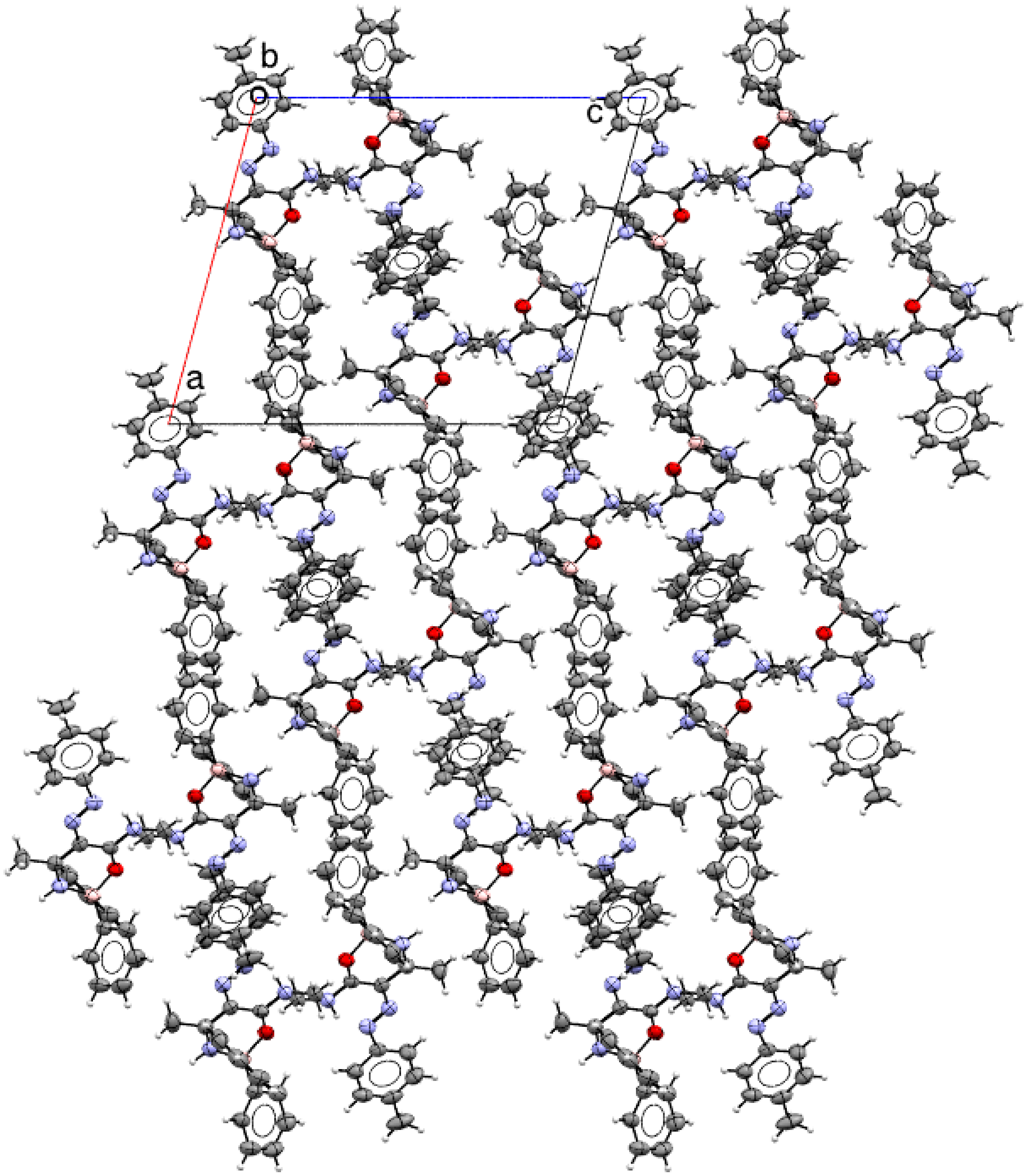

Figure S134. ORTEP view of the crystal structure of **6e** in the direction of the crystallographic axis *b*.

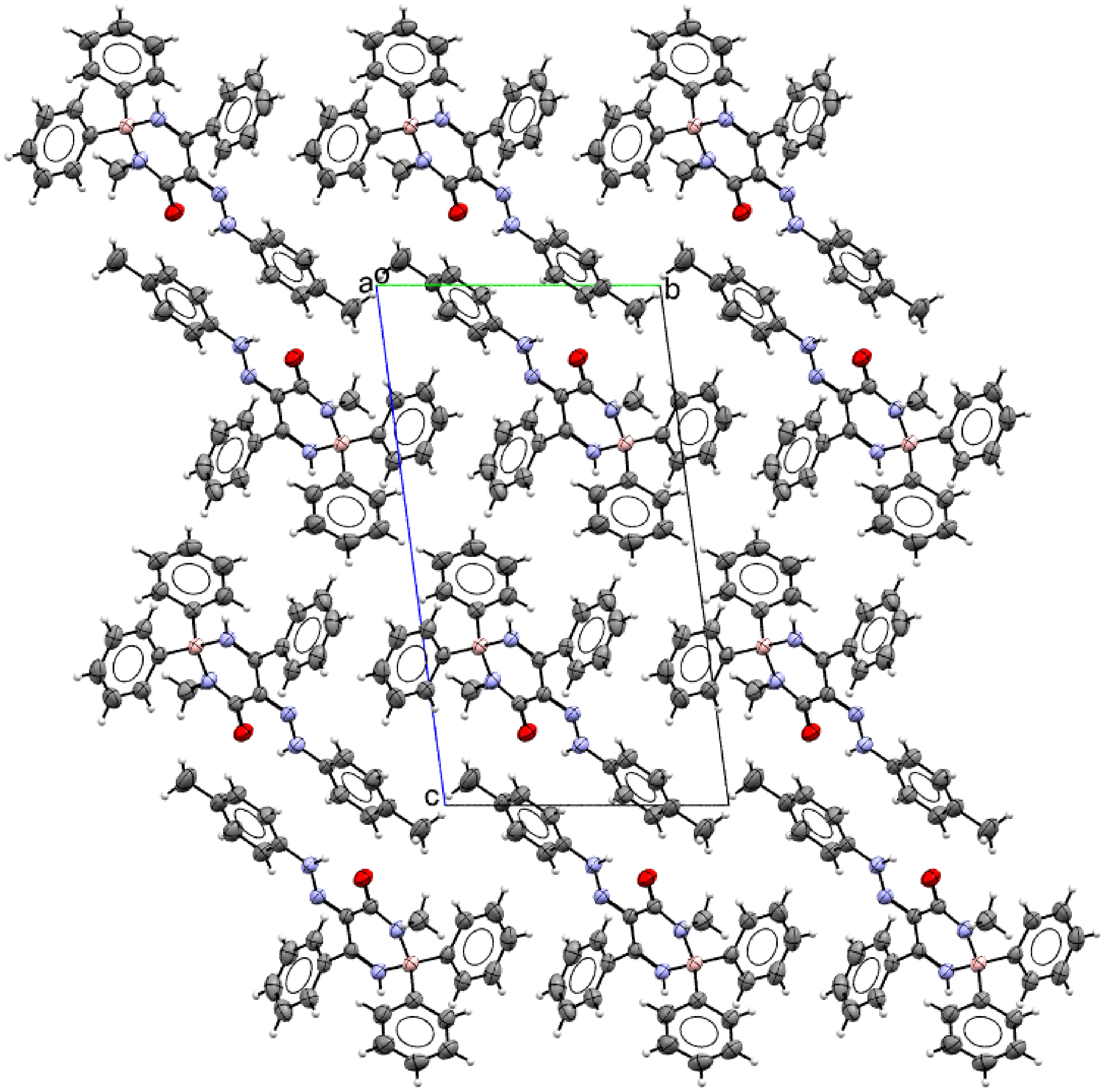

Figure S135. ORTEP view of the crystal packing of diazaborinone **7f** in the direction of the crystallographic axis *a*.

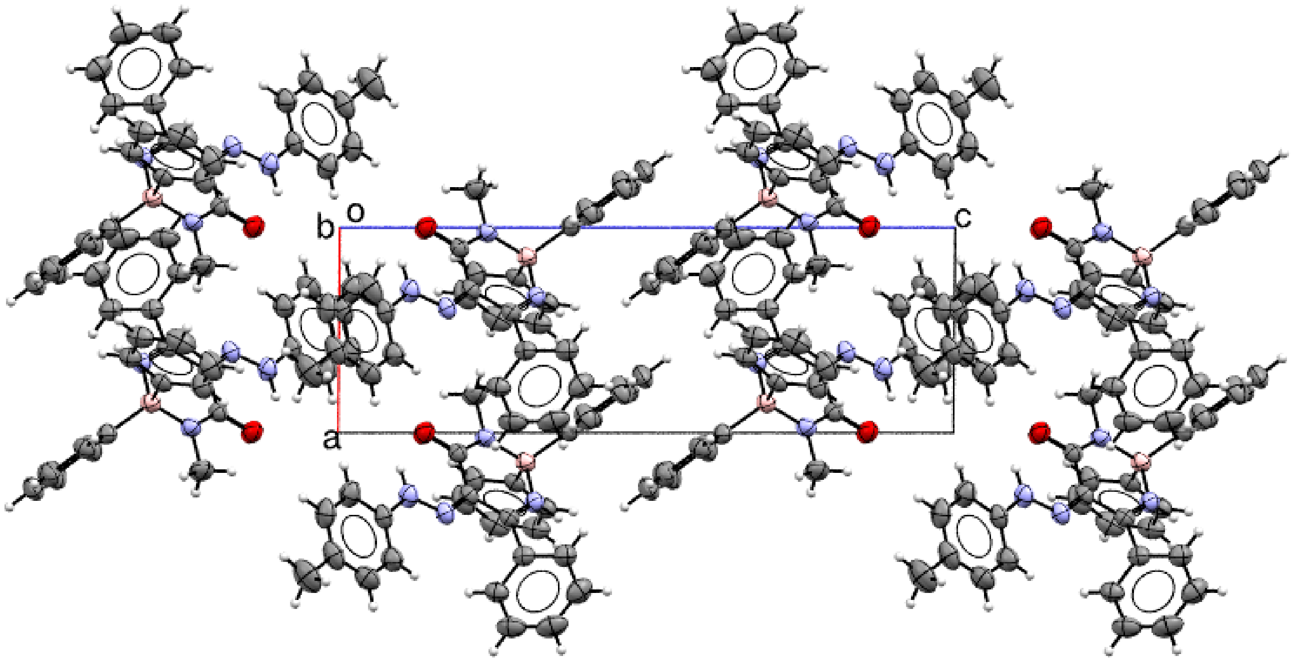

Figure S136. ORTEP view of the crystal packing of diazaborinone **7f** in the direction of the crystallographic axis *b*.

5. Fluorescence

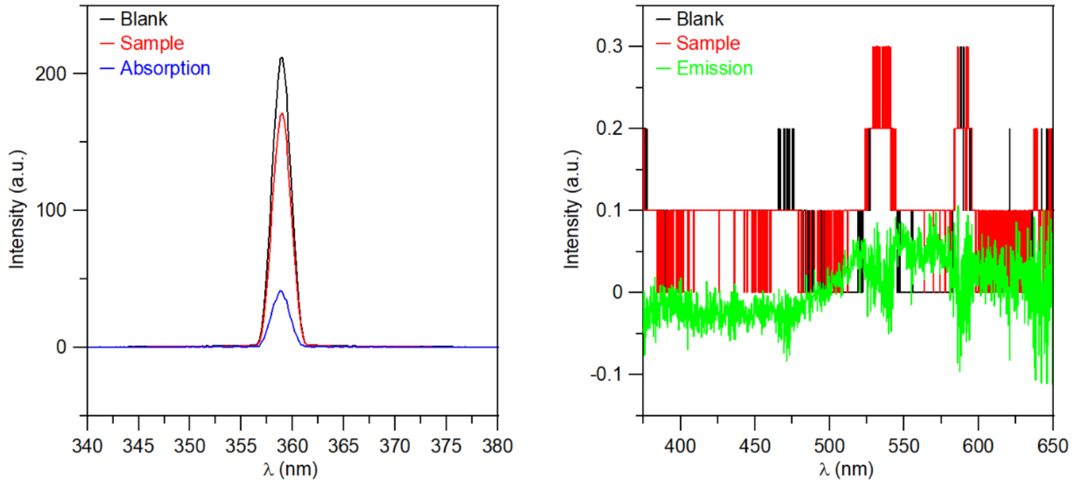

(**a**) (**b**)

Figure S137. Triazaborinone **5g**. (**a**) Absorption spectrum and (**b**) Emission spectrum.

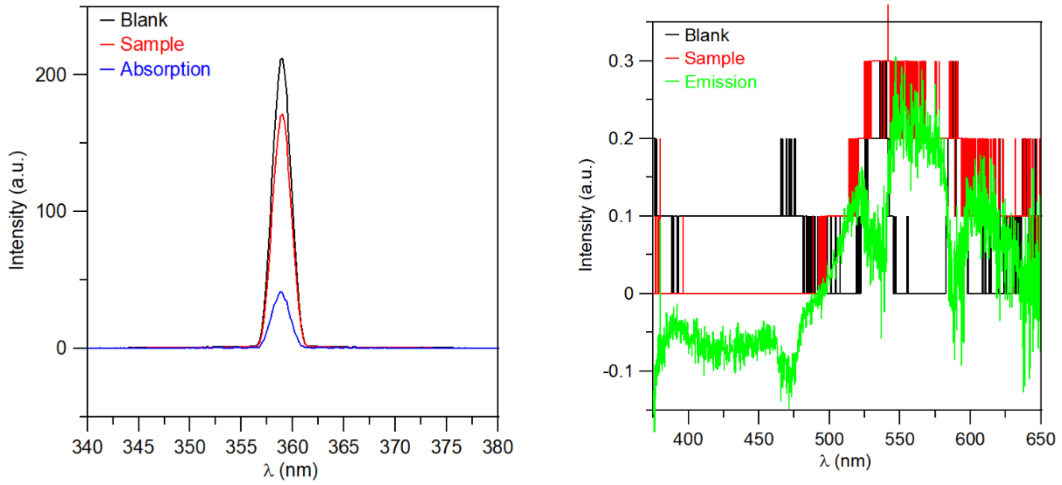

(**a**) (**b**)

Figure S138. Triazaborinone **5h**. (**a**) Absorption spectrum and (**b**) Emission spectrum.

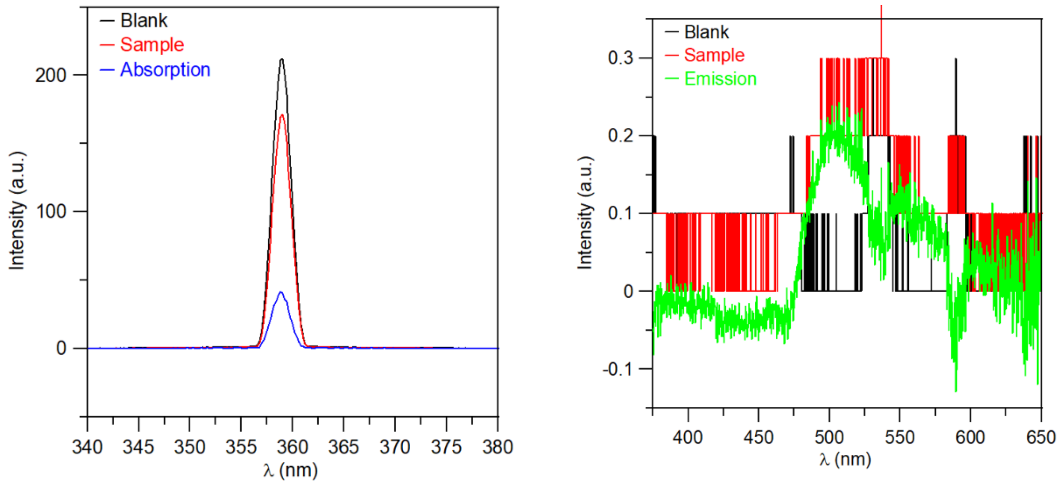

(**a**) (**b**)

Figure S139. Diazaborinone **7a**. (**a**) Absorption spectrum and (**b**) Emission spectrum.
